# Deaminative Cyanation of Anilines by Oxylanion Radical Transfer

**DOI:** 10.1021/acs.orglett.5c02410

**Published:** 2025-08-04

**Authors:** Deepak Behera, Tim Schulte, Ahmet Altun, Markus Leutzsch, Frank Neese, Tobias Ritter

**Affiliations:** † 28314 Max-Planck-Institut für Kohlenforschung, Kaiser-Wilhelm-Platz 1, Mülheim an der Ruhr 45470, Germany; ‡ Institute of Organic Chemistry, RWTH Aachen University, Landoltweg 1, 52074 Aachen, Germany

## Abstract

Herein, we report a straightforward methodology for direct deaminative cyanation of anilines via aryl diazonium salts as fleeting intermediates. The approach leverages the kinetic stability of nitrate and copper cyanide, iron’s ability to facilitate nitrate reduction, and appropriate relative rates to ensure the product-forming kinetic reaction pathway despite several thermodynamically favored, undesired reactions. We present insight into the previously unappreciated nitrate reduction mechanism by simple sulfur-based reductants, such as SO_2_. The oxylanion radical transfer mechanism is rarely encountered in synthetic chemistry but has ample precedent in biology and could provide a general, useful strategy for chemical nitrate reduction.

## Graphical abstract

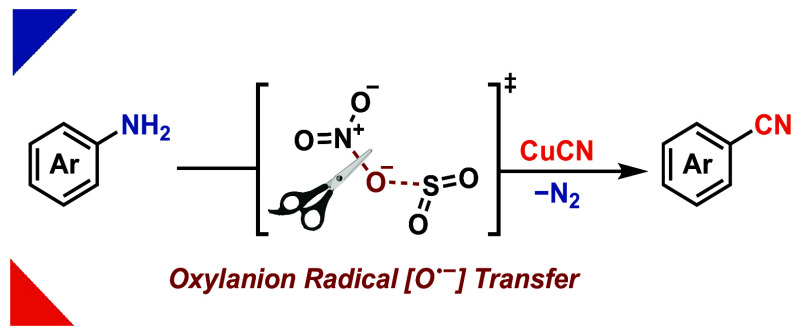

Benzonitriles play a crucial role in the production of dyes, agrochemicals, and pharmaceuticals.^1–5^ The Sandmeyer reaction^6^ is the classical method for synthesizing benzonitriles from isolated or accumulated potentially explosive aryl diazonium salts using copper­(I) cyanide (Figure 1), in two steps, possibly due to the incompatibility of nitrite with cyanide and Cu­(I) species.^7–10^ We have introduced methods for safer deaminative halogenation^11^ and sulfonylation^12^ based on nitrate reduction, also facilitated by iron nitrate. Utilization of iron species for deaminative cyanation is challenging because iron is effective at cyanide trapping to form hexacyanoferrate ion [Fe­(CN)_6_]^4–^,^13–15^ which should render cyanide unavailable for benzonitrile formation. Here we report the direct deaminative cyanation of anilines and aminoheterocycles. The process is enabled by the kinetic stability of both nitrate and Cu­(I) cyanide that avoids the otherwise thermodynamically favored but undesired oxidation of both Cu­(I) and cyanide and the undesired sequestration of cyanide by iron. Due to appropriate relative rates, nitrate reduction, diazonium formation, and cyanation can proceed productively, safer than previously, in a single step. The underlying oxylanion radical transfer (ORT) mechanism of the iron nitrate reduction was analyzed for a variety of sulfur-based anions, and it was found that the apparently simple SO_2_ + NO_3_^–^ redox transformation, which was first reported in 1892 ^16^ and had never been investigated in detail, also proceeds by ORT.

**Figure 1:**
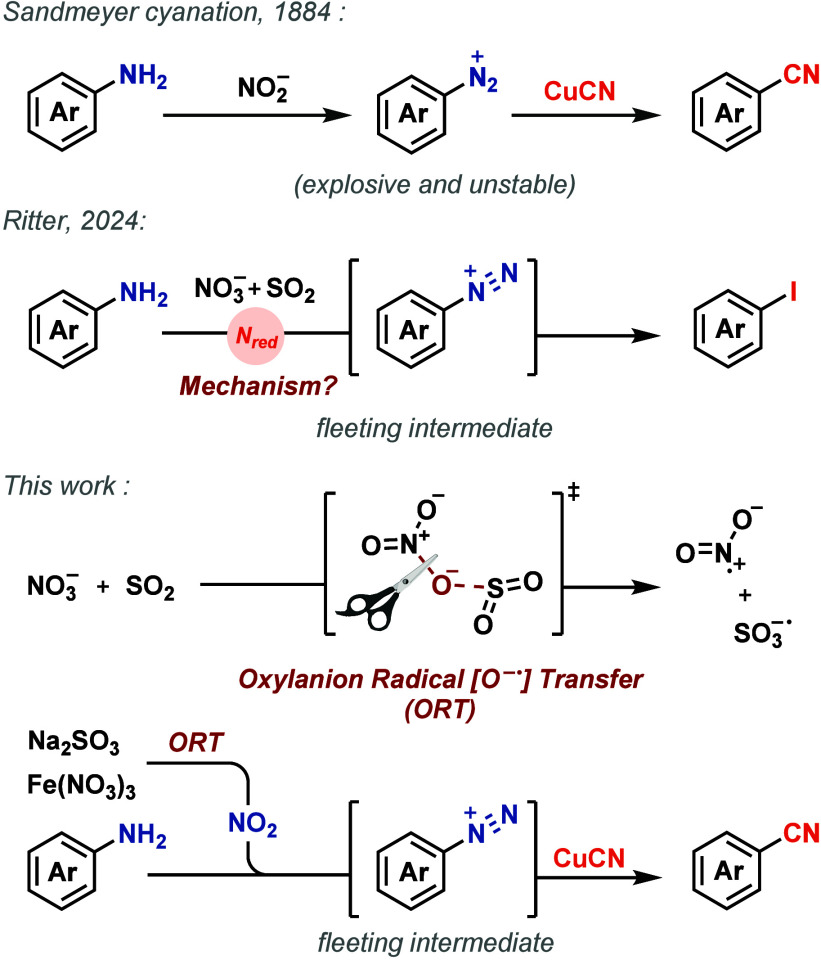
Sandmeyer cyanation, deaminative halogenation, and ORT-based nitrate reduction for deaminative cyanation.

The cyano group can be converted into a variety of other useful functional groups, such as carboxylic acids, amides, amines, tetrazoles, aldehydes, and ketones. Several methods for the preparation of nitriles have been reported, such as forcing dehydration of benzamides,^18–22^ the Rosenmund–von Braun reaction at temperatures between 150 and 250 °C,^23,24^ ammoxidation of toluene at 300–500 °C,^25–27^ and the Sandmeyer reaction.^6^ Synthesis of aryl diazonium salts with sensitive groups for the Sandmeyer reaction is often challenging due to the oxidative and highly acidic conditions. Additionally, aryl diazonium salts with electron-withdrawing groups can form reactive benzyne intermediates.^28,29^ Pd-catalyzed cross-coupling reactions of aryl diazonium salts with cyanide sources have been explored, yet cyanide can be a catalyst poison, which has complicated Pd-catalyzed cyanation.^30–32^ Diazonium cyanation typically involves the isolation or accumulation of potentially explosive aryl diazonium salts prepared from anilines and nitrites, followed by reaction with CuCN in a separate second step, because Cu­(I) and cyanide are incompatible with nitrite due to oxidation reactions.^7–10^ Zhang has shown that nitrite oxidizes copper­(I) to copper­(II) in the presence of HCl^10^ and Cu­(II) oxidizes cyanide to toxic cyanogen (N≡C–C≡N).^9^ Szymczak has also reported the oxidation of copper­(I) to copper­(II) in the presence of nitrite.^8^ Wei has reported that Cu_3_[Fe­(CN)_6_]_2_ reacts with nitrite to form [Fe­(CN)_5_NO]^2–^ and cyanogen gas.^7^ Combining the redox chemistry required for nitrate reduction with diazonium formation and Sandmeyer cyanation is therefore challenging.

Our group has recently reported nitrate reduction to achieve a variety of different deaminative transformations, such as halogenation^11^ with nitrate salts or a nitrate ester and sulfonylation with Fe­(III) nitrate.^12^ Nitrate reduction in the presence of iron was found to proceed via an ORT. ORT has been widely studied in biological systems. Nitrite reductases based on cytochrome *c* ^33^ and several other proteins such as hemoglobin,^34^ myoglobin,^35^ xanthine oxidoreductase,^36,37^ nitric oxide synthase,^38^ and cytochrome *c* oxidase^39,40^ reduce nitrite to NO via an ORT mechanism. However, the application of ORT for human-designed chemical reactions has not been well-explored^41^ and has mainly been limited to nitration reactions,^42–45^ such as the *ipso* nitration of boronic acids under photochemical irradiation.^46^ Thus, we believe that the development of new methods for nitrate reduction and the application in synthesis are desired by a broad range of synthetic chemists.

To identify further applications of ORT in organic synthesis, we aimed to apply the iron-based nitrate reduction to other deaminative transformations than sulfonylation. Here we show the deaminative cyanation of anilines and amino heterocycles as another application of Fe-mediated nitrate reduction. We present further experimental and computational evidence that the nitrate reduction with iron proceeds via ORT and demonstrate that the ORT mechanism is general over a variety of different sulfur-based reductants. Since the ORT mechanism was found to be operative for several sulfur-based anions, we analyzed our previously reported nitrate reduction with SO_2_, of which the mechanism had not been elucidated. We now provide experimental and computational evidence that this transformation also operates via an ORT mechanism.

We started our investigations by using the previously employed combination of iron nitrate and sodium thiosulfate,^12^ in the presence of copper­(I) cyanide, targeting the cyanation of darunavir at 25 °C. However, the reaction resulted in a less than 5% yield. We concluded that sodium thiosulfate is not an appropriate reductant in the presence of Fe­(III) and Cu­(I) cyanide. We found that the reductants sodium sulfite (Na_2_SO_3_), sodium bisulfite (NaHSO_3_), and sodium dithionite (Na_2_S_2_O_4_) can generate NO_2_ gas upon heating at 70 °C in the presence of iron­(III) nitrate, as evidenced by UV–vis spectroscopy (Figure S7) and gas-phase IR spectroscopy (Figure S8), presumably by acting as oxyl anion radical acceptors after single-electron oxidation by Fe­(III). All three salts provided the bezonitrile product in the presence of copper­(I) cyanide and aniline (pages S13 and S14 of the Supporting Information). This redox chemistry with thermodynamically strong oxidants proceeds in the presence of low-valent copper. Oxidation of Cu­(I) to Cu­(II) would be detrimental because (1) Cu­(I) is required for C–CN bond formation with aryldiazoniums and (2) Cu­(CN)_2_ is unstable toward cyanogen reductive elimination.^9^ Likewise, iron­(III) nitrate is required as the nitrate source because Fe­(III) can facilitate nitrate reduction,^12^ yet upon single-electron reduction, Fe­(II) is formed, which sequesters cyanide.^13^ We could not identify any other cyanide source besides copper cyanide that afforded the product in >5% yield, possibly because other cyanide sources react with Fe­(II). Despite the high formation constant from cyanide and Fe­(II),^13^ the formation of ferrocyanide was not observed with Cu­(I)­CN, possibly due to the covalent linear polymer structure of Cu­(I)­CN.^47^ Thus, Cu­(I)­CN remains available for ultimate oxidative ligation of the aryl radical generated upon single-electron aryldiazonium reduction for product-forming reductive elimination. The intricate network of redox steps required for the overall transformation proceeds productively, despite the opportunity for numerous other thermodynamically favorable redox steps that would each, if irreversible, render the deaminative cyanation transformation inefficient.

Under optimized conditions, a wide range of structurally and electronically diverse anilines and aminoheterocycles undergo direct deaminative cyanation reaction within approximately 12 h (Figure 2). Aminoheterocycles or functionalized anilines often pose challenges with conventional diazotation^48^ but are tolerated in our in situ protocol (**5**, **10**, and **20**). Anilines with bulky substituents at the *ortho* positions (**6**, **11**, and **12**) provide the product, as well.^49^ The protocol can be scaled; for example, benzonitrile **17** was prepared in 65% yield on a 5 g scale (19 mmol). As a brief but, of course, incomplete set, we show a small demonstration of the utility of the cyanodeamination to access a variety of other functional groups from cyanide (see pages S32–S36).

**Figure 2:**
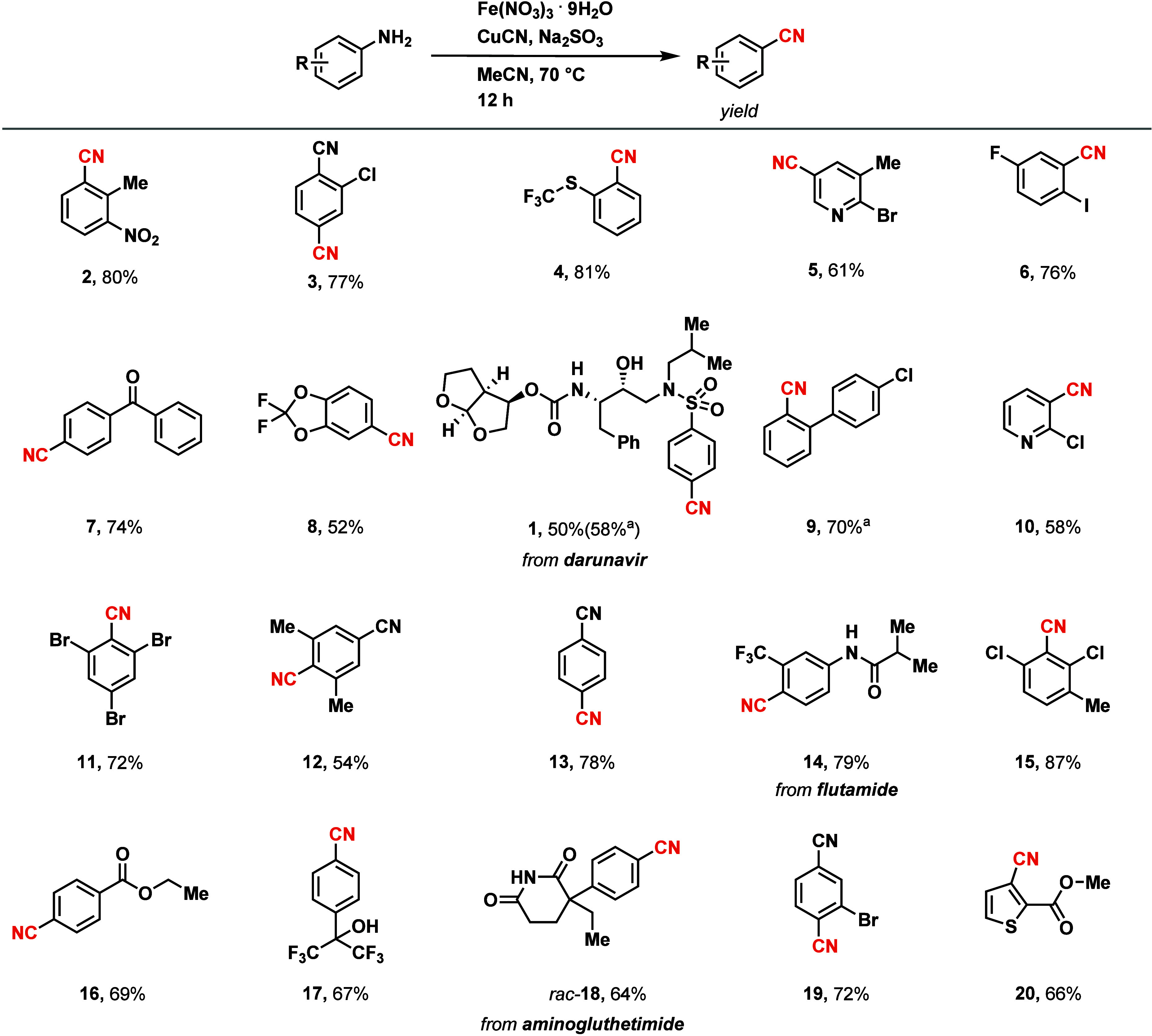
Substrate scope of the anilines. Reaction conditions: 0.500 mmol of aniline, 1.00 mmol of Fe­(NO_3_)_3_·9H_2_O, 1.00 mmol of CuCN, 0.600 mmol of Na_2_SO_3_, 1.25 mL of acetonitrile, 70 °C, 12 h. ^a^0.500 mmol of aniline, 1.00 mmol of Fe­(NO_3_)_3_·9H_2_O, 1.00 mmol of CuCN, 0.600 mmol of Na_2_S_2_O_4_, 1.25 mL of butyronitrile, 70 °C, 12 h.

To further analyze the mechanism of nitrate reduction with sodium dithionite (Na_2_S_2_O_4_), sodium sulfite (Na_2_SO_3_), and sodium bisulfite (NaHSO_3_), quantum chemical calculations were carried out (Figure 3). In the case of dithionate, Fe­(III) oxidizes dithionate to the dithionate radical anion (S_2_O_4_^•–^), which undergoes fragmentation to afford SO_2_ and the SO_2_ radical anion. Subsequently, SO_2_ can act as an oxylanion radical acceptor, to reduce nitrate to NO_2_. For sulfite and bisulfite, iron­(III) oxidizes both anions to form the sulfite radical anion (SO_3_^•–^), which can act as the oxylanion radical acceptor to reduce nitrate to NO_2_ without SO_2_ intermediacy, and generate sulfate, which was also detected by mass spectrometry (Figures S9 and S10). Our initially reported deaminative halogenation utilized SO_2_ as a chemically competent reductant for nitrate. However, the mechanism of the SO_2_ + nitrate reaction was not analyzed in detail. Based on the mechanistic data obtained for the nitrate reduction with sulfite, bisulfite, and dithionite, we were intrigued about whether the interaction of SO_2_ and nitrate also proceeds via an ORT mechanism. We started our investigation by analyzing the reaction of SO_2_ with nitrate by ^14^N, ^15^N, and ^17^O NMR spectroscopy. By ^14^N NMR spectroscopy, full conversion of ^14^NO_3_^–^ was observed after 45 min at 50 °C and the formation of NO_2_ was confirmed by EPR spectroscopy.^11^ No other new species causing a ^14^N NMR signal was detected. When the reaction was repeated at 80 °C with [18-cr-6-K]^15^NO_3_ and monitored by ^15^N NMR spectroscopy (Figure S14), which is less sensitive than ^14^N NMR spectroscopy with nonenriched material due to the lower natural abundance of the ^15^N isotope but produces sharper resonances due to the *S* = 1/2 nucleus of ^14^N, full conversion of ^15^N-nitrate was observed after 5 min. In the absence of SO_2_, nitrate was not consumed. Additionally, a colorless solid precipitated during the reactions, which mass spectrometric analysis identified as sulfate (page S47). The reaction was also monitored by ^17^O NMR spectroscopy with ^17^O-enriched (4–5 atom %) [15-cr-5-Na]­N^17^O_3_ (Figure S15). The formation of sulfate was observed by ^17^O NMR spectroscopy. The high signal intensity of the SO_2_ signal suggests that the detected SO_2_ is ^17^O-enriched, which could be due to rapid oxygen atom exchange between nitrate and SO_2_. Apart from sulfate and SO_2_, no other ^17^O-containing species were detected, which is consistent with the absence of any other sulfur-based product for the reaction of SO_2_ and nitrate to afford sulfate and NO_2_ that has a sufficient lifetime to be detected by ^17^O NMR spectroscopy. Sulfate (^17^OSO_3_^–^) containing one ^17^O atom was detected by HRMS (page S48), which is consistent with ORT. Quantum chemical calculations using the ORCA program package^50^ (Figure 3 and Figure S21) suggest that SO_2_ is acting as an oxylanion radical acceptor for nitrate. SO_2_ is oxidized to SO_3_^•–^, and nitrate is reduced to NO_2_ through ORT. The barrier of the oxylanion radical transfer was computationally determined to be 26.5 kcal/mol (Figure 3 and Table S14).

**Figure 3:**
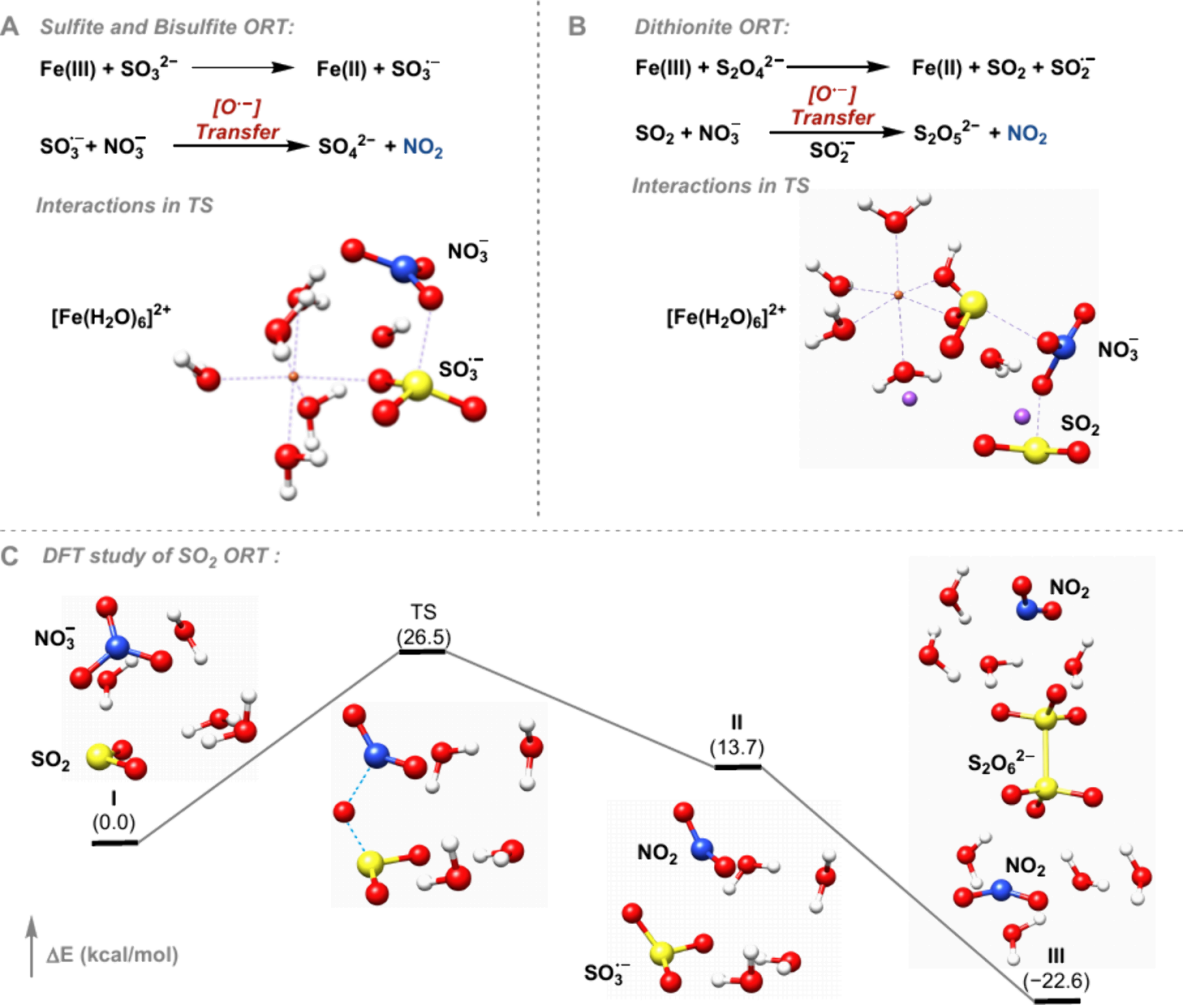
Oxylanion radical transfer (ORT) pathway was computed using coupled cluster singles and doubles with perturbative triples [CCSD­(T)] extrapolated to the complete basis set (CBS) limit (for details, see page S6).

The barrier for the rate-limiting nitrate reduction with SO_2_ based on experiment was estimated to be 27 kcal/mol.^11^ The barrier of the ORT process was found to be significantly lower if hydrogen bonding by four water molecules is considered in the calculations, underlining the importance of hydrogen bonding for the ORT process. The formed SO_3_^•–^ dimerizes to dithionate (S_2_O_6_^2–^) (eq 1), which is known to disproportionate to sulfate and SO_2_ (eq 2).^51^

$$2{{\mathrm{NO}}_{3}}^{-}+2{\mathrm{SO}}_{2}\to 2{\mathrm{NO}}_{2}+S_{2}{O_{6}}^{2-}$$ 1

2 $$S_{2}{O_{6}}^{2-}\to {{\mathrm{SO}}_{4}}^{2-}+{\mathrm{SO}}_{2}$$

In conclusion, we have demonstrated that iron nitrate can be used for nitrate reduction in the presence of various reductants, while the copper-based redox chemistry with cyanide is not influenced and neither is the availability of cyanide compromised. In that sense, the direct deaminative cyanation is fundamentally different from the previously published deaminohalogenation reactions and further expands the utility of safer diazonium chemistry with nitrate. We have presented experimental and computational data that are consistent with nitrate reduction by SO_2_ proceeding via an oxylanion radical transfer.

## Supplementary Material



## Data Availability

The data underlying this study are available in the published article and its Supporting Information.

## Notes

Experimental and computational procedures, spectroscopic data, NMR data, and NMR spectra of all products (PDF)

## Abbreviations

MeCN: acetonitrile
*n*-PrCN: butyronitrile
LAH: lithium aluminum hydride
DMF: dimethylformamide
DMSO: dimethyl sulfoxide

## References

[1] Liu J.-R., Gao Y., Jin B., Guo D., Deng F., Bian Q., Zhang H.-F., Han X.-Y., Ali A. S., Zhang M.-Z., Zhang W.-H., Gu Y.-C. (2023). Design, Synthesis, Antifungal Activity,
and Molecular Docking of Streptochlorin Derivatives Containing the
Nitrile Group. Mar. Drugs.

[2] Fatiadi A. J. (1983). Preparation
and synthetic applications of cyano compounds. Triple-Bonded Functional Groups (1983).

[3] Kluger, E. W. ; Burchette, J. T. Aromatic nitrile-containing compounds useful as dyestuff intermediates. US 4381265 A, 1983.

[4] Wang J., Liu H. (2012). Application of Nitrile
in Drug Design. Chin.
J. Org. Chem..

[5] Jiang H., Li H., Wang Y., Yu X., Chen X., Dai Y. (2023). Biodegradation
of the nitrile-containing insecticides sulfoxaflor, flonicamid, thiacloprid,
and acetamiprid by immobilized Escherichia coli harboring genes of
nitrile hydratase and a cobalt transporter. J. Environ. Chem. Eng..

[6] Sandmeyer T. (1884). Ueber die
Ersetzung der Amid-gruppe durch Chlor, Brom und Cyan in den aromatischen
Substanzen. Ber. Dtsch. Chem. Ges..

[7] Chen A., Li H., Wu H., Song Z., Chen Y., Zhang H., Pang Z., Qin Z., Wu Y., Guan X., Huang H., Li Z., Qiu G., Wei C. (2024). Anaerobic
cyanides oxidation with bimetallic modulation of biological toxicity
and activity for nitrite reduction. J. Hazard.
Mater..

[8] Moore C. M., Szymczak N. K. (2015). Nitrite reduction
by copper through ligand-mediated
proton and electron transfer. Chem. Sci..

[9] Parkash R., Zýka J. (1972). The reaction
between copper­(II) and cyanide ions. Microchem.
J..

[10] Bower J. K., Sokolov A. Y., Zhang S. (2019). Four-Coordinate Copper
Halonitrosyl
{CuNO}^10^ Complexes. Angew. Chem.,
Int. Ed..

[11] Mateos J., Schulte T., Behera D., Leutzsch M., Altun A., Sato T., Waldbach F., Schnegg A., Neese F., Ritter T. (2024). Nitrate reduction enables safer aryldiazonium chemistry. Science.

[12] Schulte T., Behera D., Carboni D., Höppner A., Waldbach F., Mateos J., Altun A., Leutzsch M., Krebs M. L., Ritter T. (2025). Iron-Mediated Nitrate
Reduction at
Ambient Temperature for Deaminative Sulfonylation and Fluorination
of Anilines. J. Am. Chem. Soc..

[13] Hollemann, A. F. ; Wiberg, N. Holleman-Wiberg Lehrbuch der Anorganische Chemie; Walter de Gruyter & Co.: Berlin, 2007; p 1649.

[14] Todd Z. R., Wogan N. F., Catling D. C. (2024). Favorable Environments for the Formation
of Ferrocyanide, a Potentially Critical Reagent for Origins of Life. ACS Earth Space Chem..

[15] Vogel, A. I. A Textbook of Macro and Semimicro Qualitative Inorganic Analysis, 4th ed.; Longmans, Green and Co. Ltd.: London, 1954; p 261.

[16] Hodgkinson W. R., Young J. (1892). Action of dry sulphur
dioxide on oxysalts. Chem. News.

[18] Šauliová J., Zmija A. R. (2003). Preparation
of Benzonitrile by Dehydration of Benzamide
with Phosphorus Pentoxide in Microwave Medium. Chem. Listy.

[19] Gagnon P.
E., Boivin J. L., Dickson J. H. (1959). THE DEHYDRATION OF UREA, BENZAMIDE,
AND PHENYLUREA BY THIONYL CHLORIDE IN THE PRESENCE OF AMMONIA. Can. J. Chem..

[20] Oxley P., Partridge M. W., Robson T. D., Short W. F. (1946). 156. Amidines. Part
II. Preparation of cyanides, amides, and amidines from carboxylic
acids. J. Chem. Soc. (Resumed).

[21] Stephens C. R., Bianco E. J., Pilgrim F. J. (1955). A New Reagent
for Dehydrating Primary
Amides Under Mild Conditions. J. Am. Chem. Soc..

[22] Ohmori H., Sakai K., Nagai N., Mizuki Y., Masui M. (1985). Reaction of
Electrochemically Generated Triphenylphosphine Radical Cation with
Amides and Ureas. Chem. Pharm. Bull..

[23] Rosenmund K. W., Struck E. (1919). Das am Ringkohlenstoff
gebundene Halogen und sein Ersatz
durch andere Substituenten. I. Mitteilung: Ersatz des Halogens durch
die Carboxylgruppe. Ber. Dtsch. Chem. Ges. (A
and B Series).

[24] Ito T., Watanabe K.-i. (1968). Studies of Organic Catalytic Reactions. VI. The Function
of Pyridine and Copper in the Rosenmund-von Braun Reaction. Bull. Chem. Soc. Jpn..

[25] Stevenson A. C. (1949). Ammonolysis. Ind. Eng. Chem..

[26] Rizayev R. G., Mamedov E. A., Vislovskii V. P., Sheinin V. E. (1992). Some fundamental
and practical aspects of the ammoxidation of alkylbenzenes. Appl. Catal. A: Gen..

[27] Cavalli P., Cavani F., Manenti I., Trifiro F. (1987). Ammoxidation of toluene
to benzonitrile on vanadium-titanium oxides catalysts prepared by
precipitation. The role of catalyst composition. Ind. Eng. Chem. Res..

[28] Nielsen M. A., Nielsen M. K., Pittelkow T. (2004). Scale-Up and
Safety Evaluation of
a Sandmeyer Reaction. Org. Process Res. Dev..

[29] Wulfman, D. S. , Patai, S. , Eds. In The Chemistry of Diazonium and DiazoGroups; Part 2: Synthetic Applications of Diazonium Ions; Wiley and Sons: New York, 1978; pp 251.

[30] Li J., Xu W., Ding J., Lee K.-H. (2016). The application of NCTS (N-cyano-N-phenyl-p-toluenesulfonamide)
in palladium-catalyzed cyanation of arenediazonium tetrafluoroborates
and aryl halides. Tetrahedron Lett..

[31] Xu W., Xu Q., Li J. (2015). Sandmeyer cyanation of arenediazonium tetrafluoroborate
using acetonitrile as a cyanide source. Org.
Chem. Front..

[32] Ahmad M. S., Shafiq Z., Meguellati K. (2022). Palladium-Catalyzed Cyanation of
Arenediazonium Tetrafluoroborate Derivatives with 2-(Piperidin-1-yl)­acetonitrile
as the Cyano Source. Synthesis.

[33] Basu S., Azarova N. A., Font M. D., King S. B., Hogg N., Gladwin M. T., Shiva S., Kim-Shapiro D. B. (2008). Nitrite
Reductase Activity of Cytochrome c*. J. Biol.
Chem..

[34] Cosby K., Partovi K. S., Crawford J. H., Patel R. P., Reiter C. D., Martyr S., Yang B. K., Waclawiw M. A., Zalos G., Xu X., Huang K. T., Shields H., Kim-Shapiro D. B., Schechter A. N., Cannon R. O., Gladwin M. T. (2003). Nitrite reduction
to nitric oxide by deoxyhemoglobin vasodilates the human circulation. Nat. Med..

[35] Shiva S., Huang Z., Grubina R., Sun J., Ringwood L. A., MacArthur P. H., Xu X., Murphy E., Darley-Usmar V. M., Gladwin M. T. (2007). Deoxymyoglobin Is a Nitrite Reductase
That Generates
Nitric Oxide and Regulates Mitochondrial Respiration. Circ. Res..

[36] Millar T. M., Stevens C. R., Benjamin N., Eisenthal R., Harrison R., Blake D. R. (1998). Xanthine oxidoreductase
catalyses
the reduction of nitrates and nitrite to nitric oxide under hypoxic
conditions. FEBS Lett..

[37] Li H., Samouilov A., Liu X., Zweier J. L. (2004). Characterization
of the Effects of Oxygen on Xanthine Oxidase-mediated Nitric Oxide
Formation*. J. Biol. Chem..

[38] Mikula I., Durocher S., Martasek P., Mutus B., Slama-Schwok A. (2009). Isoform-specific
differences in the nitrite reductase activity of nitric oxide synthases
under hypoxia. Biochem. J..

[39] Castello P. R., David P. S., McClure T., Crook Z., Poyton R. O. (2006). Mitochondrial
cytochrome oxidase produces nitric oxide under hypoxic conditions:
Implications for oxygen sensing and hypoxic signaling in eukaryotes. Cell Metab..

[40] Ball K. A., Nelson A. W., Foster D. G., Poyton R. O. (2012). Nitric
oxide produced
by cytochrome c oxidase helps stabilize HIF-1α in hypoxic mammalian
cells. Biochem. Biophys. Res. Commun..

[41] Salvitti C., Bandeira N. A. G., Pepi F., de Petris G., Troiani A. (2023). Sulfur Dioxide Oxidation by Zinc and Zinc Oxide Nitrate/Nitrite
Complexes in the Gas Phase: An Interplay between Redox-Active Ligands
and Metal. Chem. - Eur. J..

[42] Taniguchi T., Fujii T., Ishibashi H. (2010). Iron-Mediated
Radical Halo-Nitration
of Alkenes. J. Org. Chem..

[43] Jiang M., Yang H., Li Y., Jia Z., Fu H. (2013). Efficient
ipso-nitration of arylboronic acids with iron nitrate as the nitro
source. RSC Adv..

[44] Qian Y.-E., Zheng L., Xiang H.-Y., Yang H. (2021). Recent progress in
the nitration of arenes and alkenes. Org. Biomol.
Chem..

[45] Patra S., Mosiagin I., Giri R., Nauser T., Katayev D. (2023). Electron-Driven
Nitration of Unsaturated Hydrocarbons. Angew.
Chem., Int. Ed..

[46] Liu S., Lu Y., Wang H., Xue Z., Xu Z., Wan H., Yin Q., Lv T., Liu S.-X., Jin Y. (2025). Iron-Catalyzed Ipso-Nitration
of Aryl Borides via Visible-Light-Induced β-Homolysis. ACS Catal..

[47] Kroeker S., Wasylishen R. E., Hanna J. V. (1999). The Structure of Solid Copper­(I)
Cyanide: A Multinuclear Magnetic and Quadrupole Resonance Study. J. Am. Chem. Soc..

[48] Ghiazza C., Faber T., Gómez-Palomino A., Cornella J. (2022). Deaminative
chlorination of aminoheterocycles. Nat. Chem..

[49] Joshi S., Vyas S., Duffel M., Parkin S., Lehmler H.-J. (2011). Synthesis
of sterically hindered polychlorinated biphenyl derivatives. Synthesis.

[50] Neese F. (2022). Software update:
The ORCA program system—Version 5.0. Wiley Interdiscip. Rev.: Comput. Mol. Sci..

[51] Hollemann, A. F. ; Wiberg, N. Holleman-Wiberg Lehrbuch der Anorganische Chemie; Walter de Gruyter & Co.: Berlin, 2007; p 595.

