# Supplementary material for: Deaminative Cyanation of Anilines by Oxylanion Radical Transfer
Source: Org Lett. 2025 Aug 4;27(32):8921–6. doi: 10.1021/acs.orglett.5c02410 (PMC12362591; doi:10.1021/acs.orglett.5c02410)
Supplement: Supplementary file 1 [file ol5c02410_si_001.pdf]

SUPPLEMENTARY MATERIALS

Deaminative Cyanation of Anilines by Oxylanion Radical Transfer

Deepak Behera,[1,2,‡] Tim Schulte,[1,‡] Ahmet Altun,[1] Markus Leutzsch,[1] Frank Neese,[1] and Tobias Ritter*[1]

1 Max-Planck-Institut für Kohlenforschung; Mülheim an der Ruhr, D-45470, Germany.

2 Institute of Organic Chemistry, RWTH Aachen University; Aachen, 52074, Germany.

‡ The authors contributed equally.

*Corresponding author. Email: ritter@kofo.mpg.de

TABLE OF CONTENTS

MATERIALS AND METHODS 5

EXPERIMENTAL DATA 8

General procedures for deaminative cyanation 8

Optimization tables 9

Aniline Scope for Deaminative Cyanation 13

Darunavir-derived benzonitrile **1** 13
2-Methyl-3-nitrobenzonitrile (**2**) 14
2-Chloroterephthalonitrile (**3**) 15
2-(Trifluoromethylthio)benzonitrile (**4**) 16
6-Bromo-5-methylnicotinonitrile (**5**) 17
5-Fluoro-2-iodobenzonitrile (**6**) 17
4-Benzoylbenzonitrile (**7**) 18
2,2-Difluorobenzo[d][1,3]dioxole-5-carbonitrile (**8**) 19
4'-Chloro-[1,1'-biphenyl]-2-carbonitrile (**9**) 20
2-Chloronicotinonitrile (**10**) 20
2,4,6-Tribromobenzonitrile (**11**) 21
2,6-Dimethylterephthalonitrile (**12**) 22
Terephthalonitrile (**13**) 22
Reduced flutamide **S1** 23
Flutamide-dervied benzonitrile **14** 24
2,6-Dichloro-3-methylbenzonitrile (**15**) 25
Ethyl 4-cyanobenzoate (**16**) 25
4-(1,1,1,3,3,3-Hexafluoro-2-hydroxypropan-2-yl)benzonitrile (**17**) 26
rac-Aminoglutethimide-dervied benzonitrile **18** 27
2-Bromoterephthalonitrile (**19**) 28
Methyl 3-cyanothiophene-2-carboxylate (**20**) 28

Gram scale reaction 30

4-(1,1,1,3,3,3-Hexafluoro-2-hydroxypropan-2-yl)benzonitrile (**17**) 30

Diversifications after Deaminative Cyanation 32

2-(4-(Aminomethyl)phenyl)-1,1,1,3,3,3-hexafluoropropan-2-ol (**22**) 32
2-(4-(1H-tetrazol-5-yl)phenyl)-1,1,1,3,3,3-hexafluoropropan-2-ol (**23**) 33
4-(1,1,1,3,3,3-Hexafluoro-2-hydroxypropan-2-yl)benzamide (**24**) 34
4-(1,1,1,3,3,3-Hexafluoro-2-hydroxypropan-2-yl)benzoic acid (**25**) 35

Preparation of [18-crown-6-K]$^{15}NO_3$ and [15-crown-5-Na]$N^{17}O_3$ 37

[18-crown-6-K]$^{15}NO_3$ 37
[15-crown-5-Na]$N^{17}O_3$ 37

Mechanism Experiments 39

NO_2 formation from iron nitrate with different SO_2 surrogates 39
NO_2 detection with UV-Vis spectroscopy 39
NO_2 detection with gas phase IR spectroscopy 40
Diazonium trapping experiment 41
Mass spectrometric analysis 43
GC-MS of vial headspace 45
Reaction monitoring by ^{15}N NMR spectroscopy 47
Reaction monitoring by ^{17}O NMR spectroscopy 47

COMPUTATIONAL DATA 50

Nitrate reduction with Na_2SO_3 50
Nitrate reduction with $NaHSO_3$ 53
Nitrate reduction with $Na_2S_2O_4$ 57
Cyanation from aryldiazonium salt 63
NO_2 release from nitrate ion and SO_2 68
Water-assisted bisulfate ion formation from $SO_3^{\bullet-}$ 73

SPECTROSCOPIC DATA 76

^1H NMR spectrum of Darunavir-derived benzonitrile **1** 76
^{13}C NMR spectrum of Darunavir-derived benzonitrile **1** 77
^1H NMR spectrum of 2-methyl-3-nitrobenzonitrile (**2**) 78
^{13}C NMR spectrum of 2-methyl-3-nitrobenzonitrile (**2**) 79
^1H NMR spectrum of 2-chloroterephthalonitrile (**3**) 80
^{13}C NMR spectrum of 2-chloroterephthalonitrile (**3**) 81
^1H NMR spectrum of 2-(trifluoromethylthio)benzonitrile (**4**) 82
^{19}F NMR spectrum of 2-(trifluoromethylthio)benzonitrile (**4**) 83
^{13}C NMR spectrum of 2-(trifluoromethylthio)benzonitrile (**4**) 84
^1H NMR spectrum of 6-bromo-5-methylnicotinonitrile (**5**) 85
^{13}C NMR spectrum of 6-bromo-5-methylnicotinonitrile (**5**) 86
^1H NMR spectrum of 5-fluoro-2-iodobenzonitrile (**6**) 87
^{19}F NMR spectrum of 5-fluoro-2-iodobenzonitrile (**6**) 88
^{13}C NMR spectrum of 5-fluoro-2-iodobenzonitrile (**6**) 89

^{1}H NMR spectrum of 4-benzoylbenzonitrile (**7**) 90

^{13}C NMR spectrum of 4-benzoylbenzonitrile (**7**) 91

^{1}H NMR spectrum of 2,2-difluorobenzo[d][1,3]dioxole-5-carbonitrile (**8**) 92

^{19}F NMR spectrum of 2,2-difluorobenzo[d][1,3]dioxole-5-carbonitrile (**8**) 93

^{13}C NMR spectrum of 2,2-difluorobenzo[d][1,3]dioxole-5-carbonitrile (**8**) 94

^{1}H NMR spectrum of 4'-chloro-[1,1'-biphenyl]-2-carbonitrile (**9**) 95

^{13}C NMR spectrum of 4'-chloro-[1,1'-biphenyl]-2-carbonitrile (**9**) 96

^{1}H NMR spectrum of 2-chloronicotinonitrile (**10**) 97

^{13}C NMR spectrum of 2-chloronicotinonitrile (**10**) 98

^{1}H NMR spectrum of 2,4,6-tribromobenzonitrile (**11**) 99

^{13}C NMR spectrum of 2,4,6-tribromobenzonitrile (**11**) 100

^{1}H NMR spectrum of 2,6-dimethylterephthalonitrile (**12**) 101

^{13}C NMR spectrum of 2,6-dimethylterephthalonitrile (**12**) 102

^{1}H NMR spectrum of terephthalonitrile (**13**) 103

^{13}C NMR spectrum of terephthalonitrile (**13**) 104

^{1}H NMR spectrum of Flutamide-derived benzonitrile **14** 105

^{19}F NMR spectrum of Flutamide-derived benzonitrile **14** 106

^{13}C NMR spectrum of Flutamide-derived benzonitrile **14** 107

^{1}H NMR spectrum of 2,6-dichloro-3-methylbenzonitrile (**15**) 108

^{13}C NMR spectrum of 2,6-dichloro-3-methylbenzonitrile (**15**) 109

^{1}H NMR spectrum of ethyl 4-cyanobenzoate (**16**) 110

^{13}C NMR spectrum of ethyl 4-cyanobenzoate (**16**) 111

^{1}H NMR spectrum of 4-(1,1,1,3,3,3-hexafluoro-2-hydroxypropan-2-yl)benzonitrile (**17**) 112

^{19}F NMR spectrum of 4-(1,1,1,3,3,3-hexafluoro-2-hydroxypropan-2-yl)benzonitrile (**17**) 113

^{13}C NMR spectrum of 4-(1,1,1,3,3,3-hexafluoro-2-hydroxypropan-2-yl)benzonitrile (**17**) 114

^{1}H NMR spectrum of *rac*-Aminoglutethimide-derived benzonitrile **18** 115

^{13}C NMR spectrum of *rac*-Aminoglutethimide-derived benzonitrile **18** 116

^{1}H NMR spectrum of 2-bromoterephthalonitrile (**19**) 117

^{13}C NMR spectrum of 2-bromoterephthalonitrile (**19**) 118

^{1}H NMR spectrum of methyl 3-cyanothiophene-2-carboxylate (**20**) 119

^{13}C NMR spectrum of methyl 3-cyanothiophene-2-carboxylate (**20**) 120

^{1}H NMR spectrum of 4-(aminomethyl)phenyl)-1,1,1,3,3,3-hexafluoropropan-2-ol (**22**) 121

^{19}F NMR spectrum of 4-(aminomethyl)phenyl)-1,1,1,3,3,3-hexafluoropropan-2-ol (**22**) 122

^{13}C NMR spectrum of 4-(aminomethyl)phenyl)-1,1,1,3,3,3-hexafluoropropan-2-ol (**22**) 123

^{1}H NMR spectrum of 2-(4-(1H-tetrazol-5-yl)phenyl)-1,1,1,3,3,3-hexafluoropropan-2-ol (**23**) 124

^{19}F NMR spectrum of 2-(4-(1H-tetrazol-5-yl)phenyl)-1,1,1,3,3,3-hexafluoropropan-2-ol (**23**) 125

^{13}C NMR spectrum of 2-(4-(1H-tetrazol-5-yl)phenyl)-1,1,1,3,3,3-hexafluoropropan-2-ol (**23**) 126

^{1}H NMR spectrum of 4-(1,1,1,3,3,3-hexafluoro-2-hydroxypropan-2-yl)benzamide (**24**) 127

^{19}F NMR spectrum of 4-(1,1,1,3,3,3-hexafluoro-2-hydroxypropan-2-yl)benzamide (**24**) 128

^{13}C NMR spectrum of 4-(1,1,1,3,3,3-hexafluoro-2-hydroxypropan-2-yl)benzoic acid (**24**) 129

^{1}H NMR spectrum of 4-(1,1,1,3,3,3-hexafluoro-2-hydroxypropan-2-yl)benzoic acid (**25**) 130

^{19}F NMR spectrum of 4-(1,1,1,3,3,3-hexafluoro-2-hydroxypropan-2-yl)benzoic acid (**25**) 131

^{13}C NMR spectrum of 4-(1,1,1,3,3,3-hexafluoro-2-hydroxypropan-2-yl)benzoic acid (**25**) 132

^{1}H NMR spectrum of 3-(*tert*-butyl)benzo[d][1,2,3]triazin-4(3H)-one (**26**) 133

^{13}C NMR spectrum of 3-(*tert*-butyl)benzo[d][1,2,3]triazin-4(3H)-one (**26**) 134

^{1}H NMR spectrum of reduced Flutamide **S1** 135

^{19}F NMR spectrum of reduced Flutamide **S1** 136

^{13}C NMR spectrum of reduced Flutamide **S1** 137

^{1}H NMR of spectrum of [18-crown-6-K]$^{15}NO_3$ 138

^{13}C NMR of spectrum of [18-crown-6-K]$^{15}NO_3$ 139

^{15}N NMR of spectrum of [18-crown-6-K]$^{15}NO_3$ 140

^{39}K NMR of spectrum of [18-crown-6-K]$^{15}NO_3$ 141

REFERENCES 142

MATERIALS AND METHODS

All air- and moisture-insensitive reactions were carried out under ambient atmosphere and monitored by thin-layer chromatography (TLC). High-resolution mass spectra were obtained using *Q Exactive Plus* from *Thermo*. Concentration under reduced pressure was performed by rotary evaporation at 25–40°C at an appropriate pressure. Purified compounds were further dried under high vacuum (0.010–0.005 mbar). Yields refer to purified and spectroscopically pure compounds, unless otherwise stated.

Solvents

Acetonitrile was purchased from Fisher Scientific. Anhydrous solvents were obtained from Phoenix Solvent Drying Systems. All deuterated solvents were purchased from Euriso-Top.

Chromatography

Thin layer chromatography (TLC) was performed using EMD TLC plates pre-coated with 250 µm thickness silica gel 60 F254 plates and visualized by fluorescence quenching under 254 nm UV light or permanganate stain. Flash chromatography was performed using silica gel (40–63 µm particle size) purchased from Geduran®.

NMR Spectroscopy

NMR spectra were recorded on a Bruker AVANCE III HD 500 spectrometer operating at 500 MHz, 471 MHz, and 126 MHz, for ^{1}H, ^{19}F, and ^{13}C acquisitions, respectively; a Bruker AVANCE NEO 600 spectrometer equipped with a cryogenically cooled cryoBBO probe operating at 600 MHz and 61 MHz for ^{1}H and ^{15}N acquisitions, respectively; or on a Bruker AVANCE III HD 400 spectrometer operating at 400 MHz, 100 MHz, 29 MHz, and 54 MHz for ^{1}H, ^{13}C, ^{14}N, and ^{17}O acquisitions, respectively. ^{1}H and ^{13}C Chemical shifts are reported in ppm with the solvent residual peak as the internal standard. For ^{1}H NMR: $CDCl_3$, δ 7.26; DMSO-d_6, δ 2.50; CD_3CN, δ 1.94; For ^{13}C NMR: $CDCl_3$, δ 77.2; DMSO-d_6, δ 39.5; CD_3CN, δ 1.3. ^{13}C spectras are generally acquired with broadband proton decoupling. Data is reported as follows: s = singlet, d = doublet, t = triplet, q = quartet, p = pentet, h = heptet, m = multiplet, br = broad; coupling constants in Hz; integration.[1] NMR shifts of heteronuclei were referenced using their respective Ξ value using the *xiref* au program in Bruker Topspin following IUPAC recommendations.[2]

IR Spectroscopy

The gas phase was analyzed by IR spectroscopy using a Thermo Scientific Nicolet Avatar 370 FT-IR spectrometer, with 42 scans per spectrum and a spectral resolution of 2 cm^{-1}. Before each measurement, a background spectrum was recorded, which was subtracted from the actual measurement.

Mass spectrometry

ESI measurements were carried out on an Exactive (Orbitrap) spectrometer from Thermo Fisher

Scientific, Model: Q Exactive Plus. EI measurements were carried out on a GC Orbitrap spectrometer from Thermo Fisher Scientific, with a Thermo Scientific Trace 1310 gas chromatograph and a Q Exactive Orbitrap mass spectrometer. All measurements were done with an Orbitrap analyzer.

Computational details

All calculations were performed using the ORCA program package (version 6.0).[3,4] Geometry optimizations, transition-state location, and enthalpy calculations, including Gibbs free energy calculations at standard conditions (298.15 K and 1 atm) were carried out using the M06-2X-D3(0) density functional[5] and the ma-def2-TZVP basis set,[6, 7] i.e., at the level of M06-2X-D3(0)/ma-def2-TZVP for all reactions except those involving $Na_2S_2O_4$. In the latter case, we used def2-TZVP basis set due to linear dependencies in the ma-def2-TZVP basis set. Electronic energies were then refined using the larger ma-def2-QZVP basis set, i.e., at the level of the M06-2X-D3(0)/ma-def2-QZVP//M06-2X-D3(0)/(ma-)def2-TZVP. The solvent effect on geometries and energies was included in the computations treating the acetonitrile molecules implicitly within the Solvation Model based on Density (SMD) scheme.[8]

For the NO_2 release pathway from nitrate ion and SO_2 in the presence of catalytic water molecules, electronic energies were also refined using gold-standard CCSD(T) calculations[9] within SMD solvation scheme[8] for implicit acetonitrile. These computations employed the largest feasible basis sets from the aug-cc-pV*n*Z series[10,11] (abbreviated as a*n*Z, where *n* = D, T, Q, 5) to evaluate the reference UHF, CCSD correlation (C-CCSD), and perturbative triples (T) contributions. Complete Basis Set (CBS) limit extrapolations were then performed, as described in the Supporting Information of Ref. 12.

Reagents and starting materials

All reagents were used as received from commercial suppliers. $TBANO_3$, KNO_3, Na_2SO_3, $Na_2S_2O_4$, $NaHSO_3$ were obtained from *Sigma-Aldrich*; $Na_2S_2O_3$ $5H_2O$ was obtained from *Thermo Scientific* $Fe(NO_3)_3{\cdot}9H_2O$ was purchased from *Alfa Aesar*; MeCN was purchased from *Fisher Scientific*. All other reagents and substrates were used as received from commercial suppliers, such as *Sigma-Aldrich*, *TCI*, *Alfa Aesar, BLDPharm, or ChemImpex*.

Safety statement

The procedures reported in this work are intended for use only by individuals with proper training in experimental chemistry. All hazardous materials (solid, liquid, or gaseous) should be handled using the standard work procedures described in references such as Chapter 4 of "Prudent Practices in the Laboratory".[13] All chemical waste should be disposed of in accordance with local regulations. For general guidelines for the management of chemical waste, see Chapter 8 of "Prudent Practices in the Laboratory".[13] Reaction set-up, and chemical-specific hazards are highlighted in bold with "**Caution:**" notes in the procedures reported in these supplementary materials. It is important to note that the absence of a caution note does not imply that no significant hazards are associated with the chemicals involved in that procedure.

During the course of this study no explosions or violent decompositions occurred. A summary of the possible risks and hazards is described below:

Caution: When performing reactions in pressurized systems (such as closed vials, pressure tubes, and autoclaves), a blast shield must be used to minimize personal damage in case of an accident.

EXPERIMENTAL DATA

General procedures for deaminative cyanation

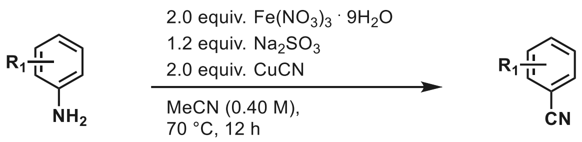

Caution: When performing reactions in pressurized systems (such as closed vials and pressure tubes), a blast shield must be used to minimize personal damage in case of an accident. See safety statement on page S6–S7.

0.5 mmol Scale: Under an ambient atmosphere, to a 4-mL borosilicate vial equipped with a Teflon-coated magnetic stir bar were added the (hetero) aromatic amine (if solid, 0.500 mmol, 1.00 equiv.), Na_2SO_3 (75.7 mg, 0.600 mmol, 1.20 equiv.), CuCN (89.6 mg, 1.00 mmol, 2.00 equiv.) and iron (III) nitrate nonahydrate (404 mg, 1.00 mmol, 2.00 equiv.) (Fig. S1A). Then, acetonitrile (MeCN) (1.25 mL, *c* = 0.40 M), (hetero) aromatic amine (if liquid, 0.500 mmol, 1.00 equiv.) were added. The vessel was sealed with a septum cap (Fig. S1B) and heated at 70°C for 12 h in an aluminum block on a heating plate (Fig. S1C). After cooling to 23 °C, the resulting mixture was passed through a pad of celite by eluting with ethyl acetate (50 mL). Then, the solvent was evaporated by rotary evaporation under reduced pressure. In order to obtain analytically pure samples of benzonitriles, the residue was purified by chromatography on silica gel.

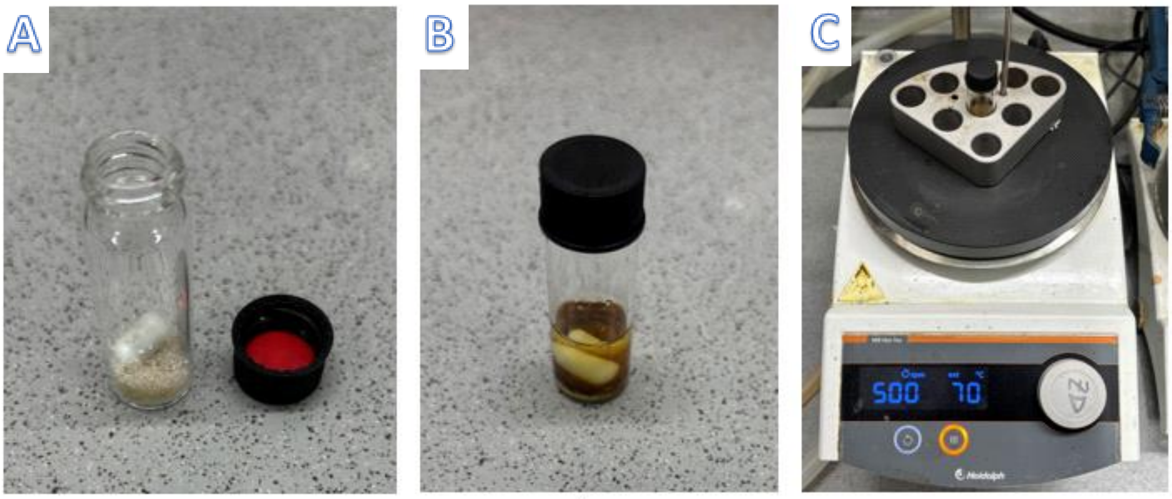

Fig S1. Reaction set-up for 0.500 mmol scale reactions. **A)** Weigh all the solids in a 4-mL borosilicate vial equipped with a magnetic stir bar. **B)** Addition of acetonitrile and sealed with a septum cap. **C)** The reaction is heated at 70°C for 12 h in an aluminum block on a heating plate.

Optimization tables

General optimization procedure : Under an ambient atmosphere, to a 4-mL borosilicate vial equipped with a magnetic stir bar were added benzocaine (16.5 mg, 0.100 mmol, 1.00 equiv.), the nitrate source (0.200 mmol, 2.00 equiv.), the reductant (0.120 mmol, 1.20 equiv.), the cyanide source (0.120 mmol, 1.20 equiv.), and the solvent (0.25 mL, c = 0.40 M). The vessel was sealed with a septum cap and heated at 70 ºC for 12 h in an aluminum block on a heating plate. After cooling to 23 °C, the resulting mixture was passed through a pad of celite by eluting with ethyl acetate (50 mL). The NMR yield was determined by ^{1}H-NMR spectroscopy by dissolving the resulting mixture in 0.50 mL of $CDCl_3$, and using CH_2Br_2 as internal standard (7.0 µL, 17 mg, 0.10 mmol, 1.0 equiv.). The integration of the CH_2Br_2 signal at 4.96 ppm (s, 2H) was compared to the signal of **16** at 7.27 ppm (d, J = 8.5 Hz, 2H).

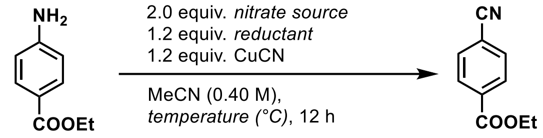

16

Table S1. Reaction outcome with different nitrate source, reductant and temperature.

Entry	Nitrate source	Reductant	Temperature (ºC)	Yield **16*** (%)
1	KNO_3	$Na_2S_2O_3·5H_2O$	85	< 5
2	$TBANO_3$	$Na_2S_2O_3·5H_2O$	85	< 5
3	KNO_3	-	85	< 5
4	$TBANO_3$	-	85	< 5
5	$Fe(NO_3)_3·9H_2O$	$Na_2S_2O_3·5H_2O$	25	< 5
6	$Fe(NO_3)_3·9H_2O$	$Na_2S_2O_3·5H_2O$	40	< 5
7	$Fe(NO_3)_3·9H_2O$	$Na_2S_2O_3·5H_2O$	85	40

*·The yield was determined by ^{1}H NMR spectroscopy at 500 MHz and 298 K by dissolving the residue of the reaction mixture in 0.5 mL of $CDCl_3$, and using CH_2Br_2 as internal standard (7.0 µL, 17 mg, 0.10 mmol, 1.0 equiv.). The integration of the CH_2Br_2 signal at 4.96 ppm (s, 2H) was compared to the signal of **16** at 7.73 ppm (d, J = 8.5 Hz, 2H).

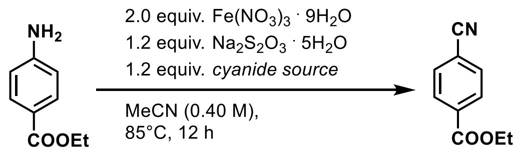

16

Table S2. Reaction outcome with different cyanide sources.

Entry	Cyanide source	Yield **16*** (%)
1	NaCN	< 5
2	CuCN	40
3	AgCN	< 5
4	$K_3[Fe(CN)_6]$	< 5
5	$K_2[Fe(CN)_6]$	< 5
6	TBACN	< 5

*.The yield was determined by ^{1}H NMR spectroscopy at 500 MHz and 298 K by dissolving the residue of the reaction mixture in 0.5 mL of $CDCl_3$, and using CH_2Br_2 as internal standard (7.0 µL, 17 mg, 0.10 mmol, 1.0 equiv.). The integration of the CH_2Br_2 signal at 4.96 ppm (s, 2H) was compared to the signal of **16** at 7.73 ppm (d, *J* = 8.5 Hz, 2H).

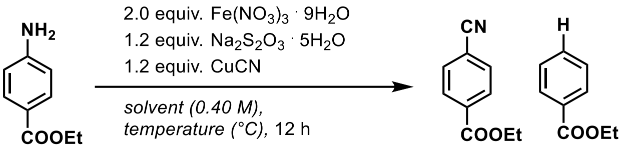

16 25

Table S3. Reaction outcome with different solvents and temperatures.

Entry	*Solvent*	Temperature (ºC)	Yield **16***(%)	Yield **25***(%)
1	*n*-PrCN	85	41	-
2	TFE	85	< 5	< 5
3	DMF	85	< 5	< 5
4	DMA	85	< 5	< 5
5	$CHCl_3$	60	-	54
6	HFIP	60	< 5	< 5
7	DCM	40	-	43
8	MeOH	60	12	16

*.The yield was determined by ^{1}H NMR spectroscopy at 500 MHz and 298 K by dissolving the residue of the reaction mixture in 0.5 mL of $CDCl_3$, and using CH_2Br_2 as internal standard (7.0 µL, 17 mg, 0.10 mmol, 1.0 equiv.). The integration of the CH_2Br_2 signal at 4.96 ppm (s, 2H) was compared to the signal of **16** at 7.73 ppm (d, *J* = 8.5 Hz, 2H) and signal of **25** at 7.51 (m, 1H).

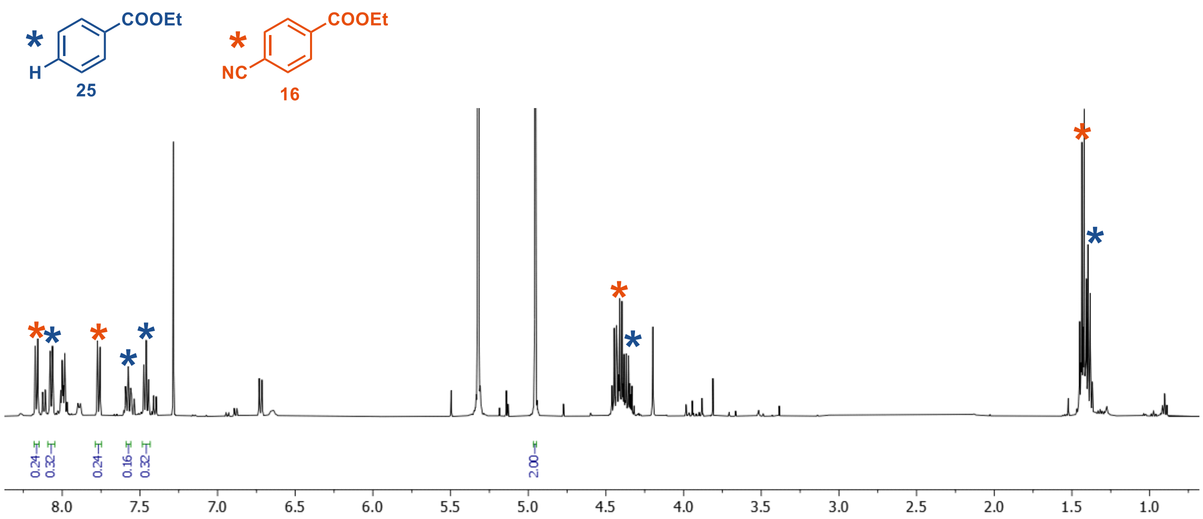

Fig S2. ^{1}H NMR spectra at 500 MHz NMR field and 298 K recorded in $CDCl_3$ solution after the reaction using CH_2Br_2 as internal standard (7.0 µL, 17 mg, 0.10 mmol, 1.0 equiv.). The integration of the CH_2Br_2 signal at 4.96 ppm (s, 2H) was compared to the signal of **16** at 7.73 ppm (d, J = 8.5 Hz, 2H) and signal of **25** at 7.51 (m, 1H). Blue stars: proto-deamination product **25**. Orange stars: aryl nitrile **16**.

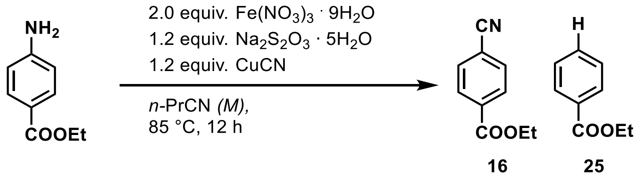

Fig S3. Reaction outcome with different conc. in *n*-PrCN.

Entry	Conc. of *n*-PrCN	Yield **16***(%)	Yield **25***(%)
1	1 M	40	13
2	0.4 M	40	11
3	0.2 M	38	14
4	0.13 M	26	19
5	0.1 M	17	34

*.The yield was determined by ^{1}H NMR spectroscopy at 500 MHz and 298 K by dissolving the residue of the reaction mixture in 0.5 mL of $CDCl_3$, and using CH_2Br_2 as internal standard (7.0 µL, 17 mg, 0.10 mmol, 1.0 equiv.). The integration of the CH_2Br_2 signal at 4.96 ppm (s, 2H) was compared to the signal of **16** at 7.73 ppm (d, J = 8.5 Hz, 2H) and signal of **25** at 7.51 (m, 1H).

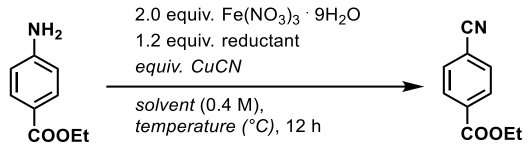

16

Table S4. Reaction outcome with different reductants and different equivalents of CuCN.

Entry	Reductant	CuCN equivalent	Solvent	Temperature (ºC)	Yield **16***(%)
1	$Na_2S_2O_3{\cdot}5H_2O$	1.2	*n*-PrCN	85	40
2	$Na_2S_2O_3{\cdot}5H_2O$	1.5	*n*-PrCN	85	42
3	$Na_2S_2O_3{\cdot}5H_2O$	2	*n*-PrCN	85	46
4	$K_2S_4O_6$	1.2	*n*-PrCN	85	36
5	$K_2S_4O_6$	1.5	*n*-PrCN	85	53
6	$K_2S_4O_6$	2	*n*-PrCN	85	54
7	$Na_2S_2O_4$	1.2	*n*-PrCN	85	47
8	$Na_2S_2O_4$	1.5	*n*-PrCN	85	57
9	$Na_2S_2O_4$	2	*n*-PrCN	85	60
10	$Na_2S_2O_4$	2	*n*-PrCN	70	60
11	$NaHSO_3$	2	MeCN	70	68
12	Na_2SO_3	2	MeCN	70	69

* The yield was determined by ^{1}H NMR spectroscopy at 500 MHz and 298 K by dissolving the residue of the reaction mixture in 0.5 mL of $CDCl_3$, and using CH_2Br_2 as internal standard (7.0 µL, 17 mg, 0.10 mmol, 1.0 equiv.). The integration of the CH_2Br_2 signal at 4.96 ppm (s, 2H) was compared to the signal of 16 at 7.73 ppm (d, *J* = 8.5 Hz, 2H).

Aniline Scope for Deaminative Cyanation

Darunavir-derived benzonitrile 1

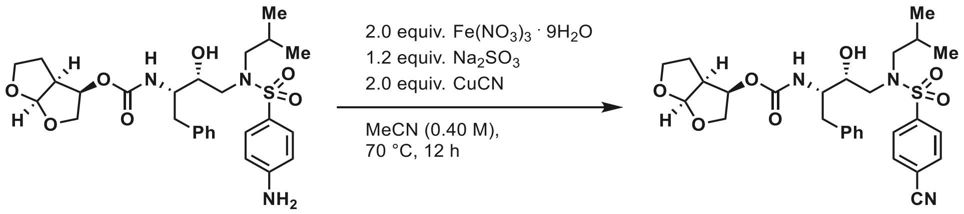

50% yield **1**

Caution: When performing reactions in pressurized systems (such as closed vials and pressure tubes), a blast shield must be used to minimize personal damage in case of an accident. See safety statement on page S6–S7.

Under an ambient atmosphere, to a 4-mL borosilicate vial equipped with a Teflon-coated magnetic stir bar were added Darunavir (274 mg, 0.500 mmol, 1.00 equiv.), Na_2SO_3 (75.7 mg, 0.600 mmol, 2.00 equiv.), CuCN (89.6 mg, 1.00 mmol, 2.00 equiv.) and iron (III) nitrate nonahydrate (404 mg, 1.00 mmol, 2.00 equiv.). Then, acetonitrile (MeCN) (1.25 mL, *c* = 0.40 M) was added. The vessel was sealed with a septum cap and heated at 70°C for 12 h in an aluminum block on a heating plate. After cooling to 23 °C, the resulting mixture was passed through a pad of celite by eluting with ethyl acetate (50 mL). Then, the solvent was evaporated by rotary evaporation under reduced pressure. The residue was purified by silica gel chromatography by eluting with EtOAc/pentane (40:60 (v/v)) to afford 139 mg (50%) of the title compound **1** as light yellow solid.

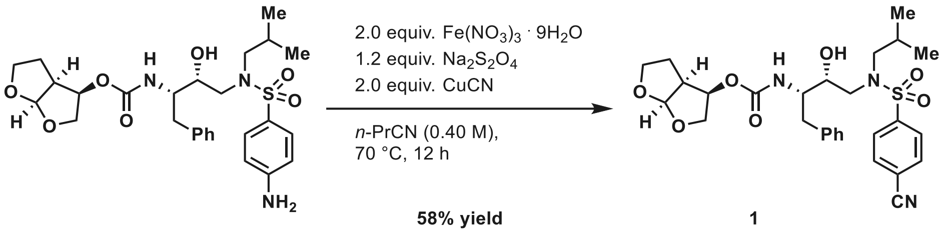

58% yield **1**

Caution: When performing reactions in pressurized systems (such as closed vials and pressure tubes), a blast shield must be used to minimize personal damage in case of an accident. See safety statement on page S6–S7.

Under an ambient atmosphere, to a 4-mL borosilicate vial equipped with a Teflon-coated magnetic stir bar were added Darunavir (274 mg, 0.500 mmol, 1.00 equiv.), $Na_2S_2O_4$ (104 mg, 0.600 mmol, 2.00 equiv.), CuCN (89.6 mg, 1.00 mmol, 2.00 equiv.) and iron (III) nitrate nonahydrate (404 mg, 1.00 mmol, 2.00 equiv.). Then butyronitrile (*n*-PrCN) (1.25 mL, *c* = 0.4 M) was added. The vessel was sealed with a septum cap and heated at 70°C for 12 h in an aluminum block on a heating plate. After cooling to 23 °C, the resulting mixture was passed through a pad of celite by eluting with ethyl acetate (50 mL). Then, the solvent was evaporated by rotary evaporation under reduced pressure. The residue was purified by silica gel chromatography by eluting with EtOAc/pentane (40:60 (v/v)) to afford 161 mg (58%) of the title compound **1** as light yellow solid.

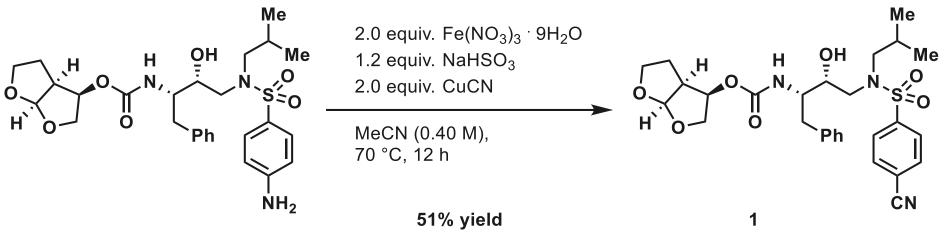

51% yield **1**

Caution: When performing reactions in pressurized systems (such as closed vials and pressure tubes), a blast shield must be used to minimize personal damage in case of an accident. See safety statement on page S6–S7.

Under an ambient atmosphere, to a 4-mL borosilicate vial equipped with a Teflon-coated magnetic stir bar were added Darunavir (274 mg, 0.500 mmol, 1.00 equiv.), $NaHSO_3$ (62.5 mg, 0.600 mmol, 2.00 equiv.), CuCN (89.6 mg, 1.00 mmol, 2.00 equiv.) and iron (III) nitrate nonahydrate (404 mg, 1.00 mmol, 2.00 equiv.). Then, acetonitrile (MeCN) (1.25 mL, *c* = 0.4 M) was added. The vessel was sealed with a septum cap and heated at 70°C for 12 h in an aluminum block on a heating plate. After cooling to 23 °C, the resulting mixture was passed through a pad of celite by eluting with ethyl acetate (50 mL). Then, the solvent was evaporated by rotary evaporation under reduced pressure. The residue was purified by silica gel chromatography by eluting with EtOAc/pentane (40:60 (v/v)) to afford 142 mg (51%) of the title compound **1** as light yellow solid.

R_f = 0.72 (EtOAc in pentane = 70%, v/v (UV)).

NMR Spectroscopy:

^{1}H NMR (500 MHz, DMSO-d_6, 25°C, δ): 8.07 (d, *J* = 8.5 Hz, 2H), 7.96 (d, *J* = 8.6 Hz, 2H), 7.28 – 7.17 (m, 5H), 7.16 – 7.11 (m, 1H), 5.51 (d, *J* = 5.2 Hz, 1H), 5.05 (d, *J* = 6.3 Hz, 1H), 4.86 (dt, *J* = 8.0, 5.6 Hz, 1H), 3.85 (dd, *J* = 9.6, 5.9 Hz, 1H), 3.73 (td, *J* = 8.2, 1.9 Hz, 1H), 3.58 (dd, *J* = 9.6, 5.4 Hz, 4H), 3.37 (dd, *J* = 14.9, 2.3 Hz, 1H), 3.13 (dd, *J* = 13.6, 8.6 Hz, 1H), 3.04 – 2.95 (m, 2H), 2.89 (dd, *J* = 13.7, 6.5 Hz, 1H), 2.82 – 2.74 (m, 1H), 2.45 (dd, *J* = 13.7, 10.5 Hz, 1H), 1.97 (ddq, *J* = 13.1, 8.3, 6.5 Hz, 1H), 1.40 (dddd, *J* = 13.1, 10.8, 9.4, 8.1 Hz, 1H), 1.30 – 1.20 (m, 1H), 0.83 (dd, *J* = 22.9, 6.6 Hz, 6H).

^{13}C NMR (125 MHz, DMSO-d_6, 25°C, δ): 155.2, 143.7, 139.2, 133.3, 129.2, 127.9, 127.8, 125.8, 117.7, 114.9, 108.8, 72.5, 71.7, 70.4, 68.8, 55.9, 55.6, 51.4, 45.0, 35.4, 25.9, 25.5, 19.8, 19.7.

HRMS-ESI(m/z) calc'd for $C_{28}H_{35}O_7N_3Na_1S_1^+$ $[M+Na]^+$, 580.2088; found, 580.2087; deviation: −0.0 ppm.

Melting point: 155 – 157 °C.

2-Methyl-3-nitrobenzonitrile (2)

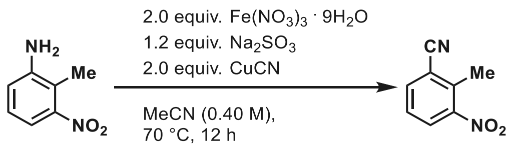

80% yield **2**

Caution: When performing reactions in pressurized systems (such as closed vials and pressure tubes), a blast shield must be used to minimize personal damage in case of an accident. See safety statement on page S6–S7.

Under an ambient atmosphere, to a 4-mL borosilicate vial equipped with a Teflon-coated magnetic stir bar were added 2-methyl-3-nitroaniline (76.1 mg, 0.500 mmol, 1.00 equiv.), Na_2SO_3 (75.7 mg, 0.600 mmol, 2.00 equiv.), CuCN (89.6 mg, 1.00 mmol, 2.00 equiv.) and iron (III) nitrate nonahydrate (404 mg, 1.00 mmol, 2.00 equiv.). Then, acetonitrile (MeCN) (1.25 mL, c = 0.4 M) was added. The vessel was sealed with a septum cap and heated at 70°C for 12 h in an aluminum block on a heating plate. After cooling to 23 °C, the resulting mixture was passed through a pad of celite by eluting with ethyl acetate (50 mL). Then, the solvent was evaporated by rotary evaporation under reduced pressure. The residue was purified by silica gel chromatography by eluting with EtOAc/pentane (5:95 (v/v)) to afford 65 mg (80%) of the title compound **2** as off white solid.

$\mathbf{R_f}$ = 0.31 (EtOAc in pentane = 10%, v/v (UV)).

NMR Spectroscopy:

^{1}**H NMR** (500 MHz, $CDCl_3$, 25°C, δ): 8.10 (dd, J = 8.2, 1.5 Hz, 1H), 7.87 (dd, J = 7.7, 1.5 Hz, 1H), 7.50 (t, J = 8.0 Hz, 1H), 2.77 (s, 3H).

^{13}**C NMR** (125 MHz, $CDCl_3$, 25°C, δ):150.3, 137.0, 136.9, 128.6, 127.7, 116.5, 116.4, 18.1.

HRMS-EI(m/z) calc'd for $C_8H_6N_2O_2^+$ $[M]^+$, 162.0424; found, 162.0425; deviation: +1.1 ppm.

Melting point: 69 – 70 °C.

2-Chloroterephthalonitrile (3)

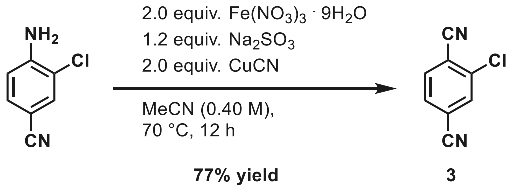

Caution: When performing reactions in pressurized systems (such as closed vials and pressure tubes), a blast shield must be used to minimize personal damage in case of an accident. See safety statement on page S6–S7.

Under an ambient atmosphere, to a 4-mL borosilicate vial equipped with a Teflon-coated magnetic stir bar were added 4-amino-2-chlorbenzonitrile (76.3 mg, 0.500 mmol, 1.00 equiv.), Na_2SO_3 (75.7 mg, 0.600 mmol, 2.00 equiv.), CuCN (89.6 mg, 1.00 mmol, 2.00 equiv.) and iron (III) nitrate nonahydrate (404 mg, 1.00 mmol, 2.00 equiv.). Then, acetonitrile (MeCN) (1.25 mL, c = 0.4 M) was added. The vessel was sealed with a septum cap and heated at 70°C for 12 h in an aluminum block on a heating plate. After cooling to 23 °C, the resulting mixture was passed through a pad of celite by eluting with ethyl acetate (50 mL). Then, the solvent was evaporated by rotary evaporation under reduced pressure. The residue was purified by silica gel chromatography by eluting with EtOAc/pentane (5:95 (v/v)) to afford 63 mg (77%) of the title compound **3** as off white solid.

R*f* = 0.28 (EtOAc in pentane = 10%, v/v (UV)).

NMR Spectroscopy:

^{1}H NMR (500 MHz, $CDCl_3$, 25°C, δ): 7.85 – 7.79 (m, 2H), 7.69 (dd, *J* = 8.1, 1.5 Hz, 1H).

^{13}C NMR (125 MHz, $CDCl_3$, 25°C, δ): 138.1, 134.7, 133.3, 130.7, 117.8, 116.0, 114.5

HRMS-EI(m/z) calc'd for $C_8H_3N_2Cl_1^+$ $[M]^+$, 161.9980; found, 161.9979; deviation: +0.3 ppm.

Melting point: 155 – 157 °C.

2-(Trifluoromethylthio)benzonitrile (4)

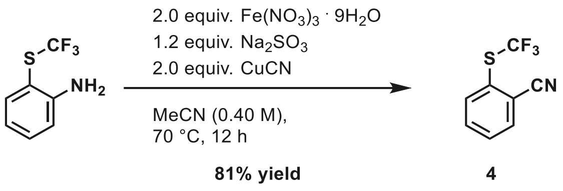

Caution: When performing reactions in pressurized systems (such as closed vials and pressure tubes), a blast shield must be used to minimize personal damage in case of an accident. See safety statement on page S6–S7.

Under an ambient atmosphere, to a 4-mL borosilicate vial equipped with a Teflon-coated magnetic stir bar were added 2-(trifluormethylthio)aniline (96.6 mg, 0.500 mmol, 1.00 equiv.), Na_2SO_3 (75.7 mg, 0.600 mmol, 2.00 equiv.), CuCN (89.6 mg, 1.00 mmol, 2.00 equiv.) and iron (III) nitrate nonahydrate (404 mg, 1.00 mmol, 2.00 equiv.). Then, acetonitrile (MeCN) (1.25 mL, *c* = 0.4 M) was added. The vessel was sealed with a septum cap and heated at 70°C for 12 h in an aluminum block on a heating plate. After cooling to 23 °C, the resulting mixture was passed through a pad of celite by eluting with ethyl acetate (50 mL). Then, the solvent was evaporated by rotary evaporation under reduced pressure. The residue was purified by silica gel chromatography by eluting with EtOAc/pentane (4:96 (v/v)) to afford 82 mg (81%) of the title compound **4** as orange colored oil.

R*f* = 0.30 (EtOAc in pentane = 10%, v/v (UV)).

NMR Spectroscopy:

^{1}H NMR (500 MHz, $CDCl_3$, 25°C, δ): 7.87 (d, *J* = 7.7 Hz, 1H), 7.82 (dd, *J* = 7.5, 1.7 Hz, 1H), 7.67 (dtd, *J* = 22.5, 7.6, 1.5 Hz, 2H).

^{13}C NMR (125 MHz, $CDCl_3$, 25°C, δ): 138.4, 134.5, 133.6, 131.8, 128.9 (q, *J* = 308.8 Hz), 127.5 (q, *J* = 2.5 Hz), 120.7, 116.4.

^{19}F NMR (470 MHz, $CDCl_3$, 25°C, δ): −41.8.

HRMS-EI(m/z) calc'd for $C_8H_4N_1S_1F_3^+$ $[M]^+$,203.0012; found, 203.0011; deviation: +0.1 ppm.

6-Bromo-5-methylnicotinonitrile (5)

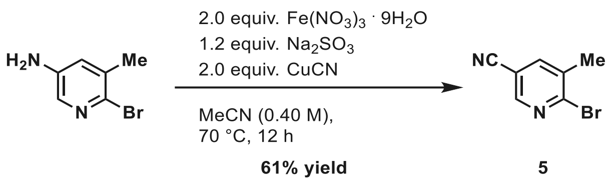

Caution: When performing reactions in pressurized systems (such as closed vials and pressure tubes), a blast shield must be used to minimize personal damage in case of an accident. See safety statement on page S6–S7.

Under an ambient atmosphere, to a 4-mL borosilicate vial equipped with a Teflon-coated magnetic stir bar were added 5-amino-2-brom-3-methylpyridine (93.6 mg, 0.500 mmol, 1.00 equiv.), Na_2SO_3 (75.7 mg, 0.600 mmol, 2.00 equiv.), CuCN (89.6 mg, 1.00 mmol, 2.00 equiv.) and iron (III) nitrate nonahydrate (404 mg, 1.00 mmol, 2.00 equiv.). Then, acetonitrile (MeCN) (1.25 mL, *c* = 0.4 M) was added. The vessel was sealed with a septum cap and heated at 70°C for 12 h in an aluminum block on a heating plate. After cooling to 23 °C, the resulting mixture was passed through a pad of celite by eluting with ethyl acetate (50 mL). Then, the solvent was evaporated by rotary evaporation under reduced pressure. The residue was purified by silica gel chromatography by eluting with EtOAc/pentane (3:97 (v/v)) to afford 60 mg (61%) of the title compound **5** as off white solid.

R_f = 0.37 (EtOAc in pentane = 21%, v/v (UV)).

NMR Spectroscopy:

> **^{1}H NMR** (500 MHz, $CDCl_3$, 25°C, δ): 8.46 (d, *J* = 2.3 Hz, 1H), 7.75 (d, *J* = 2.4 Hz, 1H), 2.45 – 2.42 (m, 3H).
>
> **^{13}C NMR** (125 MHz, $CDCl_3$, 25°C, δ): 149.8, 149.3, 140.7, 136.5, 115.9, 109.1, 22.1.

HRMS-EI(m/z) calc'd for $C_7H_5N_2Br_1^+$ $[M]^+$, 195.9630; found, 195.9631; deviation: +0.5 ppm.

Melting point: 115 – 117 °C.

5-Fluoro-2-iodobenzonitrile (6)

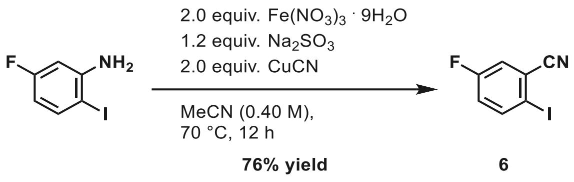

Caution: When performing reactions in pressurized systems (such as closed vials and pressure tubes), a blast shield must be used to minimize personal damage in case of an accident. See safety statement on page S6–S7.

Under an ambient atmosphere, to a 4-mL borosilicate vial equipped with a Teflon-coated magnetic stir bar were added 5-fluor-2-iodaniline (118 mg, 0.500 mmol, 1.00 equiv.), Na_2SO_3 (75.7 mg, 0.600 mmol, 2.00 equiv.), CuCN (89.6 mg, 1.00 mmol, 2.00 equiv.) and iron (III) nitrate nonahydrate

(404 mg, 1.00 mmol, 2.00 equiv.). Then, acetonitrile (MeCN) (1.25 mL, c = 0.4 M) was added. The vessel was sealed with a septum cap and heated at 70°C for 12 h in an aluminum block on a heating plate. After cooling to 23 °C, the resulting mixture was passed through a pad of celite by eluting with ethyl acetate (50 mL). Then, the solvent was evaporated by rotary evaporation under reduced pressure. The residue was purified by silica gel chromatography by eluting with EtOAc/pentane (2:98 (v/v)) to afford 94 mg (76%) of the title compound **6** as off white solid.

R_f = 0.53 (EtOAc in pentane = 10%, v/v (UV)).

NMR Spectroscopy:

^{1}H NMR (500 MHz, $CDCl_3$, 25°C, δ): 7.88 (dd, J = 8.8, 5.1 Hz, 1H), 7.35 (dd, J = 7.9, 2.9 Hz, 1H), 7.06 (ddd, J = 8.8, 7.9, 2.9 Hz, 1H).

^{13}C NMR (125 MHz, $CDCl_3$, 25°C, δ): 162.1 (q, J = 250 Hz), 141.3 (d, J = 8.7 Hz), 122.1, 122.0 (d, J = 21.2 Hz), 121.7 (d, J = 25 Hz), 118.3 (d, J = 3.7 Hz), 92.0 (d, J = 5 Hz).

19F NMR (470 MHz, $CDCl_3$, 25°C, δ): −111.1.

HRMS-EI(m/z) calc'd for $C_7H_3N_1F_1I_1^+$ [M]$^+$, 246.9289; found, 246.9289; deviation: −0.0 ppm.

Melting point: 61 – 63 °C.

4-Benzoylbenzonitrile (7)

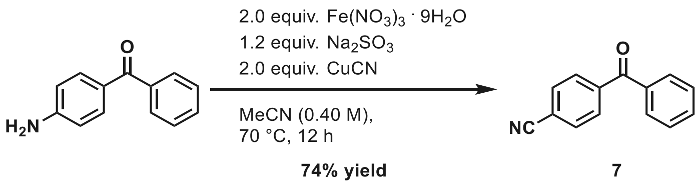

7

Caution: When performing reactions in pressurized systems (such as closed vials and pressure tubes), a blast shield must be used to minimize personal damage in case of an accident. See safety statement on page S6–S7.

Under an ambient atmosphere, to a 4-mL borosilicate vial equipped with a Teflon-coated magnetic stir bar were added 4-aminobenzophenone (98.6 mg, 0.500 mmol, 1.00 equiv.), Na_2SO_3 (75.7 mg, 0.600 mmol, 2.00 equiv.), CuCN (89.6 mg, 1.00 mmol, 2.00 equiv.) and iron (III) nitrate nonahydrate (404 mg, 1.00 mmol, 2.00 equiv.). Then, acetonitrile (MeCN) (1.25 mL, c = 0.4 M) was added. The vessel was sealed with a septum cap and heated at 70°C for 12 h in an aluminum block on a heating plate. After cooling to 23 °C, the resulting mixture was passed through a pad of celite by eluting with ethyl acetate (50 mL). Then, the solvent was evaporated by rotary evaporation under reduced pressure. The residue was purified by silica gel chromatography by eluting with EtOAc/pentane (3:97 (v/v)) to afford 77 mg (74%) of the title compound **7** as light orange solid.

R_f = 0.34 (EtOAc in pentane = 10%, v/v (UV)).

NMR Spectroscopy:

^{1}H NMR (500 MHz, $CDCl_3$, 25°C, δ): 7.86 (d, J = 8.5 Hz, 2H), 7.82 – 7.69 (m, 4H), 7.65 – 7.59

(m, 1H), 7.50 (t, J = 7.8 Hz, 2H).

^{13}C NMR (125 MHz, $CDCl_3$, 25°C, δ): 195.1, 141.3, 136.4, 133.4, 132.2, 130.3, 130.1, 128.7, 118.1, 115.7.

HRMS-EI(m/z) calc'd for $C_{14}H_9N_1O_1^+$ [M]$^+$, 207.0678; found, 207.0676; deviation: −1.0 ppm.

Melting point: 112 – 114 °C.

2,2-Difluorobenzo[d][1,3]dioxole-5-carbonitrile (8)

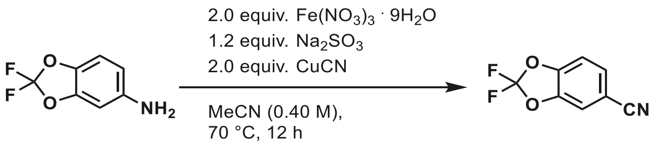

Caution: When performing reactions in pressurized systems (such as closed vials and pressure tubes), a blast shield must be used to minimize personal damage in case of an accident. See safety statement on page S6–S7.

Under an ambient atmosphere, to a 4-mL borosilicate vial equipped with a Teflon-coated magnetic stir bar were added 2,2-Difluorbenzo[d][1,3]dioxol-5-amine (86.6 mg, 0.500 mmol, 1.00 equiv.), Na_2SO_3 (75.7 mg, 0.600 mmol, 2.00 equiv.), CuCN (89.6 mg, 1.00 mmol, 2.00 equiv.) iron (III) nitrate nonahydrate (404 mg, 1.00 mmol, 2.00 equiv.). Then, acetonitrile (MeCN) (1.25 mL, c = 0.4 M) was added. The vessel was sealed with a septum cap and heated at 70°C for 12 h in an aluminum block on a heating plate. After cooling to 23 °C, the resulting mixture was passed through a pad of celite by eluting with ethyl acetate (50 mL). Then, the solvent was evaporated by rotary evaporation under reduced pressure. The residue was purified by silica gel chromatography by eluting with EtOAc/pentane (1:99 (v/v)) to afford 47 mg (52%) of the title compound **8** as a light yellow solid.

R$_f$ = 0.53 (EtOAc in pentane = 10%, v/v (UV)).

NMR Spectroscopy:

^{1}H NMR (500 MHz, $CDCl_3$, 25°C, δ): 7.47 (dd, J = 8.3, 1.6 Hz, 1H), 7.36 (d, J = 1.6 Hz, 1H), 7.18 (d, J = 8.3 Hz, 1H).

^{13}C NMR (125 MHz, $CDCl_3$, 25°C, δ): 147.0, 144.0, 131.7 (t, J = 257.5 Hz), 129.7, 117.7, 113.1, 110.6, 107.9.

19F NMR (470 MHz, $CDCl_3$, 25°C, δ): −49.6.

HRMS-EI(m/z) calc'd for $C_8H_3N_1O_2F_2^+$ [M]$^+$,183.0126; found, 183.0125; deviation: −0.5 ppm.

Melting point: 55 – 57 °C.

4'-Chloro-[1,1'-biphenyl]-2-carbonitrile (9)

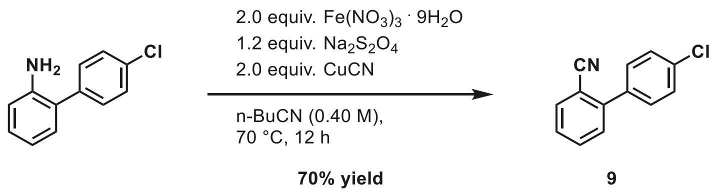

Caution: When performing reactions in pressurized systems (such as closed vials and pressure tubes), a blast shield must be used to minimize personal damage in case of an accident. See safety statement on page S6–S7.

Under an ambient atmosphere, to a 4-mL borosilicate vial equipped with a Teflon-coated magnetic stir bar were added 4'-Chlor-(1,1'-biphenyl)-2-amine (102 mg, 0.500 mmol, 1.00 equiv.), $Na_2S_2O_4$ (104 mg, 0.600 mmol, 2.00 equiv.), CuCN (89.6 mg, 1.00 mmol, 2.00 equiv.) and iron (III) nitrate nonahydrate (404 mg, 1.00 mmol, 2.00 equiv.). Then, butyronitrile (*n*-PrCN) (1.25 mL, *c* = 0.4 M) was added, the vial was quickly sealed with a septum cap and stirred at 70 °C for 12 h. After cooling to 23 °C, the resulting mixture was passed through a pad of celite by eluting with ethyl acetate (50 mL). Then, the solvent was evaporated by rotary evaporation under reduced pressure. The residue was purified by silica gel chromatography by eluting with EtOAc/pentane (3:97 (v/v)) to afford 75 mg (70%) of the title compound **9** as a light yellow solid.

$\mathbf{R}_f$ = 0.47 (EtOAc in pentane = 10%, v/v (UV)).

NMR Spectroscopy:

> **^{1}H NMR** (500 MHz, $CDCl_3$, 25°C, δ): 7.77 (dd, *J* = 7.7, 1.4 Hz, 1H), 7.65 (td, *J* = 7.7, 1.4 Hz, 1H), 7.51 – 7.44 (m, 6H).
>
> **^{13}C NMR** (125 MHz, $CDCl_3$, 25°C, δ): 144.3, 136.7, 135.2, 133.9, 133.1, 130.2, 130.0, 129.1, 128.0, 118.6, 111.4.

HRMS-EI(m/z) calc'd for $C_{13}H_8N_1Cl_1^+$ [M]$^+$, 213.0339; found, 213.0340; deviation: −0.1 ppm.

Melting point: 120 – 122 °C.

2-Chloronicotinonitrile (10)

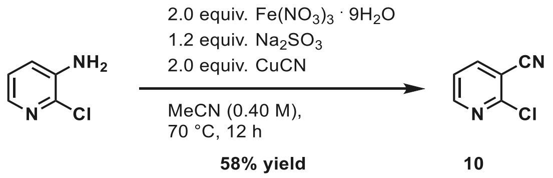

Caution: When performing reactions in pressurized systems (such as closed vials and pressure tubes), a blast shield must be used to minimize personal damage in case of an accident. See safety statement on page S6–S7.

Under an ambient atmosphere, to a 4-mL borosilicate vial equipped with a Teflon-coated magnetic stir bar were added 3-amino-2-chlorpyridine (64.3 mg, 0.500 mmol, 1.00 equiv.), Na_2SO_3 (75.7 mg,

0.600 mmol, 2.00 equiv.), CuCN (89.6 mg, 1.00 mmol, 2.00 equiv.) and iron (III) nitrate nonahydrate (404 mg, 1.00 mmol, 2.00 equiv.). Then, acetonitrile (MeCN) (1.25 mL, *c* = 0.4 M) was added. The vessel was sealed with a septum cap and heated at 70°C for 12 h in an aluminum block on a heating plate. After cooling to 23 °C, the resulting mixture was passed through a pad of celite by eluting with ethyl acetate (50 mL). Then, the solvent was evaporated by rotary evaporation under reduced pressure. The residue was purified by silica gel chromatography by eluting with EtOAc/pentane (10:90 (v/v)) to afford 40 mg (58%) of the title compound **10** as off white solid.

R*f* = 0.44 (EtOAc in pentane = 40%, v/v (UV)).

NMR Spectroscopy:

^{1}**H NMR** (500 MHz, $CDCl_3$, 25°C, δ): 8.61 (dd, *J* = 4.9, 2.0 Hz, 1H), 8.02 (dd, *J* = 7.7, 2.0 Hz, 1H), 7.40 (dd, *J* = 7.7, 4.9 Hz, 1H).

^{13}**C NMR** (125 MHz, $CDCl_3$, 25°C, δ): 153.0, 142.7, 122.3, 114.7, 111.1.

HRMS-EI(m/z) calc'd for $C_6H_3N_2Cl_1^+$ $[M]^+$, 137.9979; found, 137.9981; deviation: +1.8 ppm.

Melting point: 106 – 108 °C.

2,4,6-Tribromobenzonitrile (11)

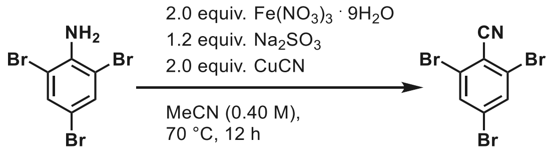

Caution: When performing reactions in pressurized systems (such as closed vials and pressure tubes), a blast shield must be used to minimize personal damage in case of an accident. See safety statement on page S6–S7.

Under an ambient atmosphere, to a 4-mL borosilicate vial equipped with a Teflon-coated magnetic stir bar were added 2,4,6-tribromaniline (165 mg, 0.500 mmol, 1.00 equiv.), Na_2SO_3 (75.7 mg, 0.600 mmol, 2.00 equiv.), CuCN (89.6 mg, 1.00 mmol, 2.00 equiv.) iron (III) nitrate nonahydrate (404 mg, 1.00 mmol, 2.00 equiv.). Then, acetonitrile (MeCN) (1.25 mL, *c* = 0.4 M) was added. The vessel was sealed with a septum cap and heated at 70°C for 12 h in an aluminum block on a heating plate. After cooling to 23 °C, the resulting mixture was passed through a pad of celite by eluting with ethyl acetate (50 mL). Then, the solvent was evaporated by rotary evaporation under reduced pressure. The residue was purified by silica gel chromatography by eluting with EtOAc/pentane (1:99 (v/v)) to afford 123 mg (72%) of the title compound **11** as off white solid.

R*f* = 0.62 (EtOAc in pentane = 10%, v/v (UV)).

NMR Spectroscopy:

^{1}**H NMR** (500 MHz, $CDCl_3$, 25°C, δ): 7.81 (s, 2H).

^{13}C NMR (125 MHz, $CDCl_3$, 25°C, δ): 134.8, 128.3, 127.2, 117.9, 115.5.

HRMS-EI(m/z) calc'd for $C_7H_2N_1Br_3^+$ $[M]^+$, 336.7732; found, 336.7736; deviation: +1.4 ppm.

Melting point: 126 – 128 °C.

2,6-Dimethylterephthalonitrile (12)

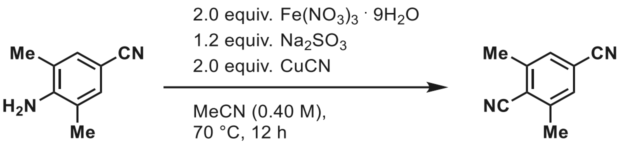

54% yield 12

Caution: When performing reactions in pressurized systems (such as closed vials and pressure tubes), a blast shield must be used to minimize personal damage in case of an accident. See safety statement on page S6–S7.

Under an ambient atmosphere, to a 4-mL borosilicate vial equipped with a Teflon-coated magnetic stir bar were added 4-amino-3,5-dimethylbenzonitrile (73.1 mg, 0.500 mmol, 1.00 equiv.), Na_2SO_3 (75.7 mg, 0.600 mmol, 2.00 equiv.), CuCN (89.6 mg, 1.00 mmol, 2.00 equiv.) and iron (III) nitrate nonahydrate (404 mg, 1.00 mmol, 2.00 equiv.). Then, acetonitrile (MeCN) (1.25 mL, c = 0.4 M) was added. The vessel was sealed with a septum cap and heated at 70°C for 12 h in an aluminum block on a heating plate. After cooling to 23 °C, the resulting mixture was passed through a pad of celite by eluting with ethyl acetate (50 mL). Then, the solvent was evaporated by rotary evaporation under reduced pressure. The residue was purified by silica gel chromatography by eluting with EtOAc/pentane (2:98 (v/v)) to afford 42 mg (54%) of the title compound **12** as off white solid.

R_f = 0.37 (EtOAc in pentane = 10%, v/v (UV)).

NMR Spectroscopy:

^{1}H NMR (500 MHz, $CDCl_3$, 25°C, δ): 7.43 (s, 2H), 2.58 (s, 6H).

^{13}C NMR (125 MHz, $CDCl_3$, 25°C, δ): 143.5, 130.7, 118.0, 117.5, 115.9, 115.7, 20.8.

HRMS-EI(m/z) calc'd for $C_{10}H_8N_2^+$ $[M]^+$, 156.0682; found, 156.0680; deviation: −0.8 ppm.

Melting point: 171 – 172 °C.

Terephthalonitrile (13)

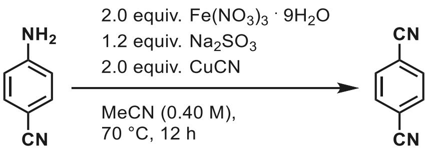

78% yield 13

Caution: When performing reactions in pressurized systems (such as closed vials and pressure tubes), a blast shield must be used to minimize personal damage in case of an accident. See safety

statement on page S6–S7.

Under an ambient atmosphere, to a 4-mL borosilicate vial equipped with a Teflon-coated magnetic stir bar were added 4-aminobenzonitrile (59.1 mg, 0.500 mmol, 1.00 equiv.), Na_2SO_3 (75.7 mg, 0.600 mmol, 2.00 equiv.), CuCN (89.6 mg, 1.00 mmol, 2.00 equiv.) and iron (III) nitrate nonahydrate (404 mg, 1.00 mmol, 2.00 equiv.). Then, acetonitrile (MeCN) (1.25 mL, *c* = 0.4 M) was added. The vessel was sealed with a septum cap and heated at 70°C for 12 h in an aluminum block on a heating plate. After cooling to 23 °C, the resulting mixture was passed through a pad of celite by eluting with ethyl acetate (50 mL). Then, the solvent was evaporated by rotary evaporation under reduced pressure. The residue was purified by silica gel chromatography by eluting with EtOAc/pentane (3:97 (v/v)) to afford 50 mg (78%) of the title compound **13** as off white solid.

$\mathbf{R_f}$ = 0.25 (EtOAc in pentane = 10%, v/v (UV)).

NMR Spectroscopy:

^{1}H NMR (500 MHz, $CDCl_3$, 25°C, δ): 7.79 (s, 4H).

^{13}C NMR (125 MHz, $CDCl_3$, 25°C, δ): 132.9, 117.1, 116.8.

HRMS-EI(m/z) calc'd for $C_8H_4N_2^+$ $[M]^+$, 128.0369; found, 128.0369; deviation: −0.1 ppm.

Melting point: 226 – 228 °C.

Reduced flutamide S1

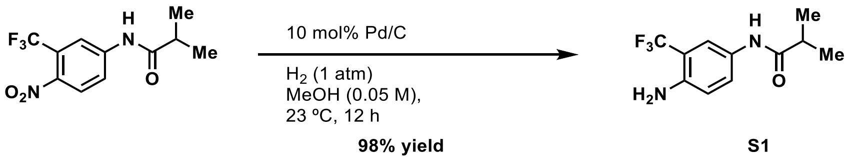

Compound **S1** was prepared following a modified literature procedure.[14] The procedure was modified as follows:

Under an Ar atmosphere, to a 500-mL round-bottomed Schlenk flask equipped with a magnetic stir bar was added Flutamide (2.00 g, 7.20 mmol, 1.00 equiv.), and methanol (144 mL, *c* = 0.05 M). Then, the mixture was stirred at 23°C for 5 min. Pd/C (770 mg, 0.724 mmol, 0.100 equiv.) was added under Ar atmosphere and argon was purged through the reaction mixture for 5 min using a venting needle. The atmosphere was evacuated and a balloon of H_2 was pierced through the septum to ensure a H_2 atmosphere. The reaction mixture was stirred on a hydrogen atmosphere. The reaction was monitored by TLC. After full consumption of the starting material (12 hours), the reaction mixture was filtered through celite. The resulting mixture was concentrated by rotary evaporation under reduced pressure. The residue was purified by silica gel chromatography by eluting with EtOAc/pentane (30:70 (v/v)) to afford 1.7 g (95%) of the title compound **S1** as a pink solid.

$\mathbf{R_f}$ = 0.67 (EtOAc/pentane (60:40 (v/v)) (UV)).

NMR Spectroscopy:

^{1}H NMR (500 MHz, DMSO-d_6, 25°C, δ): 9.62 (s, 1H), 7.71 (d, *J* = 2.5 Hz, 1H), 7.41 (d, *J* = 8.5 Hz, 1H), 6.77 (dd, *J* = 8.5, 2.5 Hz, 1H), 5.30 (s, 2H), 2.50 (h, *J* = 6.9 Hz, 1H), 1.06 (d, *J* = 6.8 Hz, 6H).

^{13}C NMR (125 MHz, DMSO-d_6, 25°C, δ): 174.7, 142.0, 128.3, 125.1, 125.0 (q, *J* = 270 Hz), 117.2, 116.9 (q, *J* = 5.0 Hz), 110.3 (q. *J* = 30.0 Hz), 34.8, 19.5.

^{19}F NMR (470 MHz, DMSO-d_6, 25°C, δ): −61.5.

HRMS-ESI(m/z) calc'd for $NaC_{11}H_{13}N_2O_1F_3^+$ [M+Na]$^+$, 269.0872; found, 269.0874; deviation: +0.7 ppm.

Melting point: 117 – 119 °C.

Flutamide-dervied benzonitrile 14

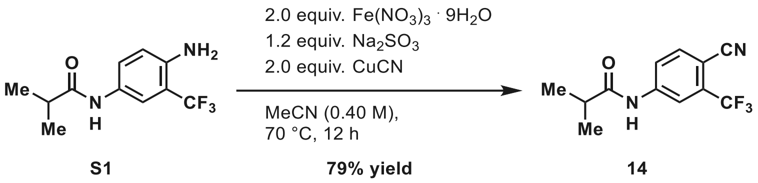

Caution: When performing reactions in pressurized systems (such as closed vials and pressure tubes), a blast shield must be used to minimize personal damage in case of an accident. See safety statement on page S6–S7.

Under an ambient atmosphere, to a 4-mL borosilicate vial equipped with a Teflon-coated magnetic stir bar were added reduced Flutamide **S1** (123 mg, 0.500 mmol, 1.00 equiv.), Na_2SO_3 (75.7 mg, 0.600 mmol, 2.00 equiv.), CuCN (89.6 mg, 1.00 mmol, 2.00 equiv.) and iron (III) nitrate nonahydrate (404 mg, 1.00 mmol, 2.00 equiv.). Then, acetonitrile (MeCN) (1.25 mL, *c* = 0.4 M) was added. The vessel was sealed with a septum cap and heated at 70°C for 12 h in an aluminum block on a heating plate. After cooling to 23 °C, the resulting mixture was passed through a pad of celite by eluting with ethyl acetate (50 mL). Then, the solvent was evaporated by rotary evaporation under reduced pressure. The residue was purified by silica gel chromatography by eluting with EtOAc/pentane (25:75 (v/v)) to afford 101 mg (79%) of the title compound **14** as yellow colored solid.

R_f = 0.41 (EtOAc in pentane = 40%, v/v (UV)).

NMR Spectroscopy:

^{1}H NMR (500 MHz, DMSO-d_6, 25°C, δ): δ 10.58 (s, 1H), 8.27 (d, *J* = 2.0 Hz, 1H), 8.03 (d, *J* = 8.6 Hz, 1H), 7.99 (dd, *J* = 8.6, 2.1 Hz, 1H), 2.62 (h, *J* = 6.8 Hz, 1H), 1.12 (d, *J* = 6.9 Hz, 6H).

^{13}C NMR (125 MHz, DMSO-d_6, 25°C, δ): δ 176.5, 144.0, 136.4, 131.7 (q, *J* = 31.5 Hz), 122.4 (q, *J* = 274.6 Hz), 121.8, 116.3 (q, *J* = 6.3 Hz), 115.8, 101.2 (q, *J* = 1.3 Hz), 35.3, 19.1.

^{19}F NMR (470 MHz, DMSO-d_6, 25°C, δ): −61.4.

HRMS-EI(m/z) calc'd for $C_{12}H_{11}N_2O_1F_3^+$ [M]$^+$, 256.0817; found, 256.0814; deviation: −1.5 ppm.

Melting point: 132 – 134 °C.

2,6-Dichloro-3-methylbenzonitrile (15)

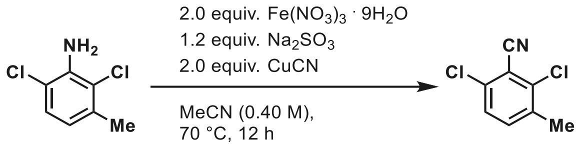

87% yield **15**

Caution: When performing reactions in pressurized systems (such as closed vials and pressure tubes), a blast shield must be used to minimize personal damage in case of an accident. See safety statement on page S6–S7.

Under an ambient atmosphere, to a 4-mL borosilicate vial equipped with a Teflon-coated magnetic stir bar were added 2,6-Dichlor-3-methylaniline (88.1 mg, 0.500 mmol, 1.00 equiv.), Na_2SO_3 (75.7 mg, 0.600 mmol, 2.00 equiv.), CuCN (89.6 mg, 1.00 mmol, 2.00 equiv.) and iron (III) nitrate nonahydrate (404 mg, 1.00 mmol, 2.00 equiv.). Then, acetonitrile (MeCN) (1.25 mL, *c* = 0.4 M) was added. The vessel was sealed with a septum cap and heated at 70°C for 12 h in an aluminum block on a heating plate. After cooling to 23 °C, the resulting mixture was passed through a pad of celite by eluting with ethyl acetate (50 mL). Then, the solvent was evaporated by rotary evaporation under reduced pressure. The residue was purified by silica gel chromatography by eluting with EtOAc/pentane (2:98 (v/v)) to afford 81 mg (87%) of the title compound **15** as orange colored solid.

R_f = 0.44 (EtOAc in pentane = 10%, v/v (UV)).

NMR Spectroscopy:

^{1}H NMR (500 MHz, $CDCl_3$, 25°C, δ): 7.39 (d, *J* = 8.4 Hz, 1H), 7.31 (d, *J* = 8.3 Hz, 1H), 2.40 (s, 3H).

^{13}C NMR (125 MHz, $CDCl_3$, 25°C, δ): 138.0, 136.5, 135.7, 135.3, 127.9, 114.4, 113.9, 20.1.

HRMS-EI(m/z) calc'd for $C_8H_5N_1Cl_2^+$ $[M]^+$, 184.9793; found, 184.9795; deviation: +0.8 ppm.

Melting point: 73 – 75 °C.

Ethyl 4-cyanobenzoate (16)

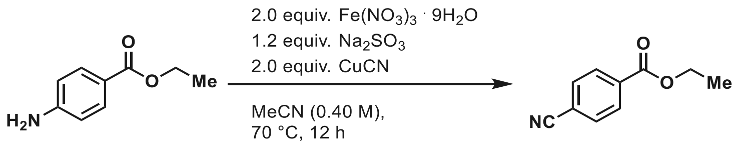

69% yield **16**

Caution: When performing reactions in pressurized systems (such as closed vials and pressure tubes), a blast shield must be used to minimize personal damage in case of an accident. See safety statement on page S6–S7.

Under an ambient atmosphere, to a 4-mL borosilicate vial equipped with a Teflon-coated magnetic stir

bar were added ethyl-4-aminobenzoate (82.6 mg, 0.500 mmol, 1.00 equiv.), Na_2SO_3 (75.7 mg, 0.600 mmol, 2.00 equiv.), CuCN (89.6 mg, 1.00 mmol, 2.00 equiv.) and iron (III) nitrate nonahydrate (404 mg, 1.00 mmol, 2.00 equiv.). Then, acetonitrile (MeCN) (1.25 mL, *c* = 0.4 M) was added. The vessel was sealed with a septum cap and heated at 70°C for 12 h in an aluminum block on a heating plate. After cooling to 23 °C, the resulting mixture was passed through a pad of celite by eluting with ethyl acetate (50 mL). Then, the solvent was evaporated by rotary evaporation under reduced pressure. The residue was purified by silica gel chromatography by eluting with EtOAc/pentane (1:99 (v/v)) to afford 60 mg (69%) of the title compound **16** as orange colored solid.

R*f* = 0.58 (EtOAc in pentane = 10%, v/v (UV)).

NMR Spectroscopy:

^{1}H NMR (500 MHz, $CDCl_3$, 25°C, δ): 8.13 (d, *J* = 8.5 Hz, 2H), 7.73 (d, *J* = 8.5 Hz, 2H), 4.40 (q, *J* = 7.1 Hz, 2H), 1.40 (t, *J* = 7.1 Hz, 3H).

^{13}C NMR (125 MHz, $CDCl_3$, 25°C, δ): 165.0, 134.4, 132.3, 130.2, 118.1, 116.4, 61.9, 14.3.

HRMS-EI(m/z) calc'd for $C_{10}H_9N_1O_2^+$ [M]$^+$, 175.0628; found, 175.0625; deviation: −1.4 ppm.

Melting point: 52 – 54 °C.

4-(1,1,1,3,3,3-Hexafluoro-2-hydroxypropan-2-yl)benzonitrile (17)

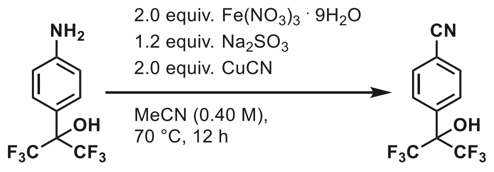

67% yield 17

Caution: When performing reactions in pressurized systems (such as closed vials and pressure tubes), a blast shield must be used to minimize personal damage in case of an accident. See safety statement on page S6–S7.

Under an ambient atmosphere, to a 4-mL borosilicate vial equipped with a Teflon-coated magnetic stir bar were added 2-(4-aminophenyl)-1,1,1,3,3,3-hexafluor-2-propanol (130 mg, 0.500 mmol, 1.00 equiv.), Na_2SO_3 (75.7 mg, 0.600 mmol, 2.00 equiv.), CuCN (89.6 mg, 1.00 mmol, 2.00 equiv.) and iron (III) nitrate nonahydrate (404 mg, 1.00 mmol, 2.00 equiv.). Then, acetonitrile (MeCN) (1.25 mL, *c* = 0.4 M) was added. The vessel was sealed with a septum cap and heated at 70°C for 12 h in an aluminum block on a heating plate. After cooling to 23 °C, the resulting mixture was passed through a pad of celite by eluting with ethyl acetate (50 mL). Then, the solvent was evaporated by rotary evaporation under reduced pressure. The residue was purified by silica gel chromatography by eluting with EtOAc/pentane (2:98 (v/v)) to afford 90 mg (67%) of the title compound **17** as off white solid.

R*f* = 0.27 (EtOAc in pentane = 10%, v/v (UV)).

NMR Spectroscopy:

^{1}H NMR (600 MHz, CD_3OD, 25°C, δ): 7.94 (d, *J* = 9.0 Hz, 2H), 7.88 – 7.84 (m, 2H).

^{13}C NMR (151 MHz, CD_3OD, 25°C, δ): 137.7, 133.4, 129.3 (h, *J* =1. Hz), 124.1 (q, *J* = 288.4 Hz), 118.9, 115.2, 78.4 (h, *J* = 30.2 Hz).

^{19}F NMR (565 MHz, CD_3OD, 23°C, δ): −76.2.

HRMS-EI(m/z) calc'd for $C_{10}H_5N_1O_1F_6^+$ $[M]^+$, 269.0269; found, 269.0270; deviation: +0.0 ppm.

Melting point: 117 – 119 °C.

rac-Aminoglutethimide-dervied benzonitrile 18

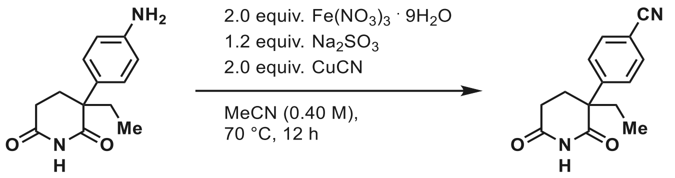

rac-**S2** **64% yield** *rac*-**18**

Caution: When performing reactions in pressurized systems (such as closed vials and pressure tubes), a blast shield must be used to minimize personal damage in case of an accident. See safety statement on page S6–S7.

Under an ambient atmosphere, to a 4-mL borosilicate vial equipped with a Teflon-coated magnetic stir bar were added Aminoglutethimide (*rac*-**S2**) (116 mg, 0.500 mmol, 1.00 equiv.), Na_2SO_3 (75.7 mg, 0.600 mmol, 2.00 equiv.), CuCN (89.6 mg, 1.00 mmol, 2.00 equiv.) and iron (III) nitrate nonahydrate (404 mg, 1.00 mmol, 2.00 equiv.). Then, acetonitrile (MeCN) (1.25 mL, *c* = 0.4 M) was added. The vessel was sealed with a septum cap and heated at 70°C for 12 h in an aluminum block on a heating plate. After cooling to 23 °C, the resulting mixture was passed through a pad of celite by eluting with ethyl acetate (50 mL). Then, the solvent was evaporated by rotary evaporation under reduced pressure. The residue was purified by silica gel chromatography by eluting with EtOAc/pentane (25:75 (v/v)) to afford 78 mg (64%) of the title compound *rac*-**18** as off white solid.

R_f = 0.31 (EtOAc in pentane = 40%, v/v (UV)).

NMR Spectroscopy:

^{1}H NMR (500 MHz, DMSO-d_6, 25°C, δ): 10.99 (s, 1H), 7.86 (d, *J* = 8.6 Hz, 2H), 7.51 (d, *J* = 8.6 Hz, 2H), 2.48 (t, *J* = 3.9 Hz, 1H), 2.44 – 2.35 (m, 1H), 2.21 (td, *J* = 13.2, 4.3 Hz, 1H), 2.10 (ddd, *J* = 17.5, 12.5, 4.8 Hz, 1H), 1.88 (m, 2H), 0.75 (t, *J* = 7.4 Hz, 3H).

^{13}C NMR (125 MHz, DMSO-d_6, 25°C, δ): 175.0, 172.5, 145.7, 132.7, 127.6, 118.5, 110.1, 50.7, 31.6, 28.9, 26.0, 8.9.

HRMS-EI(m/z) calc'd for $C_{14}H_{14}N_2O_2$ $[M]^+$, 242.1049; found, 242.1050; deviation: −0.3 ppm.

Melting point: 140 – 142 °C.

2-Bromoterephthalonitrile (19)

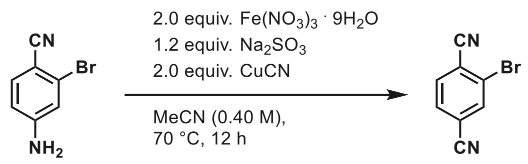

Caution: When performing reactions in pressurized systems (such as closed vials and pressure tubes), a blast shield must be used to minimize personal damage in case of an accident. See safety statement on page S6–S7.

Under an ambient atmosphere, to a 4-mL borosilicate vial equipped with a Teflon-coated magnetic stir bar were added 4-amino-3-brombenzonitrile (98.6 mg, 0.500 mmol, 1.00 equiv.), Na_2SO_3 (75.7 mg, 0.600 mmol, 2.00 equiv.), CuCN (89.6 mg, 1.00 mmol, 2.00 equiv.) and iron (III) nitrate nonahydrate (404 mg, 1.00 mmol, 2.00 equiv.). Then, acetonitrile (MeCN) (1.25 mL, *c* = 0.4 M) was added. The vessel was sealed with a septum cap and heated at 70°C for 12 h in an aluminum block on a heating plate. After cooling to 23 °C, the resulting mixture was passed through a pad of celite by eluting with ethyl acetate (50 mL). Then, the solvent was evaporated by rotary evaporation under reduced pressure. The residue was purified by silica gel chromatography by eluting with EtOAc/pentane (2:98 (v/v)) to afford 75 mg (72%) of the title compound **19** as off white solid.

$\mathbf{R}_f$ = 0.27 (EtOAc in pentane = 10%, v/v (UV)).

NMR Spectroscopy:

^{1}H NMR (500 MHz, $CDCl_3$, 25°C, δ): 7.99 (d, *J* = 1.5 Hz, 1H), 7.79 (d, *J* = 8.0 Hz, 1H), 7.73 (dd, *J* = 8.1, 1.5 Hz, 1H).

^{13}C NMR (125 MHz, $CDCl_3$, 25°C, δ): 136.4, 134.8, 131.1, 126.3, 120.3, 117.8, 115.9, 115.8.

HRMS-EI(m/z) calc'd for $C_8H_3N_2Br_1^+$ $[M]^+$, 205.9474; found, 205.9475; deviation: +0.4 ppm.

Melting point: 191 – 193 °C.

Methyl 3-cyanothiophene-2-carboxylate (20)

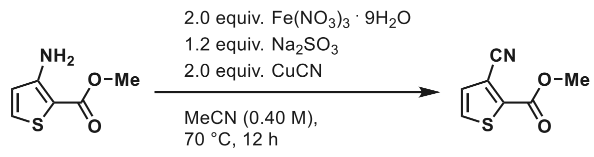

Caution: When performing reactions in pressurized systems (such as closed vials and pressure tubes), a blast shield must be used to minimize personal damage in case of an accident. See safety statement on page S6–S7.

Under an ambient atmosphere, to a 4-mL borosilicate vial equipped with a Teflon-coated magnetic stir bar were added methyl 3-amino-2-thiophenecarboxylate (78.6 mg, 0.500 mmol, 1.00 equiv.), Na_2SO_3

(75.7 mg, 0.600 mmol, 2.00 equiv.), CuCN (89.6 mg, 1.00 mmol, 2.00 equiv.) and iron (III) nitrate nonahydrate (404 mg, 1.00 mmol, 2.00 equiv.). Then, acetonitrile (MeCN) (1.25 mL, *c* = 0.4 M) was added. The vessel was sealed with a septum cap and heated at 70°C for 12 h in an aluminum block on a heating plate. After cooling to 23 °C, the resulting mixture was passed through a pad of celite by eluting with ethyl acetate (50 mL). Then, the solvent was evaporated by rotary evaporation under reduced pressure. The residue was purified by silica gel chromatography by eluting with EtOAc/pentane (10:90 (v/v)) to afford 55 mg (66%) of the title compound **20** as off white solid.

R_f = 0.25 (EtOAc in pentane = 20%, v/v (UV)).

NMR Spectroscopy:

^{1}H NMR (500 MHz, $CDCl_3$, 25°C, δ): 7.62 (d, *J* = 5.2 Hz, 1H), 7.35 (d, *J* = 5.2 Hz, 1H), 3.96 (s, 3H).

^{13}C NMR (125 MHz, $CDCl_3$, 25°C, δ): 160.1, 140.3, 132.2, 131.4, 114.5, 113.7, 53.1.

HRMS-EI(m/z) calc'd for $C_7H_5N_1O_2S_1^+$ $[M]^+$, 167.0035; found, 167.0037; deviation: +1.4 ppm.

Melting point: 104 – 106 °C.

Gram scale reaction

4-(1,1,1,3,3,3-Hexafluoro-2-hydroxypropan-2-yl)benzonitrile (17)

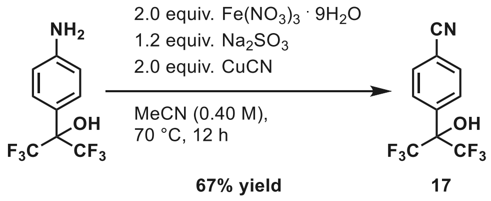

Caution: When performing reactions in pressurized systems (such as closed vials and pressure tubes), a blast shield must be used to minimize personal damage in case of an accident. See safety statement on page S6–S7.

Under an ambient atmosphere, to a 100-mL Ace pressure tube equipped with a Teflon-coated magnetic stir bar was added 2-(4-aminophenyl)-1,1,1,3,3,3-hexafluor-2-propanol (5.0 g, 19.3 mmol, 1.00 equiv.), Na_2SO_3 (2.92 g, 38.6 mmol, 1.20 equiv.), CuCN (3.46 g, 38.6 mmol, 2.00 equiv.) and iron (III) nitrate nonahydrate (15.6 g, 38.6 mmol, 2.00 equiv.) (Fig. S4, A). Then, acetonitrile (MeCN) (48 mL, *c* = 0.40 M) was added. The pressure tube was quickly sealed with a Teflon cap containing an O-ring (Fig. S4, B). Subsequently, the mixture was heated in an oil bath and stirred at 70 °C oil bath temperature for 12 h (Fig. S4, C). After cooling to 23 °C, the resulting mixture was passed through a pad of celite by eluting with ethyl acetate (500 mL). Then, the solvent was evaporated by rotary evaporation under reduced pressure. The residue was purified by silica gel chromatography by eluting with EtOAc/pentane (2:98 (v/v)) to afford 3.4 g (65%) of the title compound **17** as off white solid.

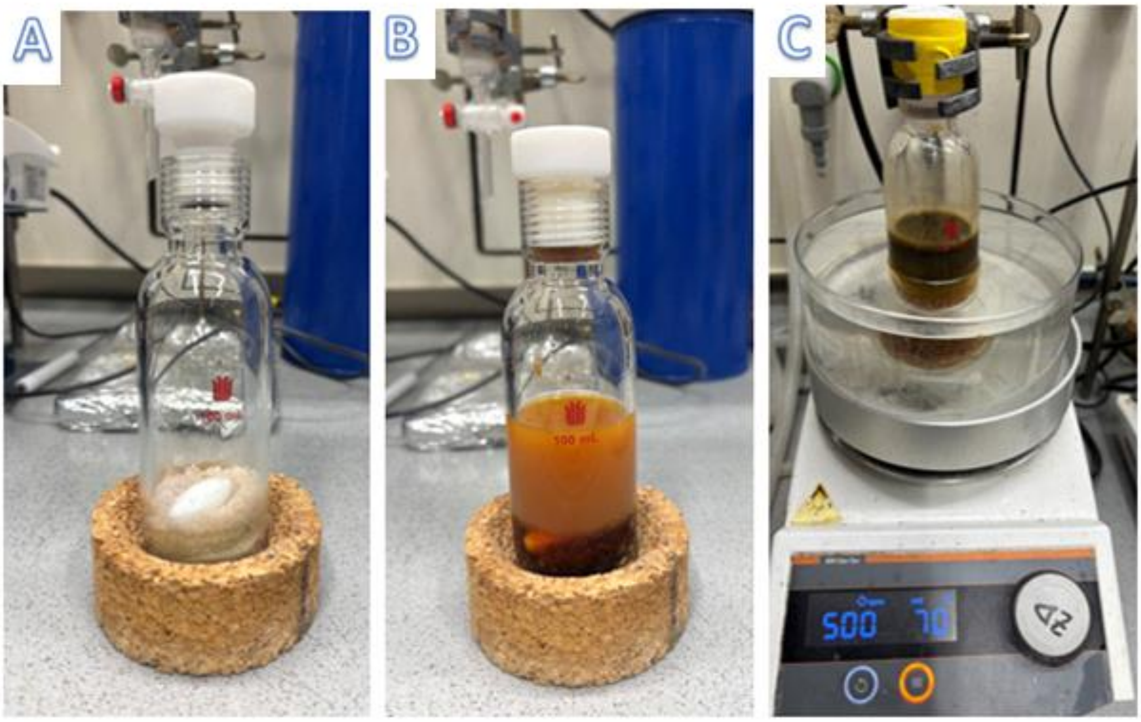

Fig S4. Reaction set-up for 5 g scale reaction for the synthesis of compound **17**. **A)** Weigh all the solids in a 100-mL Ace pressure tube equipped with a magnetic stir bar. **B)** After addition of all the solids, acetonitrile was added and sealed with a Teflon cap containing an O-ring. **C)** The reaction is heated at 70°C for 12 h in an aluminum block on a heating plate.

R_f = 0.27 (EtOAc in pentane = 10%, v/v (UV)).

NMR Spectroscopy:

> **^{1}H NMR** (600 MHz, CD_3OD, 25°C, δ): 7.94 (d, *J* = 9.0 Hz, 2H), 7.88 – 7.84 (m, 2H).
>
> **^{13}C NMR** (151 MHz, CD_3OD, 25°C, δ): 137.7, 133.4, 129.3 (h, *J* =1. Hz), 124.1 (q, *J* = 288.4 Hz), 118.9, 115.2, 78.4 (h, *J* = 30.2 Hz).
>
> **^{19}F NMR** (565 MHz, CD_3OD, 25°C, δ): −76.2.

HRMS-EI(m/z) calc'd for $C_{10}H_5N_1O_1F_6^+$ $[M]^+$, 269.0269; found, 269.0270; deviation: +0.0 ppm.

Melting point: 117 – 119 °C.

Diversifications after Deaminative Cyanation

2-(4-(Aminomethyl)phenyl)-1,1,1,3,3,3-hexafluoropropan-2-ol (22)

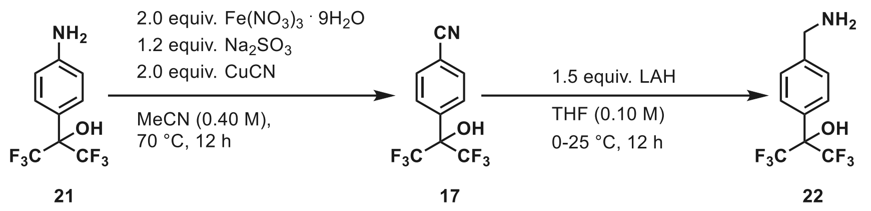

Caution: When performing reactions in pressurized systems (such as closed vials and pressure tubes), a blast shield must be used to minimize personal damage in case of an accident. See safety statement on page S6–S7.

Under an ambient atmosphere, to a 20-mL borosilicate vial equipped with a Teflon-coated magnetic stir bar were added 2-(4-aminophenyl)-1,1,1,3,3,3-hexafluor-2-propanol (**21**) (389 mg, 1.50 mmol, 1.00 equiv.), Na_2SO_3 (227 mg, 1.80 mmol, 2.00 equiv.), CuCN (269 mg, 3.00 mmol, 2.00 equiv.) and iron (III) nitrate nonahydrate (1.22 g, 3.00 mmol, 2.00 equiv.). Then, acetonitrile (MeCN) (3.75 mL, *c* = 0.40 M) was added. The vessel was sealed with a septum cap and heated at 70°C for 12 h in an aluminum block on a heating plate. After cooling to 23 °C, the resulting mixture was passed through a pad of celite by eluting with ethyl acetate (150 mL). Then, the solvent was evaporated by rotary evaporation under reduced pressure. The residue was purified by silica gel chromatography by eluting with EtOAc/pentane (2:98 (v/v)) to afford 271 mg (67%) of the title compound **17** as off white solid.

Compound **22** was prepared following a modified literature procedure.[15] The procedure was modified as follows:

Under inert atmosphere, compound **17** (271 mg, 1.01 mmol, 1.00 equiv.) was added to a 100 mL two-necked round bottomed flask equipped with a Teflon-coated magnetic stir bar. Then, THF (10 mL) was added and the mixture was stirred for 5 min at 0 °C. Lithium aluminum hydride solution (1M in THF, 1.5 mL, 1.51 mmol, 1.50 equiv.) was added to the mixture and stirred at 23 °C for 12 hour. Then, the mixture was diluted with a saturated aqueous solution of NH_4Cl (20 mL) and extracted with ethyl acetate (3 × 20 mL). The combined organic layers were dried over Na_2SO_4, filtered and concentrated under reduced pressure. The residue was purified by silica gel chromatography by eluting with MeOH/CH_2Cl_2 (20:80 (v/v)) to afford 215 mg (78%) of the title compound **22** as off-white solid.

Overall yield over two steps: 52%.

R*f* = 0.31 (MeOH in CH_2Cl_2 = 20%, v/v (UV)).

NMR Spectroscopy:

^{1}H NMR (500 MHz, CD_3CN, 25°C, δ): 7.69 (d, *J* = 8.1 Hz, 2H), 7.46 (d, *J* = 8.3 Hz, 2H), 3.85 (s, 2H), 3.34 (s, 2H).

^{13}C NMR (125 MHz, CD_3CN, 25°C, δ): 144.8, 130.6, 128.6, 127.9, 124.1 (q, *J* = 285 Hz), 78.2 (h, *J* = 28.7 Hz), 45.4.

[19]**F NMR** (470 MHz, CD_3CN, 25°C, δ): −75.5.

HRMS-ESI(m/z) calc'd for $C_{10}H_{10}N_1O_1F_6^+$ [M+H]$^+$, 274.0661; found, 274.0660; deviation: −0.4 ppm.

2-(4-(1H-tetrazol-5-yl)phenyl)-1,1,1,3,3,3-hexafluoropropan-2-ol (23)

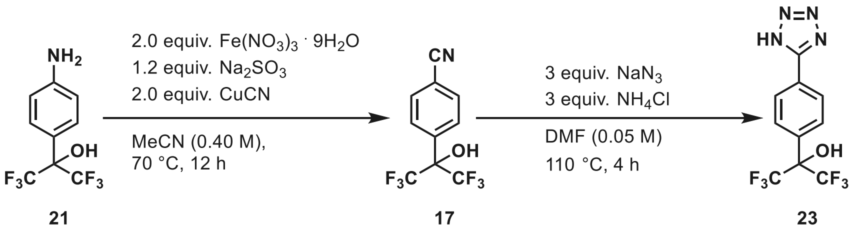

Caution: When performing reactions in pressurized systems (such as closed vials and pressure tubes), a blast shield must be used to minimize personal damage in case of an accident. See safety statement on page S6–S7.

Under an ambient atmosphere, to a 20-mL borosilicate vial equipped with a Teflon-coated magnetic stir bar were added 2-(4-aminophenyl)-1,1,1,3,3,3-hexafluor-2-propanol (**21**) (389 mg, 1.50 mmol, 1.00 equiv.), Na_2SO_3 (227 mg, 1.80 mmol, 2.00 equiv.), CuCN (269 mg, 3.00 mmol, 2.00 equiv.) iron (III) nitrate nonahydrate (1.22 g, 3.00 mmol, 2.00 equiv.). Then, acetonitrile (MeCN) (3.75 mL, c = 0.40 M) was added, the vial was quickly sealed with a septum cap and stirred at 70 °C for 12 h. After cooling to 23 °C, the resulting mixture was passed through a pad of celite by eluting with ethyl acetate (150 mL). Then, the solvent was evaporated by rotary evaporation under reduced pressure. The residue was purified by silica gel chromatography by eluting with EtOAc/pentane (2:98 (v/v)) to afford 271 mg (67%) of the title compound **17** as off white solid.

Compound **23** was prepared following a modified literature procedure.[16] The procedure was modified as follows:

Caution: Handle sodium azide with extreme caution in a well-ventilated fume hood. Avoid contact with water, acids, and heavy metals, as toxic and explosive compounds (e.g., hydrazoic acid, metal azides) may form. Use appropriate PPE including gloves, lab coat, and eye/face protection. A blast shield must be used to minimize personal damage in case of an accident.

Under an ambient temperature, compound **17** (271 mg, 1.01 mmol, 1.00 equiv.) was added to a 100 mL round-bottomed flask equipped with a Teflon-coated magnetic stir bar, followed by the addition of DMF (20 mL, c = 0.05 M). The mixture was stirred at 23 °C for 5 minutes. Sodium azide (196 mg, 3.03 mmol, 3.00 equiv.) and ammonium chloride (162 mg, 3.03 mmol, 3.00 equiv.) were then added. The vessel was heated in an oil bath at 110 °C oil bath temperature and the mixture stirred for 4 hours. After cooling to 23 °C, the mixture was diluted with water (20 mL) and extracted with ethyl acetate (3 × 20 mL).The combined organic layers were dried over Na_2SO_4, filtered and concentrated under reduced pressure. The residue was purified by silica gel chromatography by eluting with MeOH/CH_2Cl_2 (20:80 (v/v)) to afford 300 mg (96%) of the title compound **23** as off-white solid.

Overall yield over two steps: 64%.

R*f* = 0.62 (MeOH in CH_2Cl_2 = 20%, v/v (UV)).

NMR Spectroscopy:

^{1}H NMR (500 MHz, DMSO-*d*$_6$, 25°C, δ): 8.84 (s, 1H), 8.14 (d, *J* = 8.7 Hz, 2H), 7.77 (d, *J* = 8.3 Hz, 2H).

^{13}C NMR (125 MHz, DMSO-*d*$_6$, 25°C, δ): 158.1, 131.4, 130.5, 127.3, 126.3, 122.9 (q, *J* = 287.5 Hz), 76.8 (q, *J* = 28.7 Hz).

^{19}F NMR (470 MHz, DMSO-*d*$_6$, 25°C, δ): −73.8.

HRMS-EI(m/z) calc'd for $C_{10}H_6N_4O_1F_6^+$ [M]$^+$, 312.0440; found, 312.0437; deviation: −0.9 ppm.

4-(1,1,1,3,3,3-Hexafluoro-2-hydroxypropan-2-yl)benzamide (24)

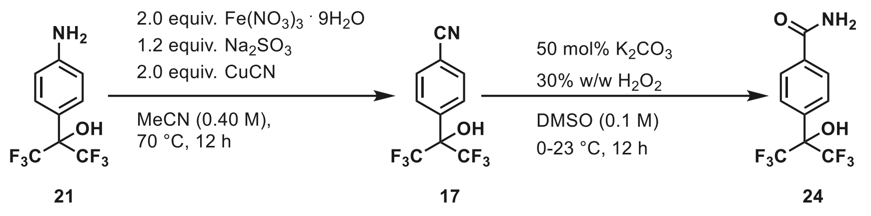

Caution: When performing reactions in pressurized systems (such as closed vials and pressure tubes), a blast shield must be used to minimize personal damage in case of an accident. See safety statement on page S6–S7.

Under an ambient atmosphere, to a 20-mL borosilicate vial equipped with a Teflon-coated magnetic stir bar were added 2-(4-aminophenyl)-1,1,1,3,3,3-hexafluor-2-propanol (**21**) (389 mg, 1.50 mmol, 1.00 equiv.), Na_2SO_3 (227 mg, 1.80 mmol, 2.00 equiv.), CuCN (269 mg, 3.00 mmol, 2.00 equiv.) and iron (III) nitrate nonahydrate (1.22 g, 3.00 mmol, 2.00 equiv.). Then, acetonitrile (MeCN) (3.75 mL, *c* = 0.40 M) was added. The vessel was sealed with a septum cap and heated at 70°C for 12 h in an aluminum block on a heating plate. After cooling to 23 °C, the resulting mixture was passed through a pad of celite by eluting with ethyl acetate (150 mL). Then, the solvent was evaporated by rotary evaporation under reduced pressure. The residue was purified by silica gel chromatography by eluting with EtOAc/pentane (2:98 (v/v)) to afford 271 mg (67%) of the title compound **17** as off white solid.

Compound **24** was prepared following a modified literature procedure.[17] The procedure was modified as follows:

Under an ambient temperature, to a 50 mL round bottomed flask equipped with a Teflon-coated magnetic stir bar were added compound **17** (271 mg, 1.01 mmol, 1.00 equiv.) and K_2CO_3 (69.6 mg, 0.503 mmol, 0.500 equiv.). Then, DMSO (10 mL) was added and the mixture was stirred at 0 °C for 5 min. Hydrogen peroxide solution (30% w/w in H_2O, 3.5 mL) was added and the resulting mixture stirred at 23 °C for 12 hours. The mixture was diluted with water (10 mL) and extracted with ethyl acetate (3 × 20 mL). The combined organic layers were dried over Na_2SO_4, filtered and concentrated under reduced pressure. The residue was purified by silica gel chromatography by eluting with EtOAc/pentane (60:40 (v/v)) to afford 268 mg (93%) of the title compound **24** as off-white solid.

Overall yield over two steps: 62%.

R*f* = 0.32 (EtOAc in pentane = 70%, v/v (UV)).

NMR Spectroscopy:

^{1}H NMR (500 MHz, DMSO-d_6, 25°C, δ): 8.85 (s, 1H), 7.99 (d, *J* = 8.4 Hz, 2H), 7.81 (d, *J* = 290 Hz 2H), 7.77 (d, *J* = 8.2 Hz, 2H).

^{13}C NMR (125 MHz, DMSO-d_6, 25°C, δ): 167.3, 136.2, 133.5, 127.8, 126.8, 122.8 (q, *J* = 287.5 Hz), 76.9 (h, *J* = 28.7 Hz).

^{19}F NMR (470 MHz, DMSO-d_6, 25°C, δ): −73.9.

HRMS-EI(m/z) calc'd for $C_{10}H_7N_1O_2F_6^+$ $[M]^+$, 287.0375; found, 287.0375; deviation: +0.0 ppm.

Melting point: 160 – 162 °C.

4-(1,1,1,3,3,3-Hexafluoro-2-hydroxypropan-2-yl)benzoic acid (25)

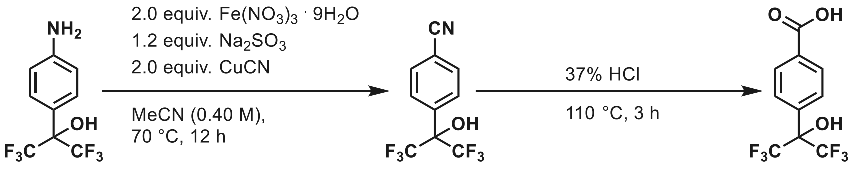

21 17 25

Caution: When performing reactions in pressurized systems (such as closed vials and pressure tubes), a blast shield must be used to minimize personal damage in case of an accident. See safety statement on page S6–S7.

Under an ambient atmosphere, to a 20-mL borosilicate vial equipped with a Teflon-coated magnetic stir bar were added 2-(4-aminophenyl)-1,1,1,3,3,3-hexafluor-2-propanol (**21**) (389 mg, 1.50 mmol, 1.00 equiv.), Na_2SO_3 (227 mg, 1.80 mmol, 2.00 equiv.), CuCN (269 mg, 3.00 mmol, 2.00 equiv.) iron (III) nitrate nonahydrate (1.22 g, 3.00 mmol, 2.00 equiv.). Then, acetonitrile (MeCN) (3.75 mL, *c* = 0.40 M) was added. The vessel was sealed with a septum cap and heated at 70°C for 12 h in an aluminum block on a heating plate. After cooling to 23 °C, the resulting mixture was passed through a pad of celite by eluting with ethyl acetate (150 mL). Then, the solvent was evaporated by rotary evaporation under reduced pressure. The residue was purified by silica gel chromatography by eluting with EtOAc/pentane (2:98 (v/v)) to afford 271 mg (67%) of the title compound **17** as off white solid.

Compound **25** was prepared following a modified literature procedure.[18] The procedure was modified as follows:

Under an ambient temperature, to a 25 mL round bottomed flask equipped with a Teflon-coated magnetic stir bar were added compound **17** (271 mg, 1.01 mmol, 1.00 equiv.) and 37% HCl (aqueous, 3 mL). Then, the mixture was stirred at 110 °C for 3 hours. After cooling to 23 °C, the mixture was diluted with ice water (15 mL) and extracted with ethyl acetate (2 × 20 mL). The combined organic layers were washed with saturated aqueous $NaHCO_3$ solution (10 mL). The aqueous layer was extracted with ethyl acetate (10 mL) and the organic phase combined with the organic layers of the

first extraction. Then, the aqueous phase was washed with 6M HCl and extracted again with ethyl acetate (3 × 10 mL). The combined organic layers were then dried over Na_2SO_4, filtered and concentrated under reduced pressure to afford 276 mg (95%) of the title compound **25** as off-white solid.

Overall yield over two steps: 64%.

R*f* = 0.75 (EtOAc in pentane = 70%, v/v (UV)).

NMR Spectroscopy:

^{1}**H NMR** (600 MHz, DMSO-*d6*, 25°C, δ): 13.21 (s, 1H), 8.93 (s, 1H), 8.08 (d, *J* = 8.7 Hz, 2H), 7.82 (d, *J* = 8.3 Hz, 2H).

^{13}**C NMR** (151 MHz, DMSO-*d6*, 25°C, δ): 166.7, 135.1, 132.6, 129.6, 127.2, 122.8 (q, *J* = 288.4 Hz), 77.0 (h, *J* = 28.6 Hz).

^{19}**F NMR** (565 MHz, DMSO-*d6*, 25°C, δ): −73.9.

HRMS-ESI(m/z) calc'd for $C_{10}H_5O_3F_6^-$ [M−H]$^-$, 287.0148; found, 287.0151; deviation: +1.0 ppm.

Melting point: 122 – 124 °C.

Preparation of [18-crown-6-K]$^{15}NO_3$ and [15-crown-5-Na]$N^{17}O_3$

[18-crown-6-K]$^{15}NO_3$

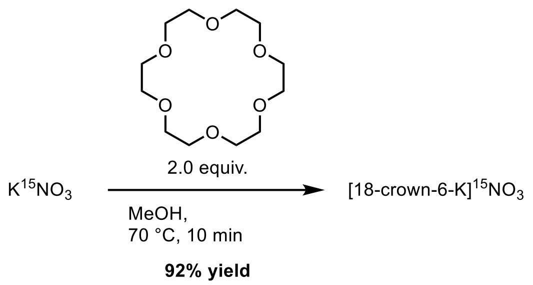

Under an ambient atmosphere, a 50-mL round-bottomed flask equipped with a stir bar and a reflux condenser was charged with 18-crown-6 (513 mg, 1.94 mmol, 1.00 equiv.), $K^{15}NO_3$ (198 mg, 1.94 mmol, 1.00 equiv.), and MeOH (15 mL, c = 0.13 M). The mixture was carefully heated to 70°C over a period of 10 min using an oil bath. After the 70°C temperature was reached (within approximately 10 min), the flask was removed from the oil bath and the mixture was filtered hot using an 8-cm diameter funnel containing a fluted filter paper. The filtrate was allowed to cool to 23°C. Then, the resulting mixture was concentrated by rotary evaporation under reduced pressure yielding an oily colorless residue. The residue was triturated with pentane for 10 min. The resulting colorless solid was filtered off, washed with pentane and diethylether, and dried in high vacuum to afford 655 mg of colorless solid in 92% yield.

NMR Spectroscopy:

^{1}H NMR (600 MHz, MeCN-d_3, 25°C, δ): 3.57 (s).

^{13}C NMR (151 MHz, MeCN-d_3, 25°C, δ): 70.9.

^{15}N NMR (61 MHz, MeCN-d_3, 25°C, δ): −0.52.

^{39}K NMR (19 MHz, MeCN-d_3, 25°C, δ): −2.86.

HRMS-ESI (m/z) calc'd for $C_{12}H_{24}O_6K^+$ [M]$^+$, 303.1204; found, 303.1203; deviation: −0.6 ppm.

calc'd for $^{15}NO_3^-$ [M]$^-$, 62.9854; found, 62.9854; deviation: +0.4 ppm.

Melting point: 220.2 °C (decomposition).

[15-crown-5-Na]$N^{17}O_3$

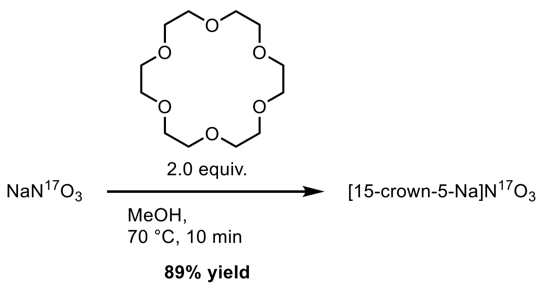

Under an ambient atmosphere, a 50-mL round-bottomed flask equipped with a stir bar and a reflux

condenser was charged with 15-crown-5 (130 mg, 0.588 mmol, 1.00 equiv.), $NaN^{17}O_3$ (50 mg, 0.588 mmol, 1.00 equiv.), and MeOH (4.5 mL, *c* = 0.13 M). The mixture was carefully heated to 70°C over a period of 10 min using an oil bath. After the 70°C temperature was reached (within approximately 10 min), the flask was removed from the oil bath and the mixture was filtered hot using an 8-cm diameter funnel containing a fluted filter paper. The filtrate was allowed to cool to 23°C. Then, the resulting mixture was concentrated by rotary evaporation under reduced pressure yielding an oily colorless residue. The residue was triturated with pentane for 10 min. The resulting colorless solid was filtered off, washed with pentane and diethylether, and dried in high vacuum to afford 159 mg of colorless solid in 89% yield.

NMR Spectroscopy:

^{17}O NMR (54 MHz, MeCN-d_3, 25°C, δ): 420.4 (s).

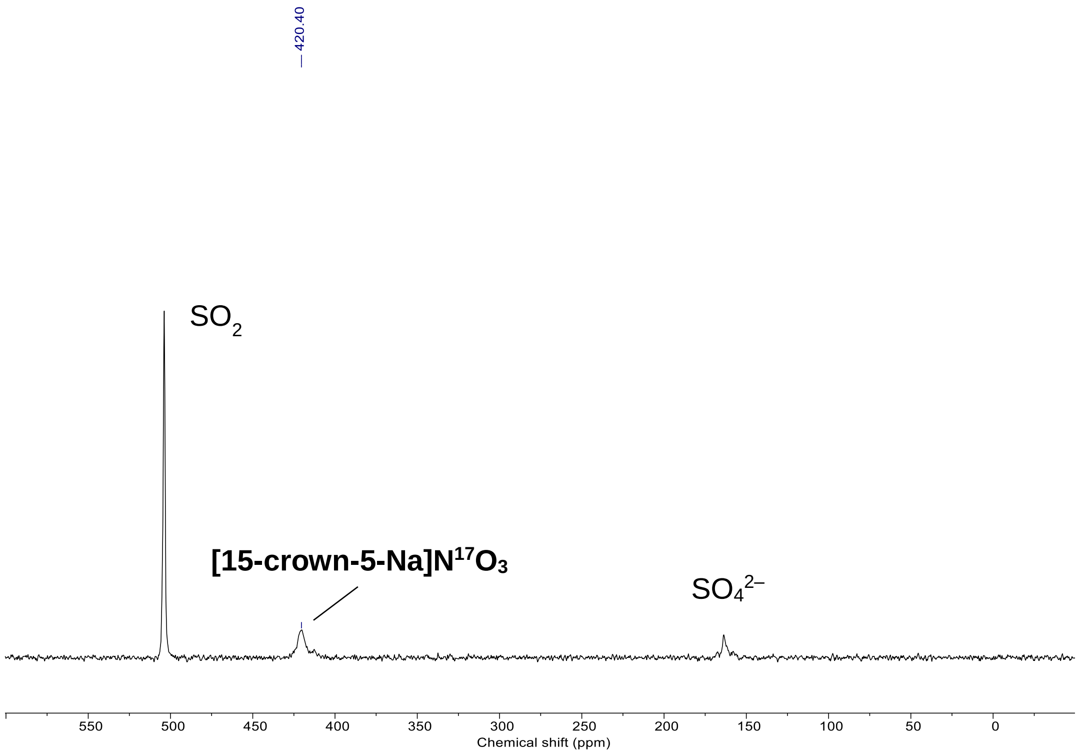

Fig S5. Excerpt of a ^{17}O NMR spectrum containing **[15-crown-5-Na]$N^{17}O_3$** at 54 Hz at 25 °C in MeCN-d_3.

Mechanism Experiments

NO_2 formation from iron nitrate with different SO_2 surrogates

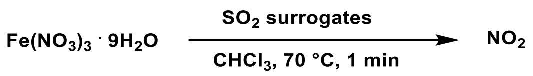

Under an ambient atmosphere, to a 4-mL borosilicate vial equipped with a Teflon-coated magnetic stir bar were added the SO_2 surrogates (0.120 mmol, 1.20 equiv.) and $Fe(NO_3)_3{\cdot}9H_2O$ (80.8 mg, 0.200 mmol, 2.00 equiv.). Then, $CHCl_3$ (0.25 mL, 0.40 M, c = 0.40 M) was added. The vial was sealed with a septum cap and the mixture heated at 75 °C for 1 min until the evolution of a brown gas was observed.

Table S5. Detection of NO_2 gas through vial headspace.

Entry	SO_2 surrogates	NO_2 observation
A	Na_2SO_3	Detected
B	$NaHSO_3$	Detected
C	$Na_2S_2O_4$	Detected

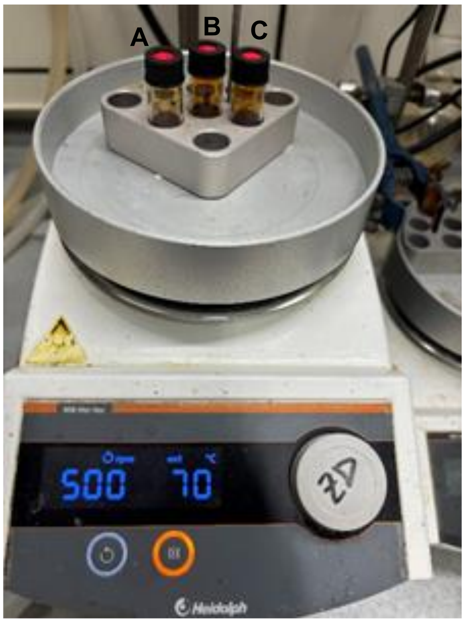

Fig S6. Picture of the reaction vessel headspaces at 70°C. Left: in presence of Na_2SO_3; middle: in presence of $NaHSO_3$; right: in presence of $Na_2S_2O_4$.

NO_2 detection with UV-Vis spectroscopy

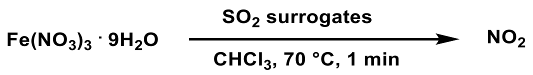

Under an ambient atmosphere, to a 4-mL borosilicate vial equipped with a Teflon-coated magnetic stir bar were added the SO_2 surrogates (0.120 mmol, 1.20 equiv.) and $Fe(NO_3)_3{\cdot}9H_2O$ (80.8 mg, 0.200 mmol, 2.00 equiv.). Then, $CHCl_3$ (0.25 mL, 0.40 M, c = 0.40 M) was added. The vial was sealed

with a septum cap and the mixture heated at 75 °C for 1 min until the evolution of a brown gas was observed. The headspace was taken up with a 10 mL plastic syringe by piercing the septum with a needle. The headspace was then bubbled through a cuvette filled with MeCN (2 mL). Subsequently a UV-Vis spectrum was recorded. The reference spectrum of NO_2 was recorded by bubbling NO_2 through a cuvette containing MeCN.

Table S6. Detection of NO_2 gas through vial headspace.

Entry	SO_2 surrogates	NO_2 observation
1	Na_2SO_3	Detected
2	$NaHSO_3$	Detected
3	$Na_2S_2O_4$	Detected

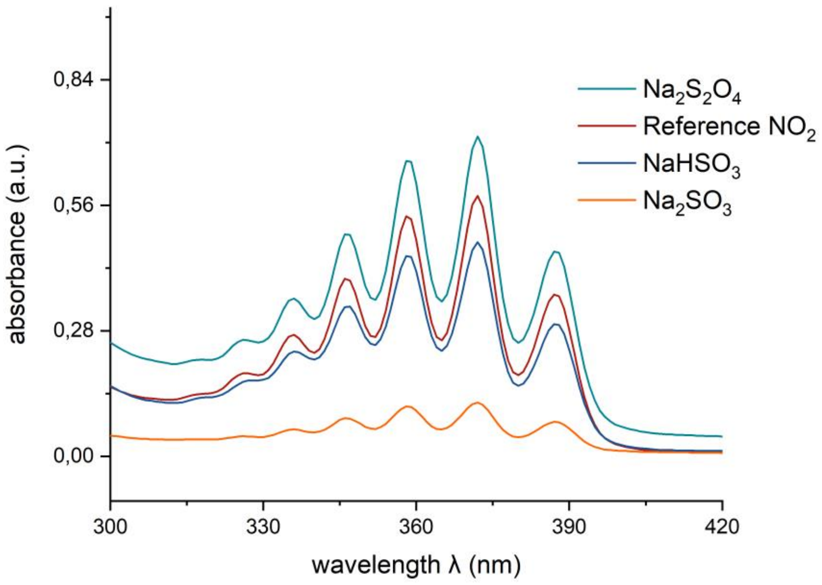

Fig S7. Stacked UV-Vis spectra at 23 °C in MeCN of reaction mixture headspace with reference spectrum of NO_2.

NO_2 detection with gas phase IR spectroscopy

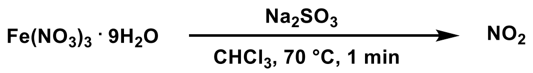

Under an ambient atmosphere, to a 4-mL borosilicate vial equipped with a Teflon-coated magnetic stir bar were added the Na_2SO_3 (15.2 mg, 0.120 mmol, 1.20 equiv.) and $Fe(NO_3)_3 \cdot 9H_2O$ (80.8 mg, 0.200 mmol, 2.00 equiv.). Then, $CHCl_3$ (0.25 mL, 0.40 M, c = 0.40 M) was added. The vial was sealed with a septum cap and the mixture heated at 70 °C for 1 min until the evolution of a brown gas was observed. The headspace was taken up with a 10 mL plastic syringe by piercing the septum with a needle. Subsequently, the headspace was directly injected into the IR spectrometer and an IR

spectrum was recorded. Reference spectra were measured by directly injecting gaseous samples into the IR spectrometer.

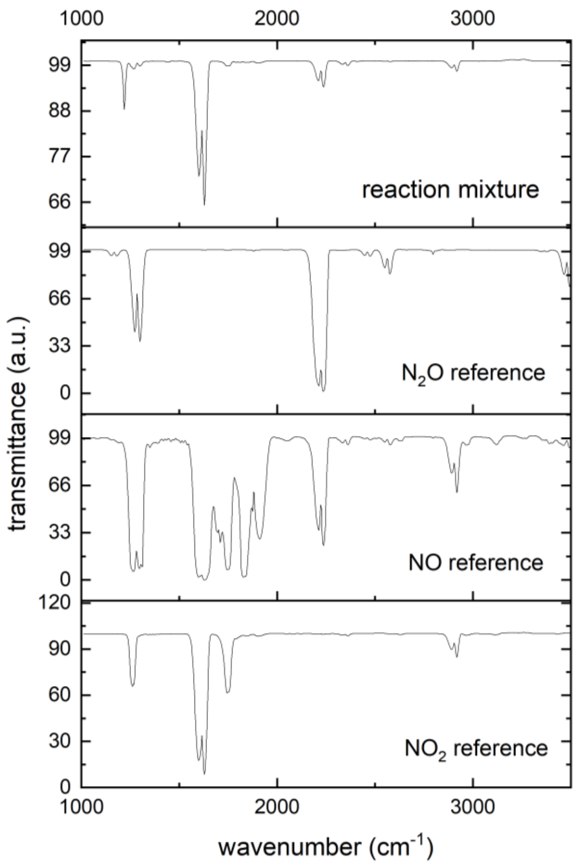

Fig S8. Stacked gas phase IR spectra at 23 °C of reaction mixture headspace (top) with reference spectra of N_2O, NO, and NO_2.

Diazonium trapping experiment

3-(tert-butyl)benzo[d][1,2,3]triazin-4(3H)-one (26)

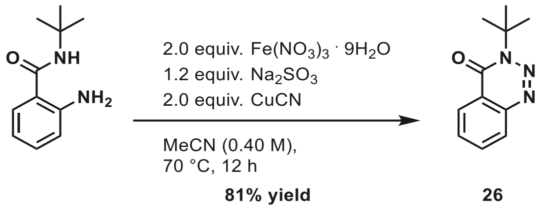

Caution: When performing reactions in pressurized systems (such as closed vials and pressure tubes), a blast shield must be used to minimize personal damage in case of an accident. See safety statement on page S6–S7.

Under an ambient atmosphere, to a 4-mL borosilicate vial equipped with a Teflon-coated magnetic stir bar were added 2-amino-*N*-(*tert*-butyl)-benzamide (96.1 mg, 0.500 mmol, 1.00 equiv.), Na_2SO_3 (75.7 mg, 0.600 mmol, 2.00 equiv.), CuCN (89.6 mg, 1.00 mmol, 2.00 equiv.) and iron (III) nitrate

nonahydrate ($Fe(NO_3)_3 \cdot 9H_2O$) (404 mg, 1.00 mmol, 2.00 equiv.). Then, acetonitrile (MeCN) (1.25 mL, *c* = 0.4 M) were added, the vial was quickly sealed with a septum cap and heated at 70 °C for 12 h in an aluminum heating block while the mixture was stirred. After cooling to 23 °C, the resulting mixture was passed through a pad of celite by eluting with ethyl acetate (50 mL). Then, the solvent was evaporated by rotary evaporation under reduced pressure. The residue was purified by silica gel chromatography by eluting with EtOAc/pentane (8:92 (v/v)) to afford 82 mg (81%) of the title compound **26** as off white solid.

R_f = 0.47 (EtOAc in pentane = 20%, v/v (UV)).

NMR Spectroscopy:

^{1}H NMR (500 MHz, $CDCl_3$, 25°C, δ): 8.32 (dd, *J* = 8.1, 1.5 Hz, 1H), 8.12 – 8.06 (m, 1H), 7.90 (ddd, *J* = 8.3, 7.2, 1.5 Hz, 1H), 7.74 (ddd, *J* = 8.2, 7.2, 1.2 Hz, 1H), 1.81 (s, 9H).

^{13}C NMR (125 MHz, $CDCl_3$, 25°C, δ): 156.3, 143.9, 134.6, 131.9, 127.7, 125.1, 120.9, 65.1, 28.7.

HRMS-EI(m/z) calc'd for $C_{11}H_{13}N_3O_1^+$ $[M]^+$, 203.1053; found, 203.1051; deviation: −0.8 ppm.

Mass spectrometric analysis

With Na_2SO_3

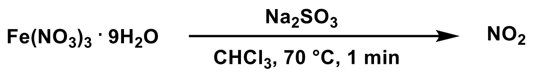

Under an ambient atmosphere, to a 4-mL borosilicate vial equipped with a Teflon-coated magnetic stir bar were added the Na_2SO_3 (15.2 mg, 0.120 mmol, 1.20 equiv.) and $Fe(NO_3)_3{\cdot}9H_2O$ (80.8 mg, 0.200 mmol, 1.00 equiv.). Then, $CHCl_3$ (0.25 mL, c = 0.40 M) was added. The vial was sealed with a septum cap and the mixture heated at 70 °C for 1 min until the evolution of a brown gas was observed. Any formed gases were vented by flushing the vial with Argon for 3 min. Then $CHCl_3$ was removed by rotary evaporation under reduced pressure. The residual solids were suspended in H_2O. The suspension was filtered and an ESI-MS measurement of the solution was carried out.

HRMS-ESI of bisulfate (m/z) calc'd for $H_1O_4S_1$ $[M]^-$, 96.9601; found, 96.9601; deviation: +0.31 ppm.

HRMS-ESI of sodium sulfate (m/z) calc'd for $Na_1O_4S_1$ $[M]^-$, 118.9420; found, 118.9421; deviation: +0.4 ppm.

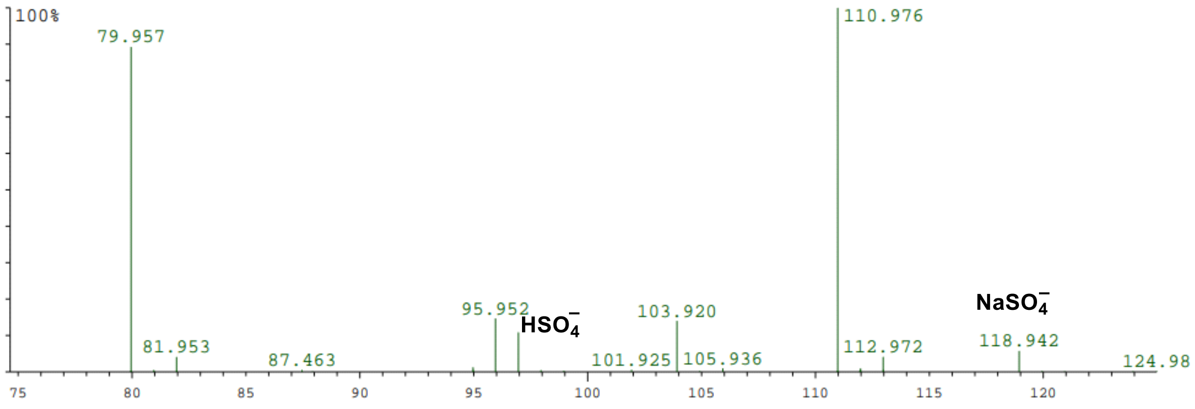

Fig S9. ESI-MS spectrum of H_2O solution after NO_2 formation with Na_2SO_3. **Negative ionization** mode.

With $NaHSO_3$

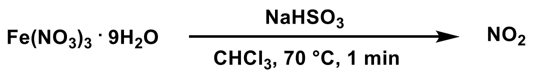

Under an ambient atmosphere, to a 4-mL borosilicate vial equipped with a Teflon-coated magnetic stir bar were added the $NaHSO_3$ (12.5 mg, 0.120 mmol, 1.20 equiv.) and $Fe(NO_3)_3{\cdot}9H_2O$ (80.8 mg, 0.200 mmol, 2.00 equiv.). Then, $CHCl_3$ (0.25 mL, c = 0.40 M) was added. The vial was sealed with a septum cap and the mixture heated at 70 °C for 1 min until the evolution of a brown gas was observed. Any formed gases were vented by flushing the vial with Argon for 3 min. Then $CHCl_3$ was removed by rotary evaporation under reduced pressure. The residual solids were suspended in H_2O. The suspension was filtered and an ESI-MS measurement of the solution was carried out.

HRMS-ESI of bisulfate (m/z) calc'd for $H_1O_4S_1$ [M]⁻, 96.9601; found, 96.9600; deviation: −0.7 ppm.

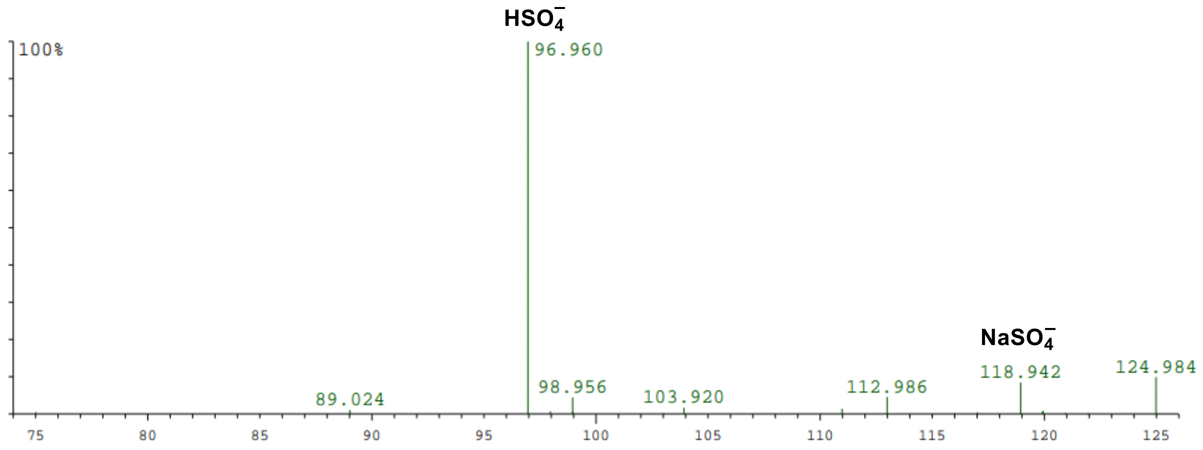

Fig S10. ESI-MS spectrum of H_2O solution after NO_2 formation with $NaHSO_3$. **Negative ionization** mode.

With $Na_2S_2O_4$

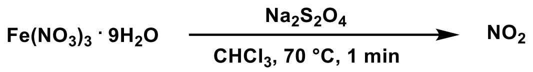

Under an ambient atmosphere, to a 4-mL borosilicate vial equipped with a Teflon-coated magnetic stir bar were added the $Na_2S_2O_4$ (20.9 mg, 0.120 mmol, 1.20 equiv.) and $Fe(NO_3)_3{\cdot}9H_2O$ (80.8 mg, 0.200 mmol, 2.00 equiv.). Then, $CHCl_3$ (0.25 mL, *c* = 0.40 M) was added. The vial was sealed with a septum cap and the mixture heated at 70 °C for 1 min until the evolution of a brown gas was observed. Any formed gases were vented by flushing the vial with Argon for 3 min. Then, $CHCl_3$ was removed by rotary evaporation under reduced pressure. The residual solids were suspended in H_2O. The suspension was filtered and an ESI-MS measurement of the solution was carried out.

HRMS-ESI of bisulfate (m/z) calc'd for $H_1O_4S_1$ [M]⁻, 96.9601; found, 96.9601; deviation: +0.4 ppm.

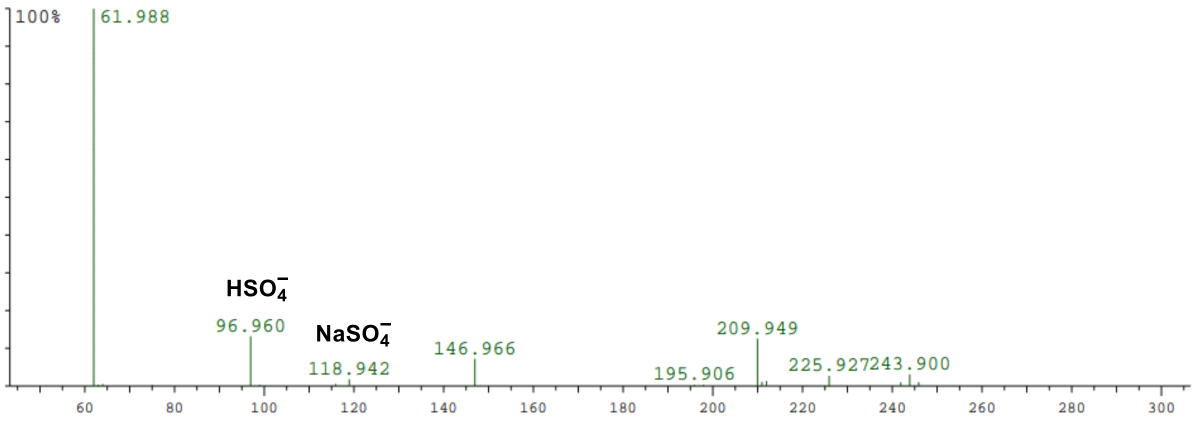

Fig S11. ESI-MS spectrum of H_2O solution after NO_2 formation with $Na_2S_2O_4$. **Negative ionization** mode.

GC-MS of vial headspace

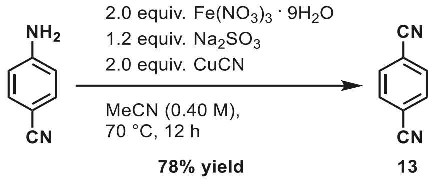

Caution: When performing reactions in pressurized systems (such as closed vials and pressure tubes), a blast shield must be used to minimize personal damage in case of an accident. See safety statement on page S6–S7.

Under an Argon atmosphere, to a 4-mL borosilicate vial equipped with a Teflon-coated magnetic stir bar were added 4-aminobenzonitrile (59.1 mg, 0.500 mmol, 1.00 equiv.), Na_2SO_3 (75.7 mg, 0.600 mmol, 2.00 equiv.), CuCN (89.6 mg, 1.00 mmol, 2.00 equiv.) and iron (III) nitrate nonahydrate (404 mg, 1.00 mmol, 2.00 equiv.). Then, acetonitrile (MeCN) (1.25 mL, c = 0.4 M) was added. The vessel was sealed with a septum cap and heated at 70°C for 12 h in an aluminum block on a heating plate. After cooling to 23 °C, a sample of the reaction headspace was taken using a Hamilton syringe penetrating the septum. The headspace was analyzed using GC-FID, and GC-MS.

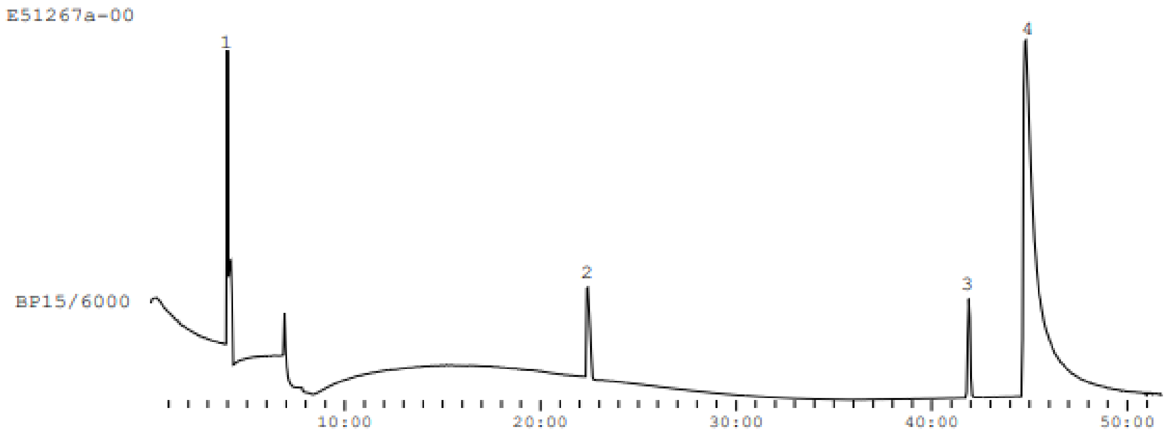

Fig S12. GC-FID chromatogram of the reaction headspace.

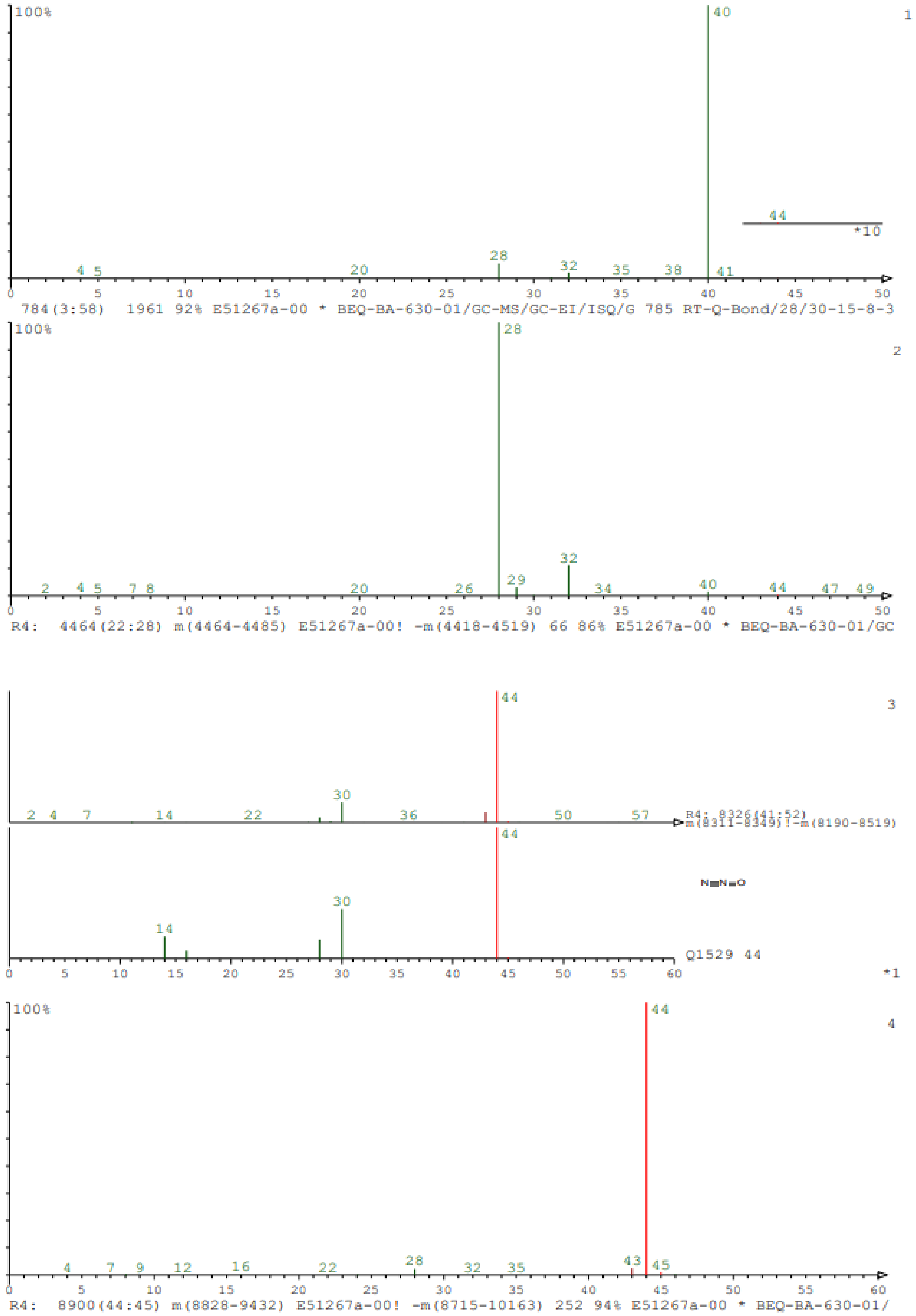

Fig S13. MS spectrum for signal 1, 2, 3, and 4 in Fig S12 showing Ar and N_2 and N_2O.

Reaction monitoring by ^{15}N NMR spectroscopy

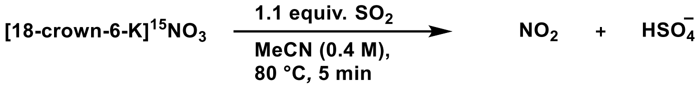

General procedure: Under ambient atmosphere, a J-Young NMR tube was charged with [18-crown-6-K]$^{15}NO_3$ (80.6 mg, 0.220 mmol, 1.00 equiv.) and MeCN-d_3 (0.5 mL, c = 0.40 M). Then, SO_2 (c = 4.0 M in MeCN, 84 µL, 0.250 mmol, 1.10 equiv.), was added via a Hamilton syringe. The J-Young tube was sealed, inverted several times for mixing, and inserted in the NMR spectrometer in a 80°C preheated probe. After 5 min, a ^{15}N NMR spectrum was recorded.

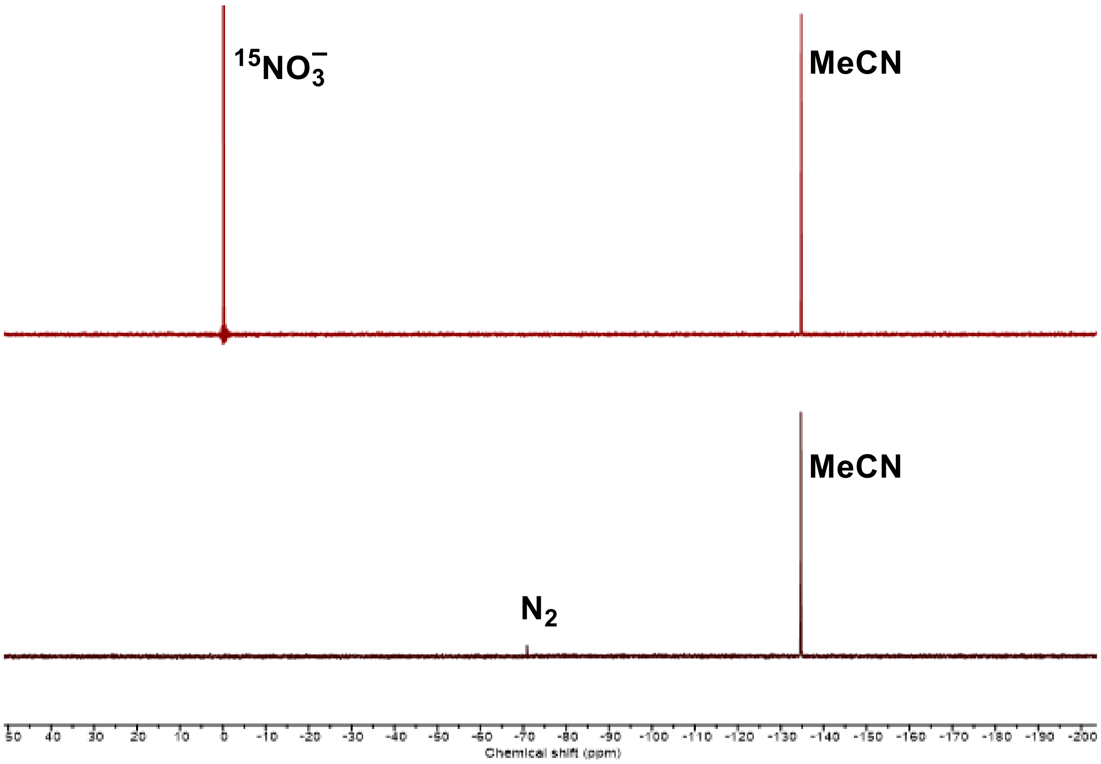

Fig S14. Stacked ^{15}N NMR spectra at 80 °C and 61 MHz in MeCN-d_3 of nitrate reduction reaction. Top: Reference experiment without SO_2. Bottom: Reaction with SO_2.

Mass spectrometric analysis after ^{15}N NMR spectroscopy

After the measurement of ^{15}N NMR spectroscopy, MeCN-d_3 was removed by rotary evaporation under reduced pressure. The residual solids were suspended in H_2O. The suspension was filtered and an ESI-MS measurement of the solution was carried out.

HRMS-ESI of bisulfate (m/z) calc'd for $H_1O_4S_1$ $[M]^-$, 96.9601; found, 96.9601; deviation: +0.2 ppm.

Reaction monitoring by ^{17}O NMR spectroscopy

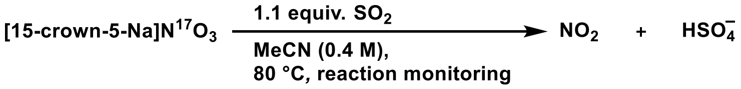

General procedure: Under ambient atmosphere, a J-Young NMR tube was charged with [15-crown-5-Na]$N^{17}O_3$ (67.0 mg, 0.220 mmol, 1.00 equiv.), and MeCN-d_3 (0.5 mL, c = 0.40 M). Then, SO_2 (c =

4.0 M in MeCN, 84 µL, 0.250 mmol, 1.10 equiv.), was added via a Hamilton syringe. The J-Young tube was sealed, inverted several times for mixing, and inserted in the NMR spectrometer in a 80°C preheated probe. The reaction was monitored by recording ^{17}O NMR spectra (every 5 minutes).

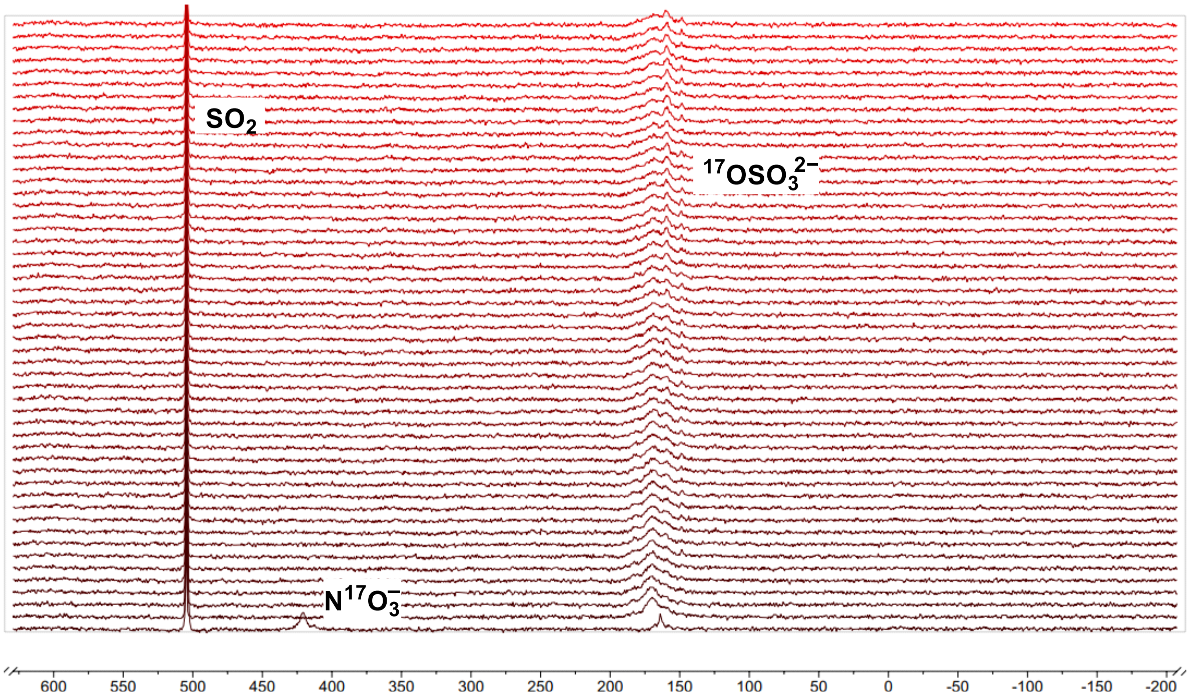

Fig S15. Stacked ^{17}O NMR spectra at 80 °C and 54 MHz in MeCN-*d*3 of nitrate reduction reaction. Bottom: t = 0; Top t = 4 hours.

Mass spectrometric analysis after ^{17}O NMR spectroscopy

After the measurement of ^{17}O NMR, MeCN-*d*3 was removed by rotary evaporation under reduced pressure. The residual solids were suspended in H_2O. The suspension was filtered and an ESI-MS measurement of the solution was carried out.

HRMS-ESI of sulfate(^{17}O) (m/z) calc'd for $H_1{}^{17}O_1O_3S_1$ $[M]^-$, 97.9643; found, 97.963; deviation: +0.3 ppm.

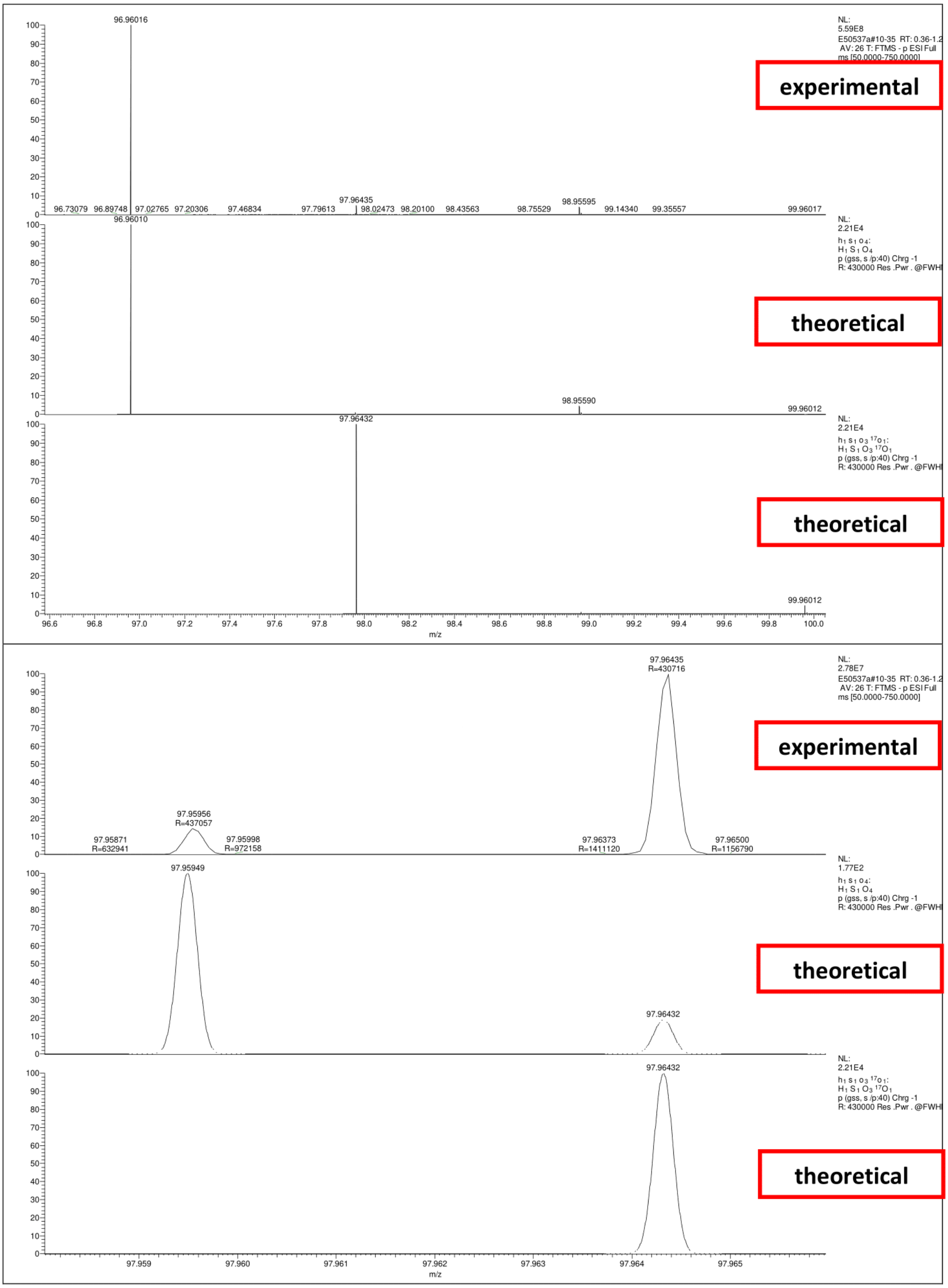

Fig S16. Comparison between theoretical and experimental mass spectrometric data for sulfate containing one ^{17}O atom.

COMPUTATIONAL DATA

Nitrate reduction with Na_2SO_3

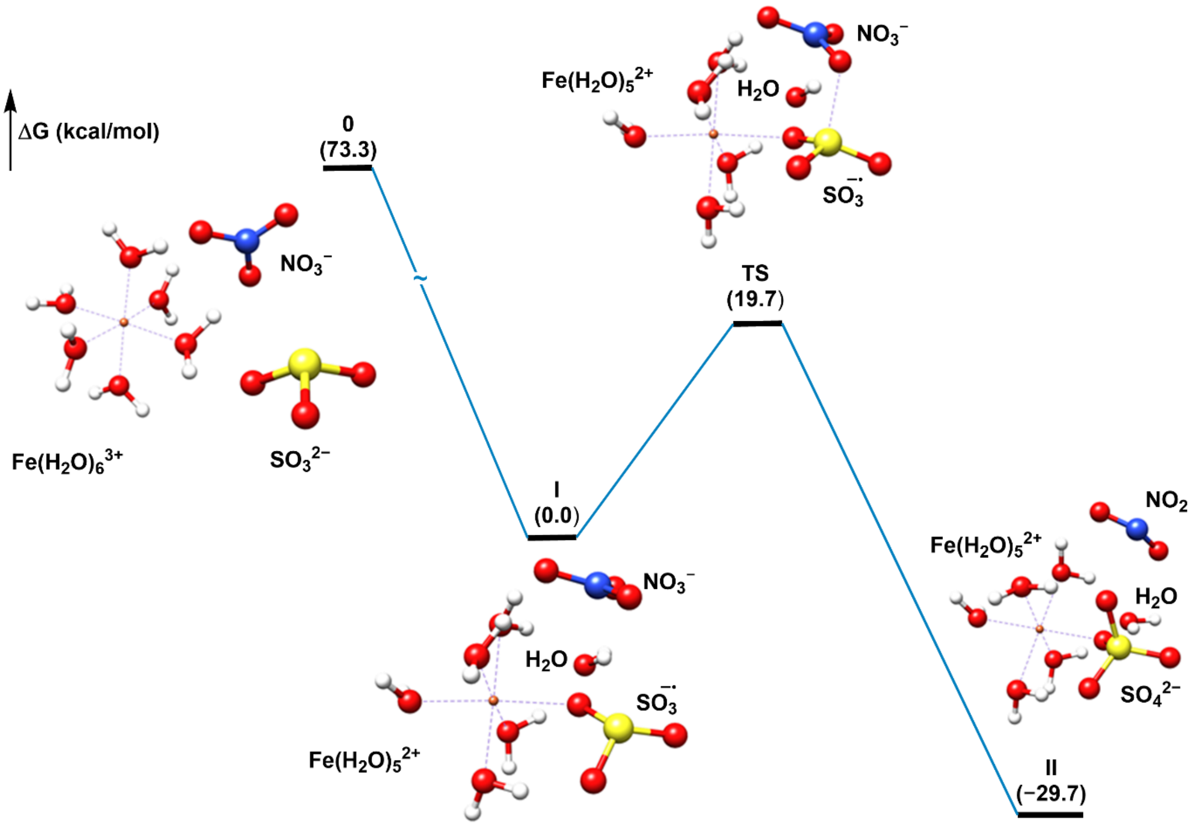

Fig S17. DFT calculations for the nitrate reduction of iron nitrate with sulfite at the M06-2X-D3(0)/ma-def2-TZVP and M06-2X-D3(0)/ma-def2-QZVP//M06-2X-D3(0)/ma-def2-TZVP levels using ORCA.

Table S7. The electronic energy Δ*E*, enthalpy Δ*H*, and free energy ΔG_{298} of the stationary points (in kcal/mol) on the lowest sextet energy surface for NO_2 release in the reaction of $Fe(H_2O)_6^{3+}$, NO_3^-, and SO_3^{2-} were computed at the M06-2X-D3(0)/ma-def2-TZVP and M06-2X-D3(0)/ma-def2-QZVP//M06-2X-D3(0)/ma-def2-TZVP levels, treating acetonitrile solvent molecules implicitly with the SMD model. All the energies are given with respect to those of **I**.

	M06-2X-D3(0)/ma-def2-TZVP			M06-2X-D3(0)/ma-def2-QZVP		
	ΔE	*ΔH*	*ΔG₂₉₈*	*ΔE*	*ΔH*	*ΔG₂₉₈*
**0**[a]	90.4	86.1	74.8	88.8	84.5	73.3
**I**	0.0	0.0	0.0	0.0	0.0	0.0
**TS**	19.1	17.7	19.7	19.0	17.7	19.7
**II**	−29.7	−29.7	−30.0	−29.4	−29.4	−29.7

[a] **0** corresponds to the isolated SO_3^{2-} and $^6Fe(H_2O)_6^{3+}$•••NO_3^-. Upon interaction between these two reactants, an electron is spontaneously transferred from the sulfur center to the iron center, resulting in the iron center adopting a quintet spin state.

Cartesian coordinates (Å) of all optimized structures given on Fig S12.

SO_3^{2-}
−2 1
S 8.46539951662324 6.09872123153172 3.39678673524255

O	7.66214096100637	6.31678263393287	4.67228562412722
O	9.64873898717371	7.05608990228699	3.45683223985123
O	9.03677810997873	4.68897839946090	3.47325620841863

$Fe(H_2O)_6^{3+}\cdots NO_3^-$

2 6

Fe	7.11878421343954	7.06244030040815	6.61884806060835
O	8.75955698771920	8.10799954354331	6.57789717988639
H	8.89994436174132	8.95099657297225	7.03936489549749
H	9.62804664685927	7.81031783534027	6.02199669591563
O	6.20727590698356	8.30882628764609	7.94609815486203
H	6.34811443952278	8.29127440997160	8.90942981634011
H	5.69757219817164	9.10523874510423	7.71374651392354
O	7.74181603095680	6.02284432846447	8.24822980384103
H	8.66204992946417	6.03575170518340	8.56794910991110
H	7.28949159417407	5.24638514850289	8.62422240074690
O	5.41588908006755	5.96243773930338	6.61929587719366
H	5.22113516260299	5.22402916678908	6.01388928795824
H	4.61819179946612	6.15987489700150	7.14259730216147
O	7.91689370856593	5.83016074229654	5.30965099632010
H	8.15119993919778	4.90446098812671	5.49107491884794
H	8.57918433262807	6.24821014148097	4.61317772235512
O	6.31515394832945	8.21982818685817	5.15654724573723
H	5.53629115585452	7.98477389333195	4.62101671055853
H	6.80647184842198	8.92676565220480	4.70089477685715
N	10.58478815369924	7.22761573906756	4.09348676044635
O	9.42837307634433	6.93363466807096	3.68148501741910
O	10.75131242566449	7.41927350396993	5.33519422522408
O	11.50642719910823	7.32414350079876	3.33354361853092

I

0 6

Fe	7.60818495575204	8.03400607660485	6.07681865816365
O	9.74613862483454	7.92405478508479	6.14520485493574
H	10.25778373754249	7.51838976375562	6.85754524428075
H	10.08319543517636	7.59630502460634	5.28667301280977
O	7.64057879989120	9.43631632430683	7.70555954261885
H	7.67700987737999	9.12147202441940	8.62058972584109
H	7.00702238752376	10.16816277462341	7.68596113580912
O	7.80167948087150	9.66842379015567	4.70688915520775
H	7.19471248075082	9.48079409605660	3.96206391852716
H	8.66071218458471	9.91691677453299	4.33862640446446
O	5.21607926590413	5.29755018712693	5.75111845862436
H	5.26327402625301	5.75569241608231	4.89404853561198
H	5.17177123690457	4.35359971566694	5.55026974655534
O	7.11987894163517	6.35841041620768	7.29548989631907
H	6.44915753479265	5.82937281155645	6.79301278925315
H	7.80794301500131	5.75165809385226	7.60053761572401
O	5.51223028232467	8.39090240778995	5.94130702235416
H	5.12461535893393	8.07474483575175	5.08180441517676
H	4.92507894253568	8.10179593811706	6.65312708144859
N	5.49432209166909	7.66981411528492	2.74816843693893
O	5.99989470020437	8.80570783032060	2.83148496323359
O	4.77005611895058	7.25671587744481	3.69563916871562
O	5.67822532466693	6.96703471536730	1.77067807273684
S	8.57077575987362	6.56549834013349	3.25565908807802
O	7.65922489360170	6.62781948704673	4.43597505254417
O	9.88418570105374	7.15772369983209	3.56139121090339
O	8.61423384138694	5.24618767827180	2.65032379312330

TS I

0 6

Fe	7.60373654818406	8.00134948212432	6.08874632045862
O	9.76327233838619	7.92905296383000	5.98989918705881
H	10.34449152791486	7.49542971496192	6.62950964345397
H	10.05214969905846	7.67434798784148	5.09598746676801
O	7.65117623386312	9.38911906712537	7.72177436915508
H	7.46875526576793	9.06963213609055	8.61759572441271
H	7.15387794476207	10.21297980591972	7.61034244678018
O	7.79499498103964	9.68087814830036	4.79028384584121
H	7.17729362786237	9.53278922632014	4.02835769559757
H	8.67166944736853	9.85572382818332	4.42146020765105
O	5.20060623680442	5.33390126112888	5.82940399398879
H	5.39607086931592	5.70732133154506	4.95180809491234
H	5.03085505232631	4.39291192128844	5.69295605019800
O	7.11401052928943	6.38633716457577	7.40852156376427
H	6.45042432876842	5.84031572310707	6.91982992501781
H	7.80190667735189	5.79013493720824	7.73424311472744
O	5.47535584394448	8.29646565339684	5.92857498762505
H	5.12790256371740	7.98280415584284	5.05716805160670
H	4.89406170149865	7.93546393204231	6.61194399736273
N	5.85516519023550	7.77256018922715	2.82343351158153
O	6.03058542597138	9.02211702080217	2.94351685927259
O	4.99510366834473	7.16556331298915	3.53554354392392
O	6.69327748236106	7.09256551740726	2.14354242324853
S	8.28456203201373	6.47492413534788	3.26541531310802
O	7.63190489419883	6.48262581824404	4.59824492298343
O	9.38723151026045	7.43014130596246	3.21185867596555
O	8.56112909996971	5.15196563360190	2.76607805183283

II

0 6

Fe	7.60465018216404	8.19626581317910	5.76928216800264
O	9.80715836289873	8.20518318214046	5.66896588339631
H	10.31350973852782	8.25755315802972	6.49034191745347
H	9.93614282132669	7.30269695256843	5.29274154640110
O	7.70118082089291	9.06367388163766	7.71876281107782
H	7.50514513204260	8.53932044450248	8.50852954304766
H	7.42245120219671	9.97508473069126	7.88766829769165
O	7.57931344069671	10.24275171898608	5.09235547658651
H	6.80322371991827	10.57357597262837	4.61892838796296
H	8.35735358480645	10.56229622701106	4.61258005033637
O	5.38054129410354	5.46063985860723	5.60747207791081
H	5.99463529044702	5.21729270549064	4.86017695615242
H	4.65702677410559	4.82330885417793	5.61850831950058
O	7.70478317906739	6.29028220803892	6.87209200803598
H	6.86605917913849	5.80940244409391	6.74991152045241
H	8.36407499640783	5.80893418094335	6.33257053348197
O	5.42909035355296	8.14005708291117	5.74290681391148
H	5.16742691405372	7.19322445881570	5.67941084725774
H	4.88752956043641	8.55336970816880	6.42816081573203
N	5.38040042796919	8.09082371702781	2.66672492876075
O	5.47214973396550	9.24818748077304	2.88141092790075
O	4.60974668768915	7.25275534340327	2.96509929037888
O	8.81151646454745	5.96121164061842	2.32010066207551
S	8.27712140436018	5.94409181444317	3.66617818210617
O	7.62299185679096	7.27985616331240	3.96421004200129
O	9.35108973322767	5.76118445259014	4.68412671787853
O	7.25299614466453	4.89818580520794	3.84600727450502

Nitrate reduction with $NaHSO_3$

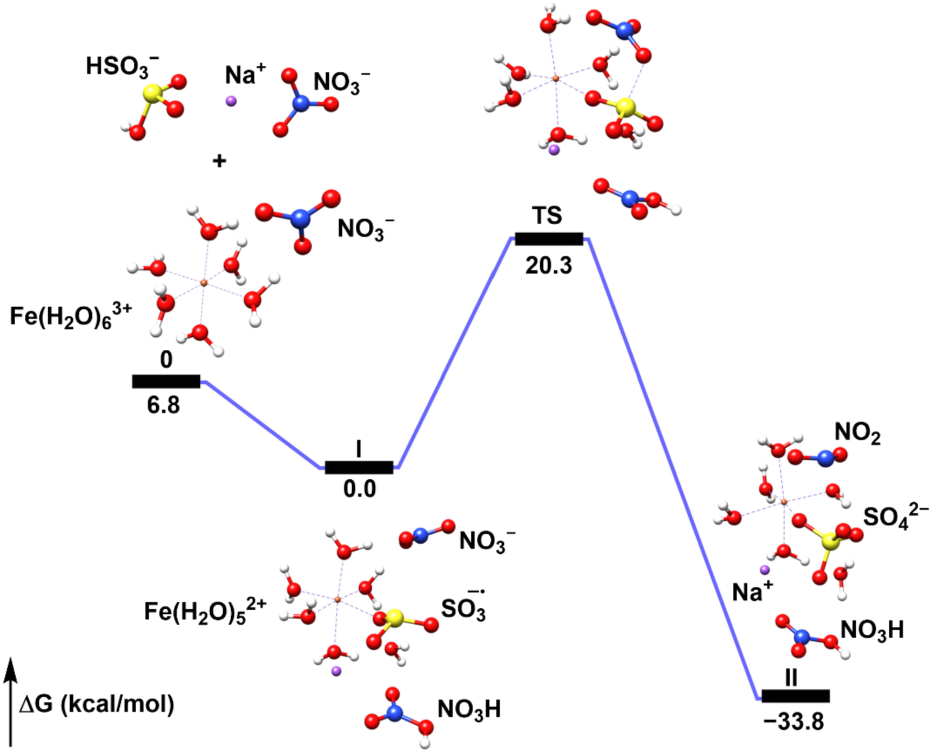

Fig S18. DFT calculations for the nitrate reduction of iron nitrate with $NaSO_3H$ at the M06-2X-D3(0)/ma-def2-QZVP//M06-2X-D3(0)/ma-def2-TZVP level using ORCA.

Table S8. The electronic energy ΔE, enthalpy ΔH, and free energy ΔG_{298} of the stationary points (in kcal/mol) on the lowest sextet energy surface for NO_2 release in the reaction of $Fe(H_2O)_6^{3+}$, $2NO_3^-$, and $NaSO_3H$ computed at the M06-2X-D3(0)/ma-def2-TZVP and M06-2X-D3(0)/ma-def2-QZVP//M06-2X-D3(0)/ma-def2-TZVP levels, treating acetonitrile solvent molecules implicitly with the SMD model. All the energies are given with respect to those of **I**.

	M06-2X-D3(0)/ma-def2-TZVP			M06-2X-D3(0)/ma-def2-QZVP		
	ΔE	ΔH	ΔG_{298}	ΔE	ΔH	ΔG_{298}
**0**[a]	29.0	24.1	10.4	25.4	20.6	6.8
**I**	0.0	0.0	0.0	0.0	0.0	0.0
**TS**	18.6	17.4	20.3	18.6	17.3	20.3
**II**	−34.5	−34.6	−33.7	−34.6	−34.7	−33.8

[a] **0** corresponds to the isolated $^6Fe(H_2O)_6^{3+}$•••NO_3^- and $NaSO_3H$•••NO_3^-. One NO_3^- was added to the starting material $NaSO_3H$ to show that hydrogen transfer does not occur before complex formation. Upon complex formation, $NaSO_3H$ spontaneously transfers a hydrogen to NO_3^- and an electron to the iron center. This spontaneous proton-coupled electron transfer results in a quintet spin state at the iron center and a spin-doublet SO_3^-. Subsequently, NO_3^- transfers an oxygen atom to the sulfur center with a free energy barrier of 20.3 kcal/mol, yielding SO_4^{2-} and NO_2.

Cartesian coordinates (Å) of all optimized structures given on Fig S12.

$Fe(H_2O)_6^{3+}\cdots NO_3^-$

2 6

Fe	7.11878421343954	7.06244030040815	6.61884806060835
O	8.75955698771920	8.10799954354331	6.57789717988639

H	8.89994436174132	8.95099657297225	7.03936489549749
H	9.62804664685927	7.81031783534027	6.02199669591563
O	6.20727590698356	8.30882628764609	7.94609815486203
H	6.34811443952278	8.29127440997160	8.90942981634011
H	5.69757219817164	9.10523874510423	7.71374651392354
O	7.74181603095680	6.02284432846447	8.24822980384103
H	8.66204992946417	6.03575170518340	8.56794910991110
H	7.28949159417407	5.24638514850289	8.62422240074690
O	5.41588908006755	5.96243773930338	6.61929587719366
H	5.22113516260299	5.22402916678908	6.01388928795824
H	4.61819179946612	6.15987489700150	7.14259730216147
O	7.91689370856593	5.83016074229654	5.30965099632010
H	8.15119993919778	4.90446098812671	5.49107491884794
H	8.57918433262807	6.24821014148097	4.61317772235512
O	6.31515394832945	8.21982818685817	5.15654724573723
H	5.53629115585452	7.98477389333195	4.62101671055853
H	6.80647184842198	8.92676565220480	4.70089477685715
N	10.58478815369924	7.22761573906756	4.09348676044635
O	9.42837307634433	6.93363466807096	3.68148501741910
O	10.75131242566449	7.41927350396993	5.33519422522408
O	11.50642719910823	7.32414350079876	3.33354361853092

$NaSO_3H{\bullet}{\bullet}{\bullet}NO_3^-$

−1 1

N	12.77894915548997	3.27332849641237	4.38114922491062
O	13.84196647703213	2.67179692014953	4.45255045316982
O	12.49776299416631	4.18435033306160	5.19522872219865
O	11.94022671679354	2.99647866773561	3.49141427647366
S	8.33197409597055	6.42706324264578	3.60194587928690
O	8.24625879820307	5.19801631572857	4.43442329635875
O	9.75122368883990	6.60911392628643	3.18529629272729
O	7.61786659519173	5.84290394208804	2.21514651695841
H	7.67898407666380	6.53067137914862	1.53378810224372
Na	10.47024540164862	4.65124377674327	4.17620423567205

I

1 6

Fe	7.71846181105411	7.93497199681207	5.96520660624925
O	9.90650595528657	8.20065980569911	6.24334988039615
H	10.13198823325357	8.64577451810962	7.07888224314336
H	10.32331525393945	8.72927633323239	5.54002653386665
O	7.49324613020077	8.99692224016290	7.80067206847152
H	6.98630543019415	8.63604192197071	8.54418944757515
H	7.36331261257925	9.95739494266168	7.81789373101384
O	7.70153241527346	9.59132103523893	4.69711764733355
H	7.30910948787714	9.41802992847773	3.78809377346577
H	7.57614893119990	10.52012556451768	4.93016723645160
O	6.38361123264265	4.80748608677143	5.45199527585977
H	6.81168688201269	4.82161125445758	4.58186857158730
H	5.93697126259322	3.95237879533427	5.53263965321806
O	8.16441119924565	6.04837509518618	7.02239249567683
H	7.55786817484910	5.39248822371063	6.60195563921106
H	8.08530693888916	5.94564322651326	7.98410761721980
O	5.69568212211973	7.54323635836173	5.63435562174181
H	5.41681227607430	7.93366198443060	4.76648675150753
H	5.54359404621087	6.58398517916145	5.57273163211829
N	5.61594533235310	8.62908058680190	2.25344357778451
O	6.82853687168956	8.94199335937176	2.36446994788521
O	4.91469530086814	8.52587646507788	3.28561359980869
O	5.13310449826221	8.43624864647375	1.15314320977179

N	10.43352341744803	3.54078263614403	4.39888985567811
O	10.45043963523610	2.61843963630453	3.42777292683765
O	11.48561786898366	3.91896730719332	4.84401421981838
O	9.34149519580320	3.91008827024870	4.73173012653342
S	9.18719192226273	6.70990554434312	3.19473978835269
O	8.19858926983748	6.65909584582133	4.32041809736894
O	10.54320571235420	6.51020742525292	3.73269955019807
O	8.81699190173407	5.85083084904470	2.08844238344445
H	11.39794921297640	2.42128441475049	3.25996905646798
Na	10.33003368792336	5.88747429994635	5.96999909647871

TS
1 6

Fe	7.61527635737440	8.09279281315195	5.98586977663719
O	9.71173657782419	8.78601320043491	6.23443772730711
H	10.03236304110127	8.95763820026714	7.13656894409905
H	9.91146972329686	9.58978629527744	5.72408476612432
O	7.14621744357146	8.73087987949263	7.95124792436396
H	6.41316299433239	8.33154523699682	8.44396370564757
H	7.20704444256917	9.65657913192480	8.23356170771822
O	7.06803465219162	9.93369601419962	5.16650132432479
H	7.19047706697534	9.91319691903287	4.16107317438178
H	6.19004742616378	10.29534329475559	5.35178435247580
O	6.86148691156285	4.67992764578748	5.40324283711004
H	7.22177602876305	4.62954176526525	4.49744917081375
H	6.56341170689455	3.79061468285518	5.63924500411206
O	8.53452535696776	6.27921210845850	6.82259967076926
H	8.05942939950055	5.50885406350951	6.43248668349432
H	8.56760509633894	6.14471141037579	7.78241585404266
O	5.75412850962155	7.23134673845529	5.43368087026469
H	5.64580596416604	7.49261166249821	4.49285704928433
H	5.83282896973328	6.25840686256733	5.44003053975688
N	7.00874758913369	8.50166890845451	2.36507756725468
O	7.56292715690002	9.59895599146198	2.68340342604737
O	5.89777703263873	8.15086911263394	2.84063267140910
O	7.71456437209942	7.63483429734383	1.72665912091842
N	10.06482632787981	3.57914833807297	4.23359231887407
O	9.08758906706109	2.64875602231586	4.27942130583353
O	10.88479180353312	3.49277411355951	3.37653077516584
O	9.98057252573729	4.40696864953779	5.10678602206127
S	8.69327848728014	6.44073072164672	2.97290681053676
O	8.41793169536014	7.11781931683698	4.26528478287285
O	10.12128873359467	6.44744547559976	2.71242557535585
O	8.04940552193298	5.13953631779314	2.88543859196767
H	9.24332118361731	2.06414057232137	3.50712871754186
Na	10.52934105750420	6.69331401469501	5.29108909396500

2
1 6

Fe	7.63109574024120	8.11700195643186	5.77614193212912
O	9.76954113579591	8.70169442349997	6.10189840849022
H	10.05241324119542	8.82407662163308	7.02327409791502
H	10.09930066746264	9.47597905982392	5.61683857829297
O	7.13154400901708	8.59250135579351	7.77980306956562
H	6.85331036347698	7.94590050207543	8.44597700681600
H	6.64423417537312	9.41308526869473	7.95020641888052
O	6.89221923926430	10.09861510848528	5.39949540153102
H	7.43110538027693	10.84125425328594	5.08909578198446
H	6.04140034675252	10.14947052847426	4.93869296418988
O	7.11258142301137	4.19937925210363	5.52397302054617

H	6.94947204276599	4.61935803679302	4.65125304949908
H	7.65744629380831	3.42174658397924	5.35106216313033
O	8.40837969124555	6.20822232215464	6.54967717303900
H	7.92801959093844	5.37732370642780	6.24865068442618
H	8.63695336021840	6.09199098671675	7.48384525106130
O	5.63428415476879	7.42808004767076	5.27590938545066
H	5.81245301351947	6.83292792912547	4.50599986167957
H	5.12649071462294	6.91709769300389	5.92110836418140
N	7.02751061633542	9.43230787776703	2.03196376252537
O	7.97318827605383	10.08298051706639	2.29340215072567
O	6.05749318503628	9.10790498094002	2.62158360770248
O	8.05081030610943	6.87821996735026	1.66226640012037
N	10.52639087188046	3.46165337620524	4.49325391715492
O	9.54689394629616	2.52456077510488	4.47083915797530
O	11.28775432770709	3.49099779442810	3.57891756563595
O	10.50631868195485	4.15662602658865	5.47349138698103
S	8.03216758573134	6.40926702536843	3.02817228434623
O	8.32070334386714	7.58649571248844	3.95549210835831
O	9.07481029994978	5.41315615853057	3.31872087633352
O	6.70211100350676	5.86538860104475	3.40898889013473
H	9.64533430984091	2.05587017595138	3.61494732448020
Na	10.21945866197350	6.49852237499116	4.99453295471637

Nitrate reduction with $Na_2S_2O_4$

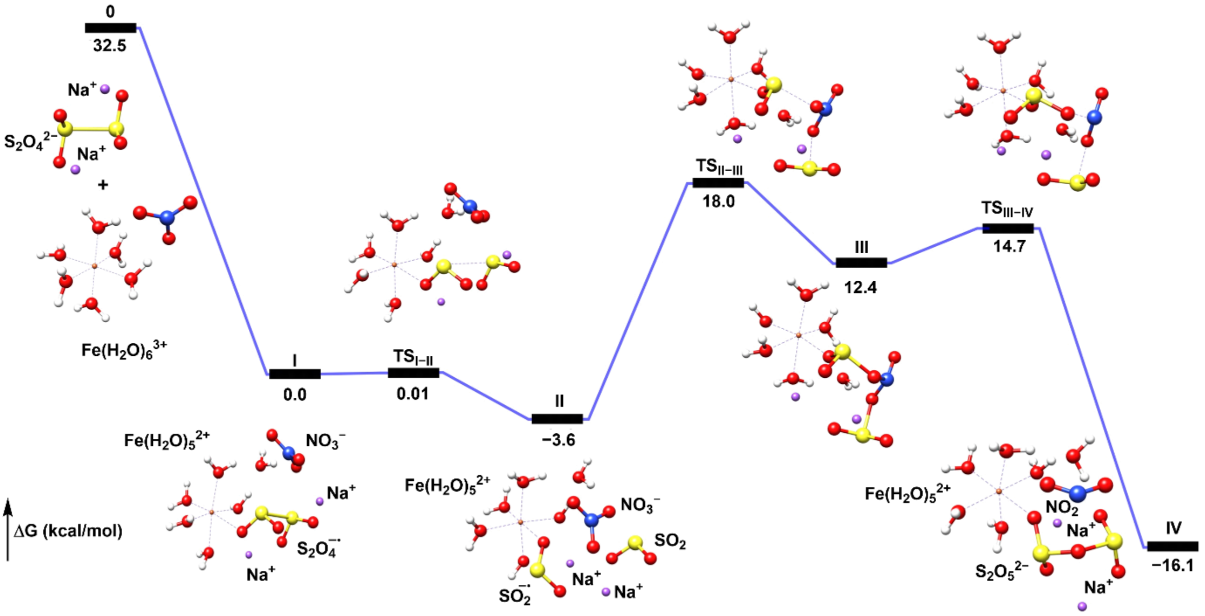

Fig S19. DFT calculations for the nitrate reduction of iron nitrate with $Na_2S_2O_4$ at the M06-2X-D3(0)/ma-def2-QZVP//M06-2X-D3(0)/def2-TZVP level using ORCA.

Table S9. The electronic energy Δ*E*, enthalpy Δ*H*, and free energy ΔG_{298} of the stationary points (in kcal/mol) on the lowest sextet energy surface for NO_2 release in the reaction of $Fe(H_2O)_6^{3+}$, NO_3^-, and $Na_2S_2O_4$ computed at the M06-2X-D3(0)/def2-TZVP and M06-2X-D3(0)/ma-def2-QZVP//M06-2X-D3(0)/ def2-TZVP levels, treating acetonitrile solvent molecules implicitly with the SMD model. All the energies are given with respect to those of **I**.

	M06-2X-D3(0)/def2-TZVP			M06-2X-D3(0)/ma-def2-QZVP		
	ΔE	*ΔH*	ΔG_{298}	*ΔE*	*ΔH*	ΔG_{298}
**0**[a]	49.5	45.6	35.2	46.8	42.9	32.5
**I**	0.0	0.0	0.0	0.0	0.0	0.0
$\mathbf{TS_{I\text{-}II}}$	0.7	0.1	-0.1	0.8	0.2	0.01
**II**	−4.9	−4.7	−4.7	−3.8	−3.6	−3.6
$\mathbf{TS_{II\text{-}III}}$	17.1	15.5	17.1	17.9	16.3	18.0
**III**	10.1	9.4	11.6	10.9	10.2	12.4
$\mathbf{TS_{III\text{-}IV}}$	13.3	11.7	13.8	14.2	12.6	14.7
**IV**	−17.4	−17.7	−17.2	−16.3	−16.6	−16.1

[a] **0** corresponds to the isolated $^6Fe(H_2O)_6^{3+}$•••NO_3^- and $Na_2S_2O_4$. Upon complex formation, $Na_2S_2O_4$ spontaneously transfers an electron to the iron center. This results in a quintet spin state at the iron center and a spin-doublet $S_2O_4^-$. Subsequently, the S-S bond is cleaved with almost no barrier. NO_3^- then binds to both S atoms with 21.6 kcal/mol barrier relative to the S–S bond cleaved intermediate **II**, which corresponds to the rate-limiting step. Finally, one S–O bond and one N–O bond are cleaved to release NO_2, with a 2.3 kcal/mol barrier relative to the NO_3^--bound intermediate **III**.

Cartesian coordinates (Å) of all optimized structures given on Fig S13.

$Fe(H_2O)_6^{3+}\cdots NO_3^-$

2 6

Fe	7.12073222933476	7.06216517780080	6.61976599470942
O	8.75958593758447	8.10947568544438	6.57860637784301

H	8.90189353038867	8.94957915562003	7.04428570758201
H	9.62830423579836	7.81084943300223	6.02142374690628
O	6.20461092475212	8.30803057112723	7.94561996479707
H	6.35149400186052	8.29670832488081	8.90793130084980
H	5.69850888763765	9.10539434159131	7.70958206659359
O	7.73805986143728	6.02351237728322	8.25153460169659
H	8.66014852210002	6.02935188561892	8.56579862183339
H	7.28327380818587	5.24810307855846	8.62629637960278
O	5.41105833427908	5.97159119365264	6.61136768930071
H	5.22908637627581	5.20761795467305	6.03496570992205
H	4.61829561927349	6.16168705401483	7.14437457283133
O	7.91875838565841	5.83191261444981	5.31215836356023
H	8.12945235484482	4.89814856967159	5.47873402510558
H	8.58610484501611	6.24764517797532	4.61428280811474
O	6.31750448946357	8.22441560567887	5.16059933279290
H	5.55985709606708	7.97035030650485	4.60393441191037
H	6.81435106597609	8.93078568335986	4.71029061078927
N	10.58058797929722	7.23192988008838	4.09210990085618
O	9.42572605605907	6.92549478184599	3.68255674596294
O	10.74886210245085	7.42198956691203	5.33394124004387
O	11.49770749524158	7.34054527668225	3.32947691753828

$Na_2S_2O_4$

0 1

S	7.53823185654091	6.77562155736574	3.68391209824017
O	8.14416102600924	7.05112054021268	5.03275781881678
O	8.65893504929669	6.80164194715518	2.65795874516735
S	7.42946870441709	4.48900277063732	3.78824879084226
O	6.97784291988591	4.19842414508152	2.38393242840028
O	8.93612682315169	4.29796437805896	3.83903574806406
Na	8.96146987833540	4.87126607357289	1.48139997619189
Na	10.14903201972555	6.12423449614357	4.46410077757342

I

2 6

Fe	7.73832175133268	8.29717791140036	6.25924797195216
O	9.69442526663493	7.75398042036903	7.16607042157590
H	9.74501899690980	7.76409538562302	8.13833170196014
H	10.42984216937341	8.31516219209678	6.85925193562446
O	7.05415020426509	8.85148719428766	8.17038509590644
H	6.12163529392985	8.76400812355836	8.42389880590408
H	7.42641995989185	9.57543292050577	8.69772623699962
O	8.46257371636882	10.17613215587197	5.67805408769472
H	8.43386937972708	10.53142606512752	4.77663763443021
H	8.75691173593456	10.88835834183229	6.26554380083846
O	5.15467704468758	6.03391280570534	4.89347848900404
H	5.15159917609513	5.86128507496633	3.92252523789836
H	4.34097006658295	5.64486738720839	5.24713925023496
O	7.25321289803203	6.20177736425271	6.66683507801704
H	6.50280586896372	5.92164116579601	6.09368430275519
H	7.03104268811114	5.92451377400171	7.57194502209279
O	5.87787494437018	8.59141086740027	5.36273318649088
H	5.36602974839781	9.40834812642363	5.28059830661782
H	5.32734505722733	7.82132752401704	5.10943327053194
N	5.15824769666888	6.78217654915294	1.61377929353301
O	5.67004770129210	7.04050254651034	0.51354999069537
O	4.35788938138444	7.52066992160393	2.15361154109133
O	5.51748969820344	5.70239172368553	2.18908050285361
S	7.83245621735733	6.97048081188120	3.38423732830751
O	8.70799558854037	7.36528171922967	4.54184959523322

O	8.57535570861216	6.83436362184467	2.13197665904130
S	7.76473215259183	4.24842162416691	3.68891484646533
O	8.04167128835269	3.94640003234422	2.30124468174568
O	8.93459570620910	4.20612182628930	4.55113983780878
Na	7.29515750353155	5.38788190628917	0.62974403872308
Na	9.64231439041982	5.64893891655753	6.11237884797235

$TS_{I\text{-}II}$
2 6

Fe	0.54736245273130	1.08880098691934	1.26553231424475
O	2.50974818007158	0.54738538661626	2.18339205742470
H	2.54981887880312	0.47933257861565	3.15320020675725
H	3.23879490274069	1.14093711900218	1.92926544910812
O	-0.14765970557116	1.58803593248643	3.19078456779295
H	-1.08392953354169	1.50043021863657	3.42939379353125
H	0.21879510837915	2.30509116960456	3.73119405671886
O	1.20673952800092	3.01753468868248	0.74289309889183
H	1.17541223885987	3.39038632251154	-0.15094974937240
H	1.50944102885211	3.71559861773920	1.34225503384952
O	-2.04727694668084	-1.21908378751282	-0.08571794944137
H	-2.05104918656704	-1.37659630446126	-1.05832290658416
H	-2.86436011339076	-1.60526514920592	0.26305210624672
O	0.09472329363555	-1.03168458829239	1.63360982004676
H	-0.66760324946525	-1.30524194718556	1.07405339110673
H	-0.09494486401158	-1.34038749695385	2.53548105852475
O	-1.33904174024825	1.34382244256366	0.39885131408995
H	-1.85314047004857	2.15348592543041	0.27427312799481
H	-1.87031299789450	0.56365822465785	0.13581040269183
N	-2.01098163369528	-0.40832791652090	-3.32561176071460
O	-1.44552547056195	-0.08747560564172	-4.38370883751906
O	-2.82756424925155	0.30760504082336	-2.77848049404171
O	-1.69061854916716	-1.52422778428003	-2.80328344771383
S	0.67401535984851	-0.03815699259407	-1.66361962034711
O	1.53016345362117	0.23494412922713	-0.42924042029391
O	1.49416183631559	-0.59046663029616	-2.76567835384570
S	0.40262578848630	-3.16112990833330	-1.30192574571618
O	0.65246781120986	-3.43337562106611	-2.68496349956339
O	1.56926625343391	-3.04786658839128	-0.46934996250410
Na	0.16991283502124	-1.75129309652033	-4.30945706700078
Na	2.45055976008469	-1.45646936626089	0.92726801563756

II
2 6

Fe	7.51344621125708	8.39031694306242	6.09753788337386
O	9.63425380960457	7.68268956364171	6.42364496293636
H	9.83073361335774	7.41605155376994	7.33835962337715
H	10.38907948923449	8.22929330875133	6.14560709451862
O	7.40628954611700	8.72226601236259	8.18245096315998
H	6.53823361190152	8.66224031690272	8.61297350243832
H	7.85859613378352	9.48661384365706	8.57324555060645
O	8.08092583534850	10.40596248405308	5.73880557459154
H	7.50277624738917	11.10160104798785	5.39241009861662
H	8.97750571429011	10.77045600730043	5.77834847013719
O	4.70234650328534	6.20280940960010	5.37467921110108
H	4.78532790400016	5.90976508374721	4.44597513108565
H	3.91097051981187	5.78325008339928	5.74144362783176
O	7.14040383008886	6.24309571280296	6.51471401640747
H	6.23995637928234	6.00994521234741	6.18726503311948
H	7.20407005136684	5.91799500188277	7.42703745929583
O	5.44485858128699	8.76658522041595	5.86298293366814

H	5.07980267974454	9.53878750004716	5.40896612255209
H	4.94003439996076	7.97656297198148	5.58023099664178
N	5.94458978179577	6.43924680494470	2.25010610220670
O	6.94343872381237	6.38391038756972	1.53443127863948
O	5.27312585309603	7.43157613255166	2.37752269155530
O	5.59066858958546	5.37521778512415	2.89321149181335
S	8.85874604549907	8.10580900503194	3.13521498103638
O	7.82058574247014	7.69545520928427	4.19001683091132
O	9.68251002687724	6.88413826379160	2.85055402197318
S	6.92628859143074	3.63487942552684	2.90848517782890
O	7.17383714163615	3.44359981034113	1.50662077611280
O	8.03775037821929	4.30029429522228	3.56431557350864
Na	8.93701916029254	5.30263281625869	1.37999088711332
Na	8.99850790416713	5.76693278663280	4.97787993183671

TS_{II-III}

2 6

Fe	7.46205380924344	8.35382649566054	6.18335705715403
O	9.57870005877809	7.63905368015557	6.38953466455549
H	9.84402171307224	7.41642873104344	7.29894059254928
H	10.30094578558818	8.18709752858769	6.03607860399239
O	7.48783915108346	8.82466396398851	8.23885021850610
H	6.65449440148879	8.84297735400711	8.73565579476061
H	8.06180501942591	9.50763355597882	8.61926390119973
O	8.05907336233045	10.24756344924824	5.45248600668310
H	7.56434502770263	10.78119714782440	4.81284952440760
H	8.73815901686000	10.81846458386347	5.84203951904896
O	4.72451959920449	6.24637945245738	5.36745620363744
H	4.92597464581140	5.99876157707047	4.44574212009621
H	3.90798704905035	5.78891751671538	5.61549970056817
O	7.11266349384274	6.22784927862796	6.63107256585985
H	6.21822531335431	5.99833212683450	6.28794229747630
H	7.16583977085026	5.89886923398403	7.54284221736988
O	5.40544008934582	8.72555095402679	6.13415387527969
H	4.95993261998020	9.56578602051575	5.95862933755102
H	4.87752388402887	7.98856662687311	5.76457745583197
N	6.11032570680473	6.45340334687347	2.04890676617822
O	7.32272452721111	6.67128527610255	1.71592750947978
O	5.19727561391955	7.23990839666015	1.83393878397986
O	5.89425008199948	5.48399617318464	2.97411612324028
S	8.45799979030823	7.84670965942986	3.01582629800916
O	7.63548100705684	7.56123963397895	4.24208658068599
O	9.60302657279612	6.91439479035421	2.97801241603130
S	6.83815476067991	3.88616976445904	2.79461358270787
O	7.10503779030242	3.85715859489371	1.36188351522481
O	8.07313634009101	4.25878952802518	3.51210047802741
Na	9.14603555378823	5.08441811778357	1.51751166063944
Na	8.93368744399452	5.66458744078490	5.04313262926368

III

2 6

Fe	7.43830542880250	8.31336272802546	6.17786738781375
O	9.55856870337544	7.61146202645402	6.35888424770455
H	9.84999653238442	7.42935839916083	7.26942090435515
H	10.26906575232086	8.14338461493944	5.96000498447241
O	7.50836410345195	8.87577497278767	8.20940586875447
H	6.68699722601940	8.91778310397884	8.72404553304041
H	8.09271532173741	9.57208353930661	8.54744973577414
O	8.02807654015039	10.17940277966887	5.35988194191042
H	7.51380648624466	10.69128589577212	4.71765888154703

H	8.69837119547120	10.77278217239006	5.73084403469382
O	4.75228917313251	6.20642039277091	5.37500114418646
H	4.99207662752131	6.01402979531174	4.44845485760595
H	3.93731938360367	5.72027412712328	5.56654805733371
O	7.10318509316458	6.20493319535807	6.68748069119131
H	6.21231665393269	5.96645647490118	6.34073352636487
H	7.15247817098054	5.89614484266352	7.60630512808973
O	5.38201997422636	8.69253209911088	6.15327751213813
H	4.94065868087868	9.52999669009726	5.95512258306250
H	4.85803126685329	7.94922682590265	5.79059138047698
N	6.12585739395838	6.52166454092911	2.02622942020110
O	7.49109331102070	6.72158654670354	1.86982027828417
O	5.40404040057577	7.50851691386166	2.09536251267723
O	5.86320035477477	5.50882714223633	2.92208944533352
S	8.32807022693869	7.75984413103756	3.01382363792341
O	7.56491295008190	7.44456994491397	4.26492049816467
O	9.57383712196063	6.97700762536739	3.01777368066382
S	6.79786286542177	3.94077503626646	2.73899163112587
O	7.11982486684242	3.94164143753751	1.31440174211943
O	8.01692512661844	4.27951764239104	3.50547708336935
Na	9.20909377811741	5.07367174066961	1.56938504317784
Na	8.89731828943278	5.61566262235770	5.07777462644064

TS$_{III-IV}$

2 6

Fe	7.42048015494101	8.30464241974160	6.18577778262441
O	9.54299162098922	7.57485372070001	6.30368475195377
H	9.89722570624687	7.51728027418554	7.20754563281088
H	10.23267959057296	8.01017613017476	5.77397976151737
O	7.62126151705562	8.92021913843354	8.19736064481553
H	6.87592844121630	8.82829093106034	8.81085710076879
H	8.09980165278998	9.72437996026078	8.45165457009794
O	7.97952745045681	10.18328962641896	5.36967617316569
H	7.52585293514863	10.63425855918433	4.64226947460432
H	8.69831630675274	10.76054700659896	5.66679392823233
O	4.78156693545901	6.22002292569979	5.35421878131316
H	5.07410107889925	6.05805748872725	4.43576933959892
H	3.96461302614089	5.71729798303059	5.48392335970671
O	7.05735806320222	6.21637109687730	6.77033586627917
H	6.17220605404383	5.98581984918627	6.40404861758176
H	7.09042308463039	5.91145943730198	7.69051620160495
O	5.35869338918644	8.69514692214245	6.23638023122540
H	4.92576188958118	9.53914382398157	6.04819378465225
H	4.84659537758507	7.96736427379572	5.82990538237741
N	5.99676390765708	6.51705825712207	1.98144880354690
O	7.59247562900301	6.78777566577416	1.93855511811823
O	5.33521757240778	7.51545975915358	1.99502721454256
O	5.76348495883097	5.55049101622809	2.82419310483311
S	8.29706945762271	7.70993576392791	3.09996234448289
O	7.42644932227311	7.40511006088151	4.30581147093440
O	9.55017023765443	6.91883506846775	3.27089974995750
S	6.87232907127644	3.83482837088282	2.59207057877713
O	7.32674436847213	3.98410723496632	1.22676280795764
O	7.93034311550642	4.17177221172899	3.53716564910024
Na	9.32783682667059	5.23772625191699	1.62951160499570
Na	8.78241025772075	5.57825877144171	5.13072816781860

IV

2 6

Fe	7.47624692463574	8.28032928135715	6.08532857921598

O	9.59591133705716	7.50397907788390	6.21981892761880
H	10.02833263968474	7.57913467537573	7.08621958016511
H	10.25196571126472	7.78900306079228	5.56236145010710
O	7.66495769428210	8.99558073965040	8.05560878654361
H	7.06770715097959	8.70832439457574	8.76410677167270
H	7.83751478303191	9.93949519782944	8.19891209425107
O	7.99296527431908	10.22645192483142	5.38369759279981
H	7.49498105242453	10.67480414544950	4.68405138134500
H	8.89753052939098	10.57245576467101	5.35549945648943
O	5.04260084599333	5.91200763203181	5.24142077993920
H	5.61404121731441	5.48821034295808	4.57067988435138
H	4.22729368719703	5.39251884132942	5.28413992787054
O	7.06832986981172	6.25759481088184	6.89558285117195
H	6.18800112659959	5.96600767994706	6.56378599516214
H	7.13801725680205	6.02065567055662	7.83327391775512
O	5.36717033308039	8.57491557628267	5.98189611939617
H	4.94914908552122	9.37190782437541	5.62723448202615
H	4.98479999997704	7.80475423316981	5.51944837149563
N	4.67963903201271	7.30610084284400	2.20923008805738
O	7.42251477736756	6.35277902604507	2.06856978495461
O	4.80864008917785	8.19621595432301	2.97082138401840
O	4.33315571753422	6.18209887001503	2.29433140363556
S	8.29594118199167	7.32016367124713	2.99921535875166
O	7.48307820176700	7.35277131097246	4.28529571491721
O	9.51802162047247	6.52009560178808	3.34336181716241
S	7.21075519605236	4.59176662013921	2.33497036779251
O	8.51831022535827	4.07196947018007	1.91656175567357
O	7.07662995181920	4.53307978394782	3.80992691802539
Na	10.50379773933189	5.07508158200919	1.96728023070167
Na	8.62867774774569	5.41972839253772	5.28239722693133

Cyanation from aryldiazonium salt

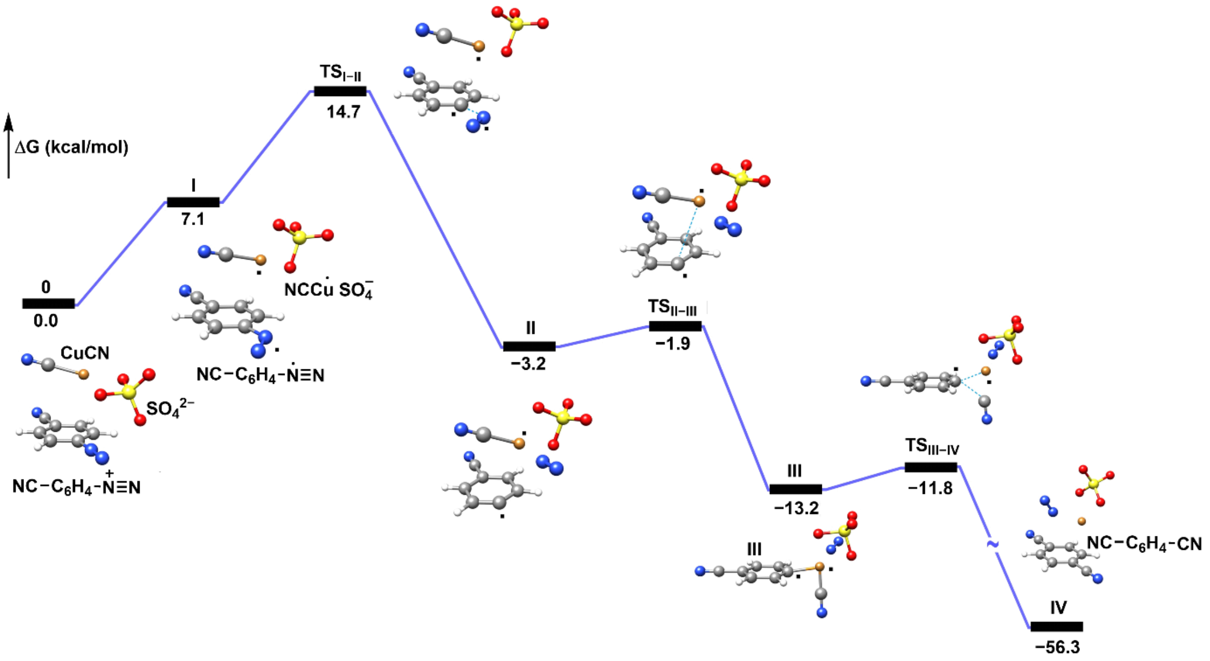

Fig S20. DFT calculations for the cyanation path of the diazonium salt through CuCN and SO_4^{2-} at the M06-2X-D3(0)/ma-def2-QZVP//M06-2X-D3(0)/ma-def2-TZVP level using ORCA.

Table S10. The electronic energy Δ*E*, enthalpy Δ*H*, and free energy ΔG_{298} of the stationary points (in kcal/mol) for the cyanation path of the diazonium salt through CuCN and SO_4^{2-} computed at the M06-2X-D3(0)/ma-def2-TZVP and M06-2X-D3(0)/ma-def2-QZVP//M06-2X-D3(0)/ma-def2-TZVP levels, treating acetonitrile solvent molecules implicitly with the SMD model. All the energies are given with respect to those of **0**.

	M06-2X-D3(0)/ma-def2-TZVP			M06-2X-D3(0)/ma-def2-QZVP		
	ΔE	ΔH	ΔG_{298}	ΔE	ΔH	ΔG_{298}
**0** [a]	0.0	0.0	0.0	0.0	0.0	0.0
**I**	9.8	9.1	9.4	7.5	6.9	7.1
$\mathbf{TS_{I-II}}$	19.5	17.2	17.0	17.2	14.9	14.7
**II**	2.1	0.8	−1.2	0.1	−1.3	−3.2
$\mathbf{TS_{II-III}}$	2.8	0.9	0.2	0.8	−1.2	−1.9
**III**	−9.3	−10.3	−11.7	−10.8	−11.8	−13.2
$\mathbf{TS_{III-IV}}$	−8.7	−10.2	−10.3	−10.2	−11.7	−11.8
**IV**	−54.7	−54.6	−55.7	−55.2	−55.1	−56.3

[a] The ground-state reactant complex (**0**) is a closed-shell singlet. Its open-shell singlet structure (**I**) is energetically accessible, and the reaction proceeds along the open-shell surface from **I** to $\mathbf{TS_{III-IV}}$. Product complex **IV** reverts to a closed-shell structure.

Cartesian coordinates (Å) of all stationary points given on Fig S14

0

−1 1

C	-1.39814743102819	0.40209180534876	0.81077941832729
C	-0.50241653738809	1.13562970498104	1.58957771065791
C	0.82031531002265	1.21766033766274	1.20387962375357

C	1.17566056913117	0.55307486515604	0.03702468171885
C	0.31416372126367	-0.18111132803513	-0.76703934313706
C	-1.00454056355991	-0.25202072467341	-0.35695413121884
C	-2.77826593388367	0.34904143117288	1.20779710785467
N	2.51734781276090	0.63785135039490	-0.35441447178715
N	3.56518211009808	0.69519080293189	-0.63331236372406
H	-0.84138547850306	1.64201782256791	2.48231274494028
H	1.55137223159192	1.77483150876191	1.77268498341262
H	0.67293936670545	-0.64174596825548	-1.67669357871231
H	-1.72604337009964	-0.80301303517020	-0.94371017200099
N	-3.87998332452235	0.31081040001245	1.52305618596358
S	1.69136514305705	1.71971677840854	-3.36256901509924
O	2.28748044877567	2.35668522141154	-4.53201052514772
O	1.84395922928156	2.56240269457406	-2.13816887722363
O	0.19462975108681	1.58493423721406	-3.55705170988837
O	2.26106659949397	0.38476725342665	-3.11638753214033
Cu	-0.53291397150729	2.60795581330905	-1.99418965405826
C	-1.65703392662178	3.48828046973444	-0.66398413073982
N	-2.31681375615505	3.99251355906500	0.14266204824928

I

−1 1

C	-1.16048313711094	0.41292122986289	0.93732024406967
C	-0.26705297764563	1.31364523435078	1.48652989288304
C	1.02809840012342	1.35855930661376	0.99121989178444
C	1.40099603374819	0.50419415365104	-0.04171452638028
C	0.49187807619113	-0.40876366046848	-0.59099367135887
C	-0.80455580401820	-0.45614955394495	-0.08937504488703
C	2.74145972221388	0.55540442635488	-0.55310932029826
N	-2.53200824723862	0.31318478152911	1.42343551798428
N	3.81212055877997	0.59819849082382	-0.96207228560141
H	-0.57456595141079	1.96442903789221	2.29473519071668
H	1.74788912078823	2.04973820913833	1.40859260812105
H	0.78821201961349	-1.04369948967160	-1.41657720638813
H	-1.54514518601218	-1.12415244808214	-0.51289038013368
N	-3.03222067187811	1.05979349508055	2.16251153982852
S	0.14612455920275	-4.81897120045433	-0.07957606305870
O	-0.22992985924993	-3.41972690961315	-0.59240181634359
O	0.71543749833125	-4.42995926142047	1.27940438065885
O	-1.03804570359744	-5.63443513594562	0.06732744680510
O	1.16501837553720	-5.40316811417872	-0.92214561619906
Cu	0.39363645480266	-2.48500531046641	0.97773018981598
C	1.03161115059423	-1.61272003509678	2.64250226581845
N	1.41136156823546	-1.14159024595471	3.62367076216291

TS_{I-II}

−1 1

C	-1.09334284593519	0.40634668930746	0.88564558694156
C	-0.25033146894682	1.31234858477442	1.46502157469903
C	1.05366026635693	1.36598973960268	0.97731932111974
C	1.43649135774530	0.51451880331688	-0.05587023589226
C	0.53854944918948	-0.40063948831470	-0.62375930002905
C	-0.76959835883801	-0.45845699866542	-0.13745302857597
C	2.78161506689344	0.57357211306790	-0.55359964732834
N	-2.84358802168427	0.28220749160434	1.52044259093726
N	3.85653652876992	0.62283247821012	-0.95113258809863
H	-0.57449106313164	1.95850775953884	2.27306483711535
H	1.76940372796490	2.05896032681382	1.40033365538596
H	0.85090757377985	-1.02784271871325	-1.45023186272144
H	-1.50577800045402	-1.12286220503897	-0.57582578515374

N	-3.15101067468851	0.95076260006324	2.35718401237075
S	0.11125793038459	-4.80957739499918	-0.05747791084654
O	-0.18622496186066	-3.41208930134156	-0.62222333438978
O	0.64343537049592	-4.40644332450998	1.31209927504283
O	-1.11079792072165	-5.57232828643626	0.06400428394493
O	1.13665558102471	-5.45963036625811	-0.84218622827116
Cu	0.40963979294885	-2.45751057457733	0.95075438403616
C	1.05720756083559	-1.59046930265105	2.61682967977806
N	1.44415910987130	-1.13196862479384	3.60124271993527

II

−1 1

C	-1.65447315380538	0.45669331331976	0.63882300707317
C	-1.16400974281759	0.85430364559266	1.87954655619672
C	0.19436865465378	0.70827242659841	2.16448634983596
C	0.97531517795353	0.16911108505015	1.18227793852535
C	0.54801301918835	-0.24317464212484	-0.06166476968457
C	-0.81432198362299	-0.09256985318168	-0.34003611463973
C	-3.05172766663099	0.61289669726589	0.35192208895252
N	4.14368804716121	1.65480674699218	-1.15913232096416
N	3.06049716676582	1.71477727657253	-1.14715702523826
H	-1.83440417054173	1.28101714830532	2.61442480248856
H	0.59330078751638	1.01790156258743	3.12199402744544
H	1.21256917511593	-0.70540659068972	-0.78239977941356
H	-1.22416468057693	-0.42686923416907	-1.28571466111412
N	-4.16950485529063	0.73713538034708	0.12477595099557
S	1.02287236532424	1.72130001191405	-4.01572706688902
O	0.06226299416040	1.68467089102452	-5.09531523419908
O	0.82765937322107	2.95665272757540	-3.14783527164715
O	0.70183431773432	0.63614859504475	-2.97777884024783
O	2.39955111024261	1.60567852267017	-4.44603166740740
Cu	0.42676034397561	1.87313230950124	-1.51012083629048
C	0.09310625722810	3.24595025498564	-0.10781565940806
N	-0.09125453695519	4.07513672481759	0.67176752563050

TS_{II-III}

−1 1

C	-1.77156954023067	0.42628156437941	0.84739216898393
C	-0.95652661089310	0.84794943049549	1.90014536978755
C	0.42247854062467	0.68038300025036	1.81718425313545
C	0.89860681940238	0.08182082024266	0.68138284712712
C	0.14366505190925	-0.36532457981464	-0.38161084694571
C	-1.24020889420563	-0.17529044642640	-0.29399421043576
C	-3.19006653531885	0.62452337854469	0.93816417404955
N	4.09125489621264	2.02444985529403	-0.72763064479857
N	3.01889452997133	1.97180170366059	-0.88401027583098
H	-1.39998218820941	1.31513417859509	2.77011607138900
H	1.07128275558648	1.01317111833545	2.61708875972004
H	0.57878432261907	-0.88915631811931	-1.22342171620305
H	-1.89100097826272	-0.50862466892988	-1.09245095165285
N	-4.32393839105736	0.78465360536149	1.01122039971405
S	1.49403689969505	1.75437497049515	-4.00218706872362
O	0.75250670641019	1.61407789446333	-5.23512451072342
O	1.00534305481270	2.95093029407032	-3.19859108649875
O	1.12049754573978	0.63161818996732	-3.02293489046155
O	2.92934862769787	1.80270108053783	-4.17866780366550
Cu	0.45607836688331	1.81582309534513	-1.63927103462086
C	-0.27241454950072	3.15647653454785	-0.36327432948512
N	-0.67913242988633	3.97979029870334	0.33376432613952

III
−1 1

C	-1.82784121393098	-0.11684378136575	1.29917119245926
C	-0.68225907245043	0.56589316234695	1.70688895211879
C	0.02733100561741	1.32386464164351	0.78481248616110
C	-0.43555777517456	1.33864777603538	-0.51351493138290
C	-1.57828561052157	0.70006408720248	-0.94845739079552
C	-2.28086211360272	-0.05461162052105	-0.01849785801196
C	-2.56170061330551	-0.89109860843137	2.25908378198487
N	4.12908883225391	3.08012510904918	-0.31616661733230
N	3.09083936355409	2.85385389834717	-0.53582314136263
H	-0.35125970275231	0.51127713249323	2.73549764575619
H	0.91143651837467	1.86948684913273	1.08726953013092
H	-1.92042753396197	0.76857315352764	-1.97282885821893
H	-3.17669606332155	-0.58497189579504	-0.31337799291882
N	-3.14781294753429	-1.50928790637482	3.02820612511207
S	2.54860365459496	1.07436401228620	-3.44595807290839
O	2.25763062523278	0.42781834881606	-4.70476810892746
O	1.95876031407722	2.48063962213594	-3.40563812754342
O	1.73284412625381	0.43041865211147	-2.31338072121065
O	3.95200805647579	1.09475712212389	-3.10123404095019
Cu	0.74929838366027	2.04033709539377	-1.91142606311069
C	-0.27858335208560	3.58153504571190	-1.47098271152692
N	-0.85861688145339	4.55272410413046	-1.25558907752237

$TS_{III\text{-}IV}$
−1 1

C	-1.79679985086977	-0.08949984400919	1.28071152005440
C	-0.69560337941088	0.65580837187293	1.70157414442497
C	-0.02953345033308	1.46396250339983	0.79245769986596
C	-0.47995714531611	1.47070158634041	-0.51459180196228
C	-1.58830347679124	0.76913188386437	-0.95348997309983
C	-2.24829793441156	-0.03615929164504	-0.03787279449547
C	-2.48632194721143	-0.92045612681911	2.22595263263294
N	4.14759172498700	2.88774581576579	-0.27524574009118
N	3.09569105761843	2.71575880834629	-0.47885061426294
H	-0.36646845155139	0.60867520108710	2.73097184544653
H	0.81714729895016	2.06055536163603	1.10489253011560
H	-1.93091385363519	0.83239084694554	-1.97793150753685
H	-3.11023783481306	-0.61442269733454	-0.34304055822608
N	-3.03799745399680	-1.58517816534753	2.98158894071700
S	2.55335923432465	1.06163265245508	-3.46251688163398
O	2.25509217386114	0.43490952721117	-4.73257960035664
O	1.96024225326597	2.46661508689846	-3.40212614899465
O	1.76300196566242	0.39161816153048	-2.33589847559432
O	3.96464053756355	1.08978662580469	-3.14099983700310
Cu	0.75269599506506	2.01348407661723	-1.90430152101013
C	-0.35239062904308	3.43977337512038	-1.38350836141217
N	-0.92869983391484	4.42073324025961	-1.20190849757775

IV
−1 1

C	-1.02001655445438	0.18410082330035	0.85896664211350
C	-0.60069080068214	1.22825727343095	1.66632606966339
C	-0.63733460616422	2.53241297039980	1.17984037676119
C	-1.09142873734340	2.77147567357420	-0.10649541299962
C	-1.52891299718170	1.71895866730960	-0.93606992994193
C	-1.49247711710404	0.41435903121532	-0.44915704076670
C	-0.99313071387967	-1.15944349580961	1.36381393516005
N	3.22992132567278	1.64763587559431	0.10826779438324

N	2.18108699799365	1.47127029936203	-0.10864419486226
H	-0.24645877598150	1.03086370508327	2.66868053462027
H	-0.31029037901297	3.35445402228304	1.80158907374491
H	-1.97088790986996	1.93703298712795	-1.90087809377999
H	-1.90586868528761	-0.40320783803374	-1.02742538660786
N	-0.97365614159321	-2.23306293566732	1.76675771182024
S	2.35699534660195	0.19592480212804	-3.39343161279078
O	2.26252673909605	-0.34686382805468	-4.74750261277934
O	1.55385920501352	1.47141470564683	-3.28329555458812
O	1.68889842190802	-0.73615015818734	-2.40833361907237
O	3.74313756289674	0.43883657972362	-2.99548685641065
Cu	0.30989273857029	0.71329197885755	-1.76817421358057
C	-1.13208365261051	4.11612373625458	-0.60715998863213
N	-1.16514226658770	5.18988012446125	-1.00890362145444

NO_2 release from nitrate ion and SO_2

DFT Study

Although the first step of this reaction is endergonic, $^{2}SO_3^{-}$ further transforms with the aid of water to bisulfate ion (see the next section) consistent with experiment. As a result, the overall reaction becomes exergonic.

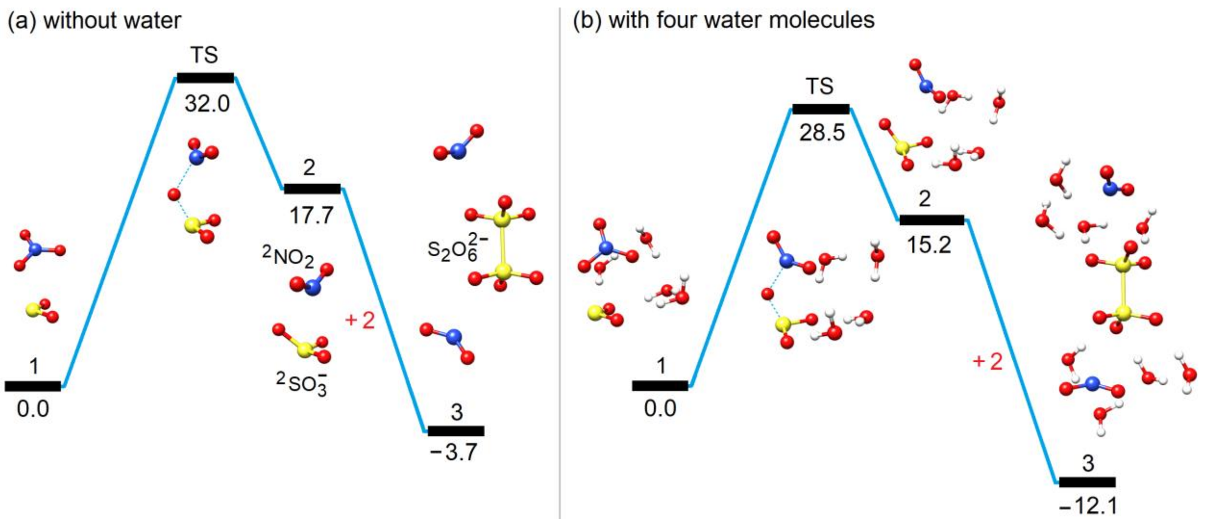

Fig S21. DFT calculations for the NO_2 release path from nitrate ion and SO_2 at the M06-2X-D3(0)/ma-def2-QZVP//M06-2X-D3(0)/ma-def2-TZVP level using ORCA.

Table S11. The electronic energy Δ*E*, enthalpy Δ*H*, and free energy Δ*G*298 of the stationary points (in kcal/mol) for the NO_2 release path from nitrate ion and SO_2 computed at the M06-2X-D3(0)/ma-def2-TZVP and M06-2X-D3(0)/ma-def2-QZVP//M06-2X-D3(0)/ma-def2-TZVP levels, treating acetonitrile solvent molecules implicitly with the SMD model. All the energies are given with respect to those of **I**.

	M06-2X-D3(0)/ma-def2-TZVP			M06-2X-D3(0)/ma-def2-QZVP		
	ΔE	ΔH	ΔG_{298}	ΔE	ΔH	ΔG_{298}
Without water						
**I**	0.00	0.00	0.00	0.00	0.00	0.00
**TS**	33.1	31.4	32.0	33.1	31.4	32.0
**II**	19.7	18.9	17.8	19.5	18.8	17.7
**III**	−8.0	−6.4	−3.7	−8.0	−6.4	−3.7
With four water molecules						
**I**	0.00	0.00	0.00	0.00	0.00	0.00
**TS**	29.5	27.6	28.5	29.5	27.6	28.5
**II**	17.3	16.4	15.1	17.1	16.3	15.0
**III**	−17.0	−16.8	−12.2	−16.9	−16.6	−12.1

Single-Point CCSD(T) Study in the Presence of Four Water Molecules

Since correlated ab initio energies typically converge slowly with respect to basis set size, the basis set dependence of each CCSD(T) component (UHF, see Table S12; C-CCSD and (T), see Table S13) was investigated. The best available CCSD(T) energies are summarized in Table S14.

Table S12. The single-point **UHF** electronic energy of the stationary points (in kcal/mol) for the NO_2 release path from nitrate ion and SO_2 in the presence of catalytic water molecules, treating acetonitrile solvent molecules implicitly with the SMD model. All the energies are given with respect to those of **I**.

	Finite Basis Energies				CBS-Extrapolated Energies		
	aDZ	aTZ	aQZ	a5Z[a]	CBS(2/3)	CBS(3/4)	CBS(4/5)[a]
**I**	0.0	0.0	0.0	0.0	0.0	0.0	**0.0**
**TS**	29.8	23.6	22.8	21.8	23.4	22.4	**21.5**
**II**	6.6	−3.4	−5.4	−7.0	−3.8	−6.1	**−7.7**
**III**	−10.6	−35.6	−41.7	−46.2	−36.6	−44.0	**−47.9**

[a] The UHF energies computed with a5Z and CBS(4/5) are very close to each other, indicating that the CBS(4/5) extrapolated energies are close to basis set convergence.

Table S13. The single-point **CCSD** and **(T)** electronic **correlation** energy contributions to the stationary points (in kcal/mol) on the NO_2 release path from nitrate ion and SO_2 in the presence of catalytic water molecules, treating acetonitrile solvent molecules implicitly with the SMD model. All the energies are given with respect to those of **I**.

	C-CCSD[a]					(T)[b]		
	Finite Basis Energies			Extrap.		Finite Basis Energies		Extrap.
	aDZ	aTZ	aQZ	CBS(2/3)	CBS(3/4)	aDZ	aTZ	CBS(2/3)
**I**	0.0	0.0	0.0	0.0	**0.0**	0.0	0.0	**0.0**
**TS**	5.5	6.7	6.7	7.4	**6.7**	−1.9	−1.8	**−1.8**
**II**	18.2	19.6	19.3	20.5	**19.0**	2.2	2.3	**2.4**
**III**[c]	**22.2**	–	–	–	–	**3.1**	–	–

[a] The C-CCSD energies obtained with aQZ and CBS(3/4) are nearly identical, suggesting that the latter is effectively converged.
[b] The (T) contribution exhibits minimal basis set dependence, with aDZ already providing effectively converged values.
[c] Correlation calculations on structure **III** are only feasible with aDZ. For structure **II**, the C-CCSD/aDZ energy deviates from its CBS(3/4) value by 0.8 kcal/mol. Given that structure **III** is twice the size of **II**, a proportional error (0.8 x 2 = 1.6 kcal/mol) can be expected due to the size-extensive nature of the error in computed correlation energies. Thus, the C-CCSD/aDZ energy of **III** is expected to be accurate within 2 kcal/mol.

Table S14. Best available single-point CCSD(T) estimates of electronic energy ΔE, enthalpy ΔH, and free energy ΔG_{298} (kcal/mol) for the stationary points along the NO_2 release path from nitrate ion and SO_2 in the presence of catalytic water molecules, treating acetonitrile solvent molecules implicitly with the SMD model. ΔE values are obtained by summing the bolded values in Tables S12 and S13; ΔH and ΔG_{298} values are derived by adding the corresponding DFT corrections from Table S11 to the CCSD(T) electronic energies. All the energies are given with respect to those of **I**.

	ΔE	ΔH	ΔG_{298}
**I**	0.0	0.0	0.0
**TS**	26.5	24.6	25.5
**II**	13.7	12.8	11.5
**III**	−22.6	−22.3	−17.8

Cartesian coordinates (Å) of all stationary points given on Fig S15(a)

I

−1 1

N	-0.93394234928634	5.12127312477711	-5.99147856684126
O	-0.72258403148837	4.18951071387024	-6.76072952035860
O	-2.04035814445855	5.64023385212670	-5.87482428019162
O	0.03099431223395	5.56670607898306	-5.28279104096965
S	0.99667182814394	3.90001392612005	-4.07355197739137
O	2.05290424849904	3.47154758669703	-4.94697990892177
O	-0.14884886364363	3.03310371742553	-4.01072970532534

TS

−1 1

N	-0.97529236732923	5.04682777474517	-6.16110135981194
O	-0.61227548571668	4.52710418596836	-7.16036103306726
O	-2.04090333469029	5.42493667389712	-5.81770906612676
O	0.31617753858964	5.30786091935078	-4.86072585148396
S	0.91645434406520	4.05710206232274	-4.16965446953686
O	1.82612398125479	3.34165737325676	-5.07139364491537
O	-0.19544767617339	3.21690001045870	-3.70013957505728

II

−1 1

N	-1.03095991673018	4.76073901199085	-6.48017271416311
O	-0.61123840795467	5.22169165161260	-7.48096425492004
O	-2.11601268282701	4.62017748161279	-6.03455457809736
O	0.73875779996383	5.48360612219336	-4.46490215102994
S	1.01309454079175	4.07752692967830	-4.13100664675862
O	1.05934528193363	3.20302859340969	-5.31189069221328
O	0.18185038482267	3.55561920950234	-3.03759396281754

III

−2 1

N	7.17069776451565	4.62192390755854	0.43317270803744
O	7.45979013531517	5.71799049539974	0.75953680071003
O	6.71746656464162	3.68760359750390	0.99097886913775
O	5.01405435340496	5.20437656995648	-1.18577800474144
S	5.66740779612221	5.28016944409797	-2.48703035182784
O	5.82998530362975	6.63956877971735	-2.98273156694358
O	6.87580606934138	4.47118796432706	-2.57819289049757
N	1.16008796324134	3.11529446375127	-3.81082086741327
O	0.92878704736128	3.11041928355437	-4.96746925110205
O	0.55037445331655	3.42111050715891	-2.84914846254020
O	3.06223028615703	5.15442819470915	-3.75693732457049
S	4.27511001950487	4.35173068751457	-3.85850630451424
O	4.92290689012031	4.43299073309659	-5.16007574397253
O	4.11570623033236	2.99065410856897	-3.36364525854410

Cartesian coordinates (Å) of all stationary points given on Fig S15(b)

I

−1 1

N	-1.42134086201651	5.49991743539158	-5.13854080575737
O	-0.52215385920935	4.75034035049212	-5.49834485649735
O	-2.60203864531641	5.24819778228483	-5.31375649626595
O	-1.11096841917991	6.59898392916137	-4.54644687082416
S	0.68917458667374	6.52130966426888	-3.39709178280700
O	1.70272414816459	6.41814131919201	-4.41675223240886
O	0.42310452512297	5.27458084903272	-2.70607875529872

O	2.23896044271981	3.95413465872124	-5.95000162459654
H	2.02917085460002	4.80263397445605	-5.52700625828455
H	1.66477712142750	3.92456579444091	-6.72560382272466
O	0.86974874830114	2.58909741790374	-3.85535962251781
H	1.34100201533965	2.87920593812725	-4.65749548186841
H	0.74925618342975	3.41747444449020	-3.36693133776874
O	-1.90231727341322	2.39025196780476	-4.34719995376865
H	-2.10414622109389	3.04262735019073	-5.03135212436738
H	-0.92592872551690	2.37556116157260	-4.30232395424351
O	-2.17172367404880	3.96617793901497	-1.98159771350678
H	-2.18337985258129	3.40412850783271	-2.77661593730158
H	-1.38142509340288	4.51072751562125	-2.07954736919192

TS

−1 1

N	-1.57285432527569	5.72334152100181	-5.14438983484320
O	-0.99717254802235	4.76016699436276	-5.50821365007106
O	-2.63701711634056	6.16557692053175	-5.36462714339044
O	-0.58922931329114	6.87041461717615	-3.99111713846103
S	0.71624286779926	6.40194881536653	-3.33856610818965
O	1.74903663950944	6.14170268326671	-4.35910832971989
O	0.44138835371957	5.19412947776679	-2.51997582745065
O	2.00621258789013	3.98064941406163	-6.07737985099556
H	1.89771320756241	4.80826743276968	-5.56926119800615
H	1.36032204080576	4.02870668383458	-6.79196334996816
O	0.96842140855074	2.70391940469707	-3.78489091923759
H	1.34460379910029	2.99101324814618	-4.63743495088664
H	0.84850320367878	3.53436520583957	-3.28903968200618
O	-1.63300995898211	1.87181879886392	-4.03399636578002
H	-1.90719377604768	1.92573542304749	-4.95697274559510
H	-0.67890683214657	2.10510627277990	-4.02423298231963
O	-2.32467466030343	4.27521189762848	-2.63244674287905
H	-2.16846249220220	3.43648668830283	-3.09920532132442
H	-1.44142908600464	4.64949550055605	-2.49522385887550

II

−1 1

N	-1.82313495666482	5.49082602045648	-5.03465077034232
O	-1.09728096931549	4.81499763498232	-5.67626724130931
O	-2.81331725757233	6.08514020042971	-5.26430576109770
O	-0.16339872631230	7.38491911903305	-3.86702547372905
S	0.83672330049205	6.45663965206322	-3.34121985614756
O	1.88645998879044	6.08579081706977	-4.30309667546209
O	0.24534258504619	5.27957875049720	-2.66675627739787
O	2.13600131287099	3.93847333811959	-6.07542374280292
H	2.04170118006056	4.77659304437815	-5.58800240972363
H	1.57826406871486	4.02613630065522	-6.85768706218061
O	0.93675248682568	2.74760801487156	-3.81845808994911
H	1.35872809075152	2.99918588349459	-4.66034033843259
H	0.78234189550289	3.59490254738038	-3.36590960095726
O	-1.64057307206414	1.85234196505021	-4.04733275603137
H	-1.93452428058162	1.88462286259739	-4.96510261372029
H	-0.69024362894984	2.09922064882631	-4.05668253104208
O	-2.43056073355154	4.11887082931632	-2.51220670765621
H	-2.22688935757996	3.31612445973519	-3.02312284568250
H	-1.59989692646313	4.61608591104305	-2.49445624633523

III

−2 1

N	7.70312418634436	4.89438321223732	0.33392753373893

O	7.84483908015640	6.04211651427116	0.10078515448435
O	7.54111332125261	4.26474842408259	1.31587695546809
O	5.09053444313290	4.75401442531969	-0.84207453187044
S	5.55793972360753	4.99103780467190	-2.18994006112230
O	5.68065420848896	6.39530873353908	-2.55674928794220
O	6.73509028169270	4.20005805810469	-2.55504682069194
O	7.97439736470555	7.98830894401509	-2.45950011587803
H	7.12726897194847	7.50332926889315	-2.45620549317605
H	8.05583031316116	8.36824408435724	-1.57656671194298
O	9.24161507688277	5.49912045181005	-2.82551188001583
H	8.97087614372964	6.42323620191324	-2.67103250073177
H	8.39789108694227	5.01271935400386	-2.83232618725525
O	10.40038265237274	4.11822864751009	-0.77240288032230
H	10.71725635381559	4.66657797058541	-0.04594137772160
H	10.08030982251037	4.72776343892271	-1.47250839300240
O	8.35260520858268	2.16390609286374	-1.29773595787112
H	9.06931335266867	2.73703622357744	-0.98383085825932
H	7.69346530021254	2.77751026809272	-1.65566895094193
N	0.96928295934457	3.23146060797122	-4.43739122535364
O	1.41774664927912	3.35664334394529	-5.52138470776119
O	-0.03621268795054	3.55472161742534	-3.91627223129937
O	2.82392608427556	5.00663185984286	-3.14236920001148
S	4.00440127743255	4.23832930068007	-3.47342510883261
O	4.51042772245420	4.44763450098135	-4.82263607341199
O	3.90754362749373	2.82236033384243	-3.10994804417637
O	4.10701200842324	2.84242678162017	-7.07487764108014
H	4.25589360815998	3.46421057612347	-6.33730920673456
H	3.33058503831742	3.17784619865967	-7.53853021055970
O	3.54144529052227	0.87363317699446	-5.13508046968366
H	3.67661874948591	1.42079432757540	-5.93060452087471
H	3.74267641929201	1.48451802935360	-4.40371362861530
O	0.98996751520813	0.22068837667426	-4.40448107200827
H	0.33142704286196	0.34438014837643	-5.09709374283090
H	1.86611861176480	0.40991505077168	-4.80560775576135
O	1.71819016600962	1.37178540752236	-1.87566104489400
H	1.32381180728720	0.99813320146240	-2.67933090403981
H	2.41988775812938	1.95665625140565	-2.19918160701626

Water-assisted bisulfate ion formation from $SO_3^{\bullet -}$

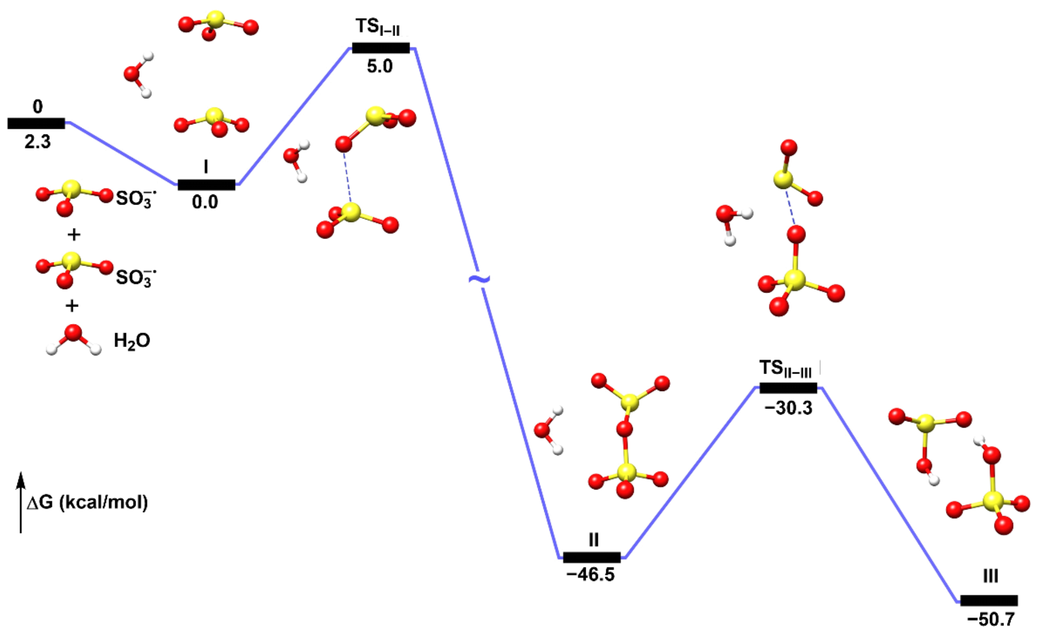

Fig S22. DFT calculations for the water-assisted bisulfate ion formation path from SO_3^- at the M06-2X-D3(0)/ma-def2-QZVP//M06-2X-D3(0)/ma-def2-TZVP level using ORCA.

Table S15. The electronic energy ΔE, enthalpy ΔH, and free energy ΔG_{298} of the stationary points (in kcal/mol) for the water-assisted bisulfate ion formation path from SO_3^- computed at the M06-2X-D3(0)/ma-def2-TZVP and M06-2X-D3(0)/ma-def2-QZVP//M06-2X-D3(0)/ma-def2-TZVP levels, treating acetonitrile solvent molecules implicitly with the SMD model. All the energies are given with respect to those of **1**.

	M06-2X-D3(0)/ma-def2-TZVP			M06-2X-D3(0)/ma-def2-QZVP		
	ΔE	ΔH	ΔG_{298}	ΔE	ΔH	ΔG_{298}
**0**	8.95	5.27	2.47	8.78	5.10	2.30
**I**	0.00	0.00	0.00	0.00	0.00	0.00
**TS$_{I-II}$**	5.02	3.97	4.71	5.33	4.27	5.02
**II**	−49.37	−48.76	−46.61	−49.21	−48.60	−46.45
**TS$_{II-III}$**	−31.87	−32.06	−30.84	−31.33	−31.51	−30.30
**III**	−54.32	−54.41	−50.99	−54.06	−54.15	−50.73

Cartesian coordinates (Å) of all stationary points given on Fig S16

$SO_3^{-\cdot}$
−1 2

S	4.78431743453369	6.63405822287304	-0.21407129665123
O	3.31696083426737	6.67854868806722	-0.12444684439556
O	5.30989802240509	7.08453073595825	-1.51169131860128
O	5.36569560978689	5.36549552259769	0.25012131049001

H_2O
0 1

O	6.81588774054396	5.36813770166798	-3.21286436577396
H	6.22253942961716	4.60884370637329	-3.24952285045860

H	6.38095946285764	5.99588955609713	-2.62386025166411

I

−2 1

S	4.78971627664087	6.62563498780265	-0.18820350980111
O	3.32581795039944	6.69573609255719	-0.11685583401646
O	5.33190058135894	7.07731080411594	-1.48516684424743
O	5.35652145491311	5.35123379250358	0.26741299102194
S	3.60409781402957	3.94998612068731	-2.57547896850833
O	4.83062006772519	3.31099378382608	-3.09806971787276
O	2.78548377068480	3.04120521411235	-1.76542485220628
O	2.85116135423356	4.67772616479781	-3.60440799331171
O	6.82841186310908	5.37640744797673	-3.23453403254575
H	6.22480194617351	4.61715900361007	-3.26650183170640
H	6.38543692073181	5.99143458801012	-2.62679940680565

$TS_{I\text{-}II}$

−2 1

S	4.71929497088772	6.55696615716750	-0.25929289434787
O	3.38021528193035	6.78531344540273	0.30736975352488
O	4.65017928561038	6.23273435164467	-1.72934583390251
O	5.48855662429968	5.53831959869627	0.47608502617666
S	3.69923609102025	4.16322755084234	-2.63136411302187
O	4.90145223943850	3.37953087577583	-2.96534073271802
O	2.81366388347123	3.48533555846697	-1.68246158143179
O	3.01627729674482	4.70239760882569	-3.80777115314904
O	6.90779900098630	5.39311388255828	-3.30783287145378
H	6.41145572349800	4.56108039289811	-3.35790356097739

II

−2 1

S	4.85377244725813	5.52222649227871	-0.28216057210800
O	5.66057080566260	6.70783527623086	-0.62459400335415
O	3.83958320160745	5.47464906163472	-1.77176566699138
O	3.83348064921993	5.84125787815915	0.71682319325251
S	3.69315451699216	4.11004052855002	-2.56493989932601
O	5.02170932712180	3.81631430401413	-3.10777354638377
O	3.25422356566256	3.10789884945991	-1.60868249338550
O	2.71548409805250	4.41133671963131	-3.59343567375225
O	6.76724024748269	6.04447743761624	-3.20485615621806
H	6.19648799414811	5.26120205725120	-3.29611404018184
H	6.47826314679184	6.41759039517351	-2.35653014155146

$TS_{II\text{-}III}$

−2 1

S	0.96553460024382	1.16414428467728	2.03437320028333
O	0.36256683117092	2.44016994008760	1.80265828610802
O	-1.08214507292513	0.32965721255844	-0.50995623304682
O	0.19581380685777	0.21141118908474	2.77296468873523
S	-1.04276413556891	-1.11748421483749	-0.89002439538320
O	0.33630676248898	-1.62880924492005	-0.63983078910002
O	-2.01426716689101	-1.86977226131443	-0.07154042473906
O	-1.36745986100172	-1.25420091738458	-2.32612019331759
O	1.54410689067329	0.85761055535458	-0.71126748642367
H	1.53159844683181	-0.11795594969552	-0.71800050101881
H	0.57070889812020	0.98522940638944	-0.74325615209740

III

−2 1

S	6.34820833520925	6.55292144296662	-0.78770897045571

O	5.33171002071665	6.89424904248167	0.23123814628819
O	3.62049005203120	5.54124342102584	-2.94525566577548
O	6.35138333027576	5.05643582839009	-1.00375154439211
S	3.67831946357701	4.07188530838921	-2.90883908608216
O	4.26119913676493	3.70293969920691	-1.49529506493192
O	2.36439381528739	3.46060164139463	-2.93371983723810
O	4.59238156346070	3.53916865964015	-3.90607580950372
O	5.72035324555564	7.09549736315568	-2.19655050128528
H	5.09917566500350	4.27063260588301	-1.29217451582408
H	4.94635537211703	6.52925398746517	-2.45589615079927

SPECTROSCOPIC DATA

^{1}H NMR spectrum of Darunavir-derived benzonitrile 1

DMSO-d_6, 25°C, 500 MHz

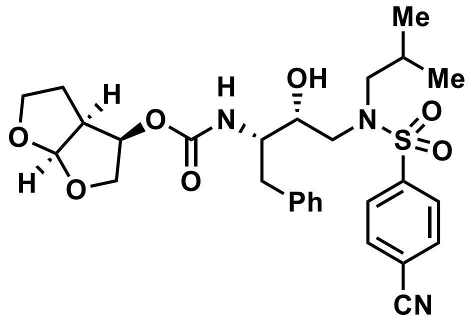

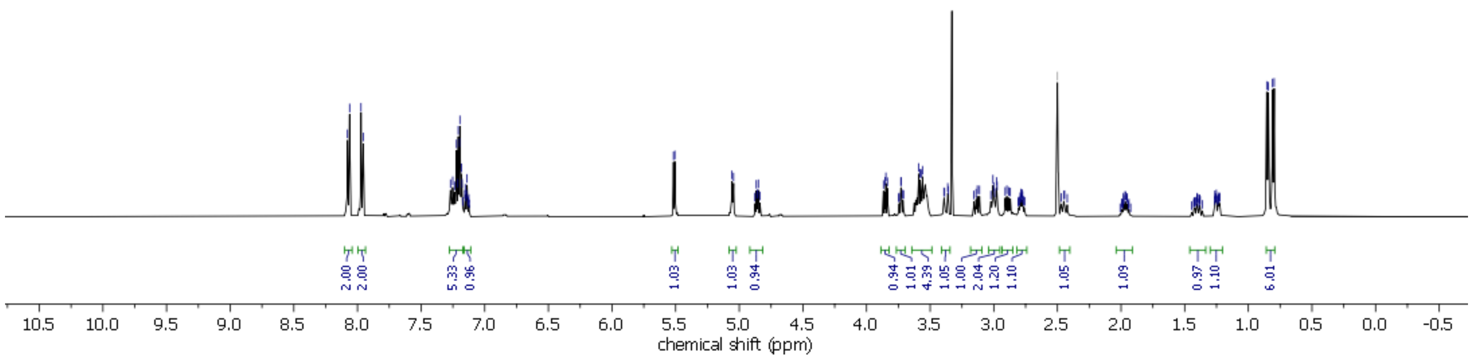

^{13}C NMR spectrum of Darunavir-derived benzonitrile 1

DMSO-d_6, 25°C, 125 MHz

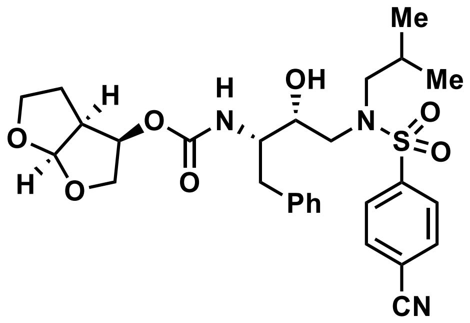

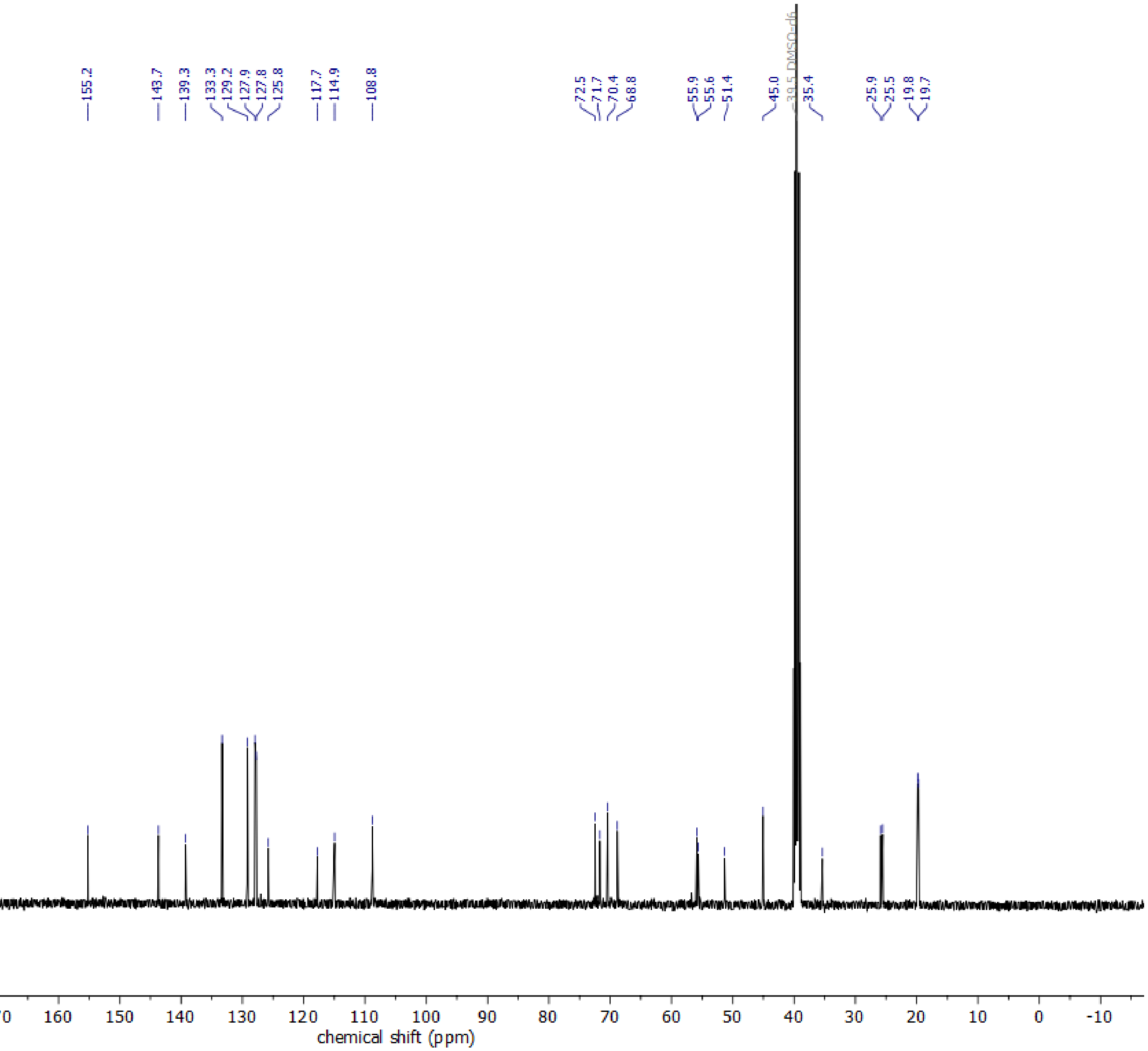

^{1}H NMR spectrum of 2-methyl-3-nitrobenzonitrile (2)

$CDCl_3$, 25°C, 500 MHz

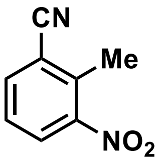

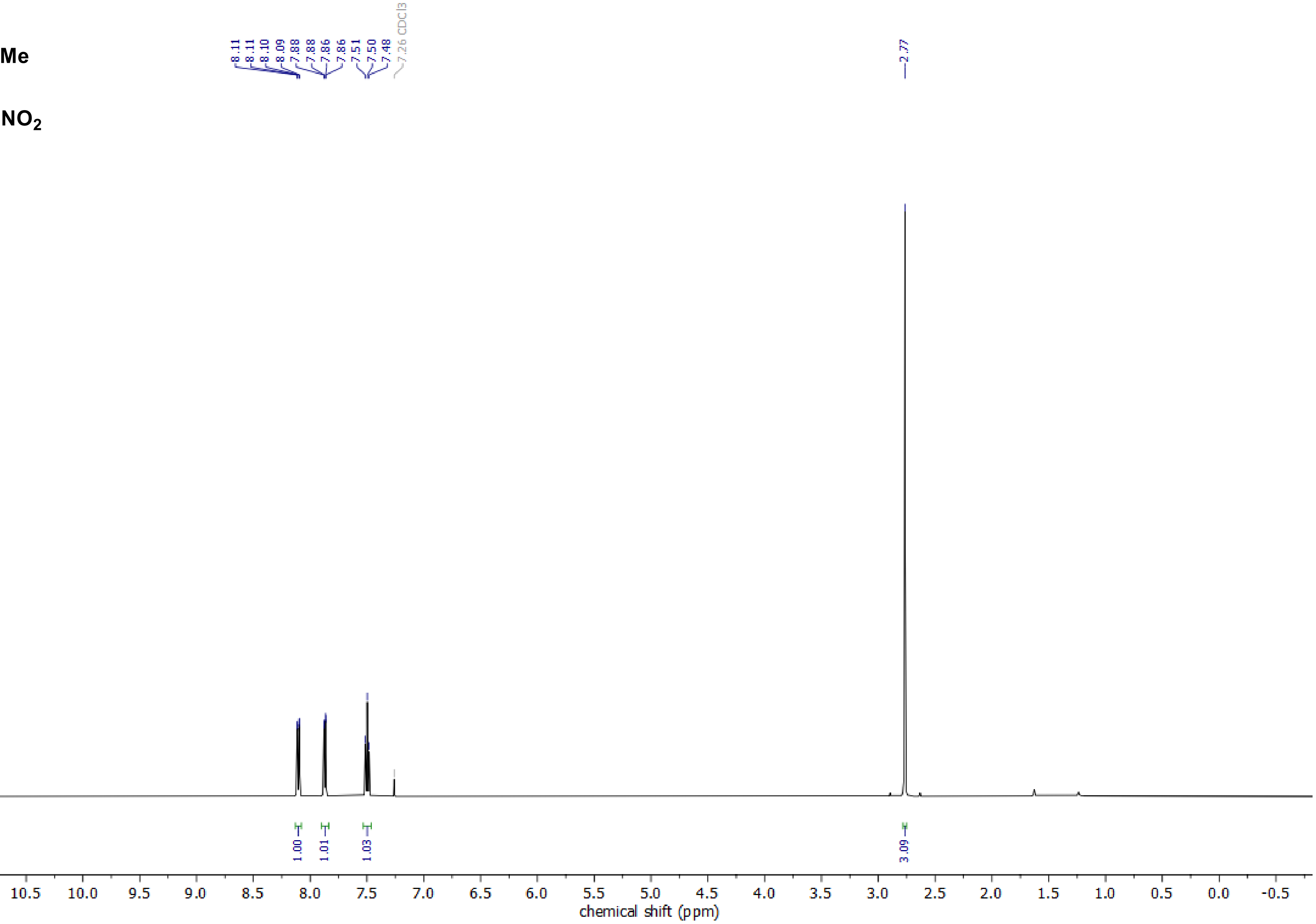

^{13}C NMR spectrum of 2-methyl-3-nitrobenzonitrile (2)

$CDCl_3$, 25°C, 125 MHz

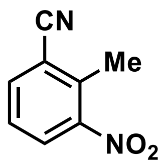

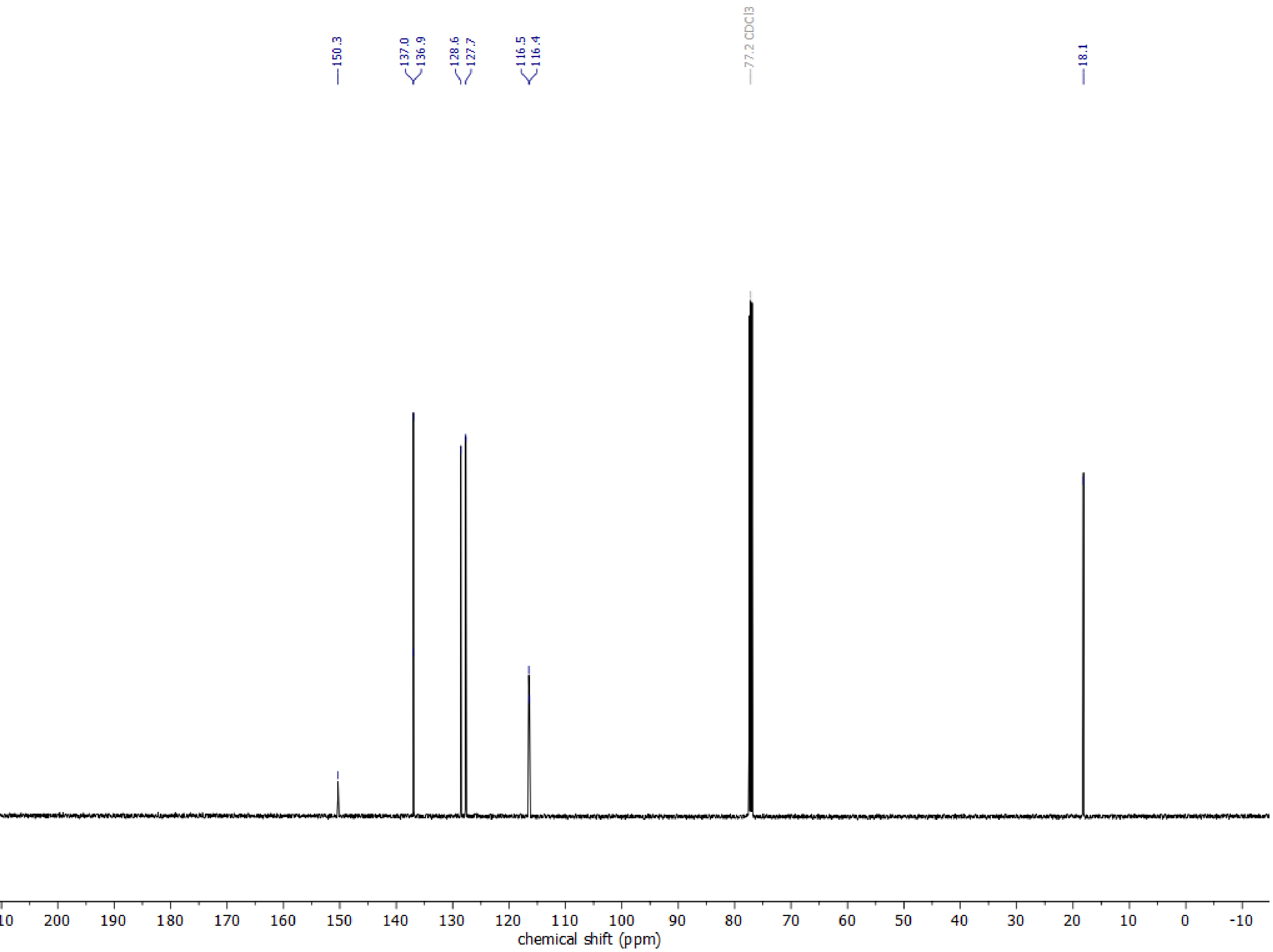

^{1}H NMR spectrum of 2-chloroterephthalonitrile (3)

$CDCl_3$, 25°C, 500 MHz

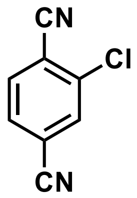

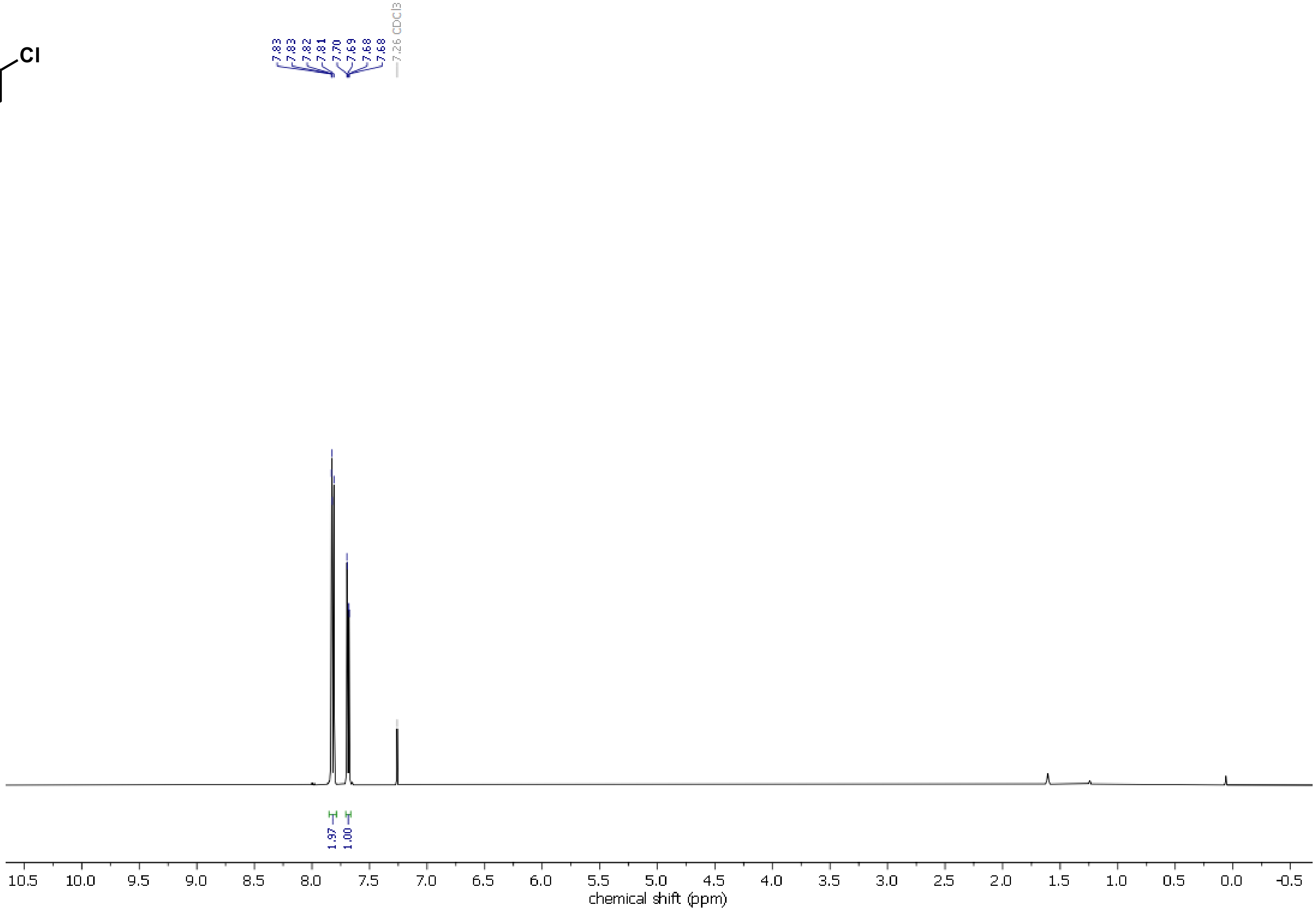

^{13}C NMR spectrum of 2-chloroterephthalonitrile (3)

$CDCl_3$, 25°C, 125 MHz

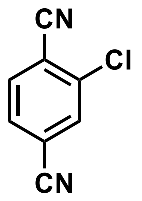

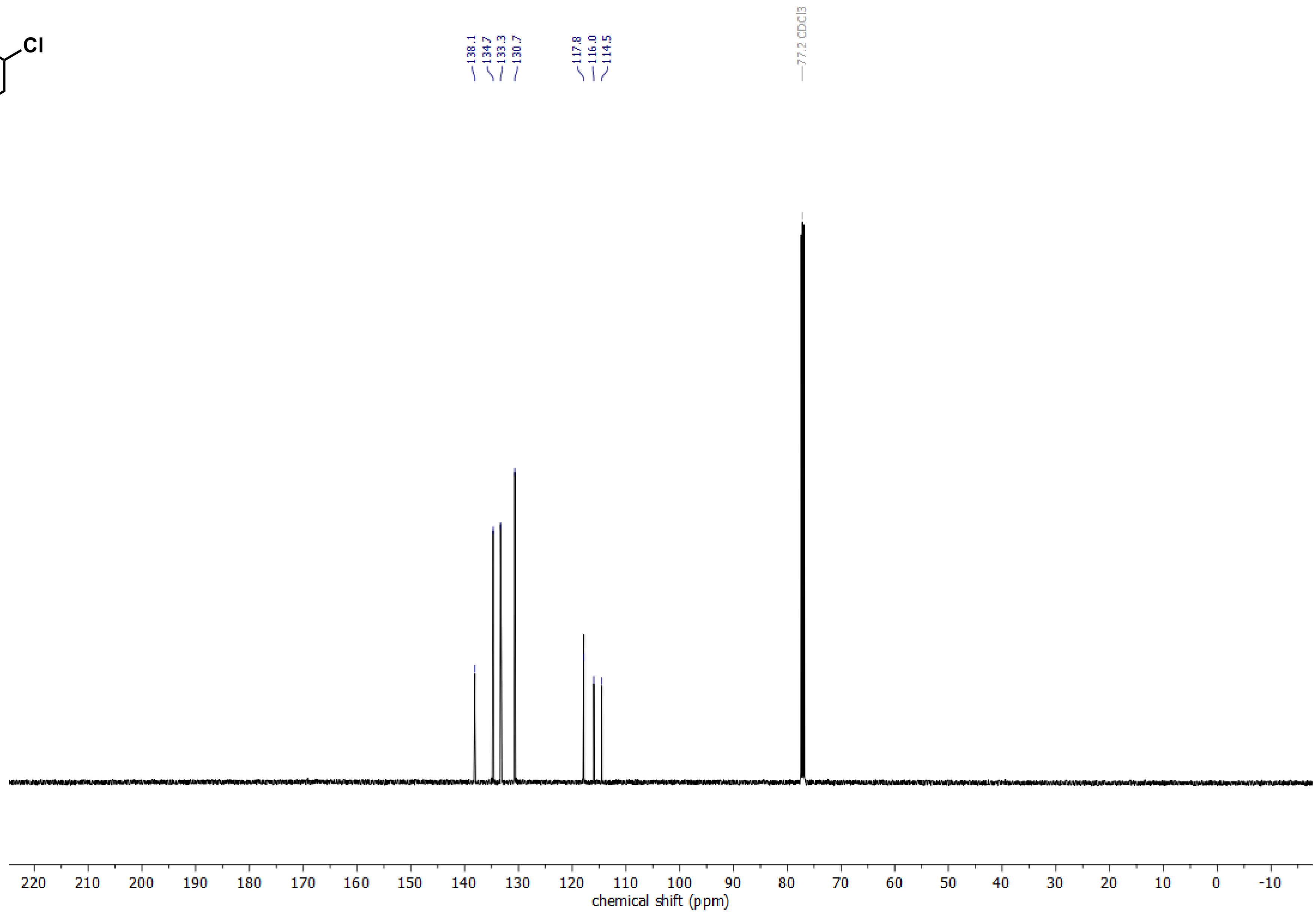

^{1}H NMR spectrum of 2-(trifluoromethylthio)benzonitrile (4)

$CDCl_3$, 25°C, 500 MHz

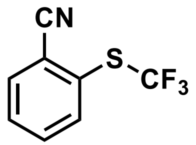

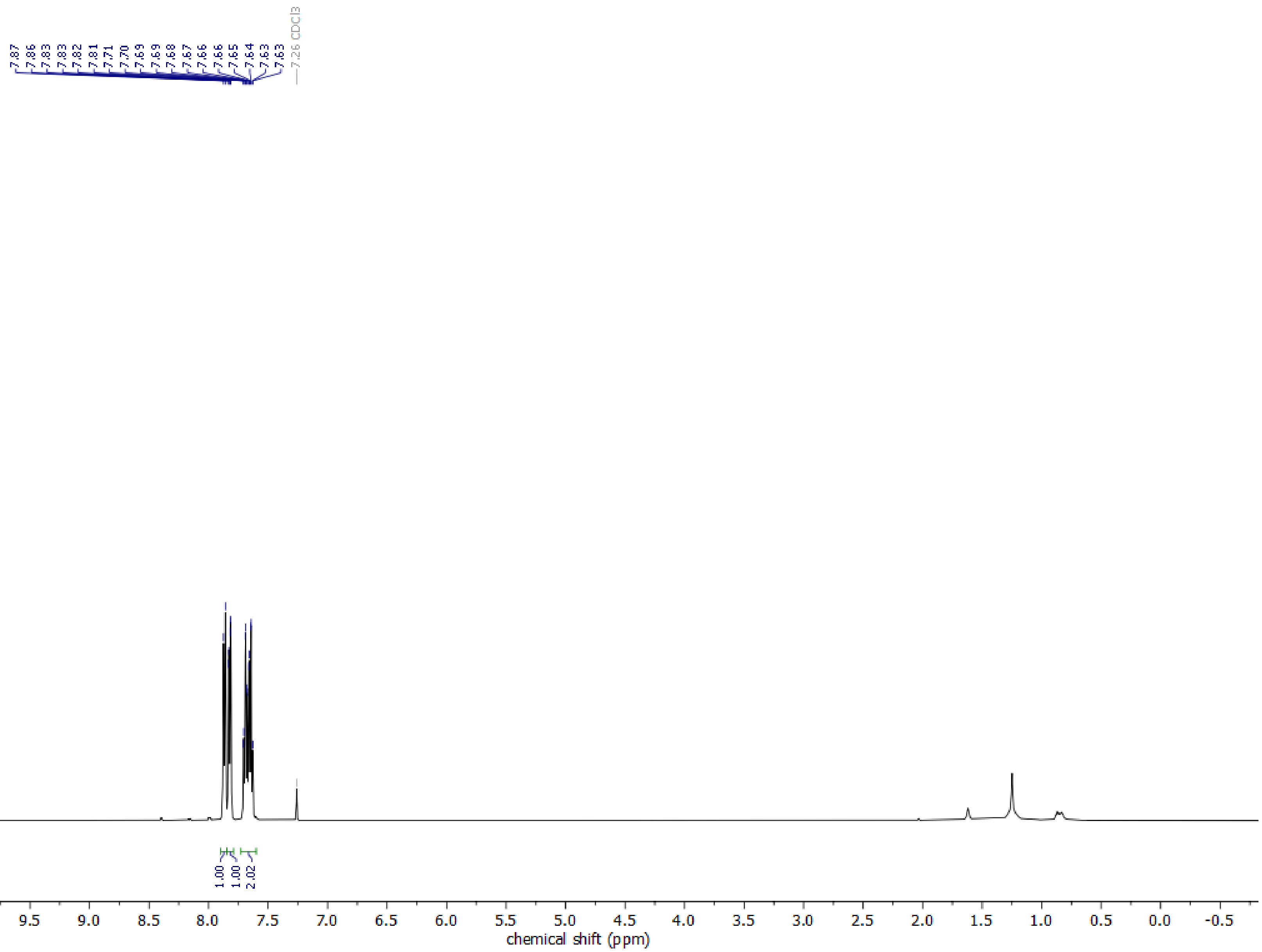

^{19}F NMR spectrum of 2-(trifluoromethylthio)benzonitrile (4)

$CDCl_3$, 25°C, 470 MHz

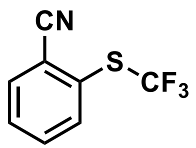

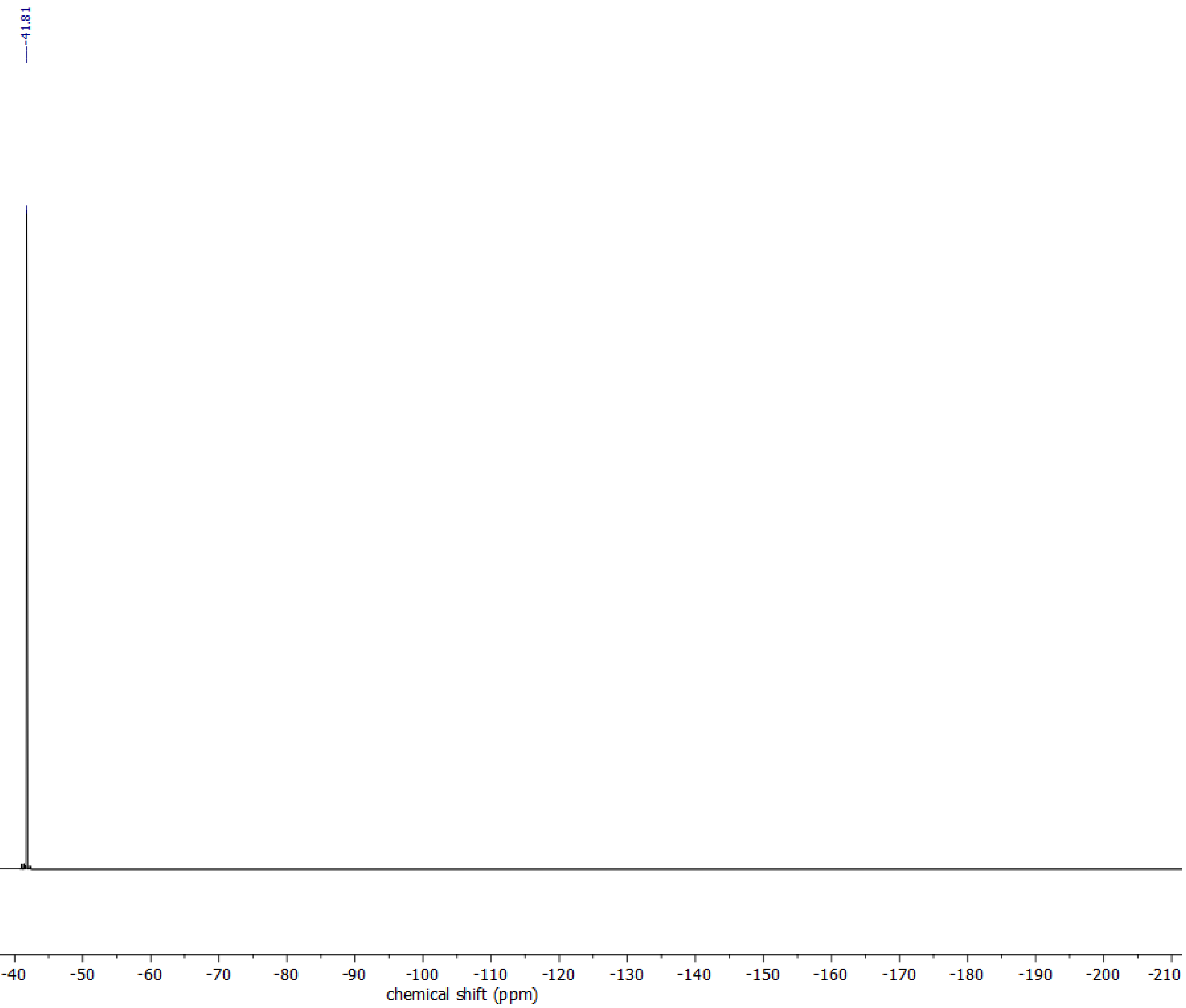

^{13}C NMR spectrum of 2-(trifluoromethylthio)benzonitrile (4)

$CDCl_3$, 25°C, 125 MHz

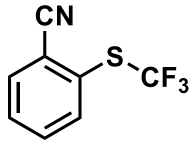

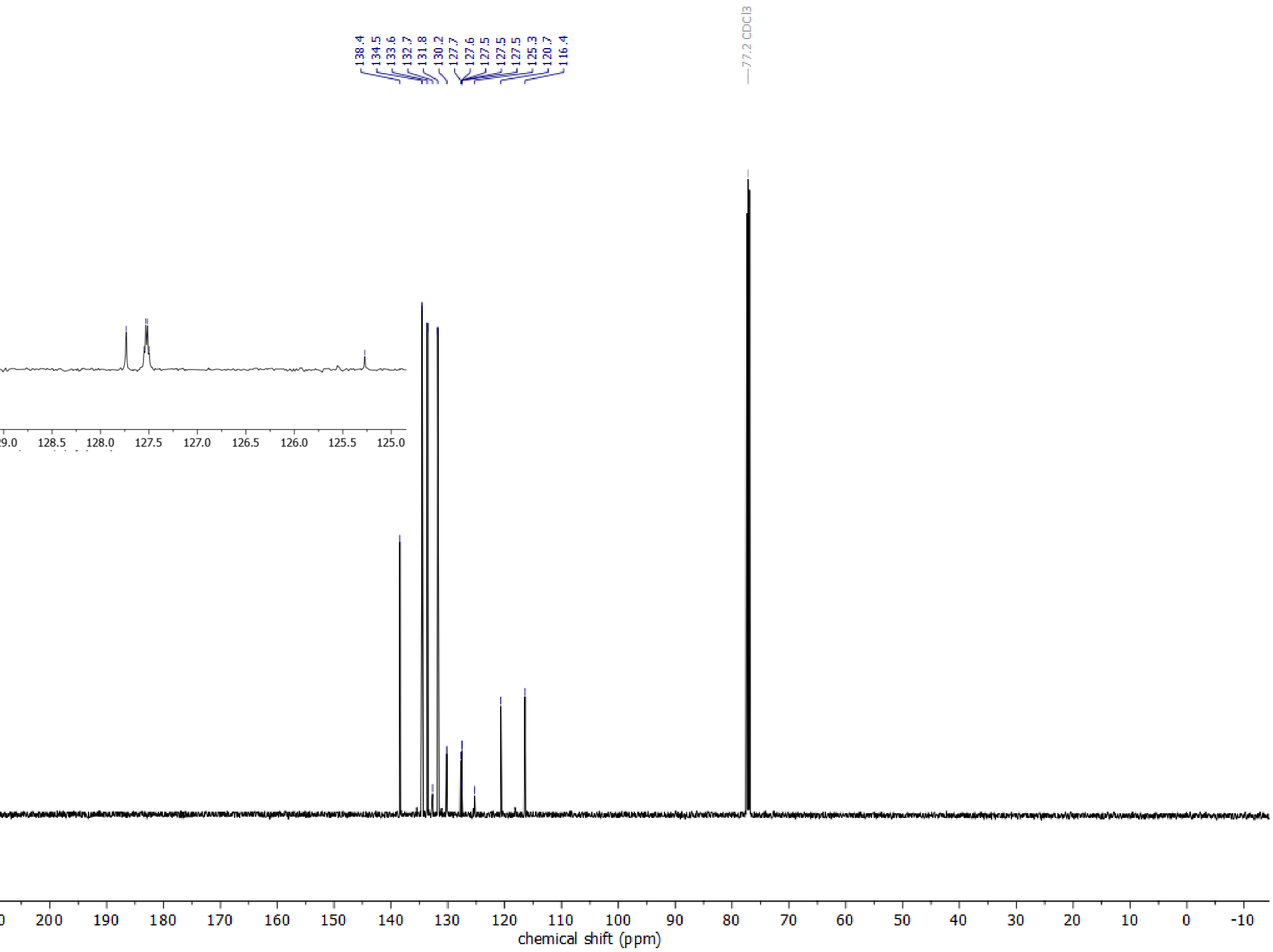

^{1}H NMR spectrum of 6-bromo-5-methylnicotinonitrile (5)

$CDCl_3$, 25°C, 500 MHz

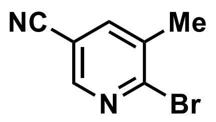

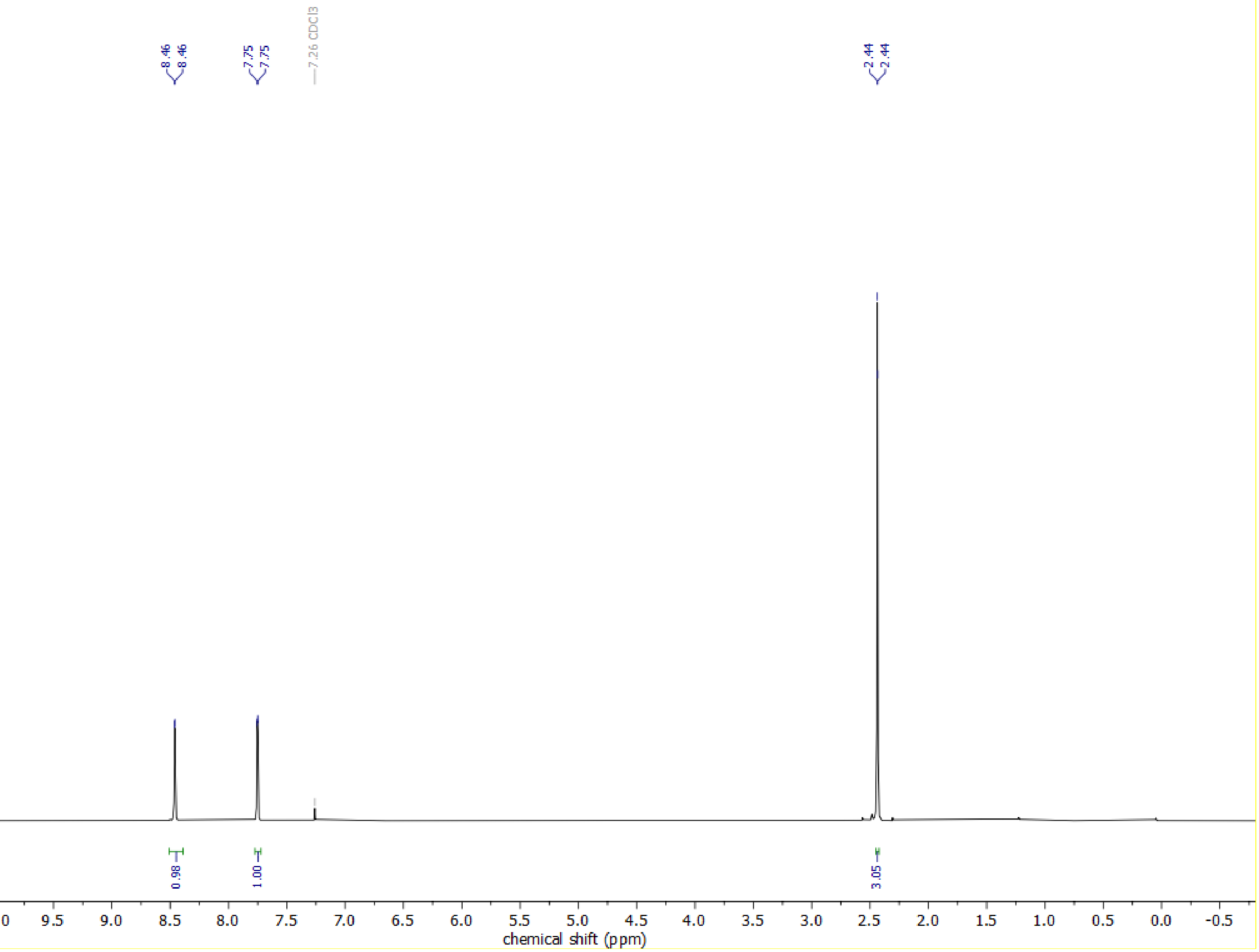

^{13}C NMR spectrum of 6-bromo-5-methylnicotinonitrile (5)

$CDCl_3$, 25°C, 125 MHz

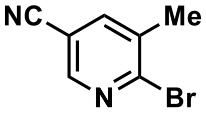

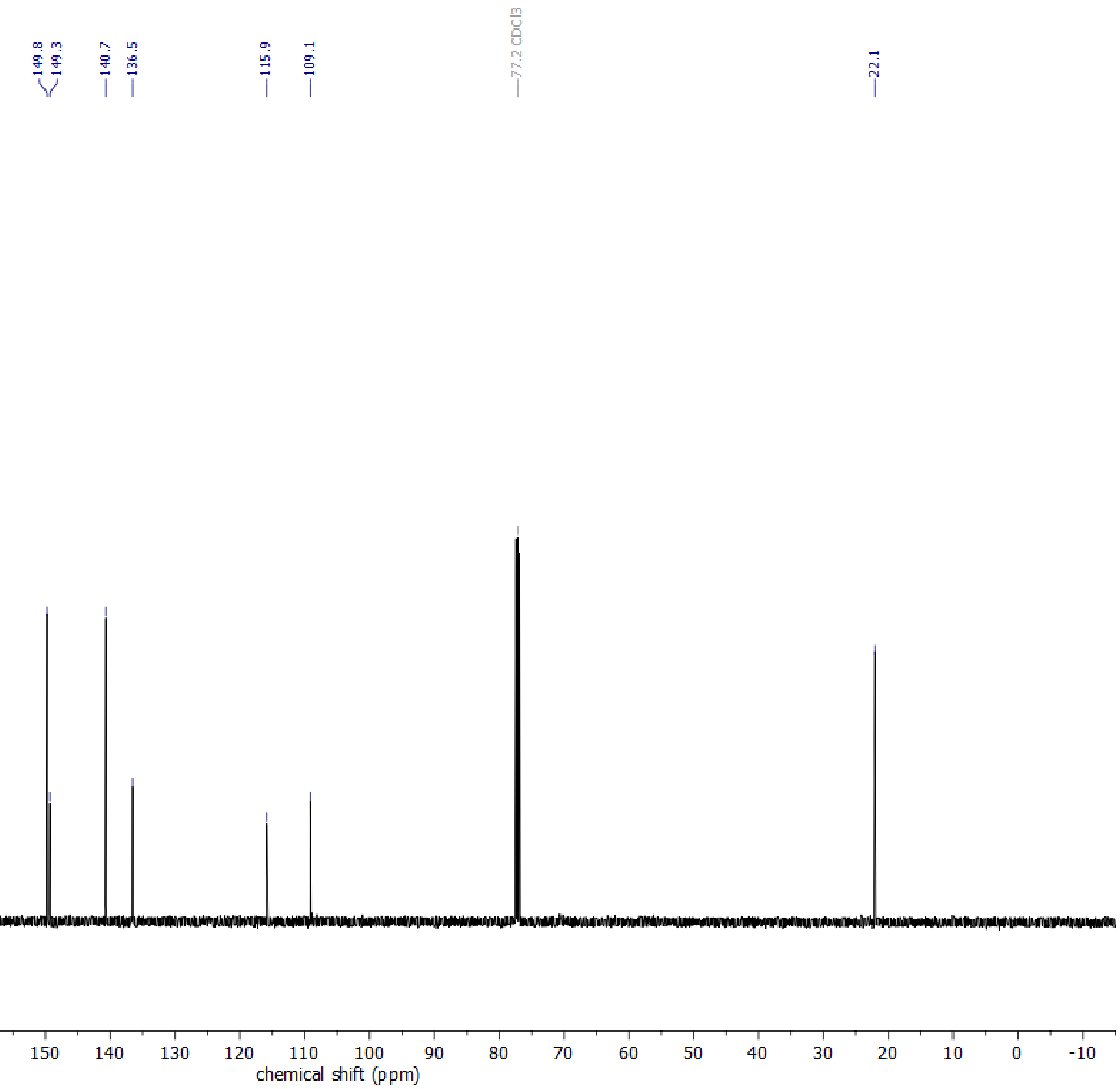

^{1}H NMR spectrum of 5-fluoro-2-iodobenzonitrile (6)

$CDCl_3$, 25°C, 500 MHz

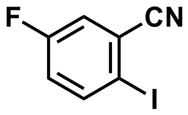

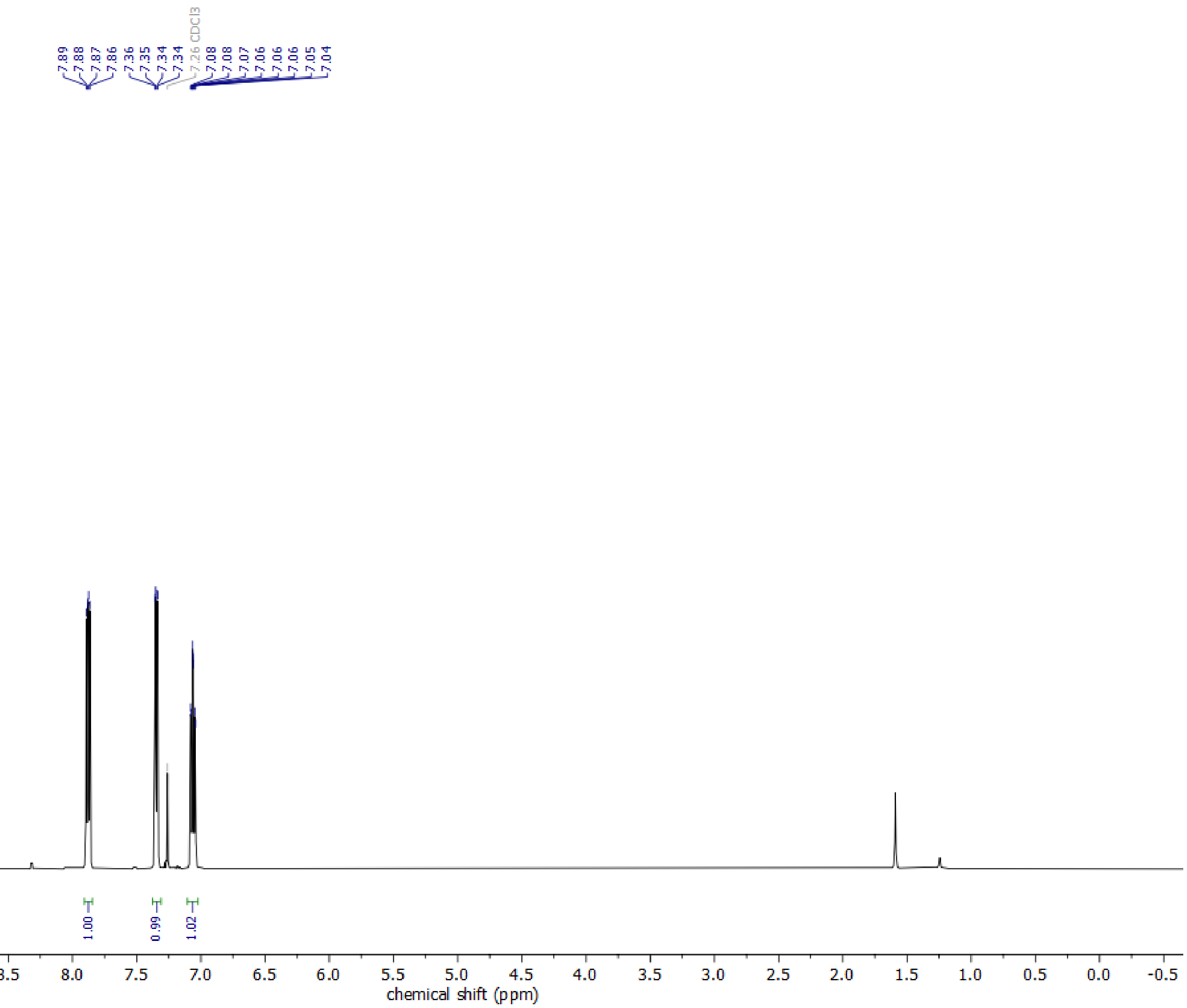

^{19}F NMR spectrum of 5-fluoro-2-iodobenzonitrile (6)

$CDCl_3$, 25°C, 470 MHz

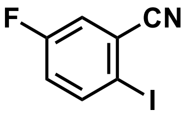

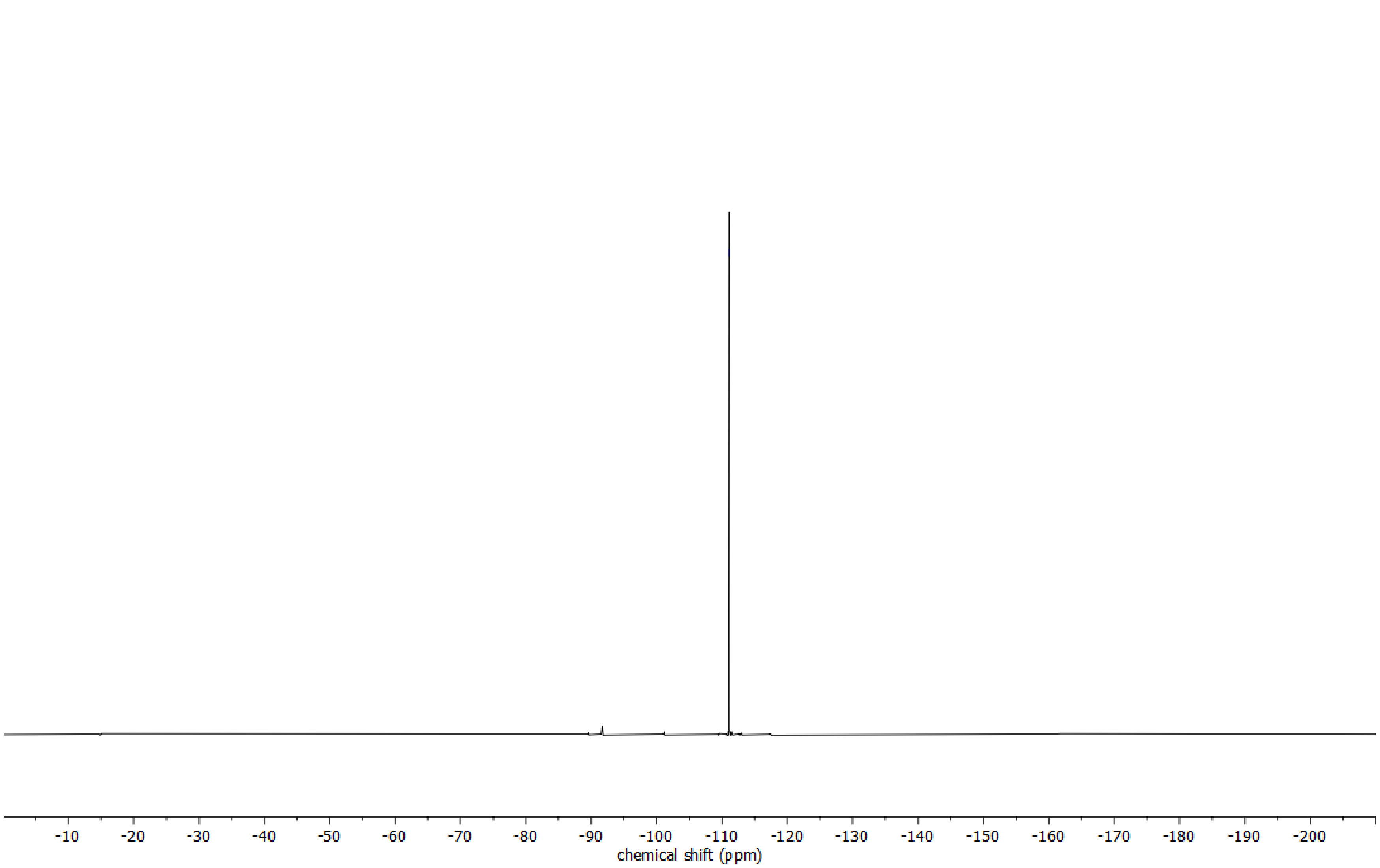

^{13}C NMR spectrum of 5-fluoro-2-iodobenzonitrile (6)

CDCl$_3$, 25°C, 125 MHz

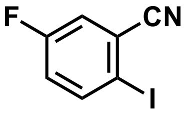

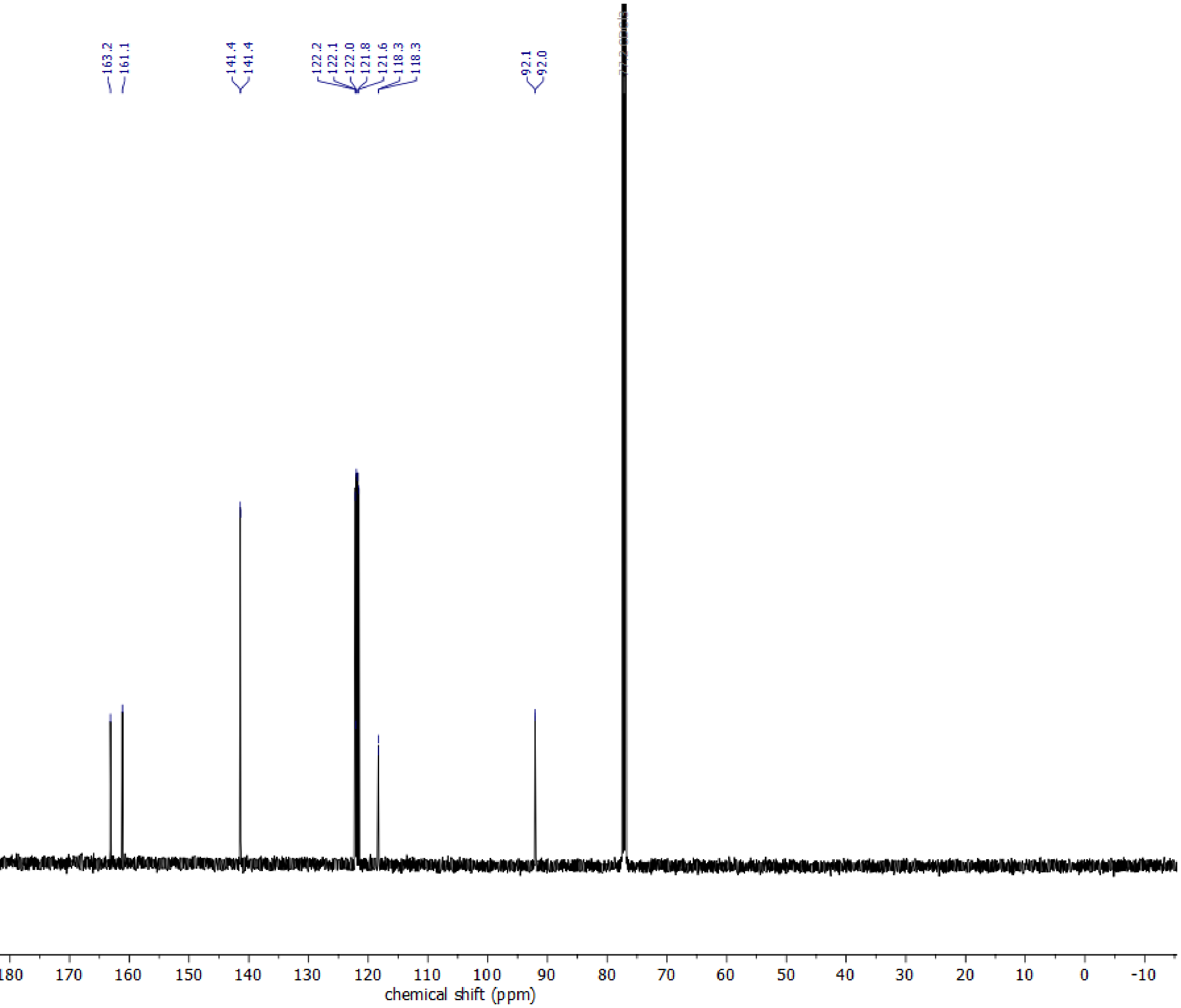

^{1}H NMR spectrum of 4-benzoylbenzonitrile (7)

$CDCl_3$, 25°C, 500 MHz

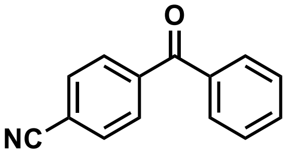

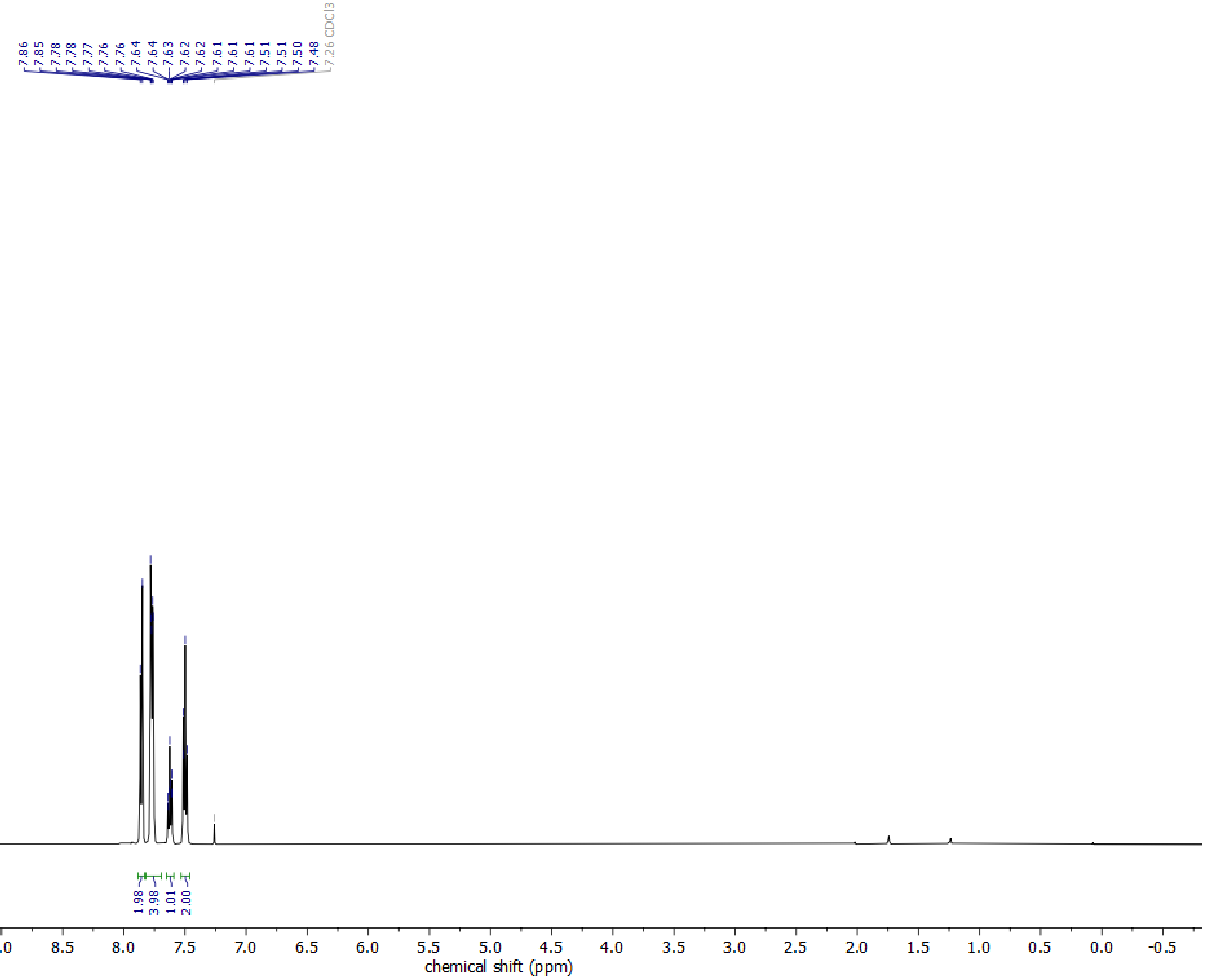

^{13}C NMR spectrum of 4-benzoylbenzonitrile (7)

$CDCl_3$, 25°C, 125 MHz

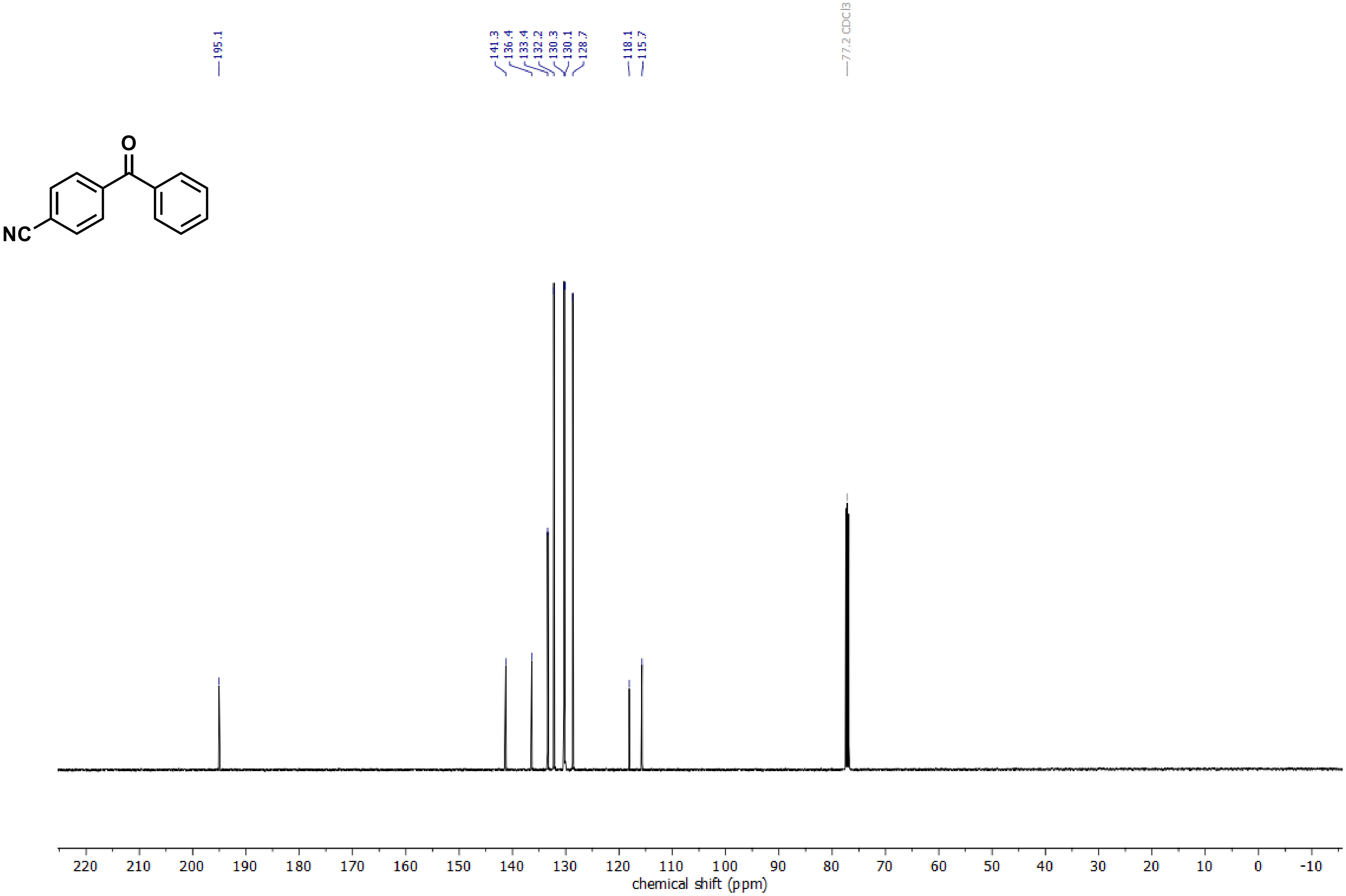

^{1}H NMR spectrum of 2,2-difluorobenzo[d][1,3]dioxole-5-carbonitrile (8)

$CDCl_3$, 25°C, 500 MHz

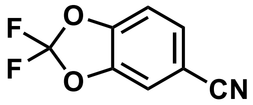

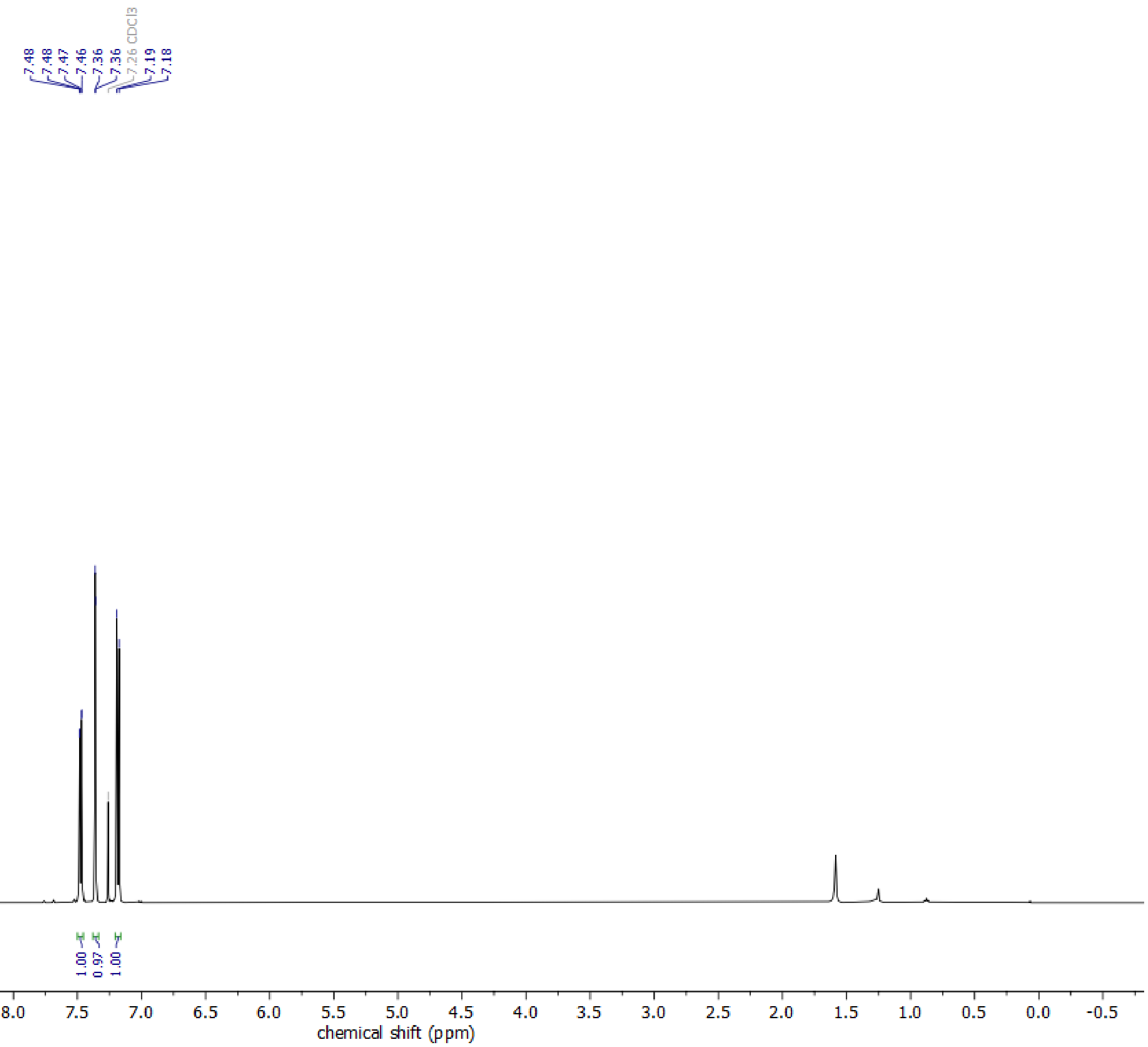

^{19}F NMR spectrum of 2,2-difluorobenzo[d][1,3]dioxole-5-carbonitrile (8)

$CDCl_3$, 25°C, 470 MHz

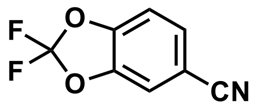

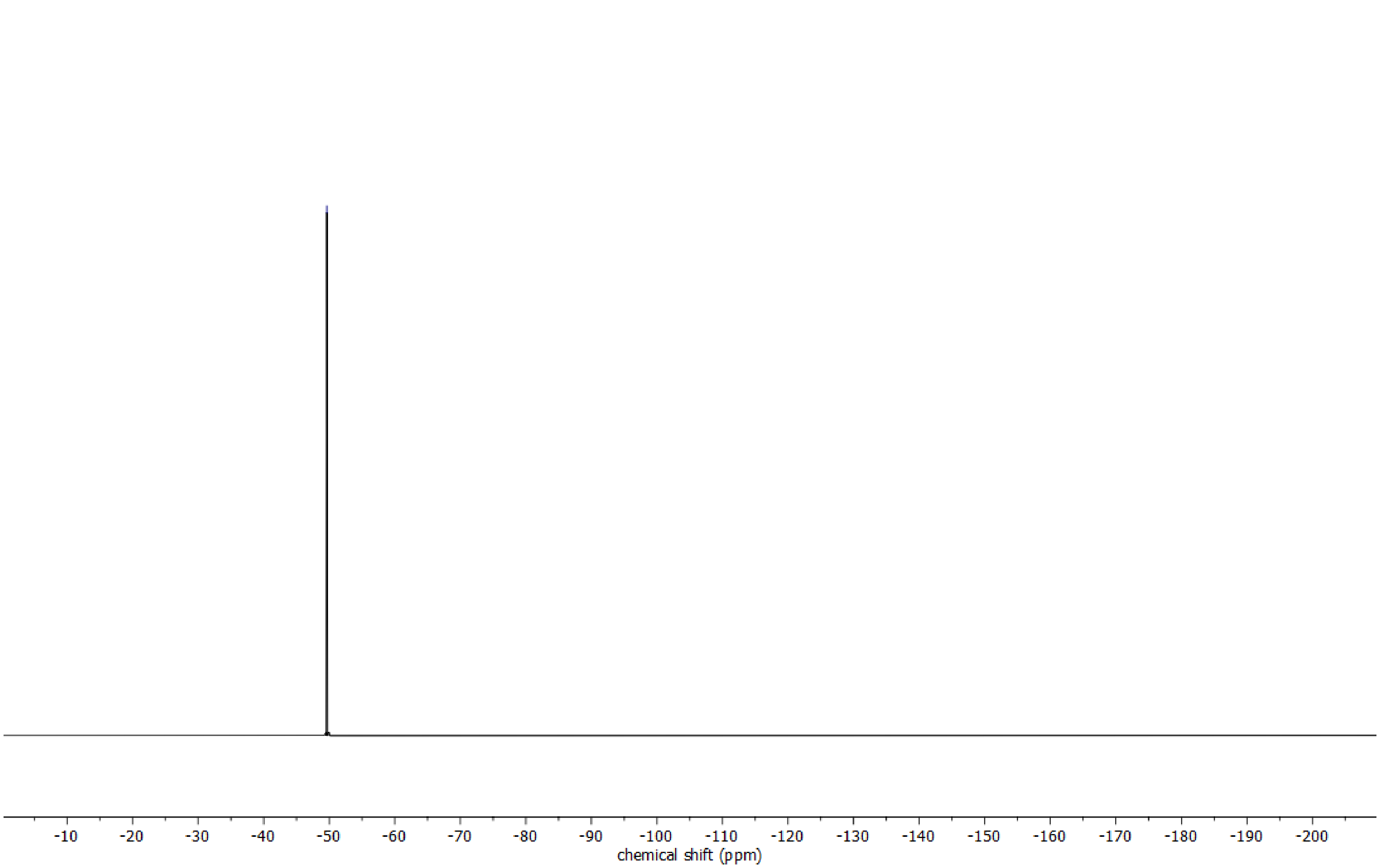

^{13}C NMR spectrum of 2,2-difluorobenzo[d][1,3]dioxole-5-carbonitrile (8)

$CDCl_3$, 25°C, 125 MHz

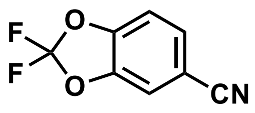

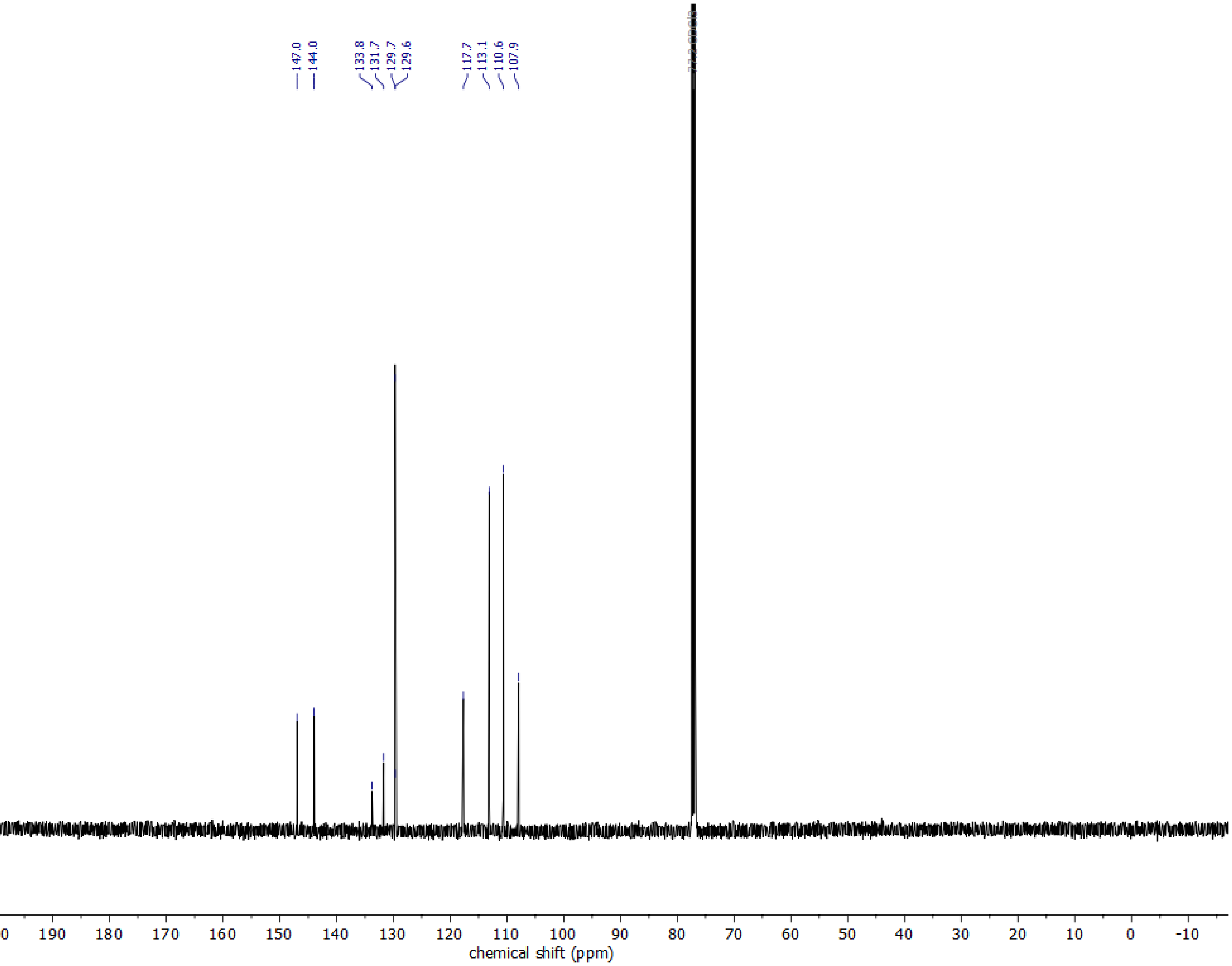

^{1}H NMR spectrum of 4'-chloro-[1,1'-biphenyl]-2-carbonitrile (9)

$CDCl_3$, 25°C, 500 MHz

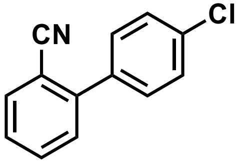

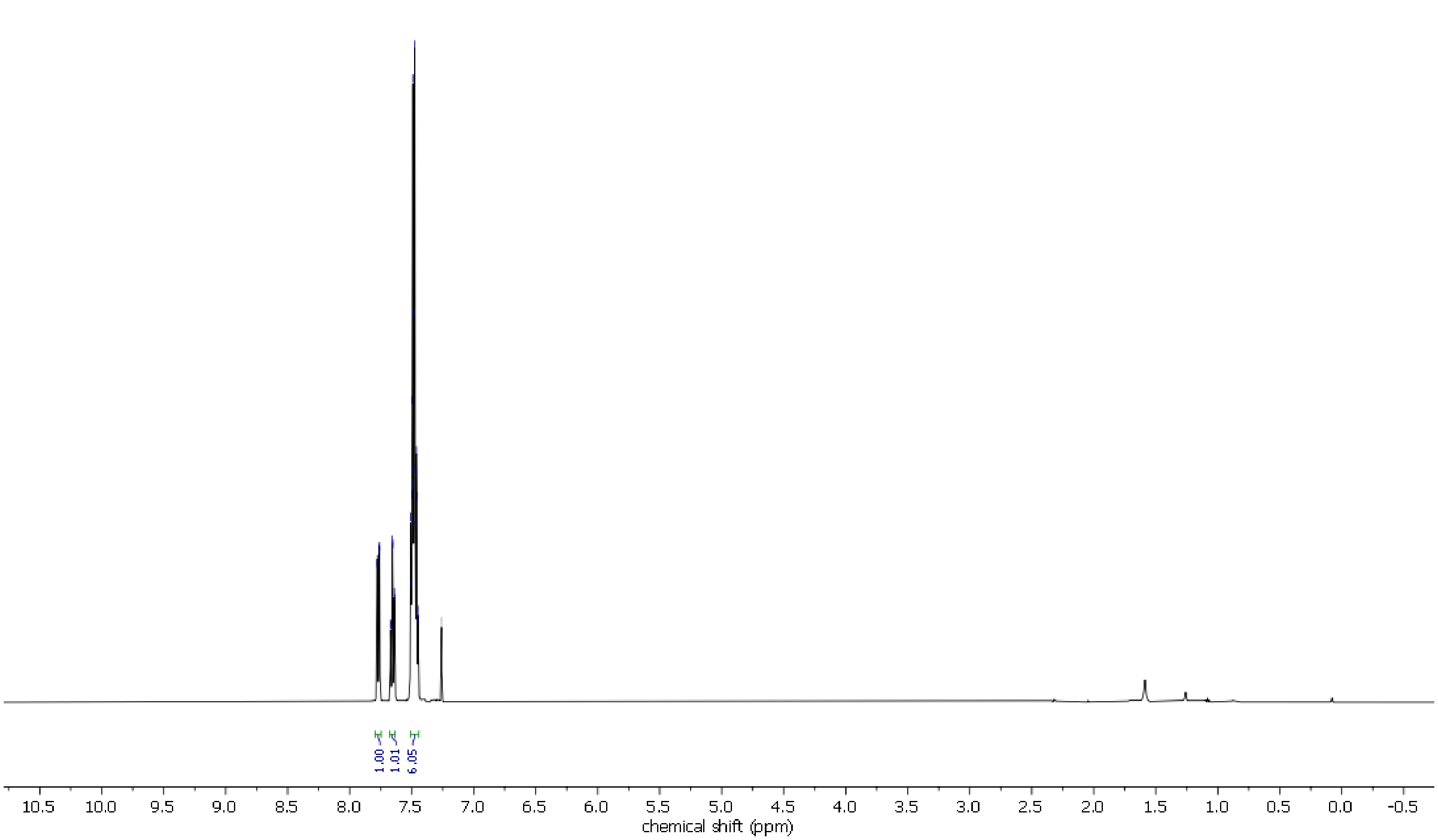

^{13}C NMR spectrum of 4'-chloro-[1,1'-biphenyl]-2-carbonitrile (9)

$CDCl_3$, 25°C, 125 MHz

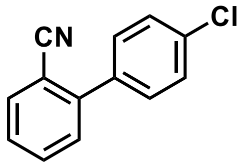

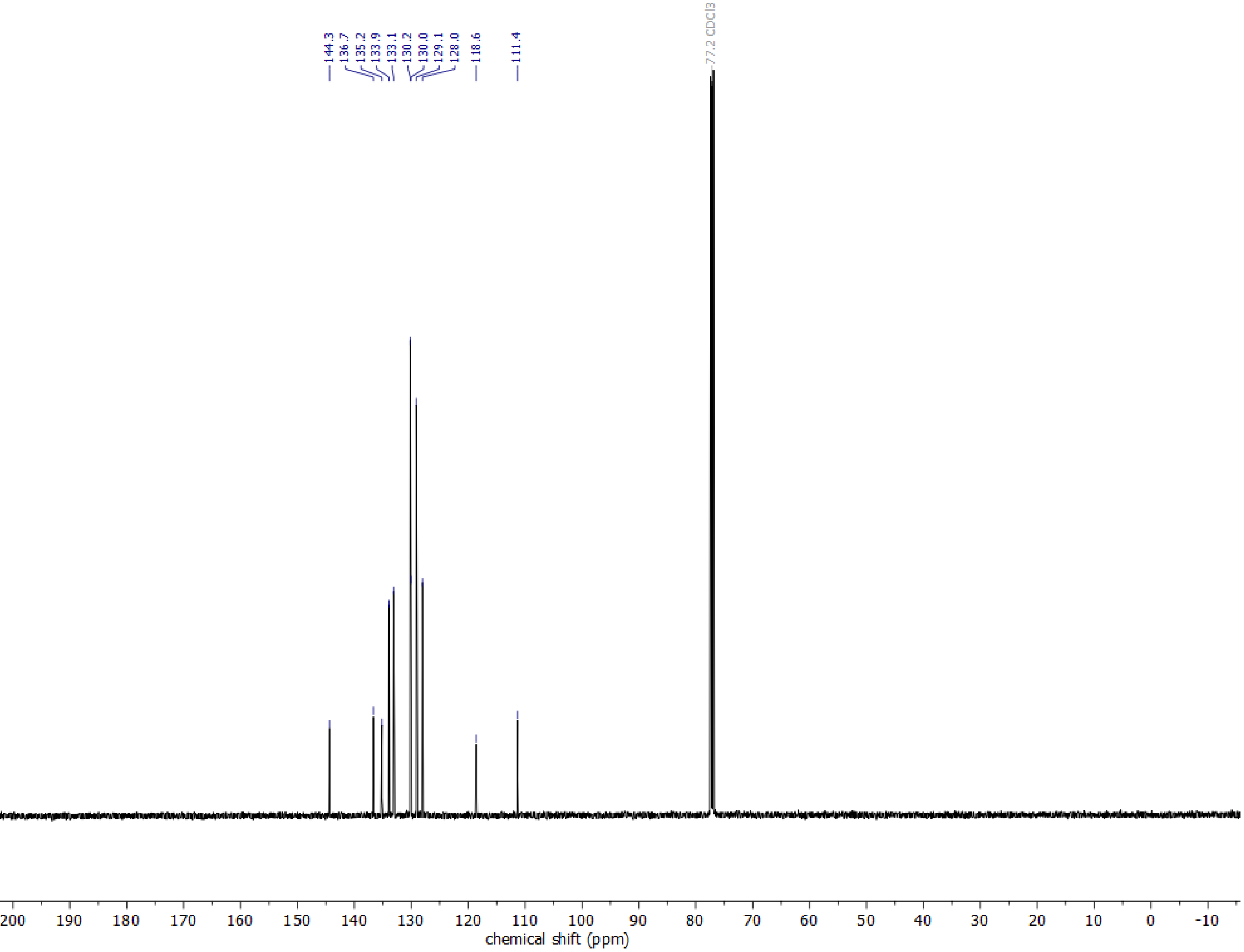

^{1}H NMR spectrum of 2-chloronicotinonitrile (10)

$CDCl_3$, 25°C, 500 MHz

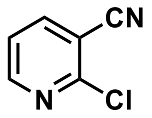

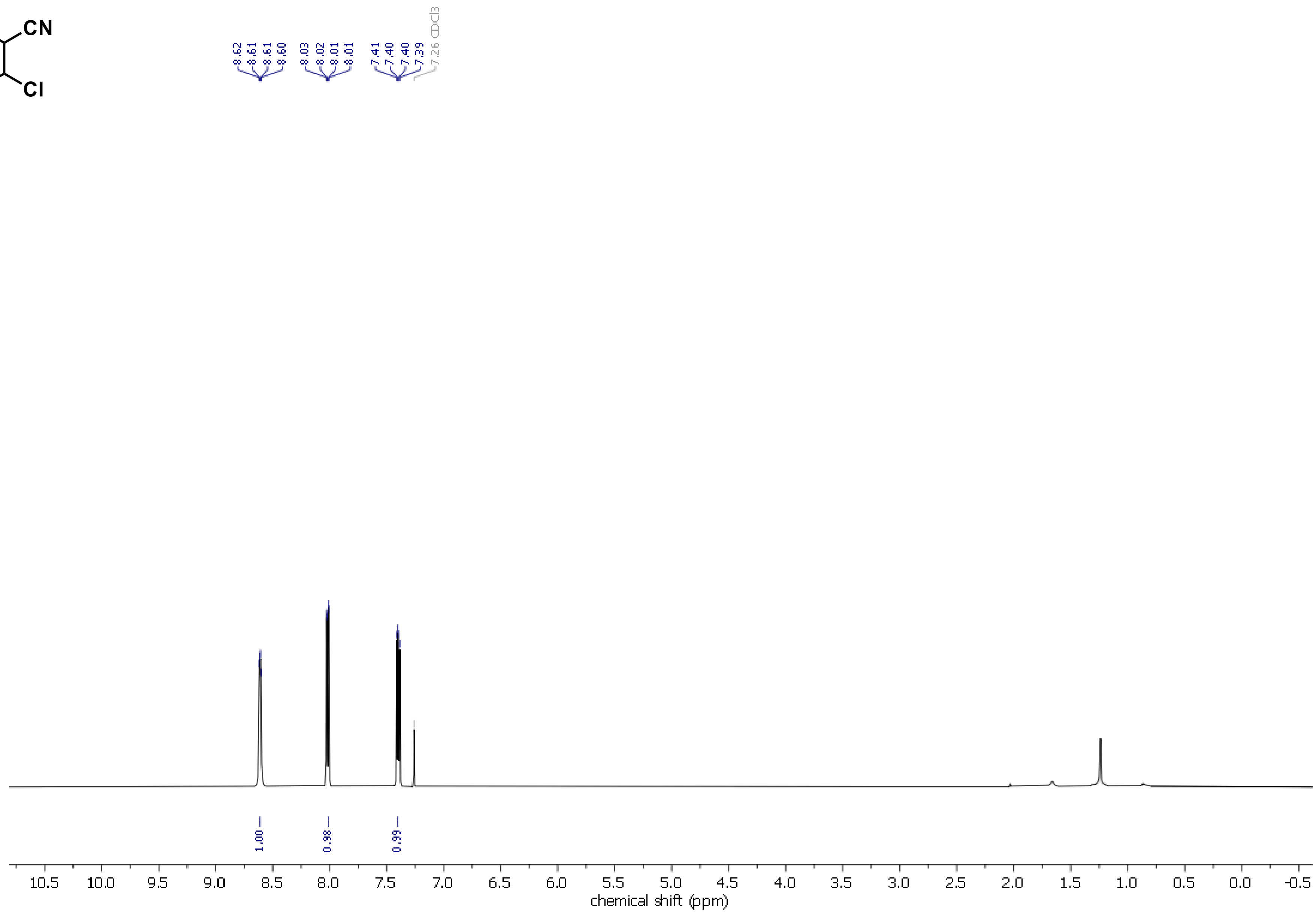

^{13}C NMR spectrum of 2-chloronicotinonitrile (10)

$CDCl_3$, 25°C, 125 MHz

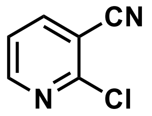

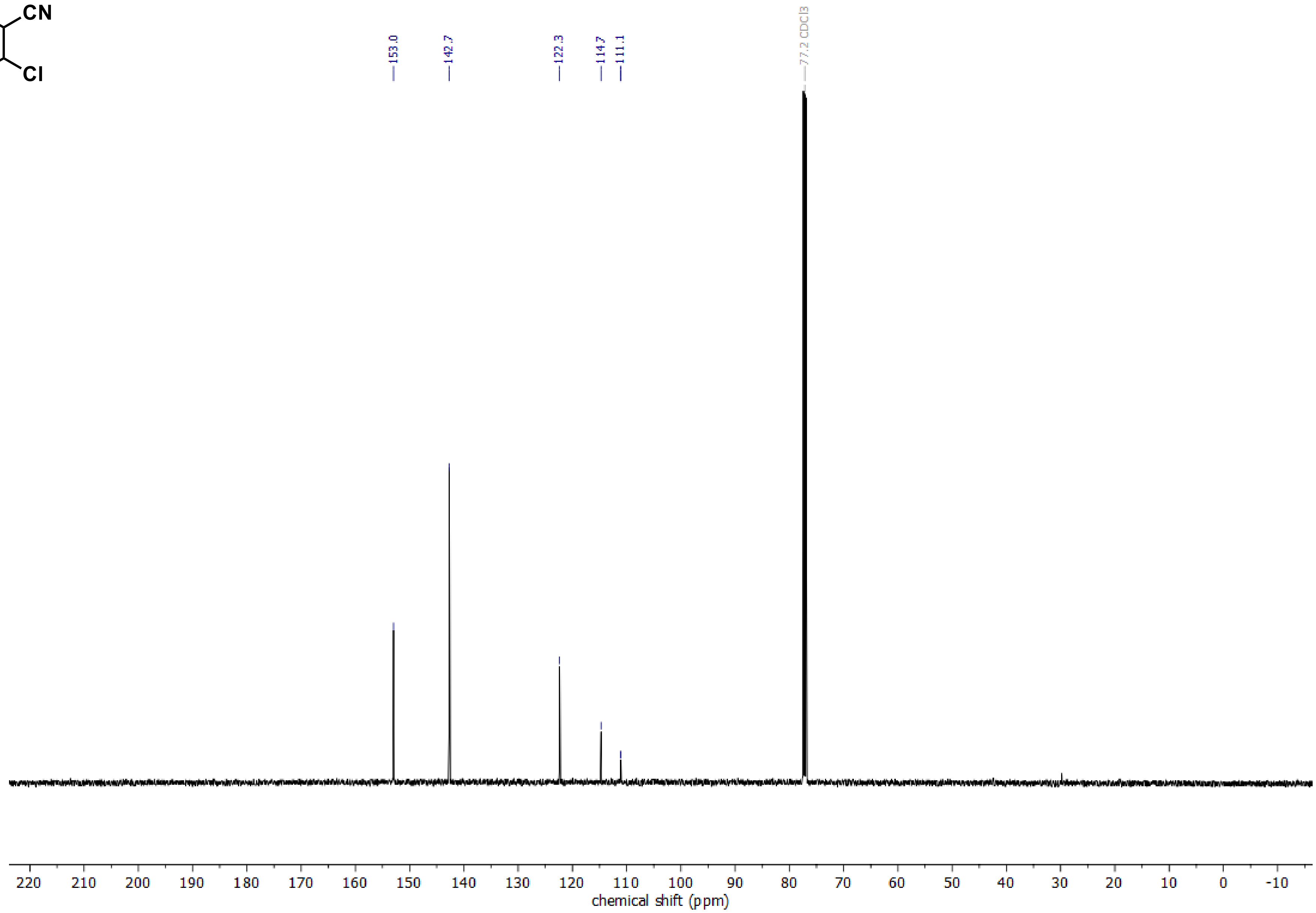

^{1}H NMR spectrum of 2,4,6-tribromobenzonitrile (11)

$CDCl_3$, 25°C, 500 MHz

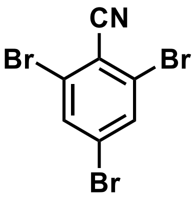

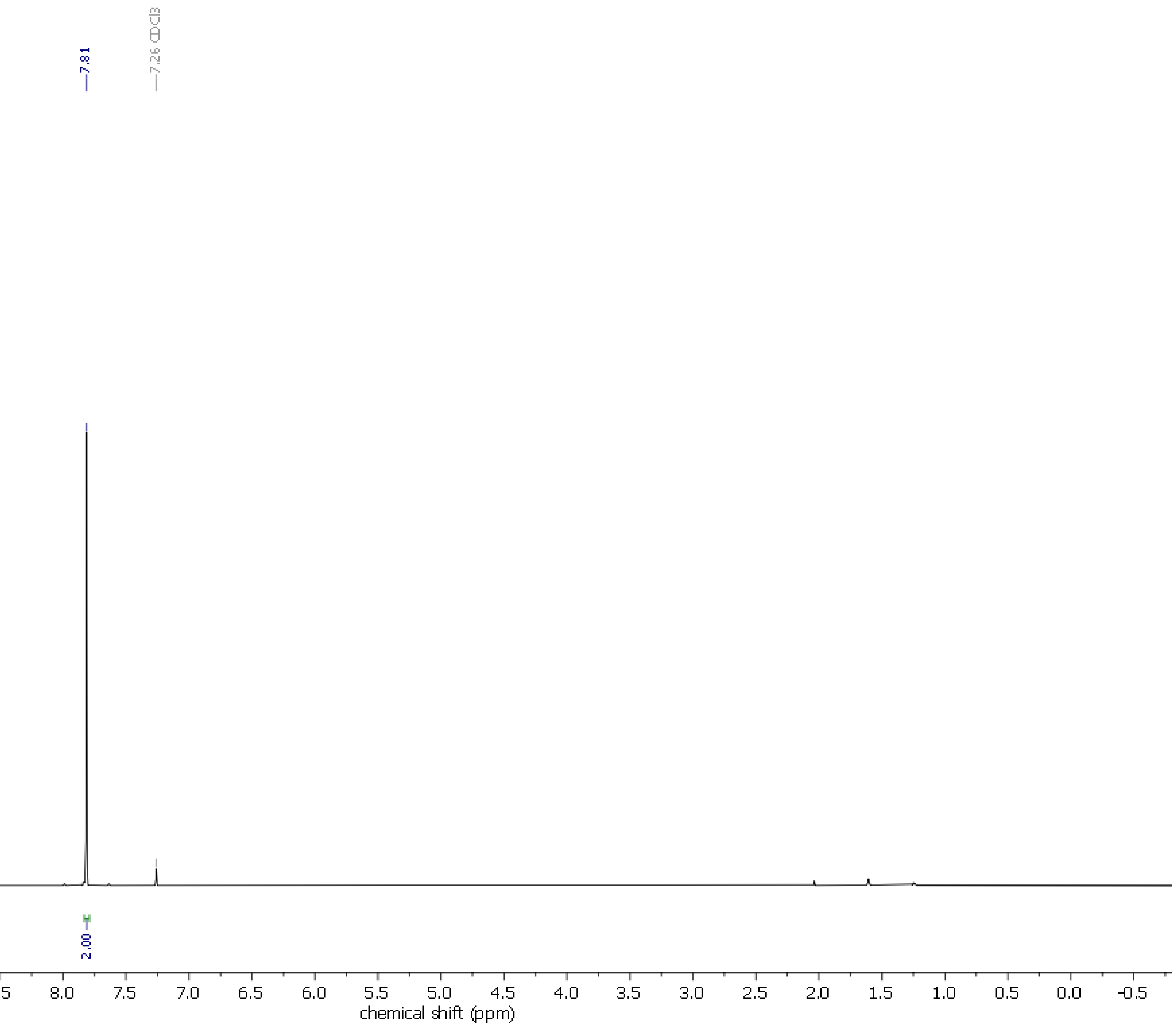

^{13}C NMR spectrum of 2,4,6-tribromobenzonitrile (11)

$CDCl_3$, 25°C, 125 MHz

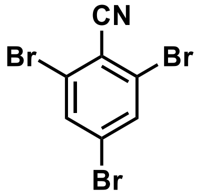

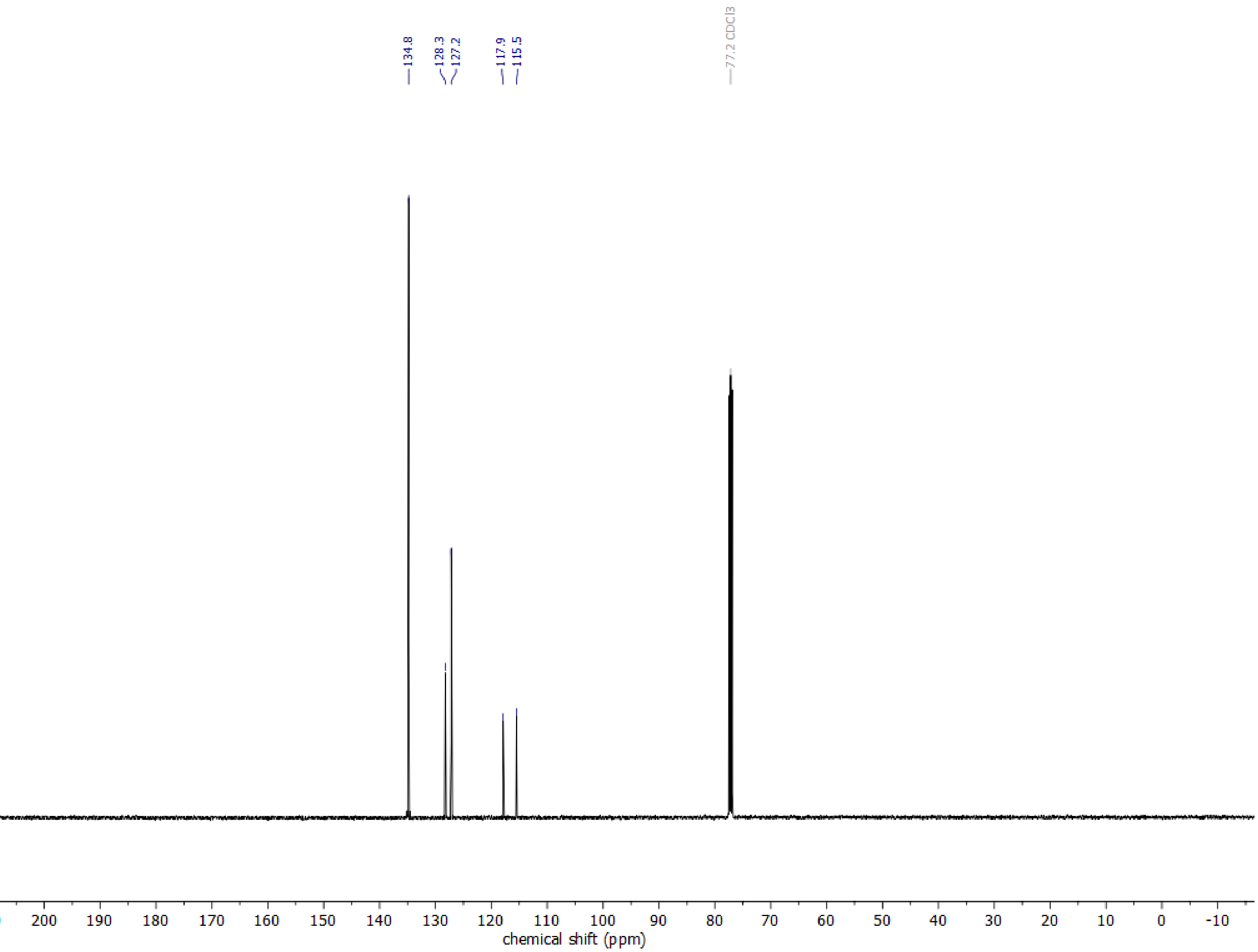

^{1}H NMR spectrum of 2,6-dimethylterephthalonitrile (12)

$CDCl_3$, 25°C, 500 MHz

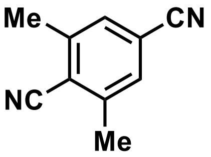

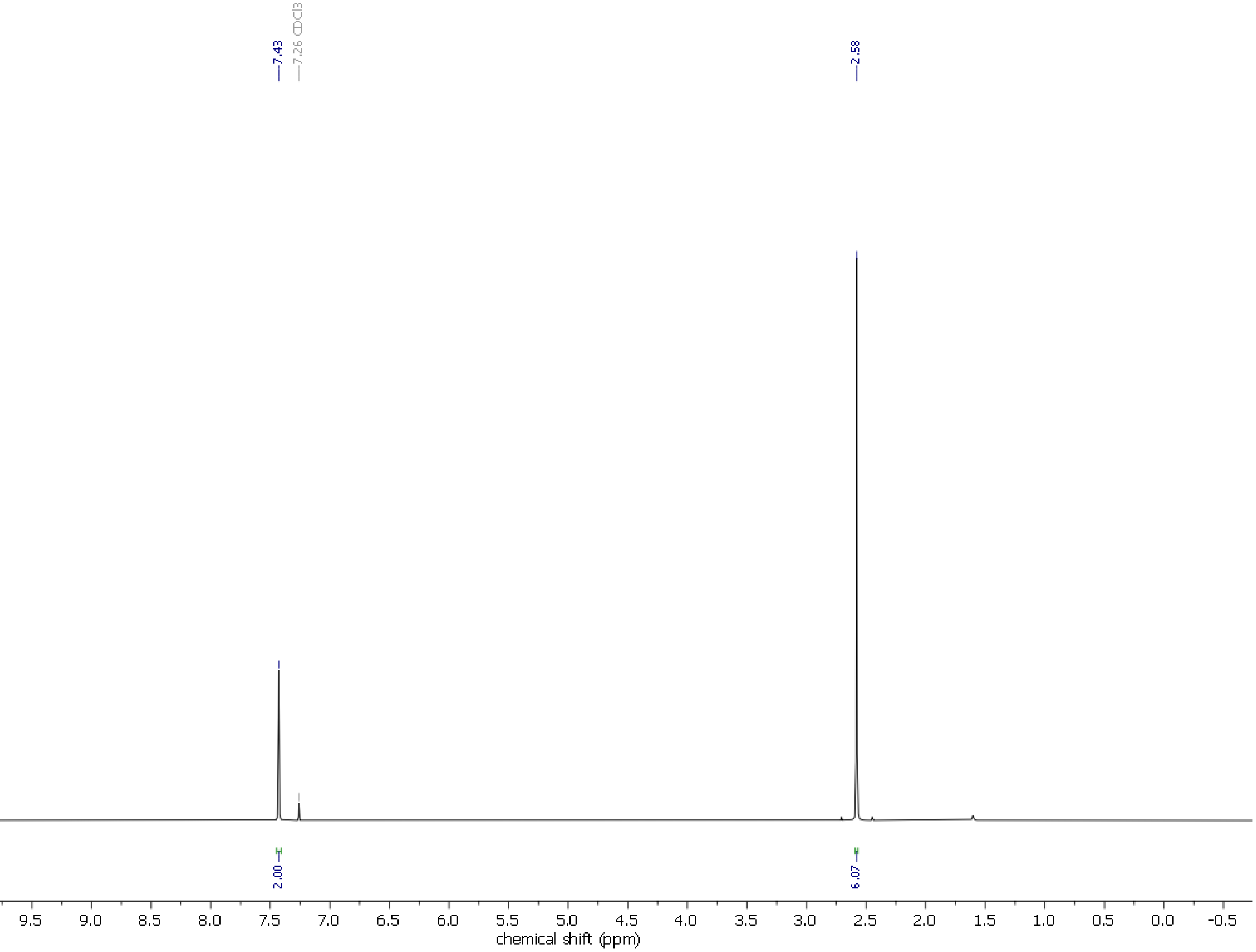

^{13}C NMR spectrum of 2,6-dimethylterephthalonitrile (12)

$CDCl_3$, 25°C, 125 MHz

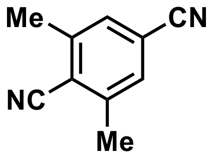

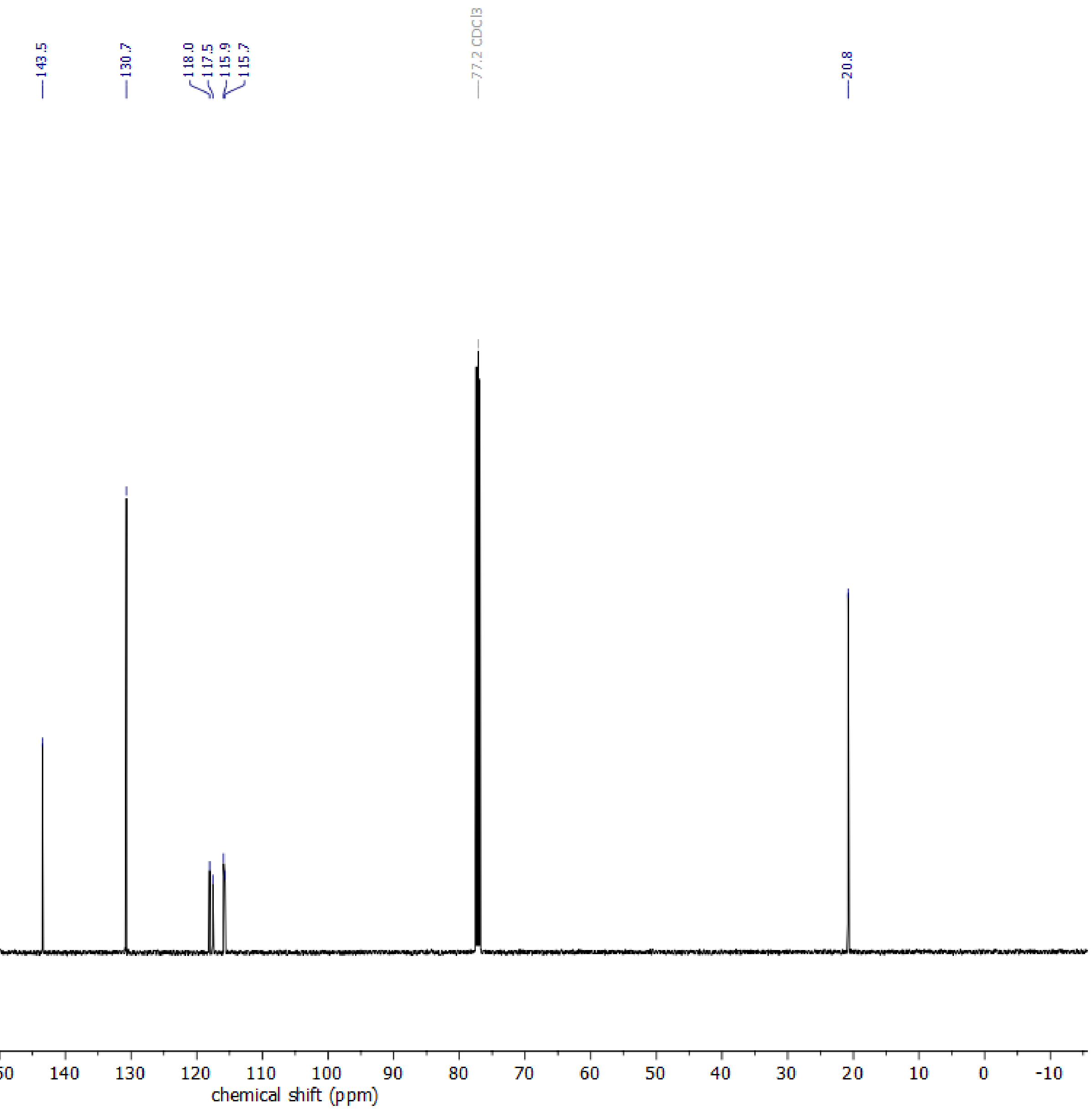

^{1}H NMR spectrum of terephthalonitrile (13)

$CDCl_3$, 25°C, 500 MHz

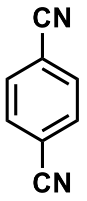

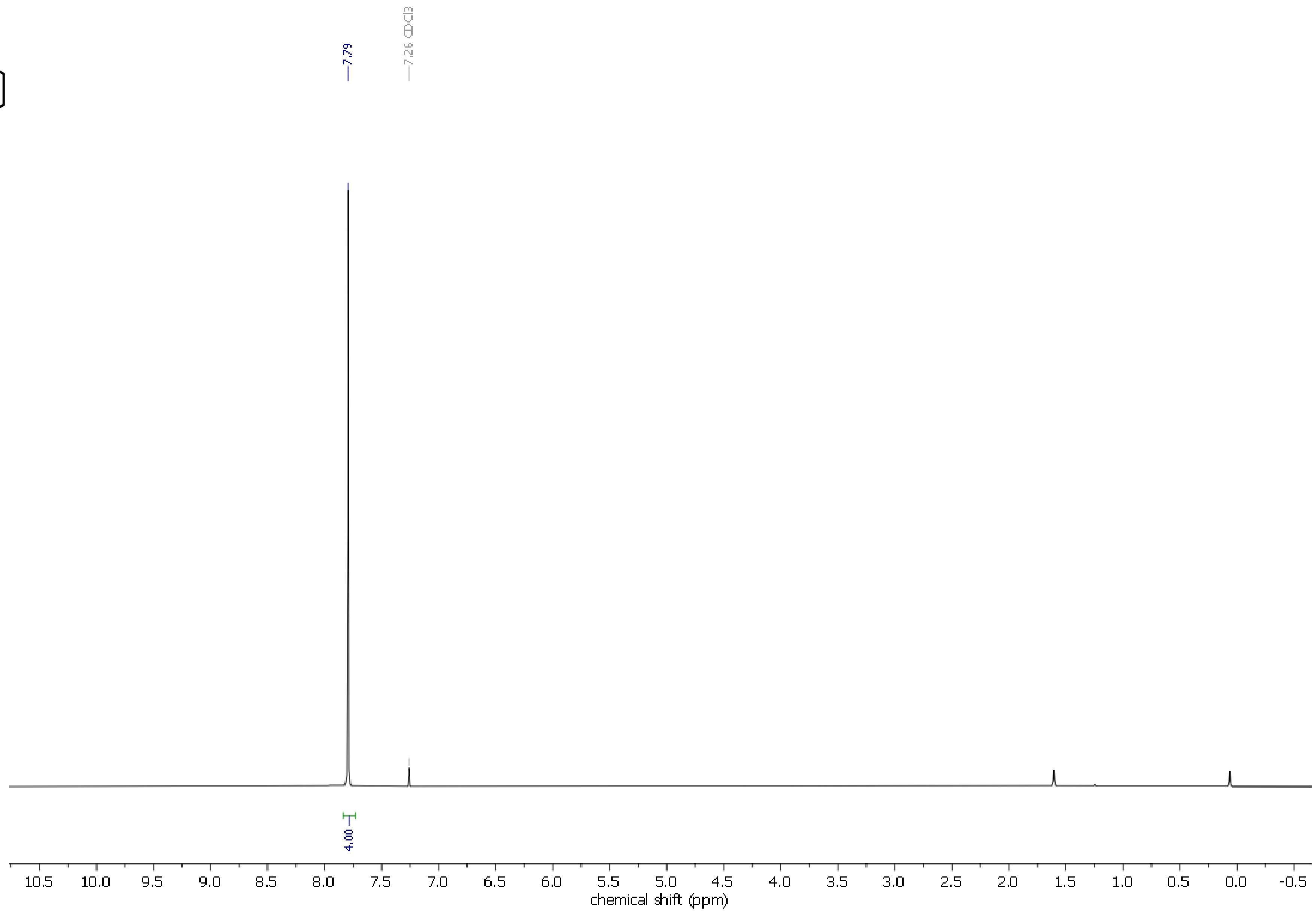

^{13}C NMR spectrum of terephthalonitrile (13)

$CDCl_3$, 25°C, 125 MHz

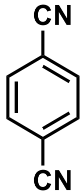

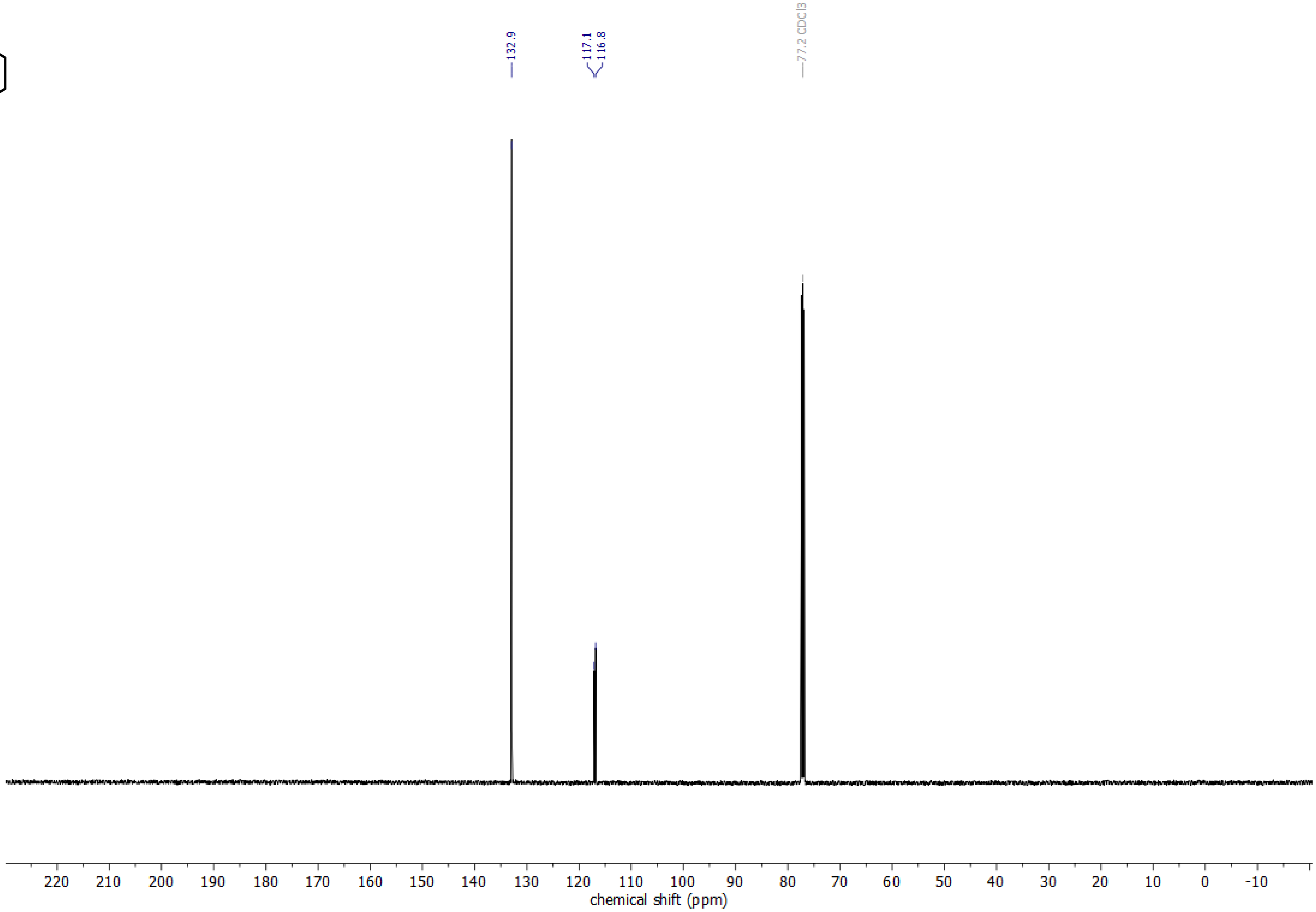

^{1}H NMR spectrum of Flutamide-derived benzonitrile 14

DMSO-d_6, 25°C, 500 MHz

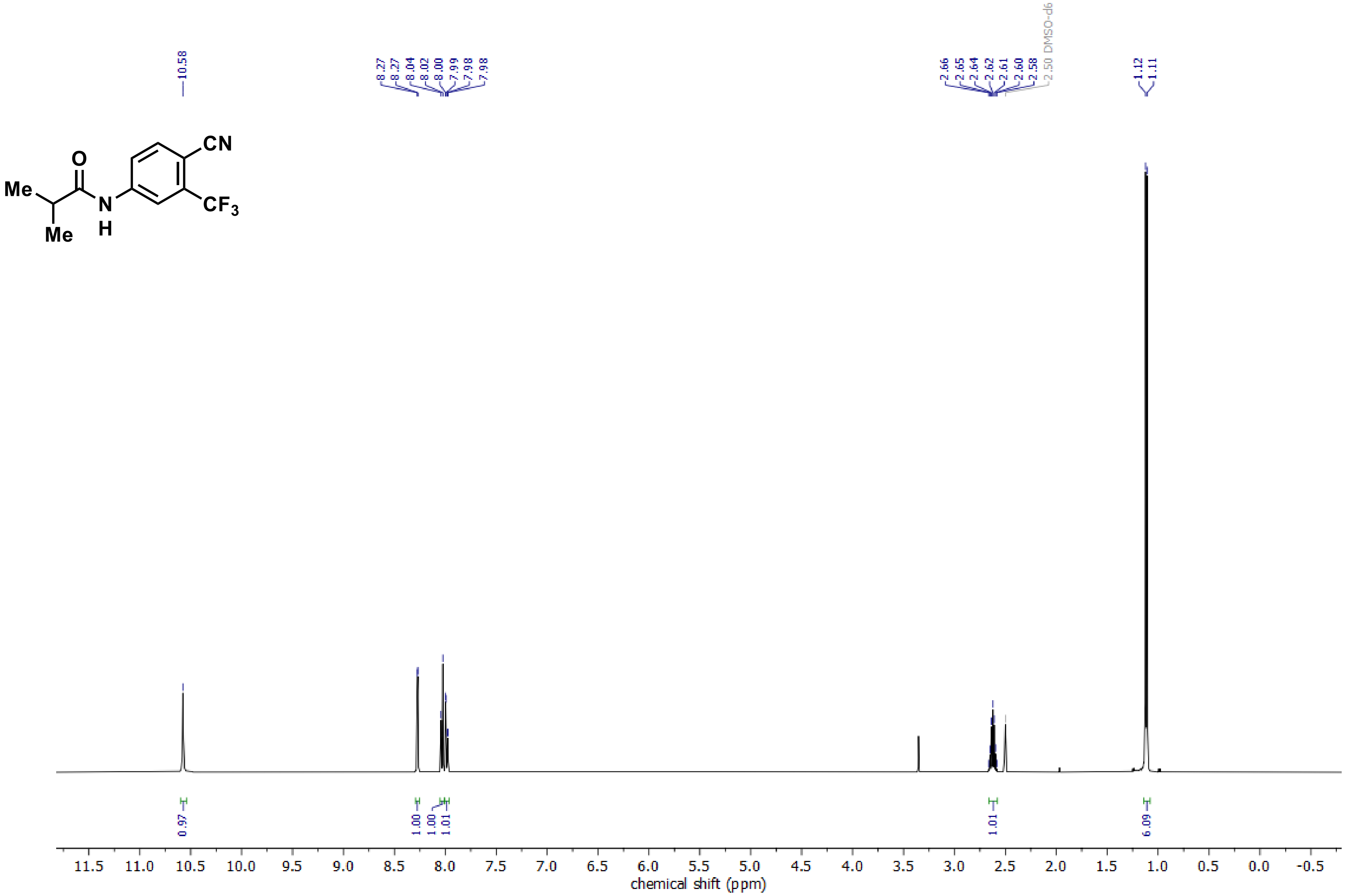

^{19}F NMR spectrum of Flutamide-derived benzonitrile 14

DMSO-d_6, 25°C, 470 MHz

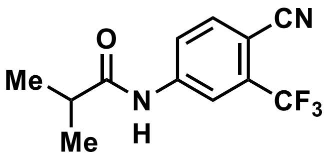

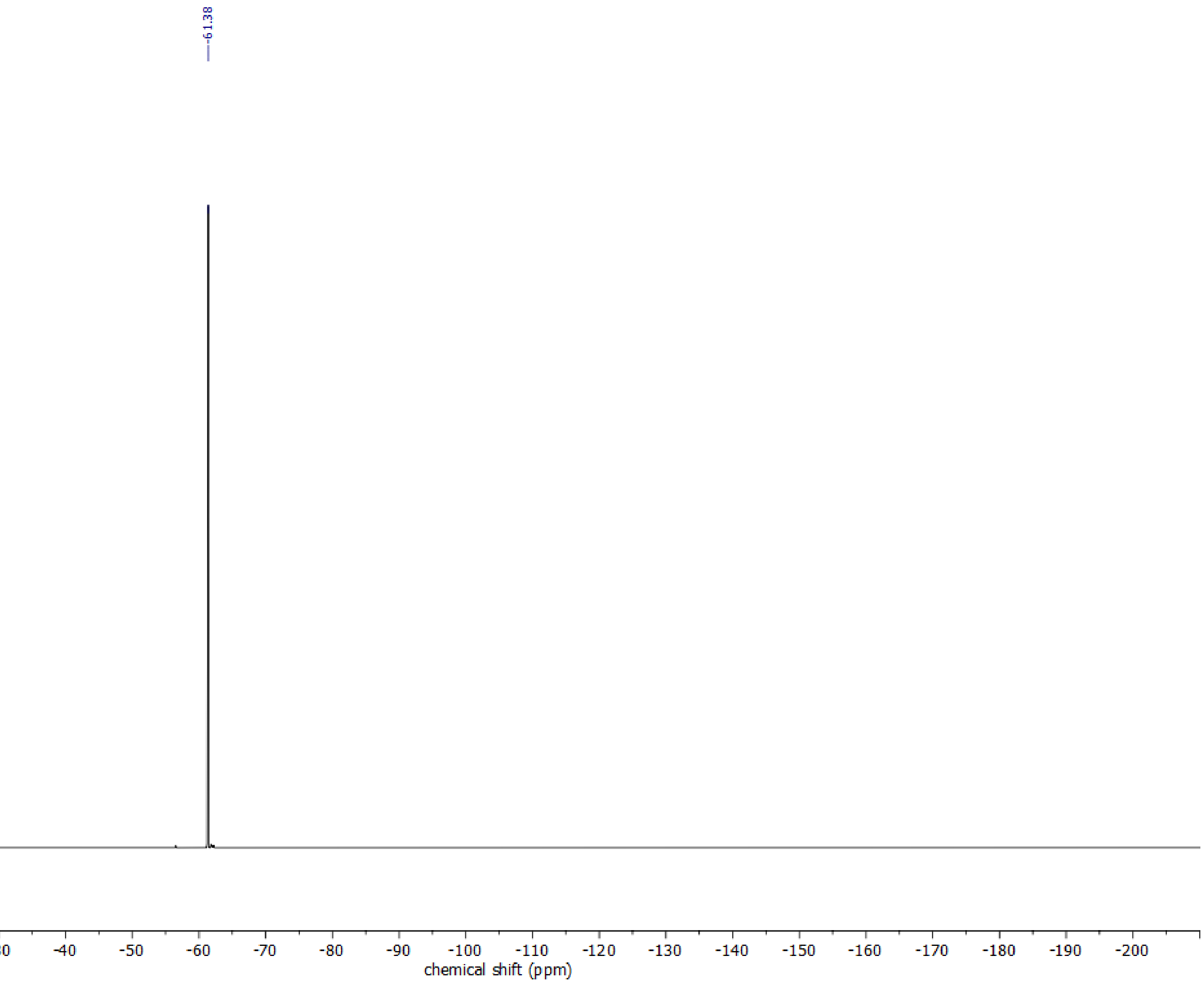

^{13}C NMR spectrum of Flutamide-derived benzonitrile 14

DMSO-d_6, 25°C, 125 MHz

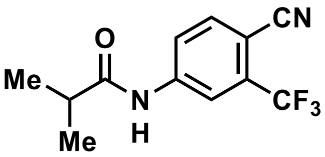

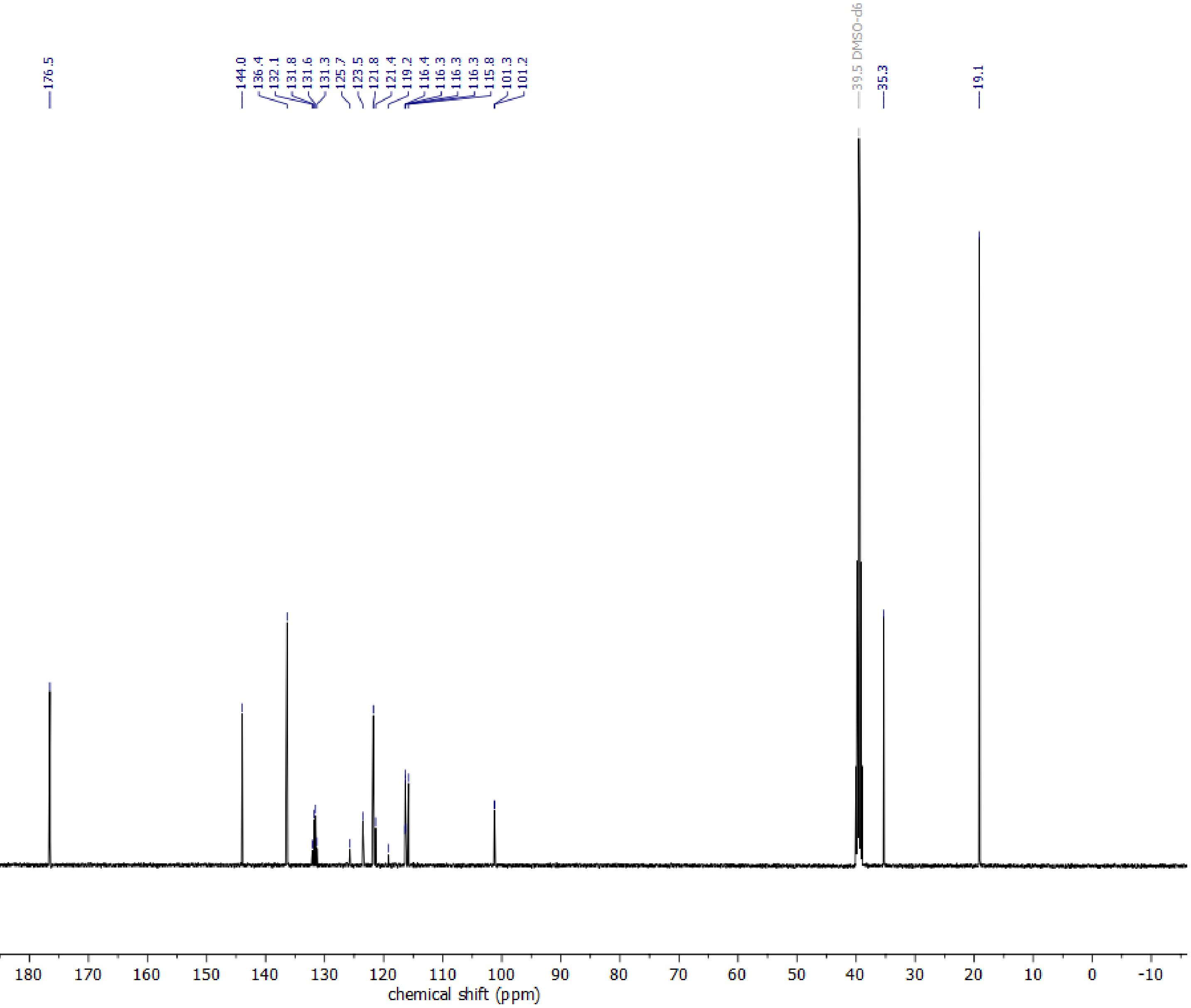

^{1}H NMR spectrum of 2,6-dichloro-3-methylbenzonitrile (15)

$CDCl_3$, 25°C, 500 MHz

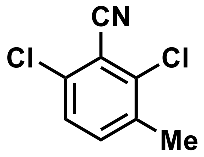

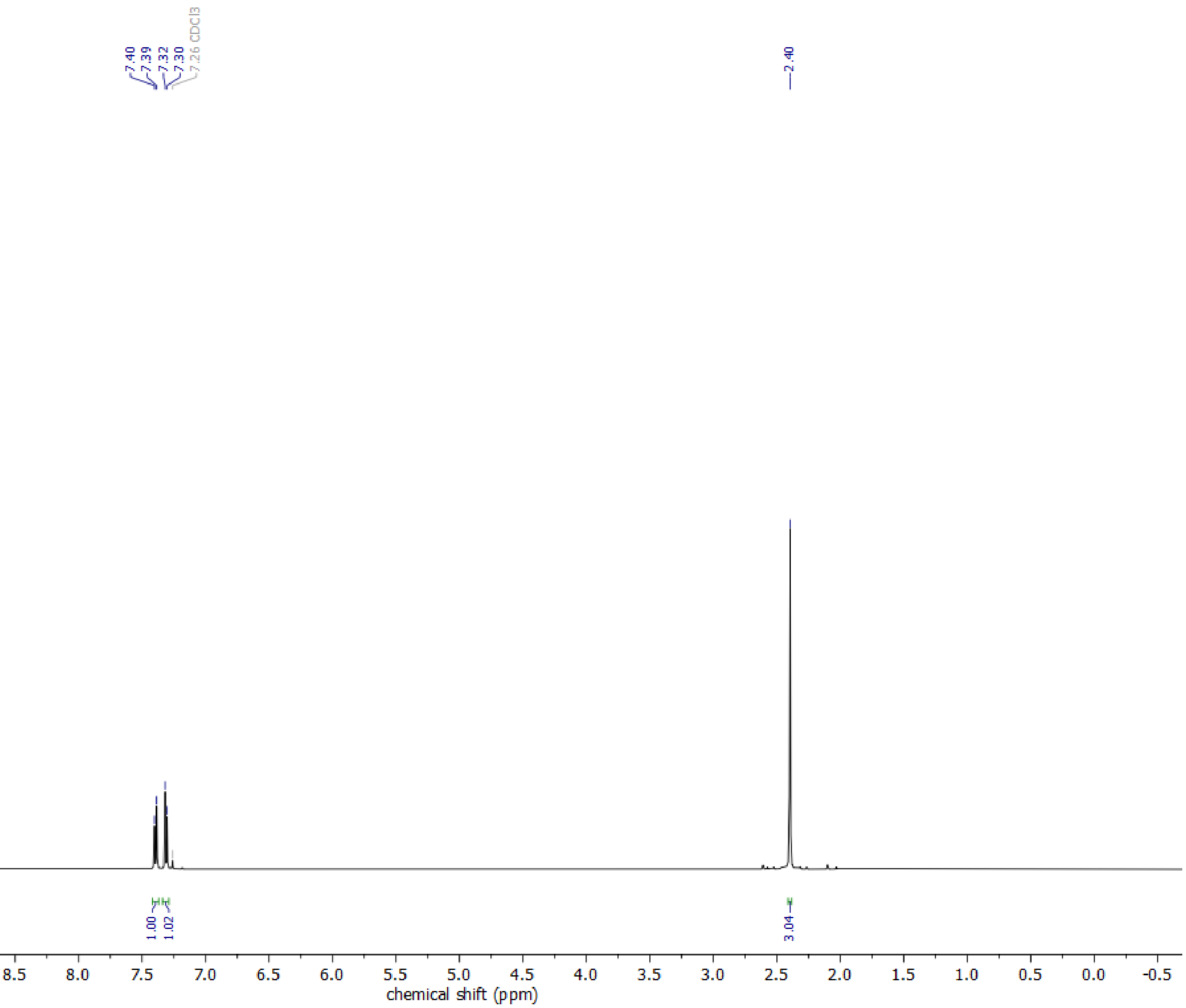

^{13}C NMR spectrum of 2,6-dichloro-3-methylbenzonitrile (15)

$CDCl_3$, 25°C, 125 MHz

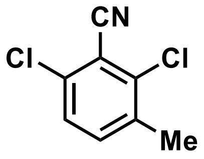

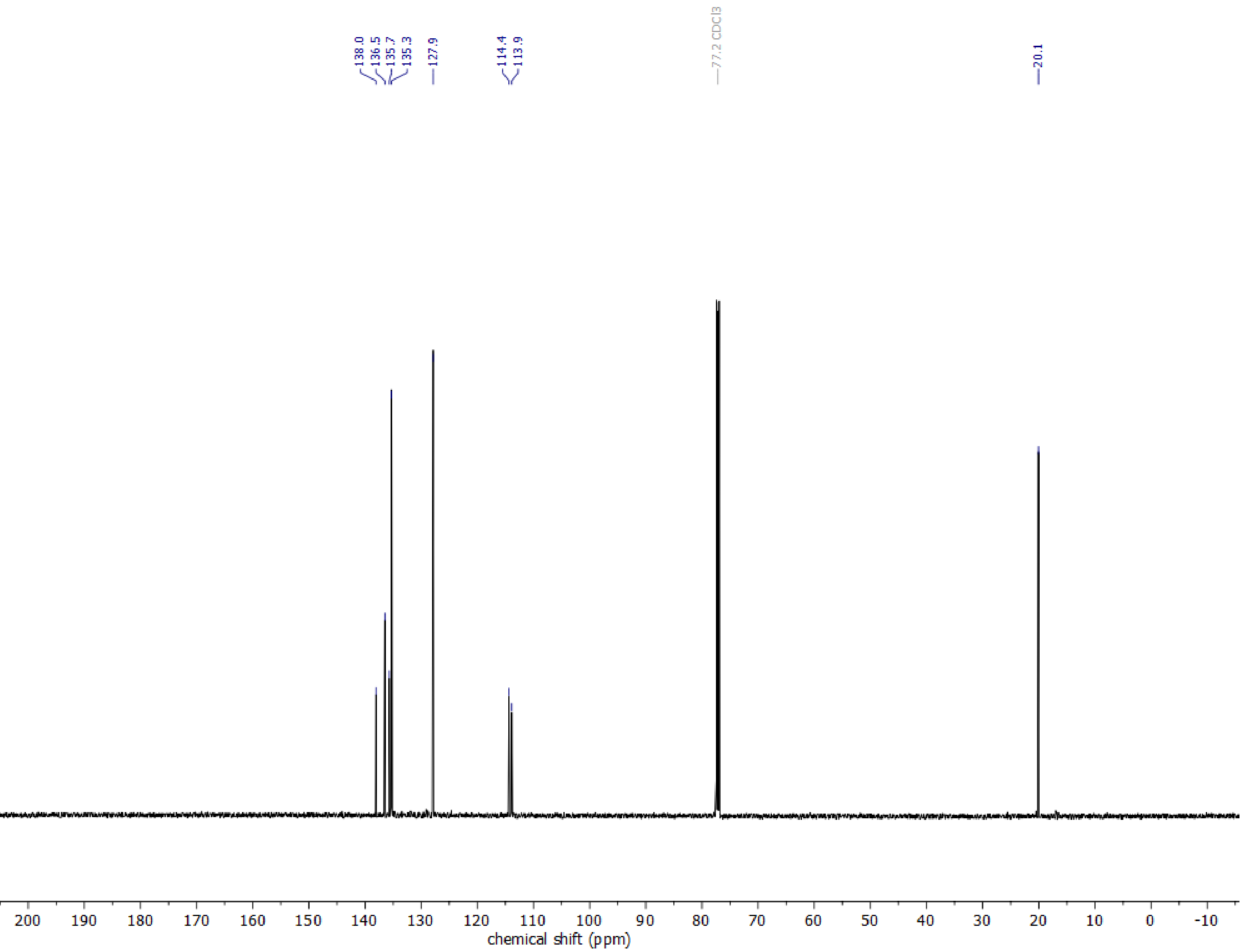

^{1}H NMR spectrum of ethyl 4-cyanobenzoate (16)

$CDCl_3$, 25°C, 500 MHz

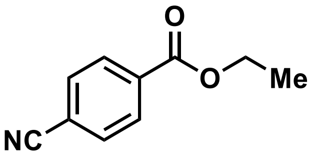

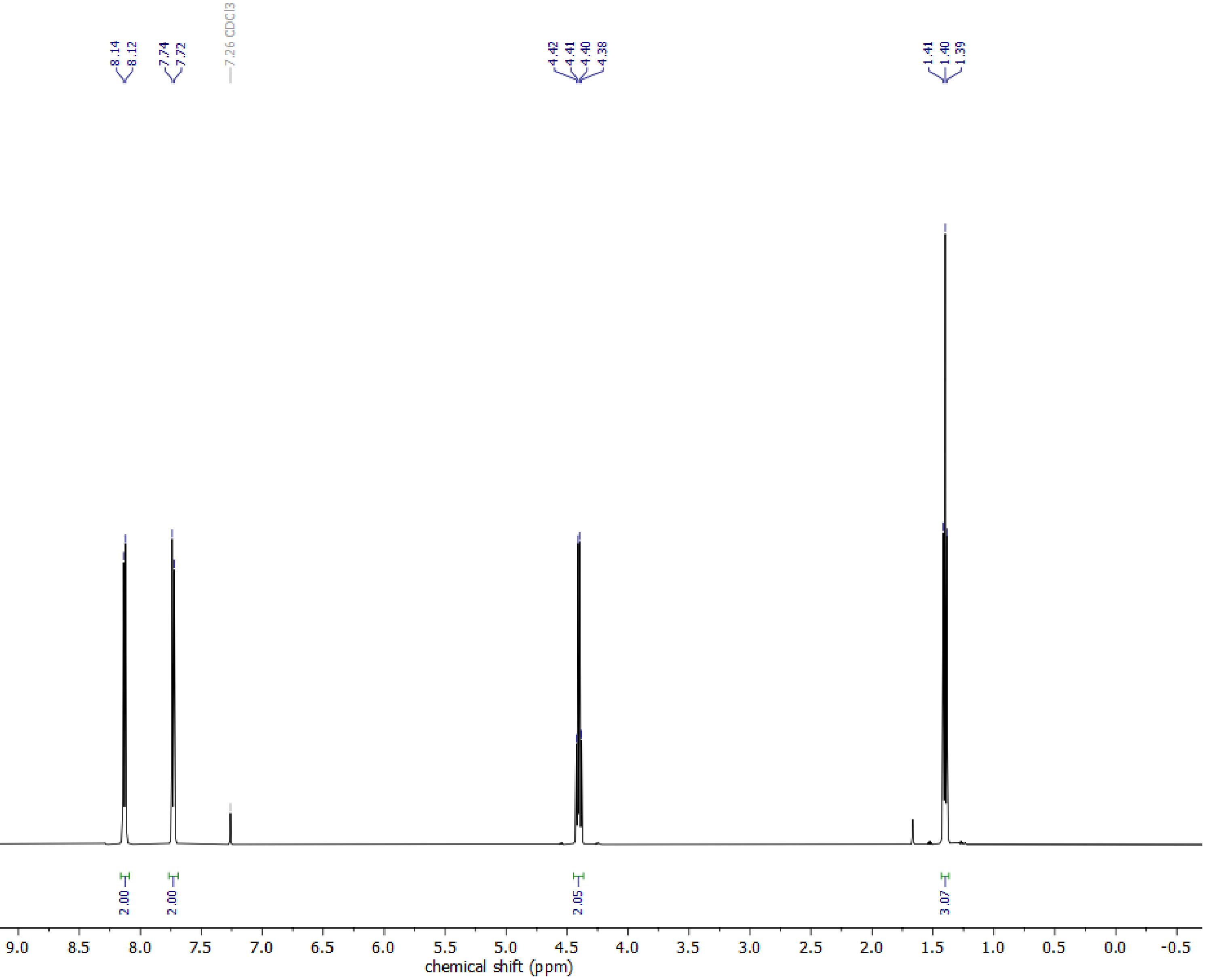

^{13}C NMR spectrum of ethyl 4-cyanobenzoate (16)

$CDCl_3$, 25°C, 125 MHz

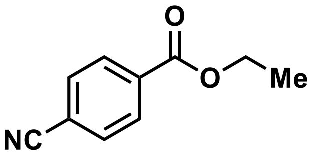

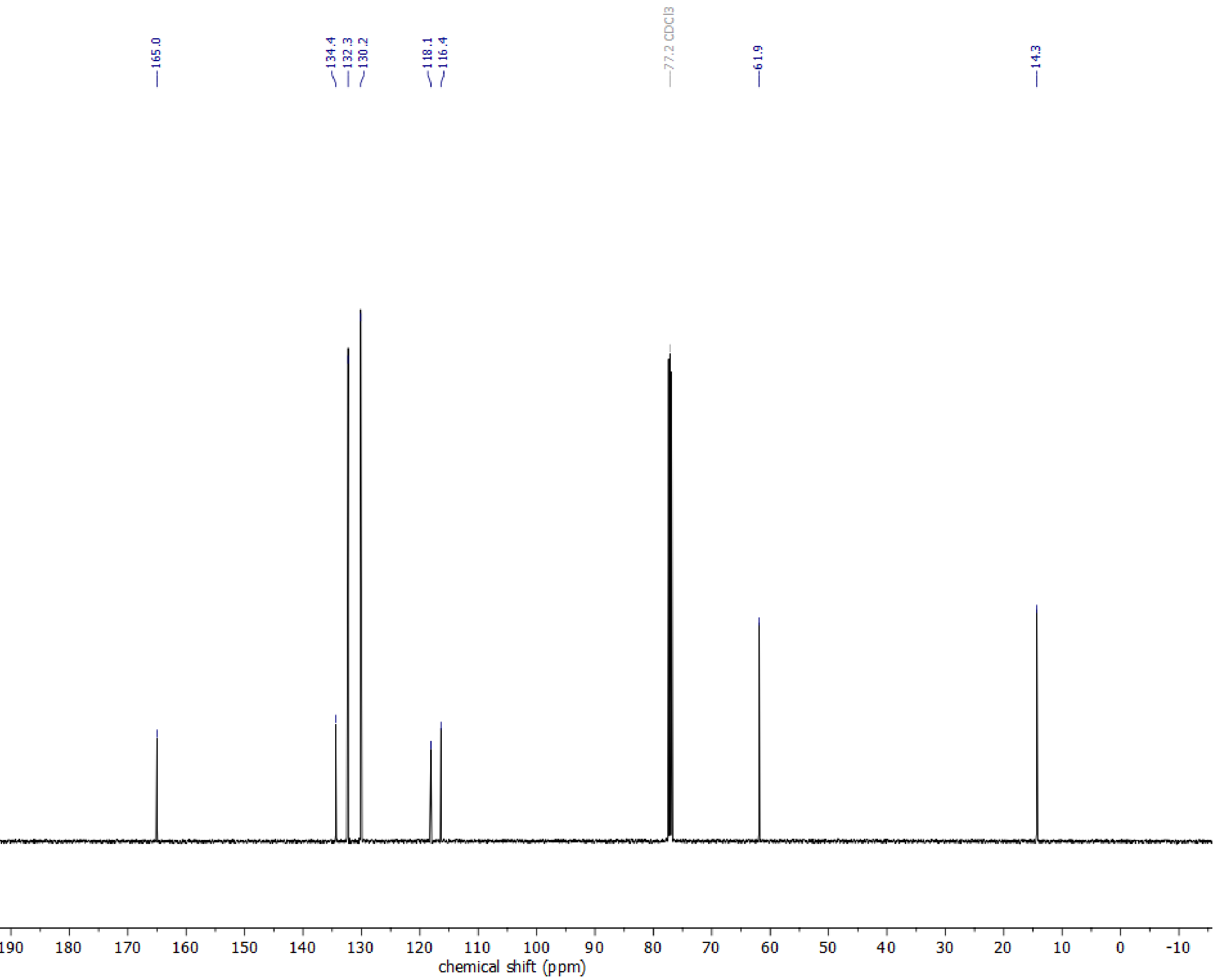

^{1}H NMR spectrum of 4-(1,1,1,3,3,3-hexafluoro-2-hydroxypropan-2-yl)benzonitrile (17)

CD_3OD, 25°C, 600 MHz

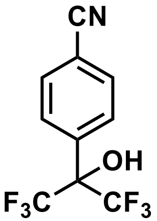

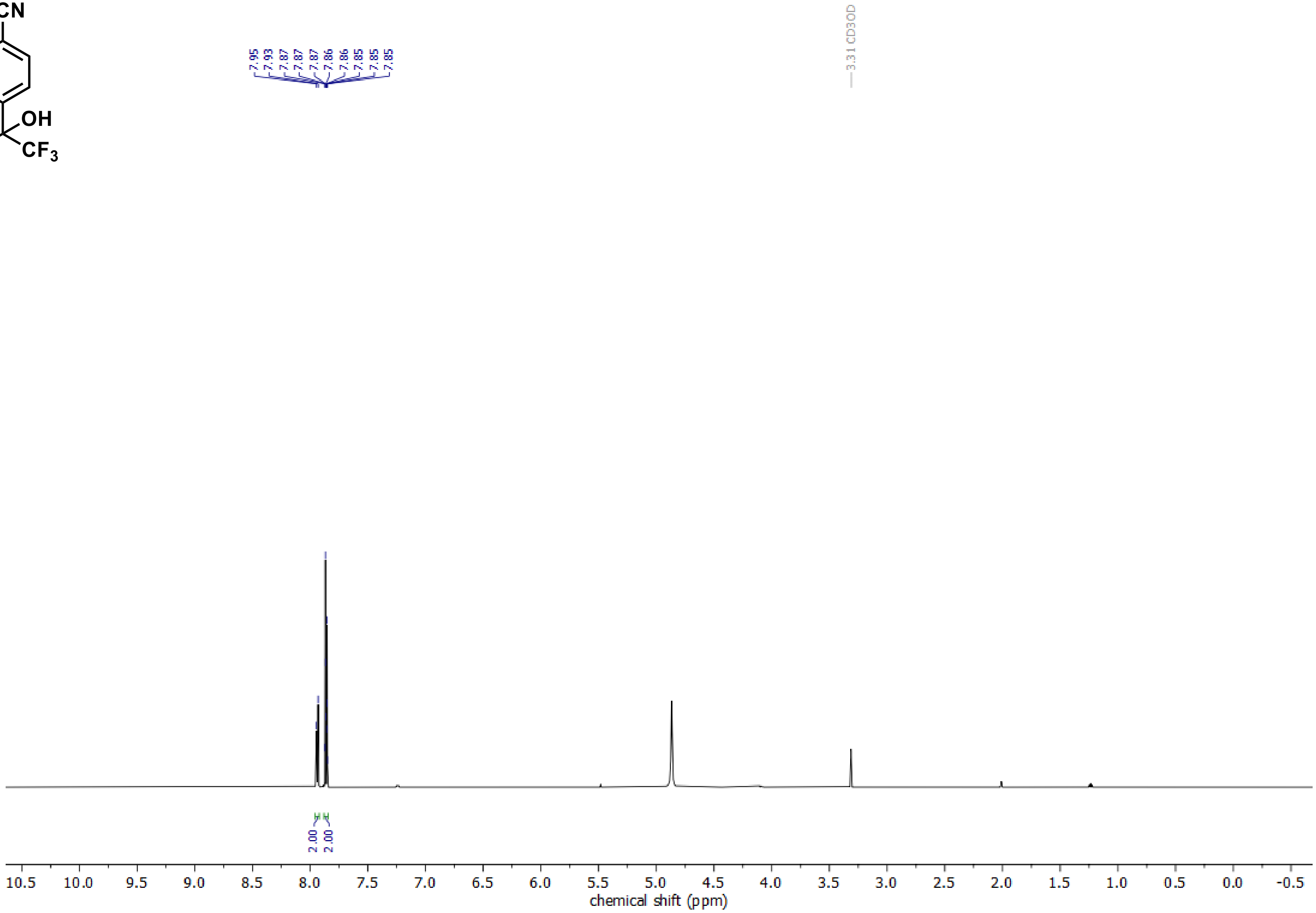

^{19}F NMR spectrum of 4-(1,1,1,3,3,3-hexafluoro-2-hydroxypropan-2-yl)benzonitrile (17)

CD_3OD, 25°C, 565 MHz

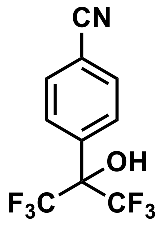

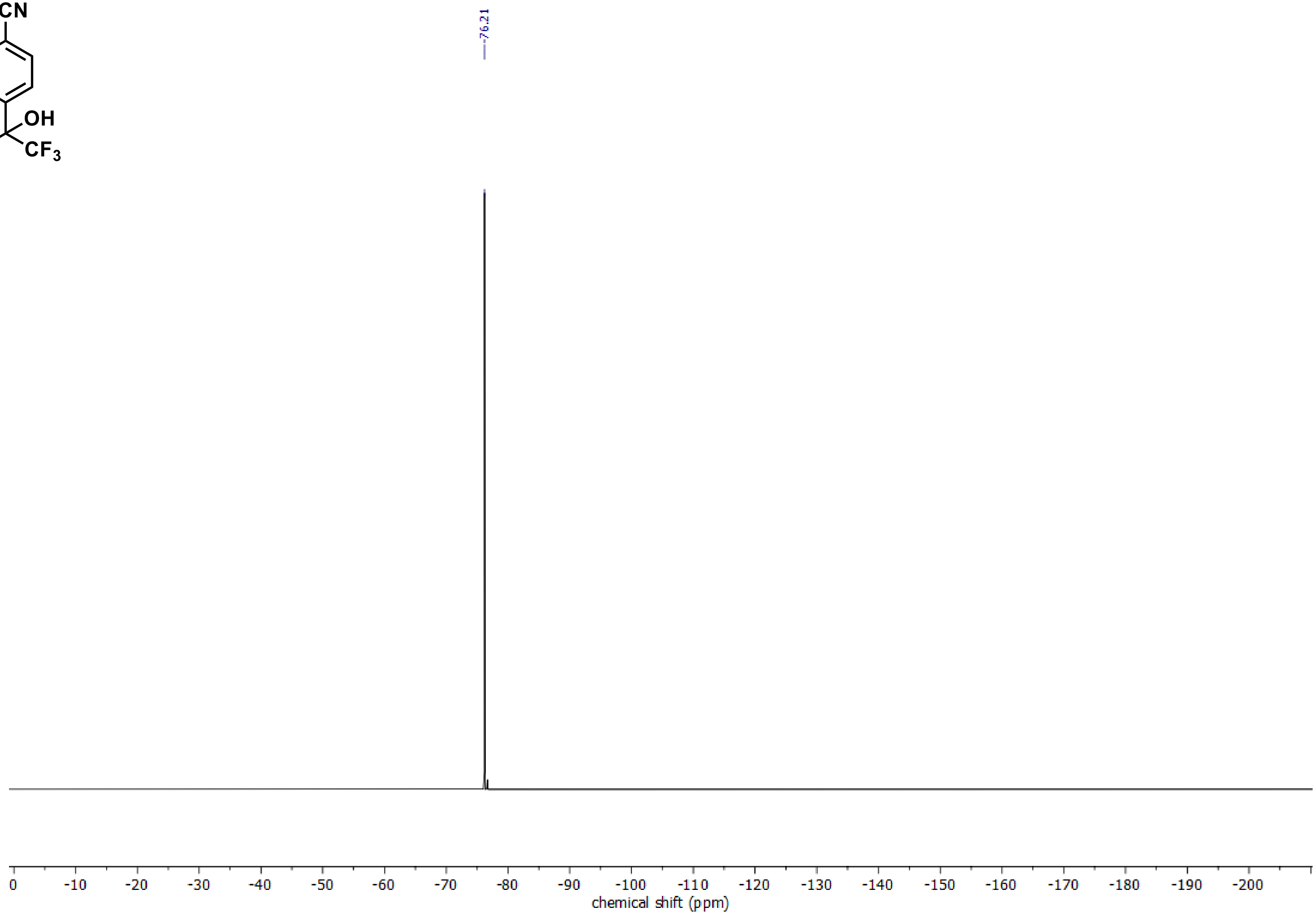

^{13}C NMR spectrum of 4-(1,1,1,3,3,3-hexafluoro-2-hydroxypropan-2-yl)benzonitrile (17)

CD_3OD, 25°C, 151 MHz

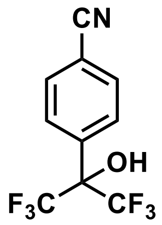

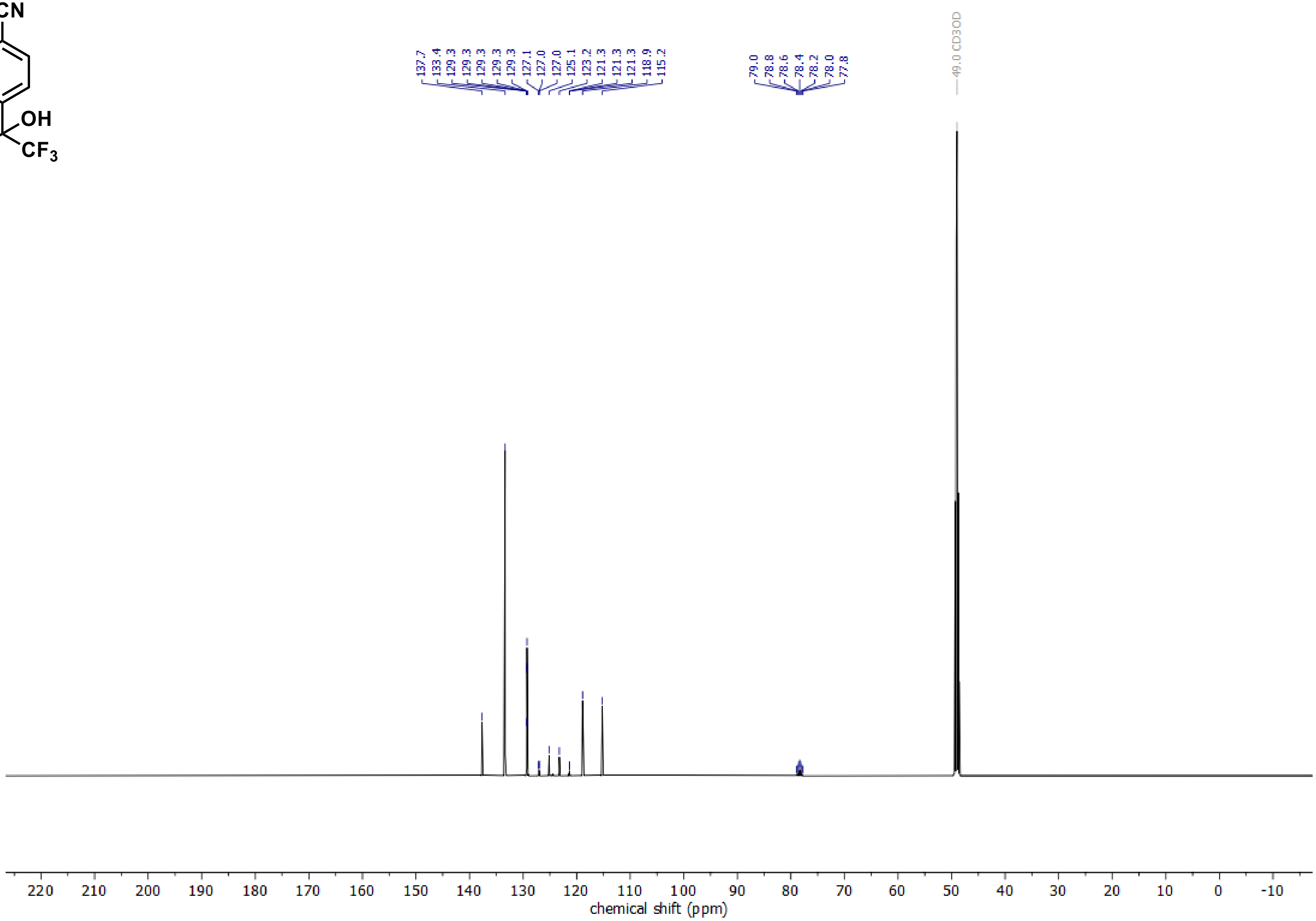

^{1}H NMR spectrum of *rac*-Aminoglutethimide-derived benzonitrile 18

DMSO-d_6, 25°C, 500 MHz

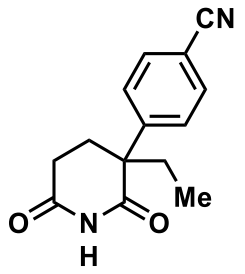

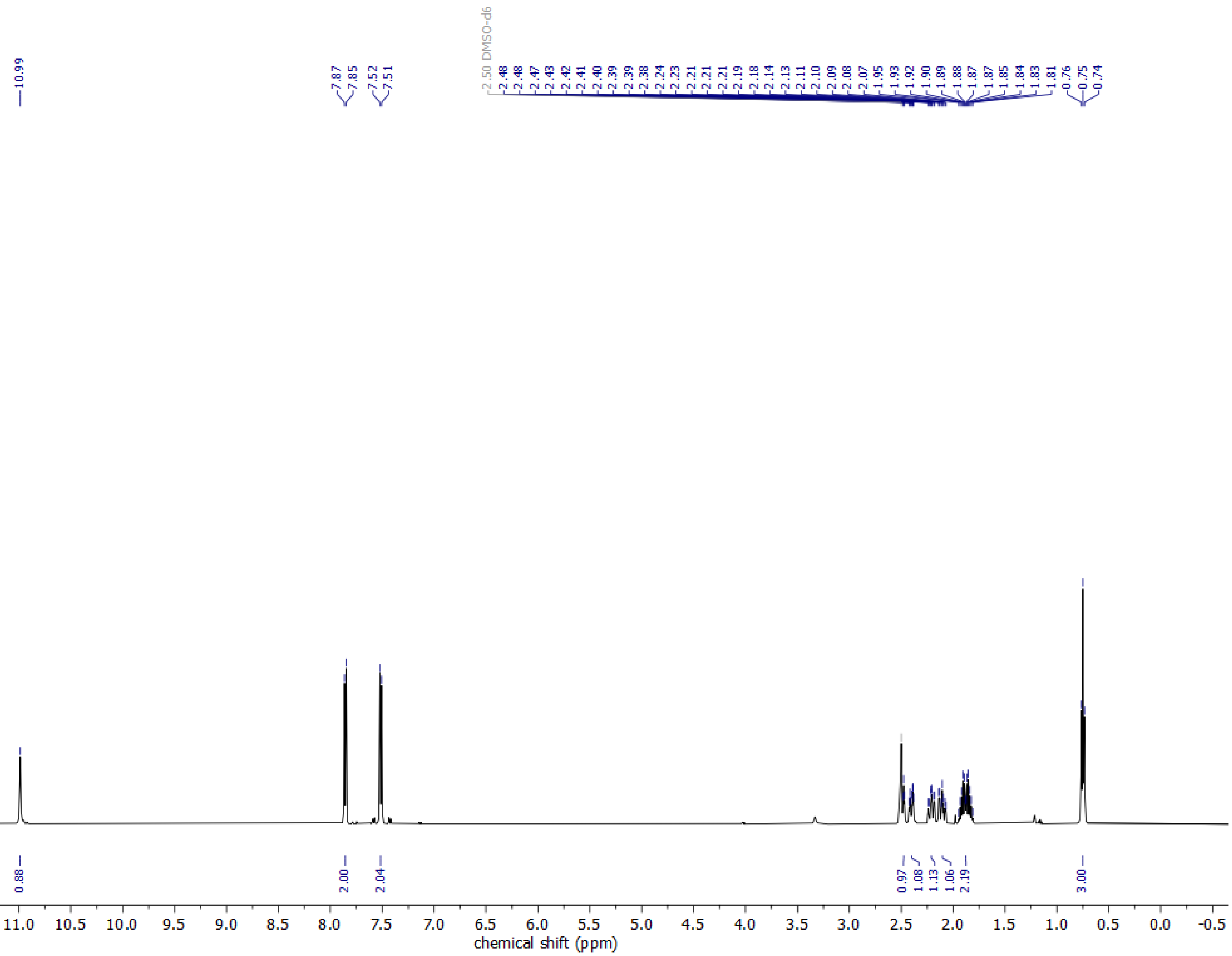

^{13}C NMR spectrum of *rac*-Aminoglutethimide-derived benzonitrile 18

DMSO-d_6, 25°C, 125 MHz

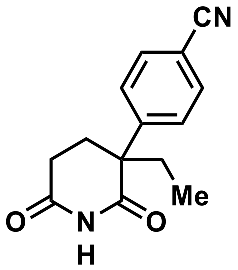

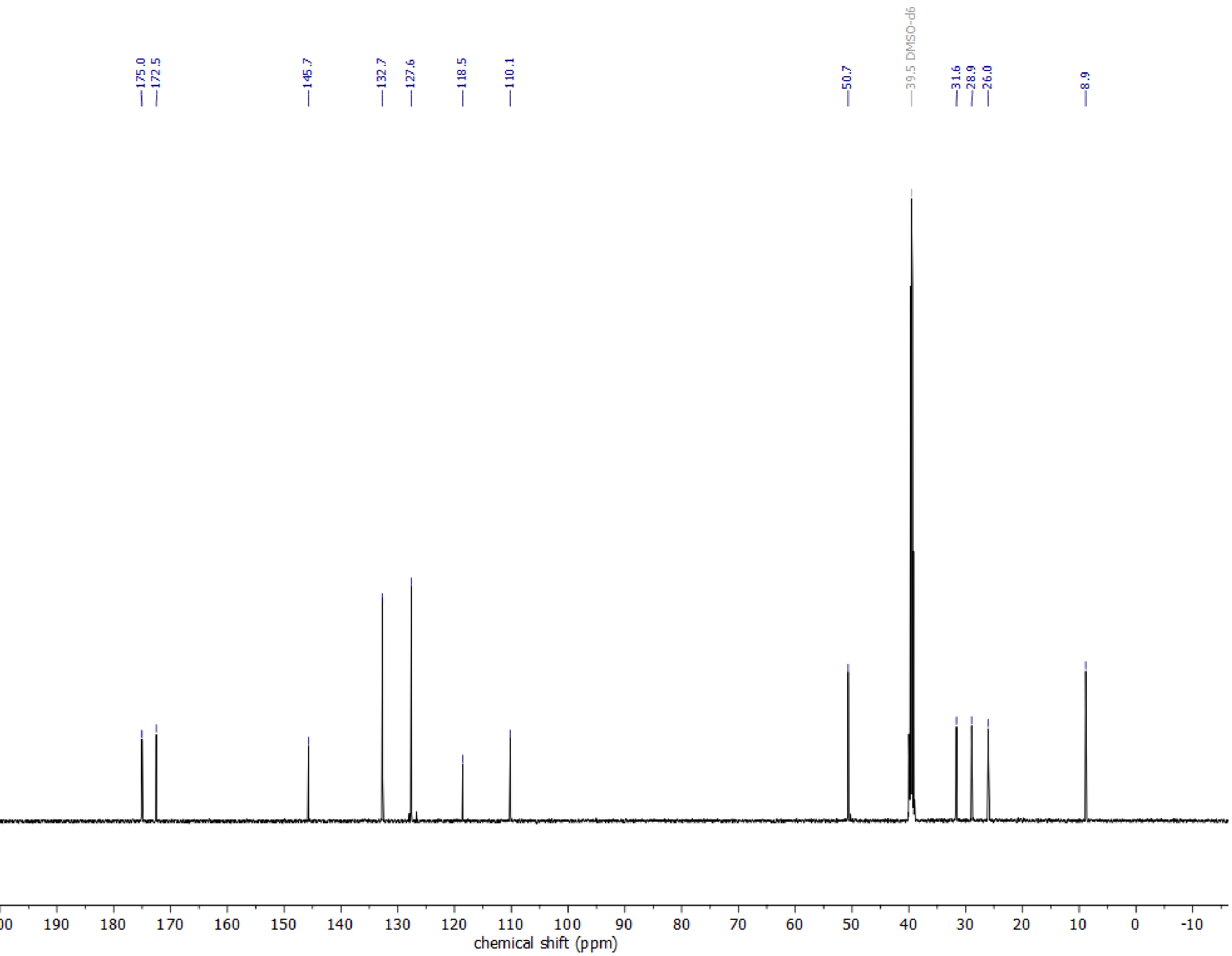

^{1}H NMR spectrum of 2-bromoterephthalonitrile (19)

$CDCl_3$, 25°C, 500 MHz

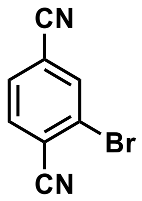

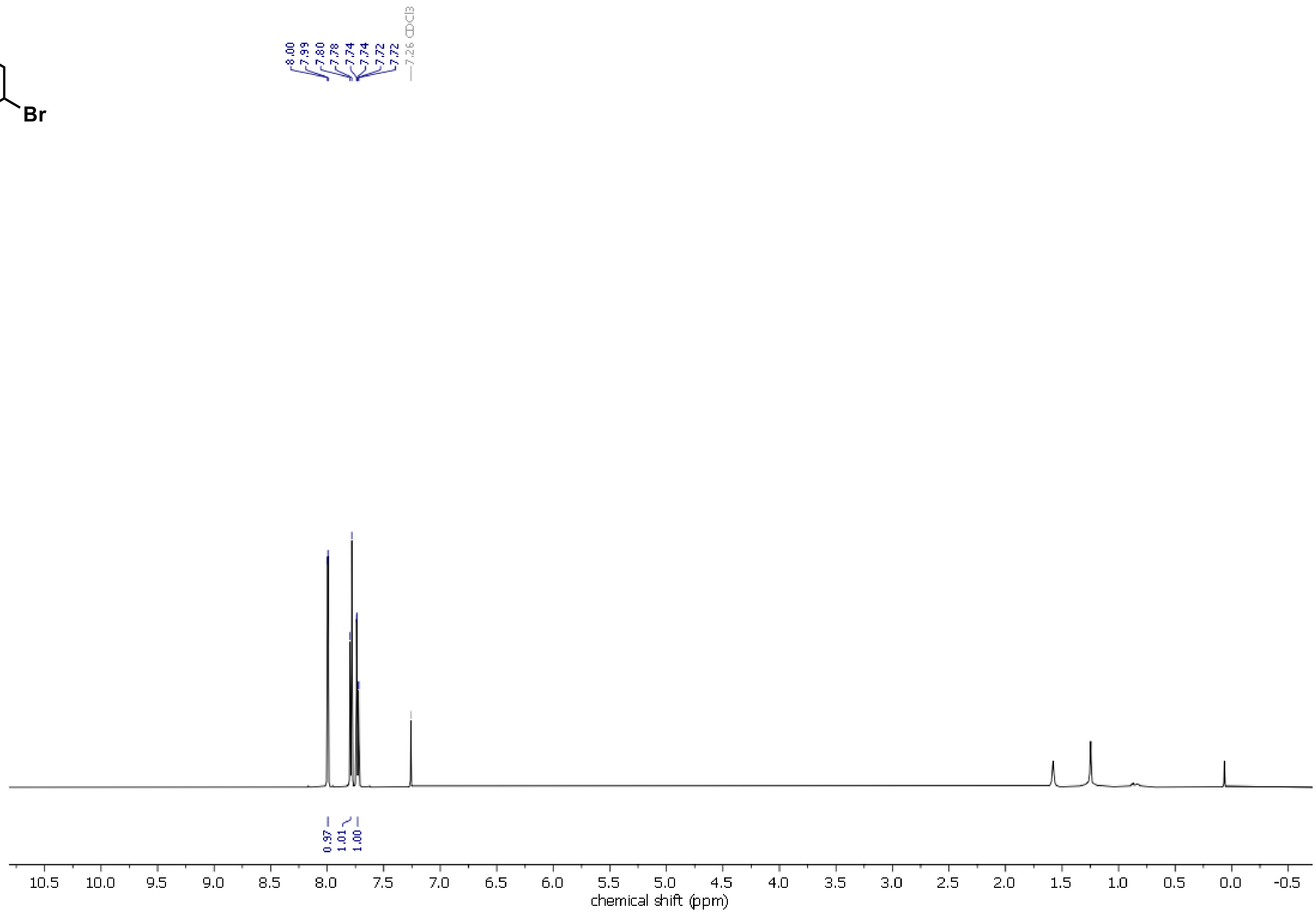

^{13}C NMR spectrum of 2-bromoterephthalonitrile (19)

$CDCl_3$, 25°C, 125 MHz

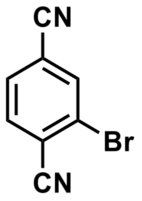

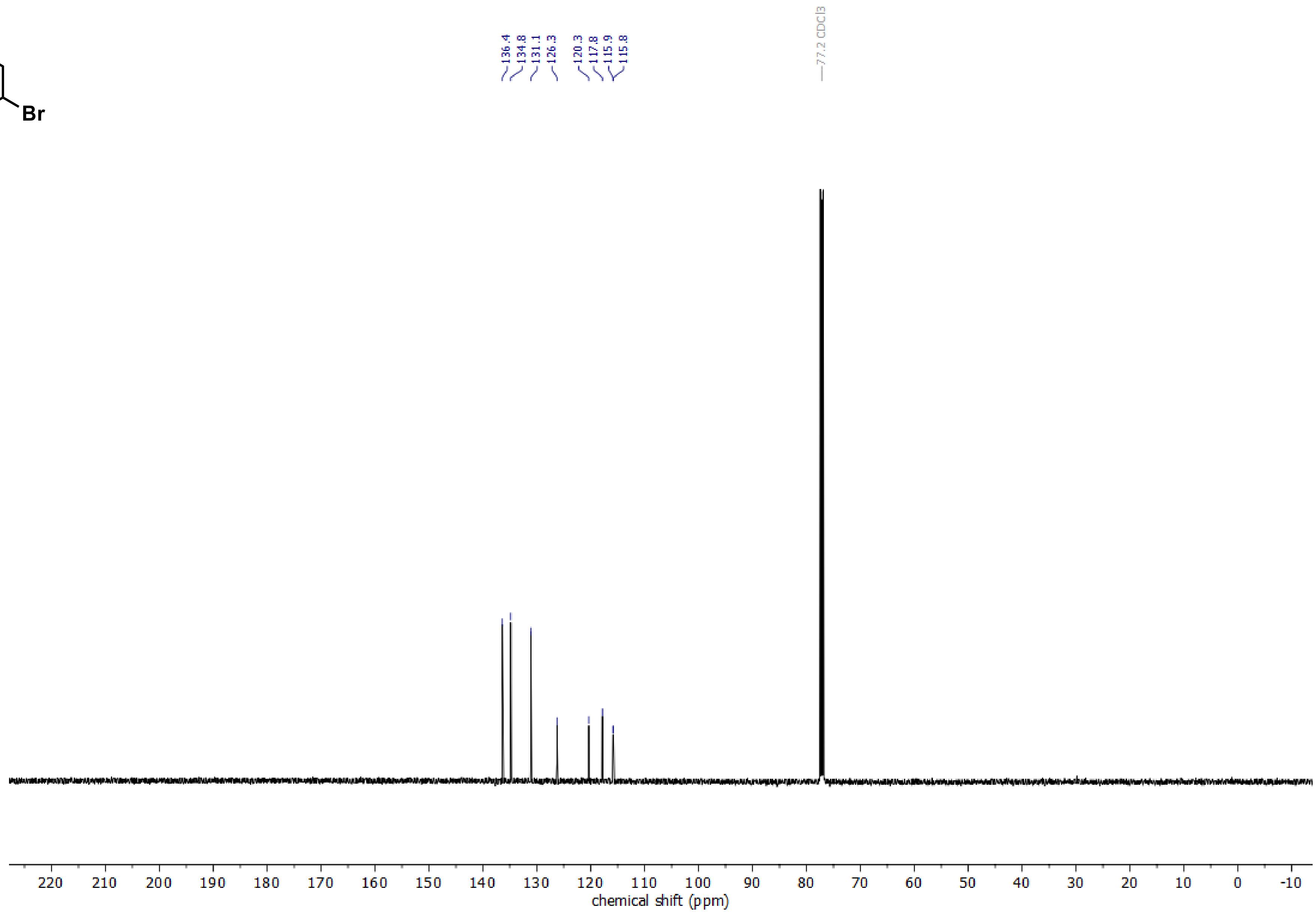

^{1}H NMR spectrum of methyl 3-cyanothiophene-2-carboxylate (20)

$CDCl_3$, 25°C, 500 MHz

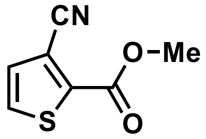

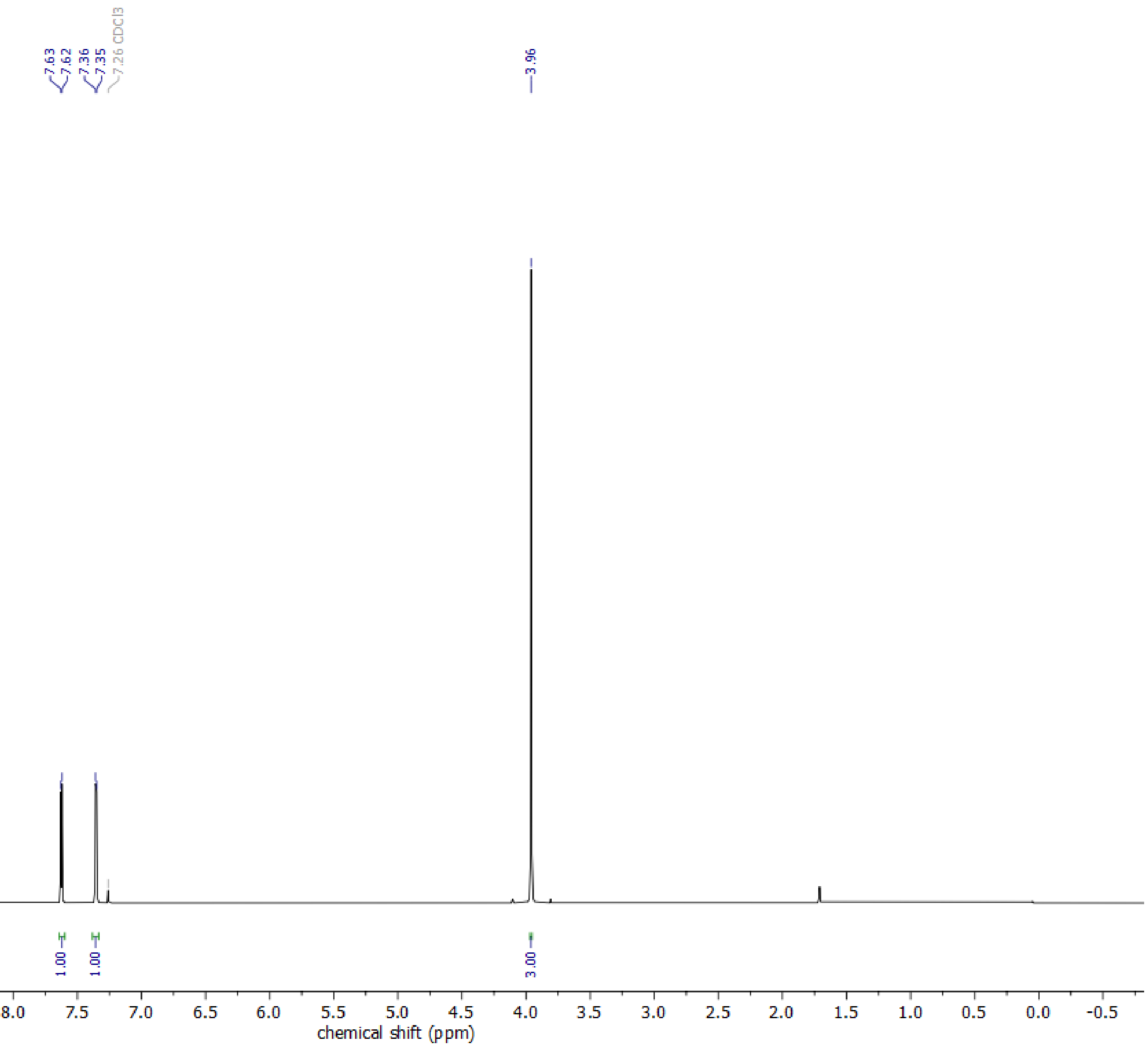

^{13}C NMR spectrum of methyl 3-cyanothiophene-2-carboxylate (20)

$CDCl_3$, 25°C, 125 MHz

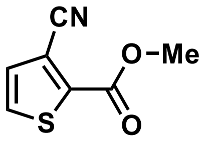

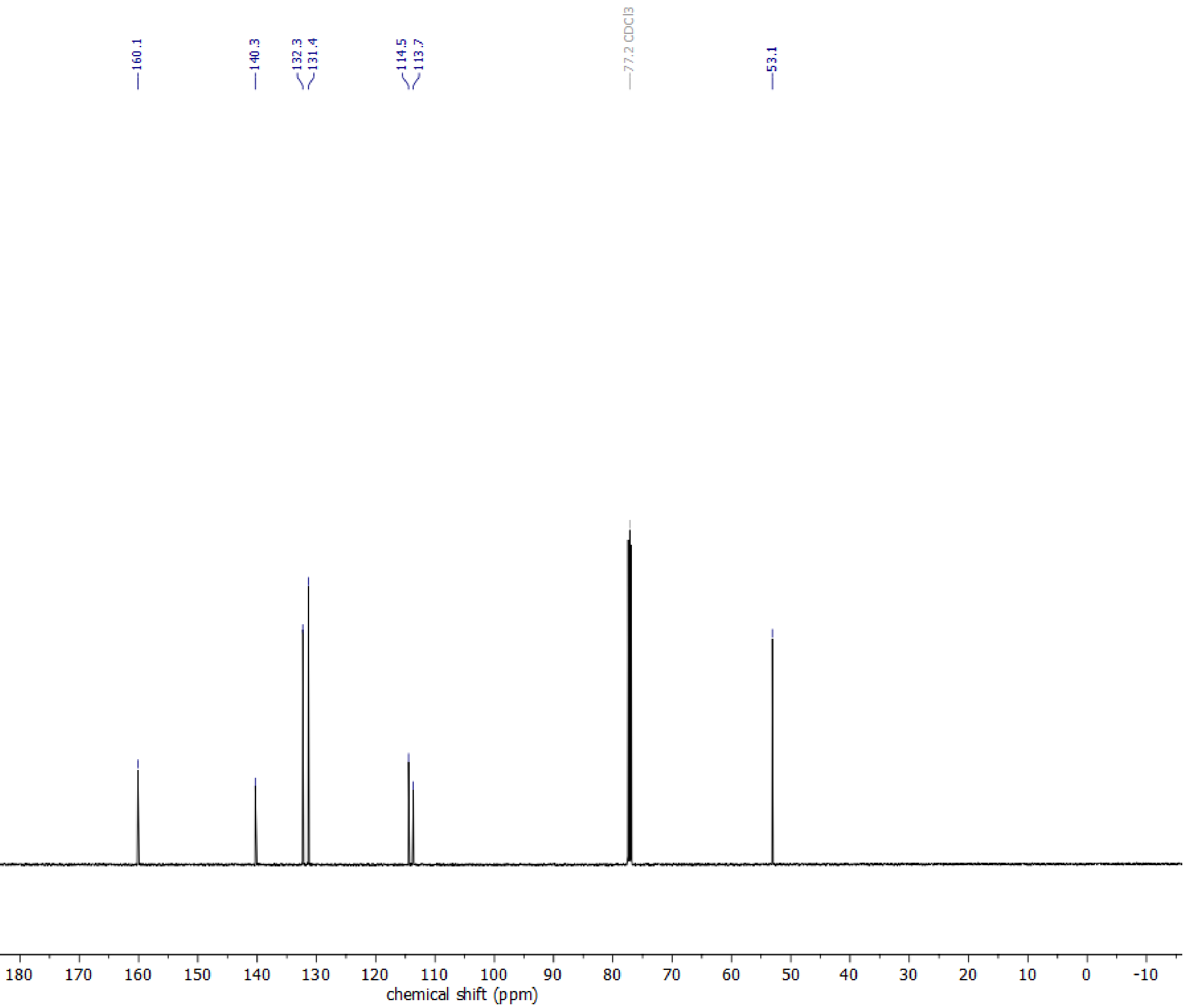

^{1}H NMR spectrum of 4-(aminomethyl)phenyl)-1,1,1,3,3,3-hexafluoropropan-2-ol (22)

CD_3CN, 25°C, 500 MHz

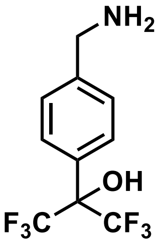

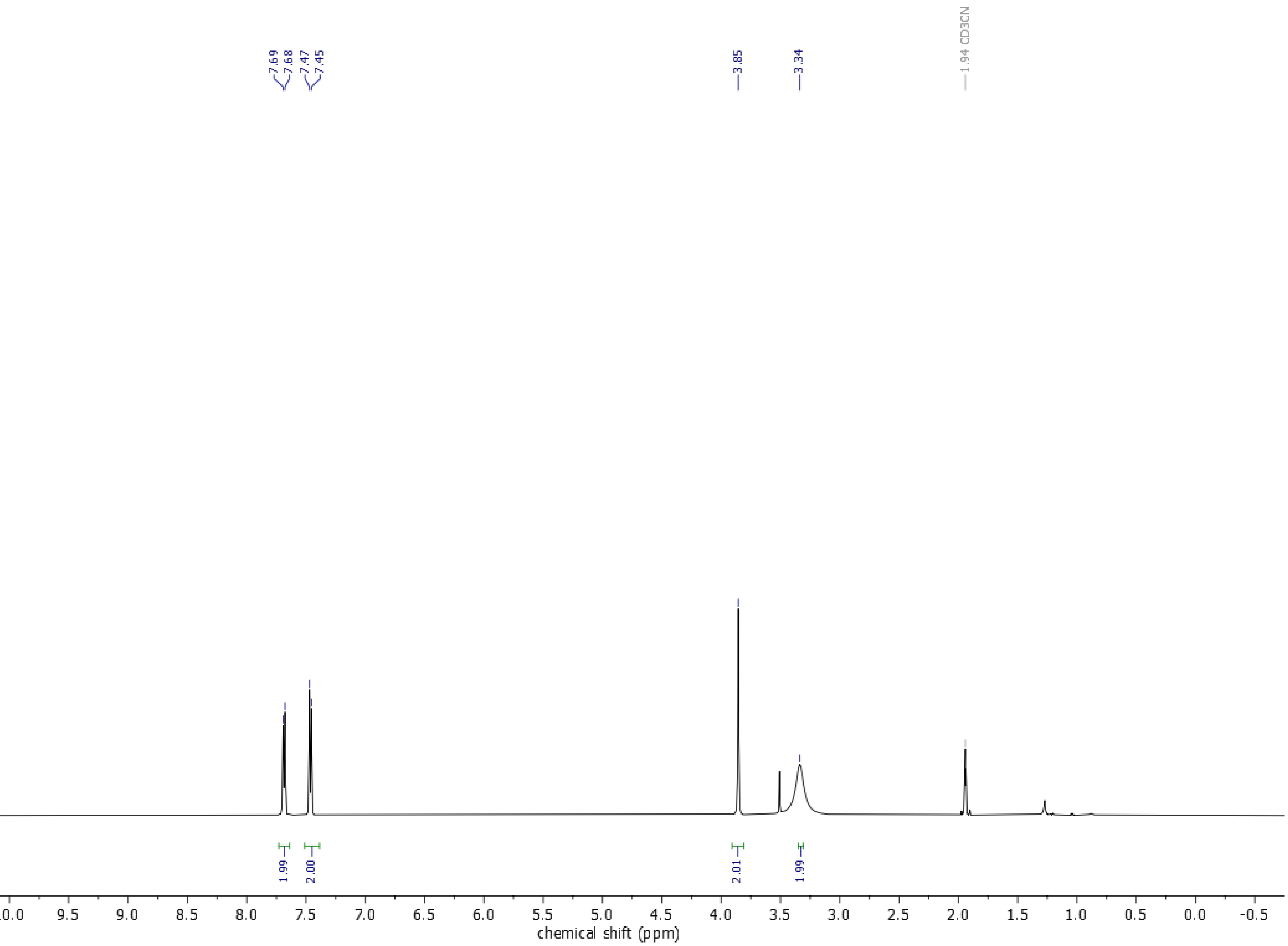

^{19}F NMR spectrum of 4-(aminomethyl)phenyl)-1,1,1,3,3,3-hexafluoropropan-2-ol (22)

CD_3CN, 25°C, 470 MHz

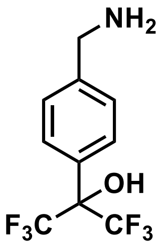

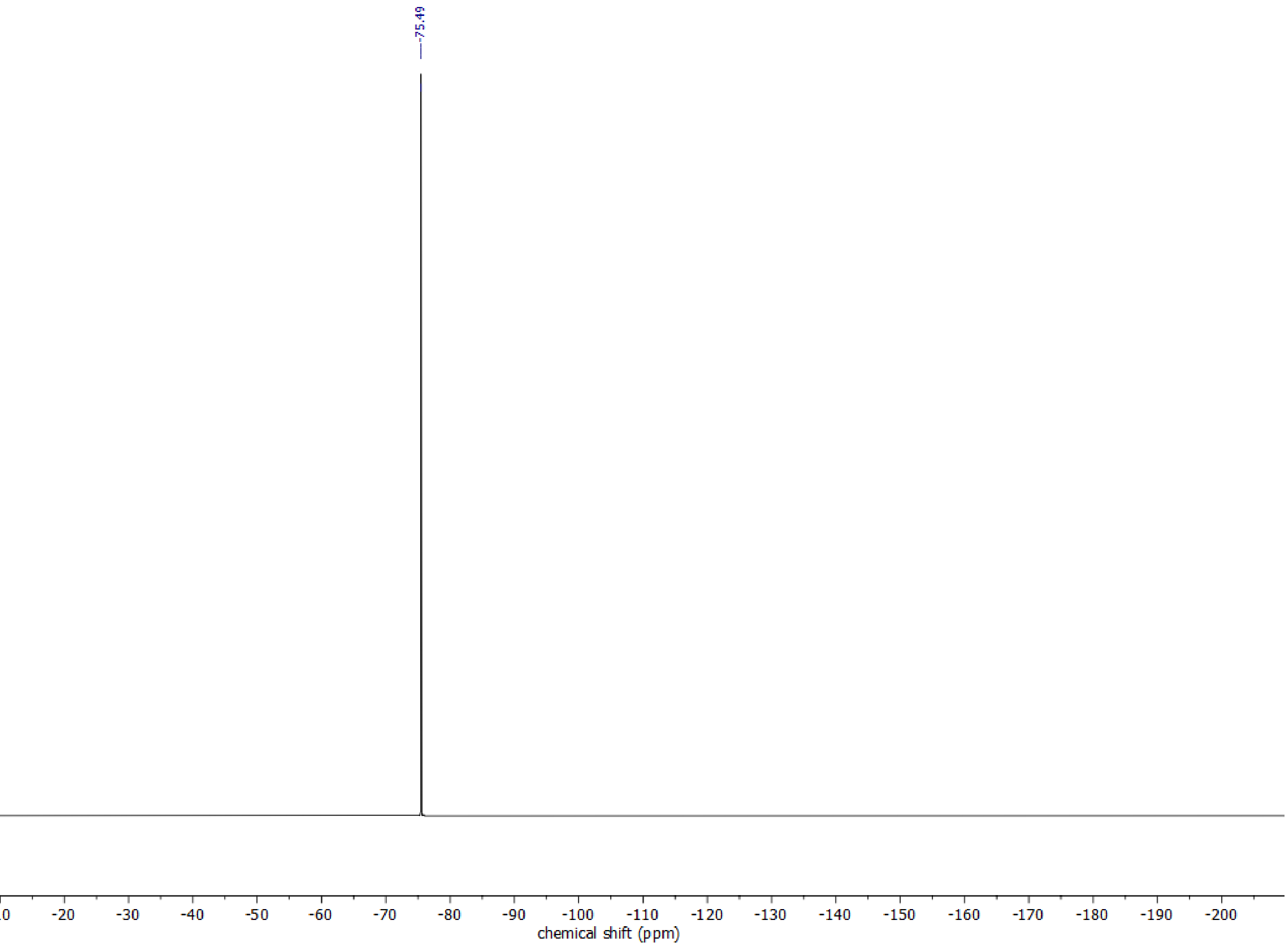

^{13}C NMR spectrum of 4-(aminomethyl)phenyl)-1,1,1,3,3,3-hexafluoropropan-2-ol (22)

CD_3CN, 25°C, 125 MHz

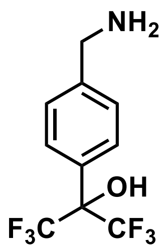

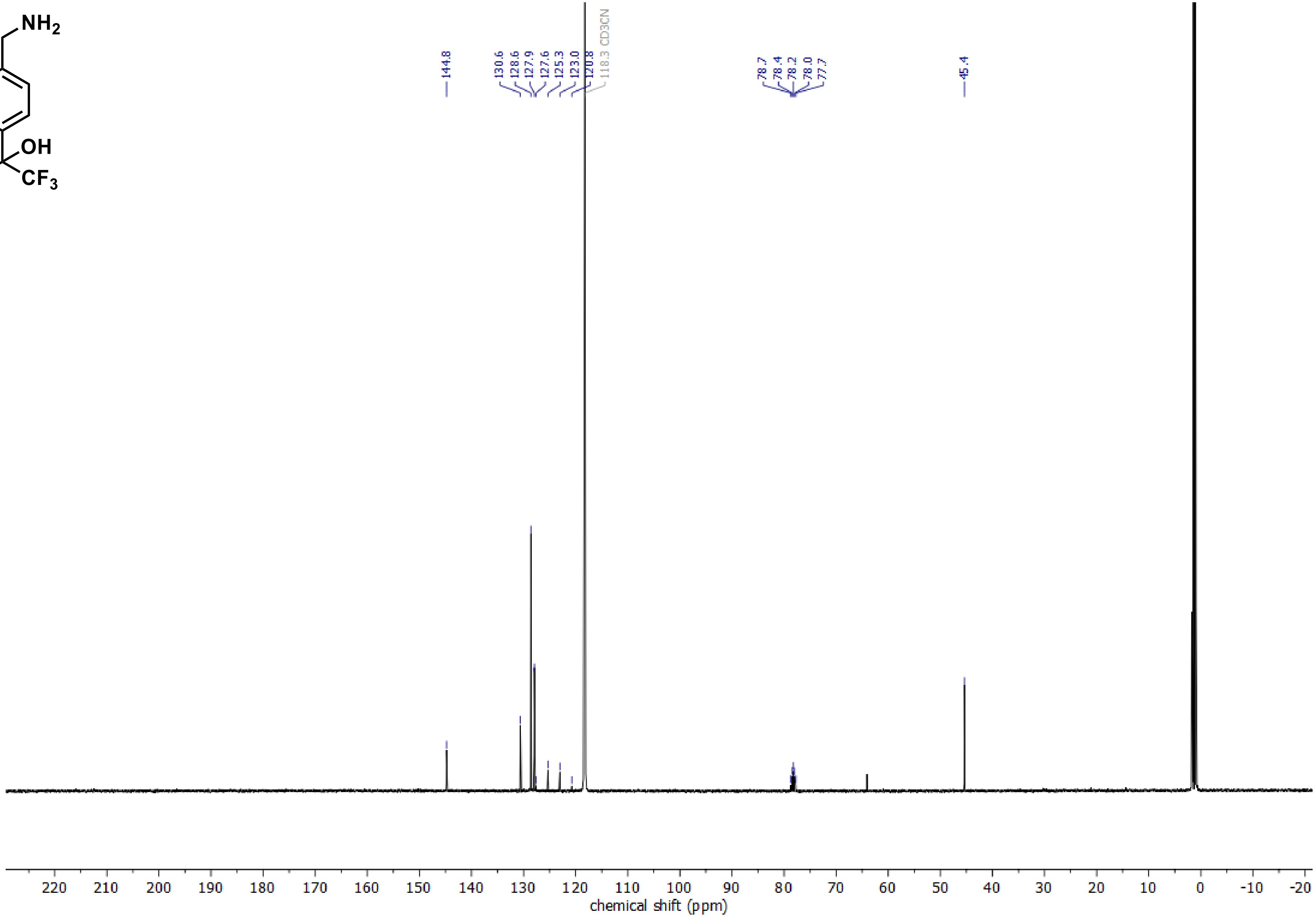

^{1}H NMR spectrum of 2-(4-(1H-tetrazol-5-yl)phenyl)-1,1,1,3,3,3-hexafluoropropan-2-ol (23)

DMSO-d_6, 25°C, 500 MHz

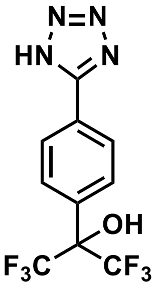

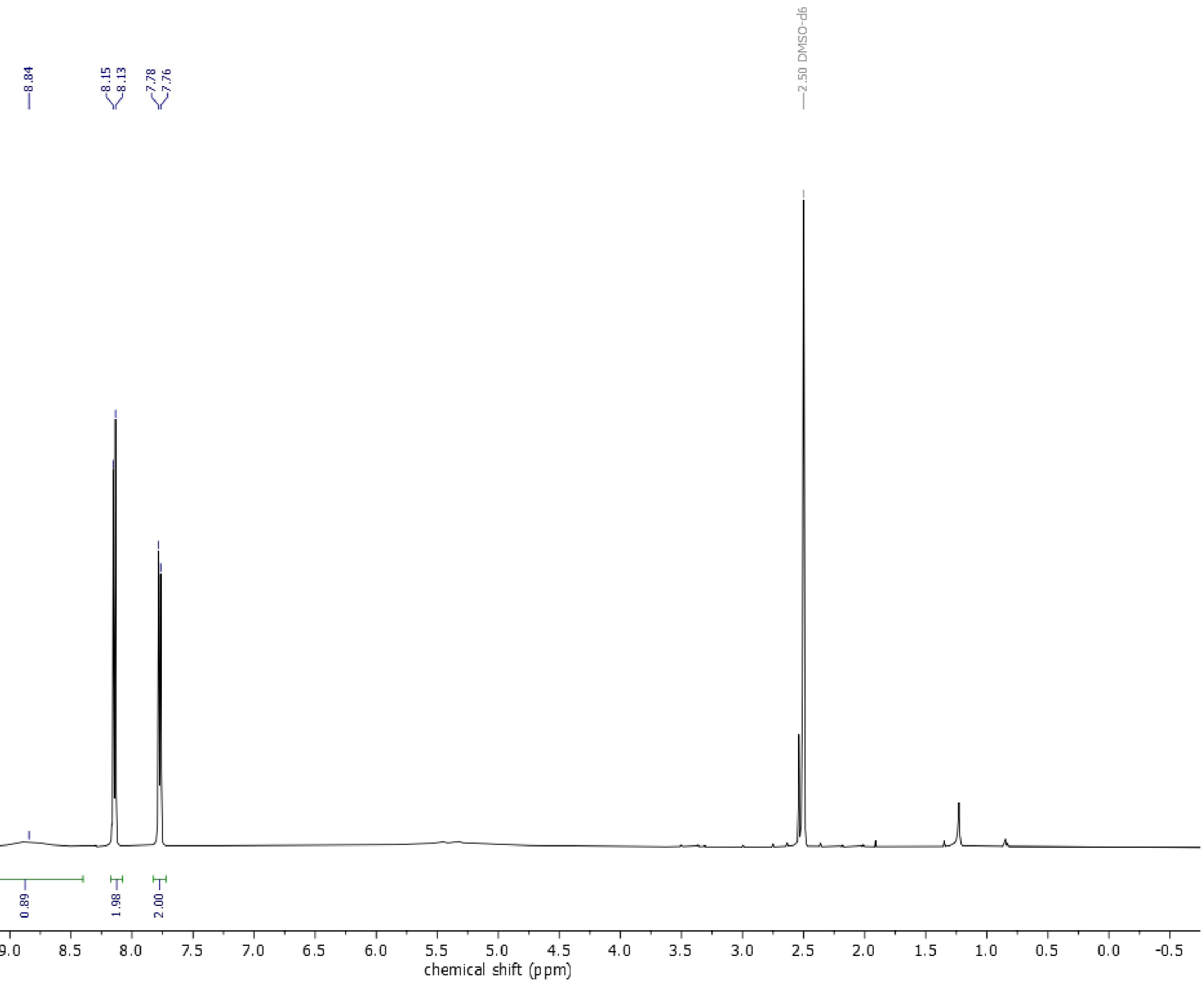

^{19}F NMR spectrum of 2-(4-(1H-tetrazol-5-yl)phenyl)-1,1,1,3,3,3-hexafluoropropan-2-ol (23)

DMSO-d_6, 25°C, 470 MHz

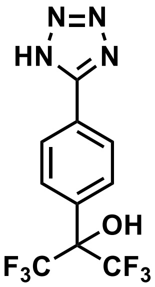

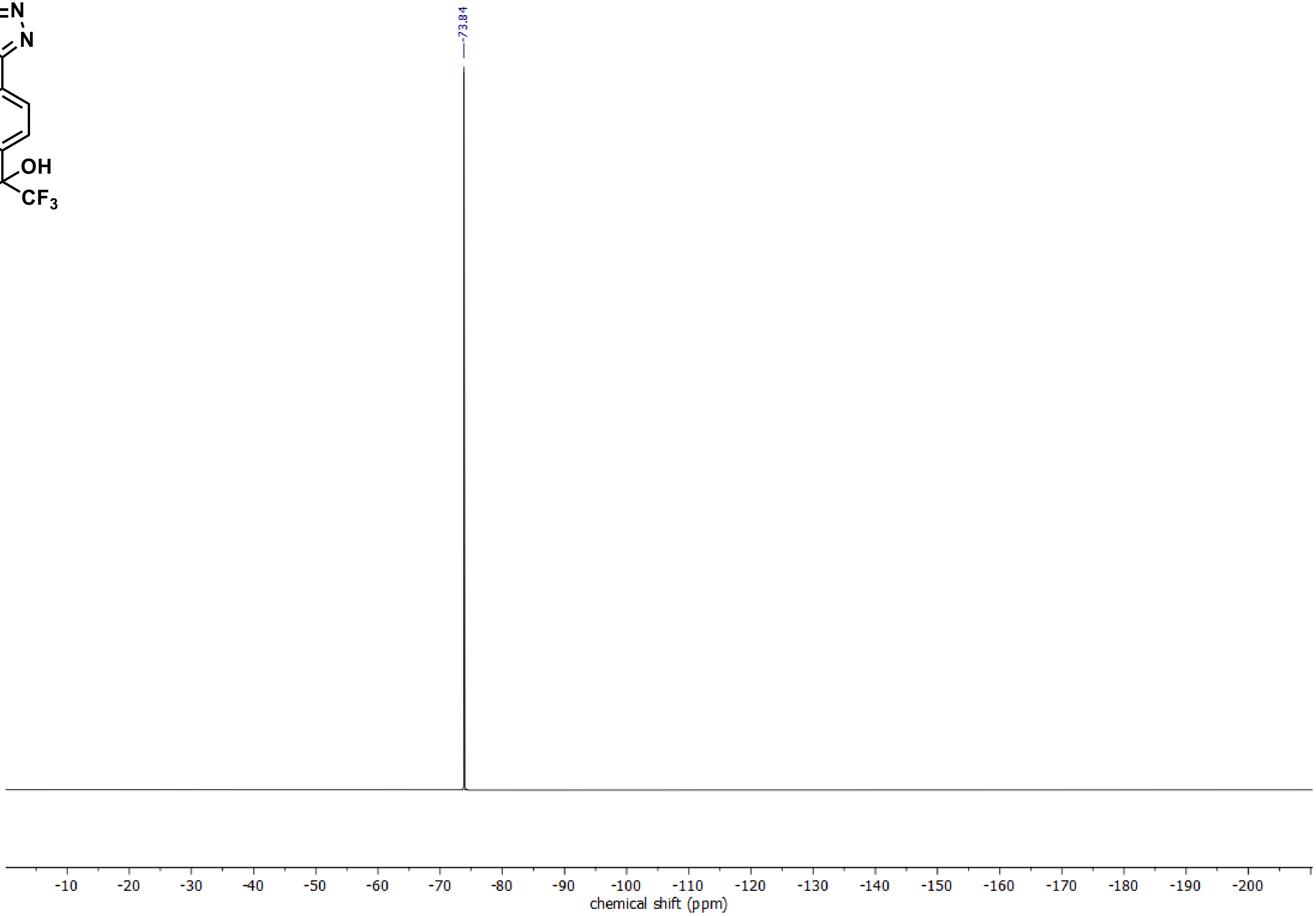

^{13}C NMR spectrum of 2-(4-(1H-tetrazol-5-yl)phenyl)-1,1,1,3,3,3-hexafluoropropan-2-ol (23)

DMSO-d_6, 25°C, 125 MHz

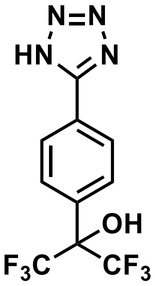

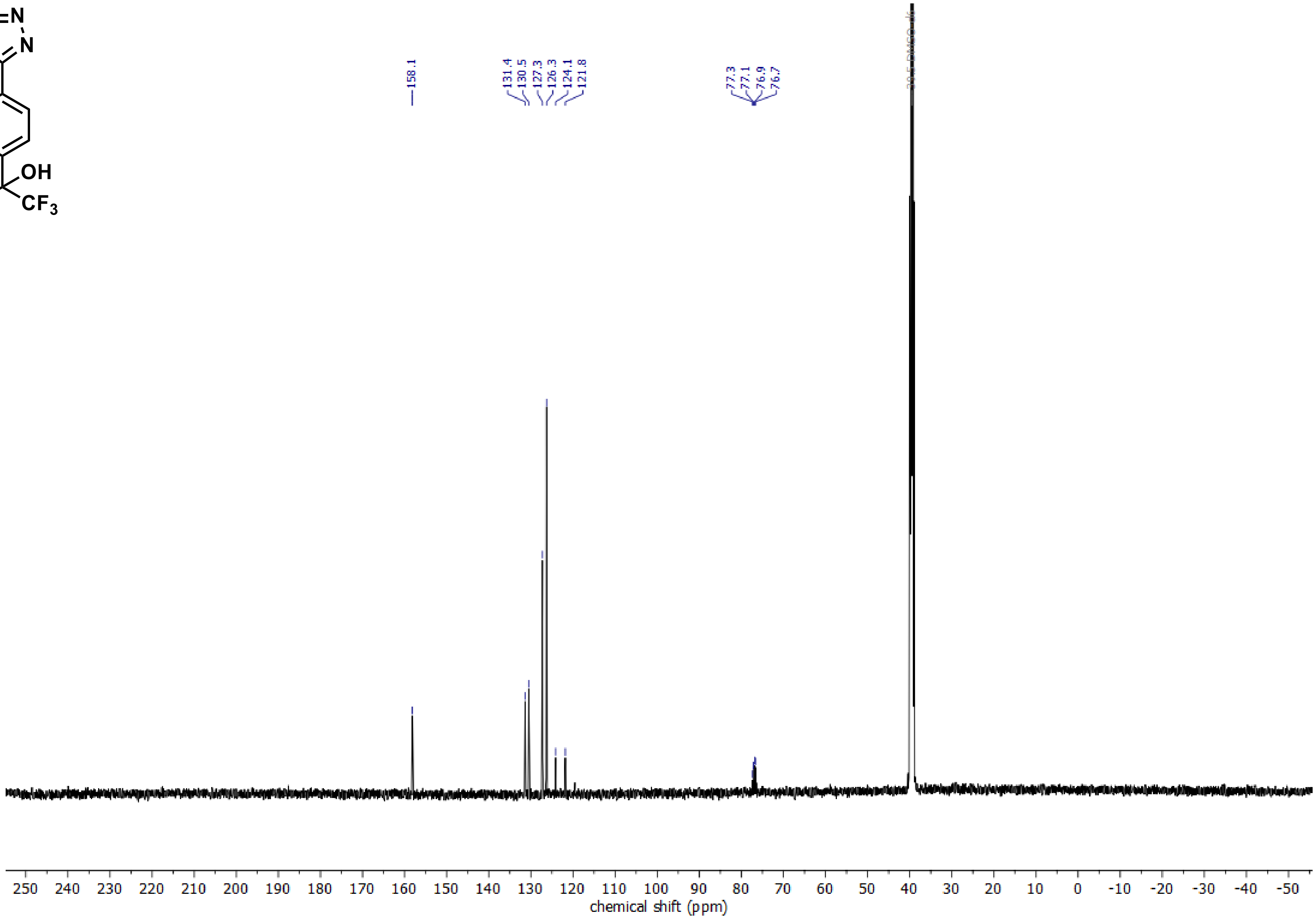

^1H NMR spectrum of 4-(1,1,1,3,3,3-hexafluoro-2-hydroxypropan-2-yl)benzamide (24)

DMSO-d_6, 25°C, 500 MHz

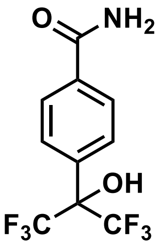

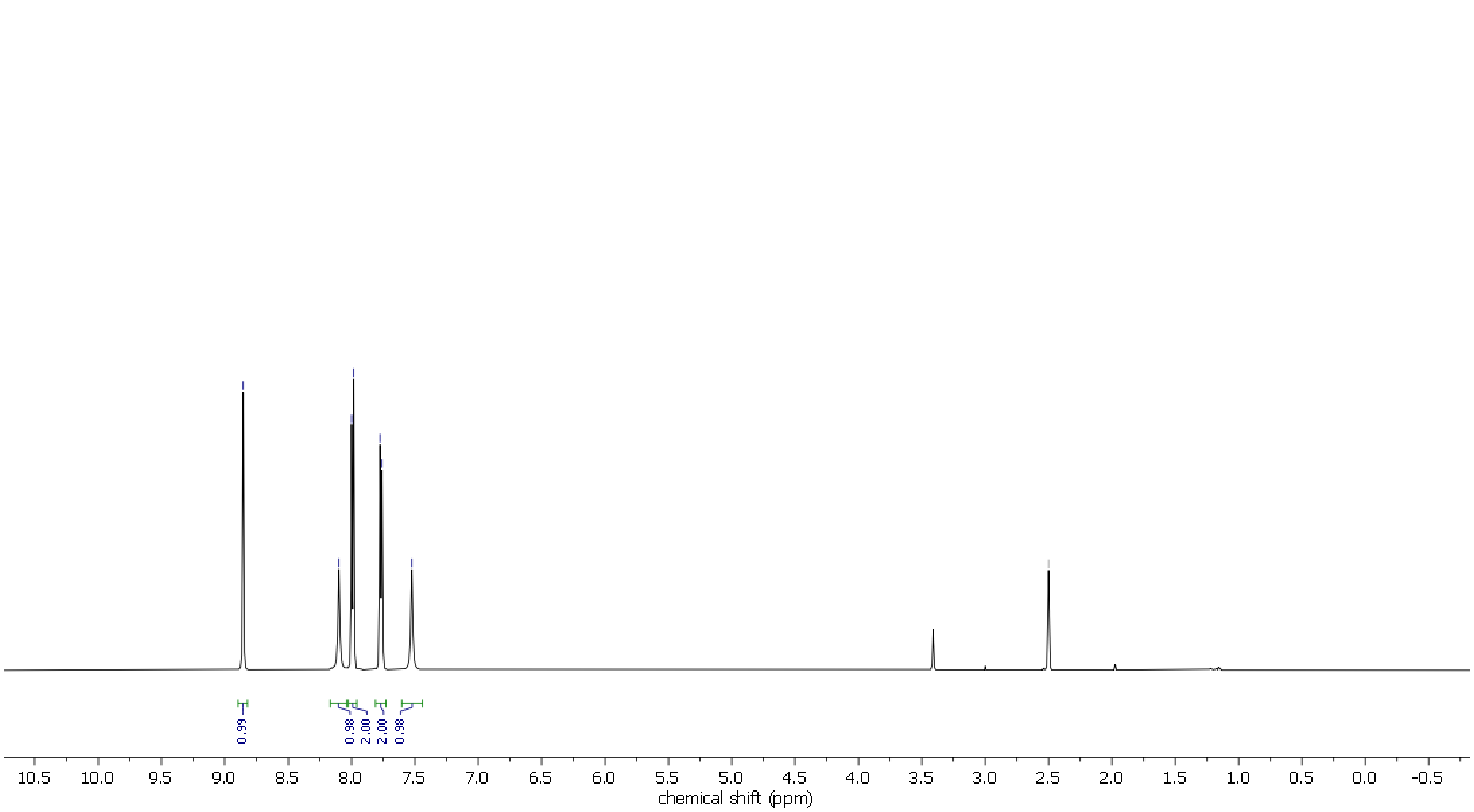

^{19}F NMR spectrum of 4-(1,1,1,3,3,3-hexafluoro-2-hydroxypropan-2-yl)benzamide (24)

DMSO-d_6, 25°C, 470 MHz

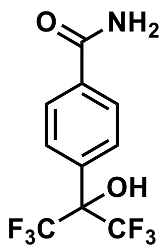

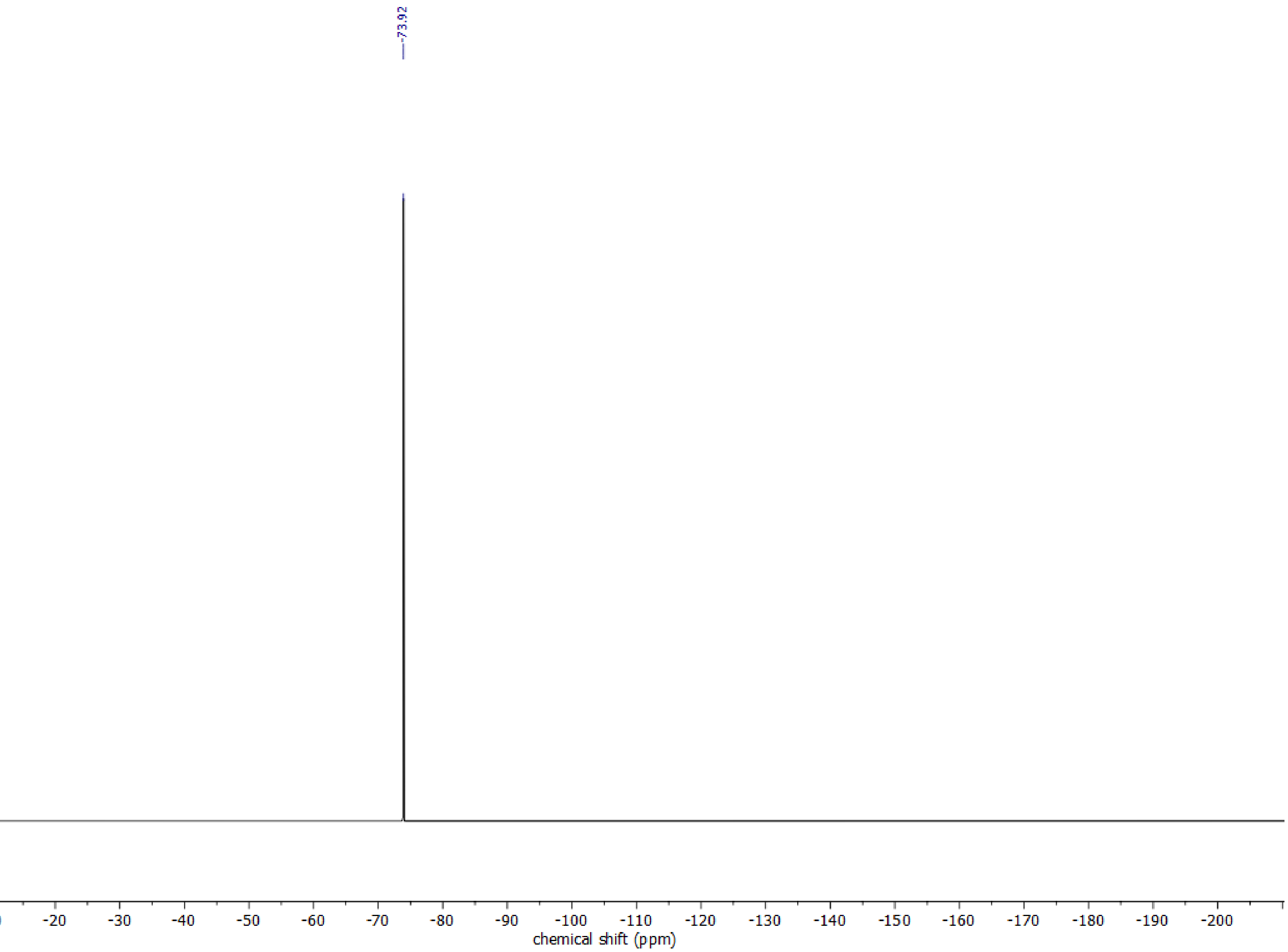

^{13}C NMR spectrum of 4-(1,1,1,3,3,3-hexafluoro-2-hydroxypropan-2-yl)benzoic acid (24)

DMSO-d_6, 25°C, 125 MHz

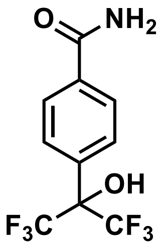

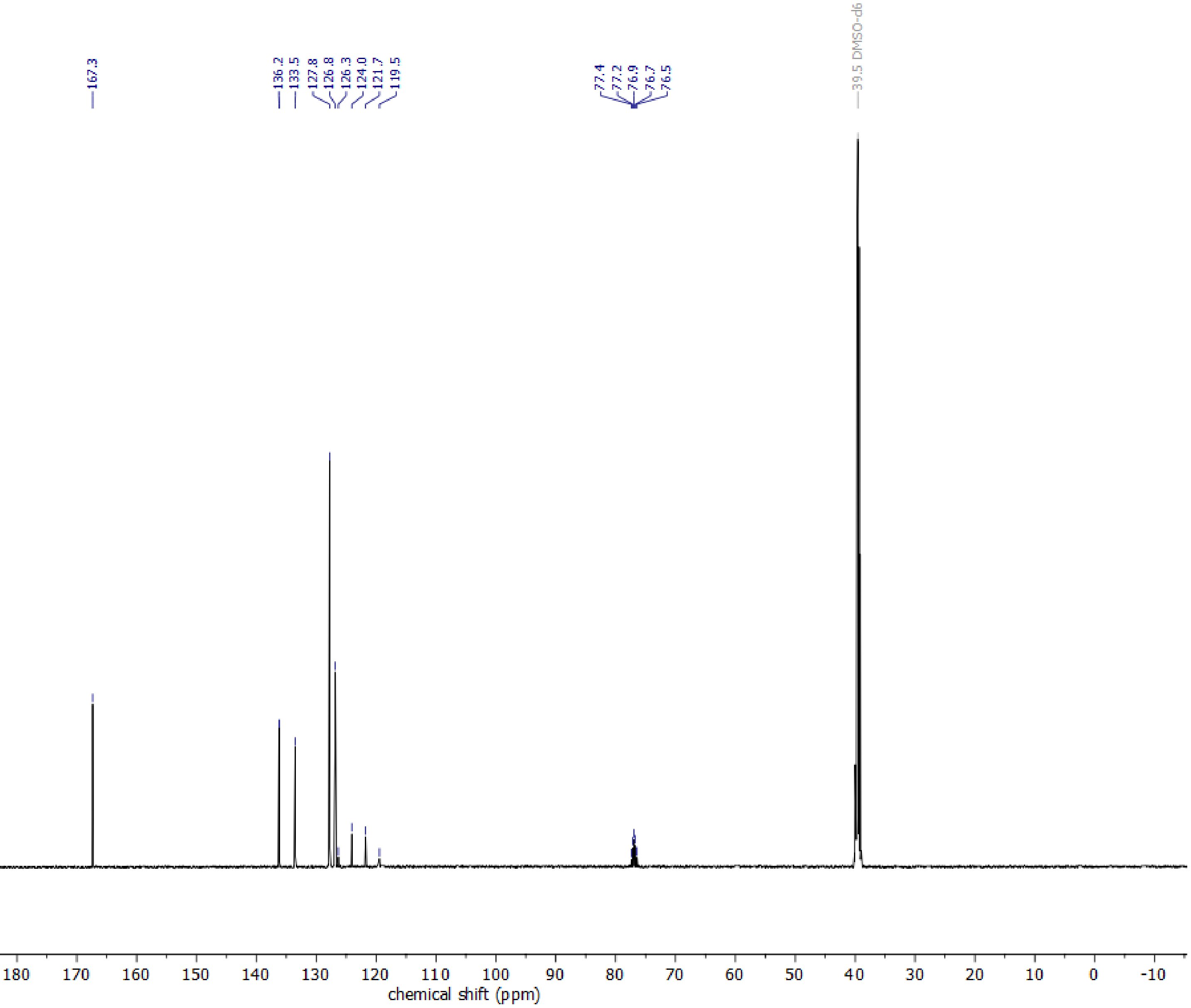

^{1}H NMR spectrum of 4-(1,1,1,3,3,3-hexafluoro-2-hydroxypropan-2-yl)benzoic acid (25)

DMSO-d_6, 25°C, 600 MHz

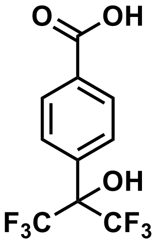

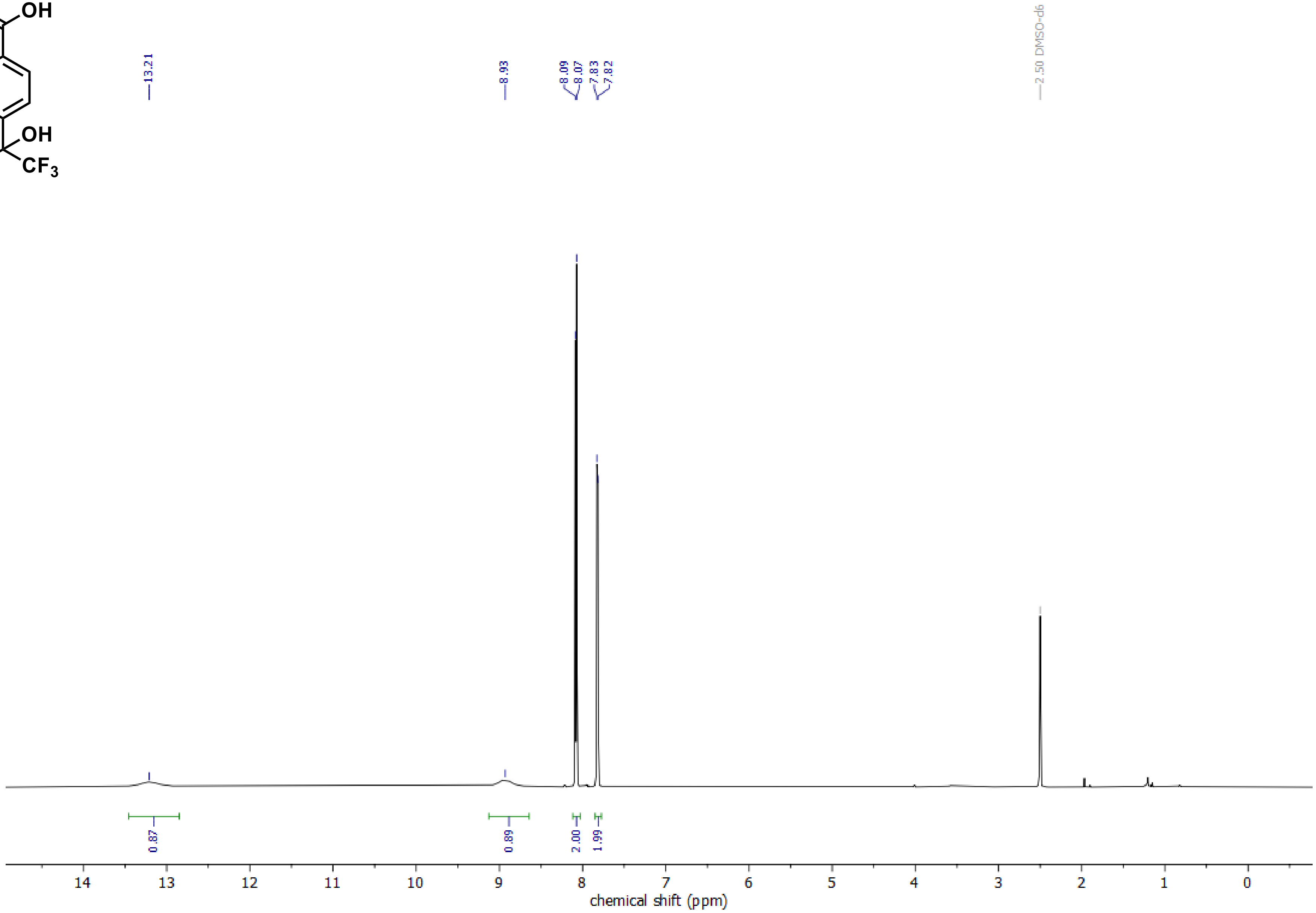

^{19}F NMR spectrum of 4-(1,1,1,3,3,3-hexafluoro-2-hydroxypropan-2-yl)benzoic acid (25)

DMSO-d_6, 25°C, 565 MHz

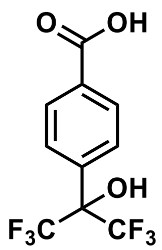

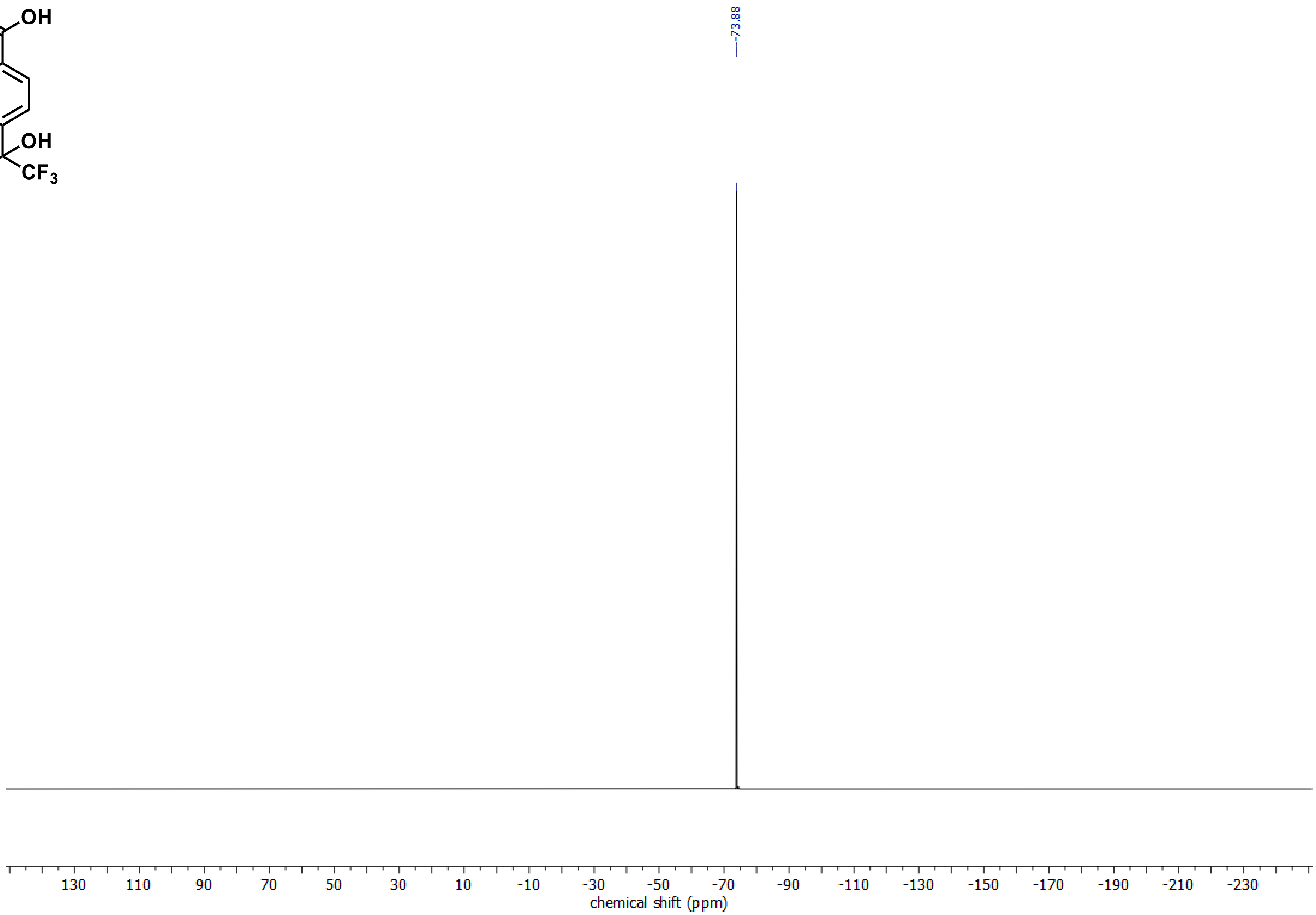

^{13}C NMR spectrum of 4-(1,1,1,3,3,3-hexafluoro-2-hydroxypropan-2-yl)benzoic acid (25)

DMSO-d_6, 25°C, 151 MHz

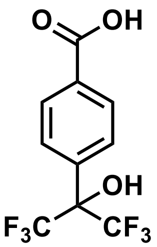

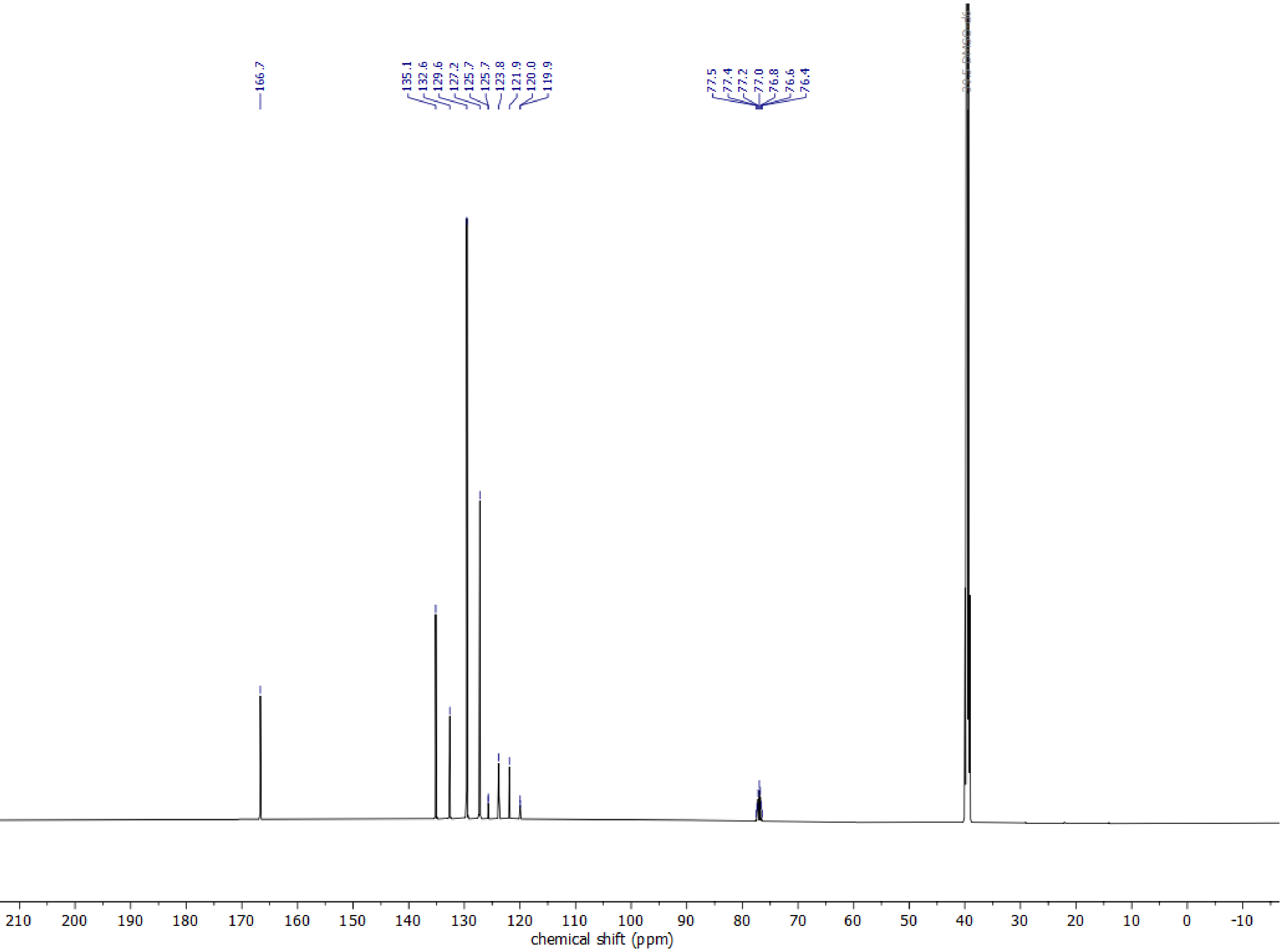

^{1}H NMR spectrum of 3-(*tert*-butyl)benzo[d][1,2,3]triazin-4(3H)-one (26)

$CDCl_3$, 25°C, 500 MHz

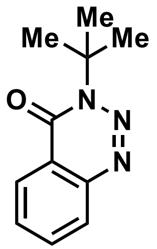

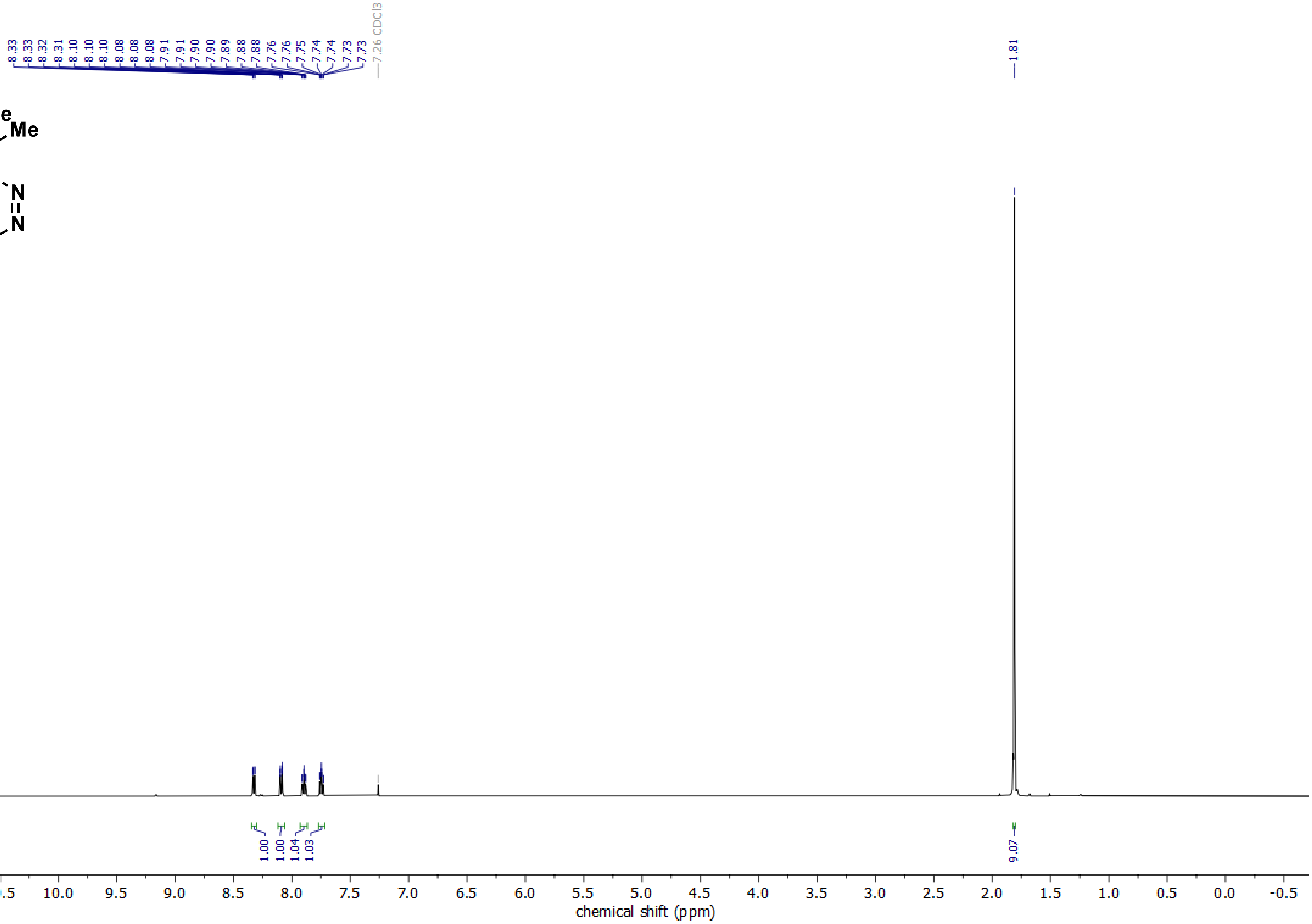

^{13}C NMR spectrum of 3-(*tert*-butyl)benzo[d][1,2,3]triazin-4(3H)-one (26)

$CDCl_3$, 25°C, 125 MHz

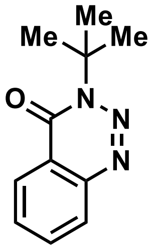

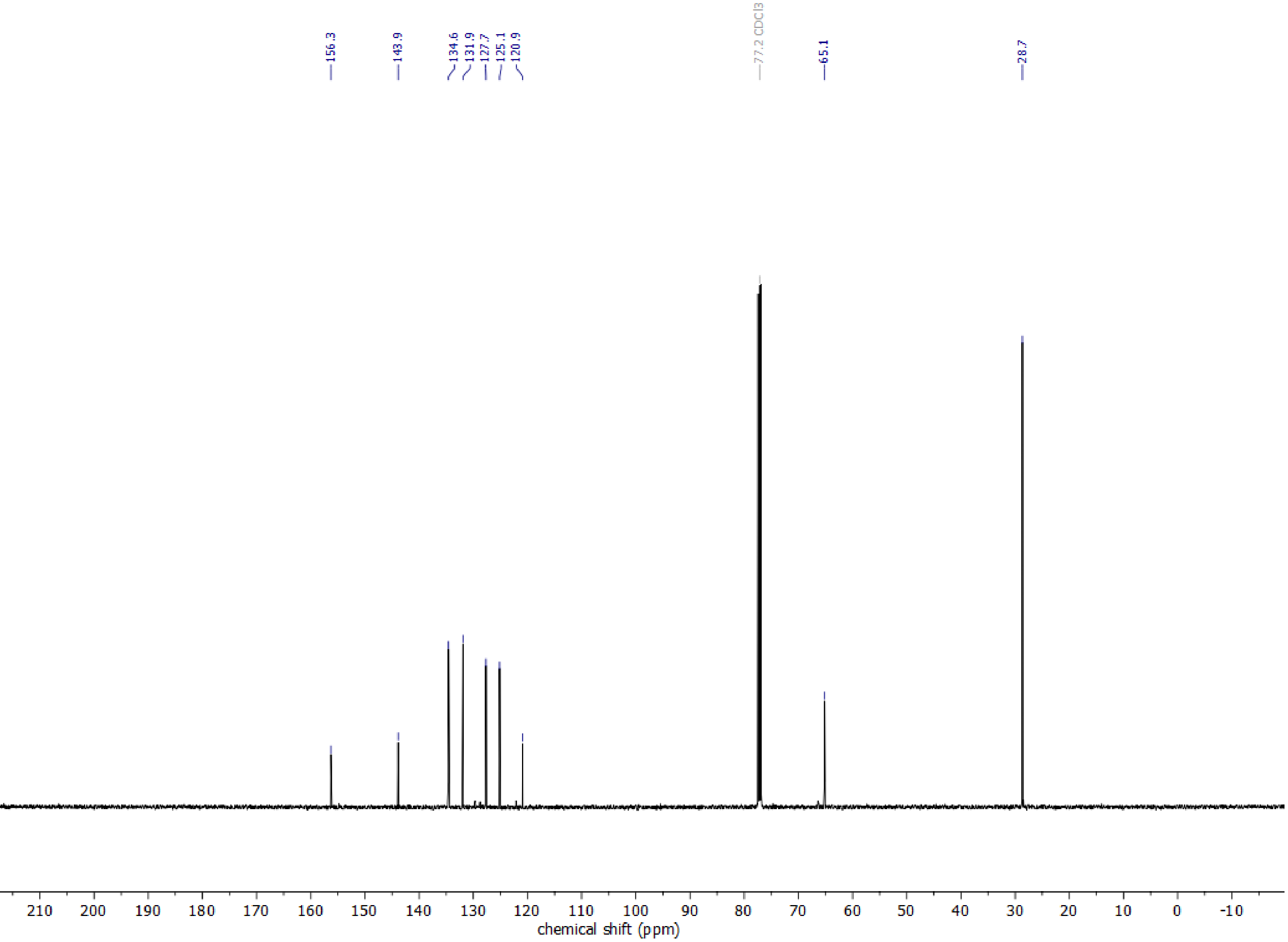

^{1}H NMR spectrum of reduced Flutamide S1

DMSO-d_6, 25°C, 500 MHz

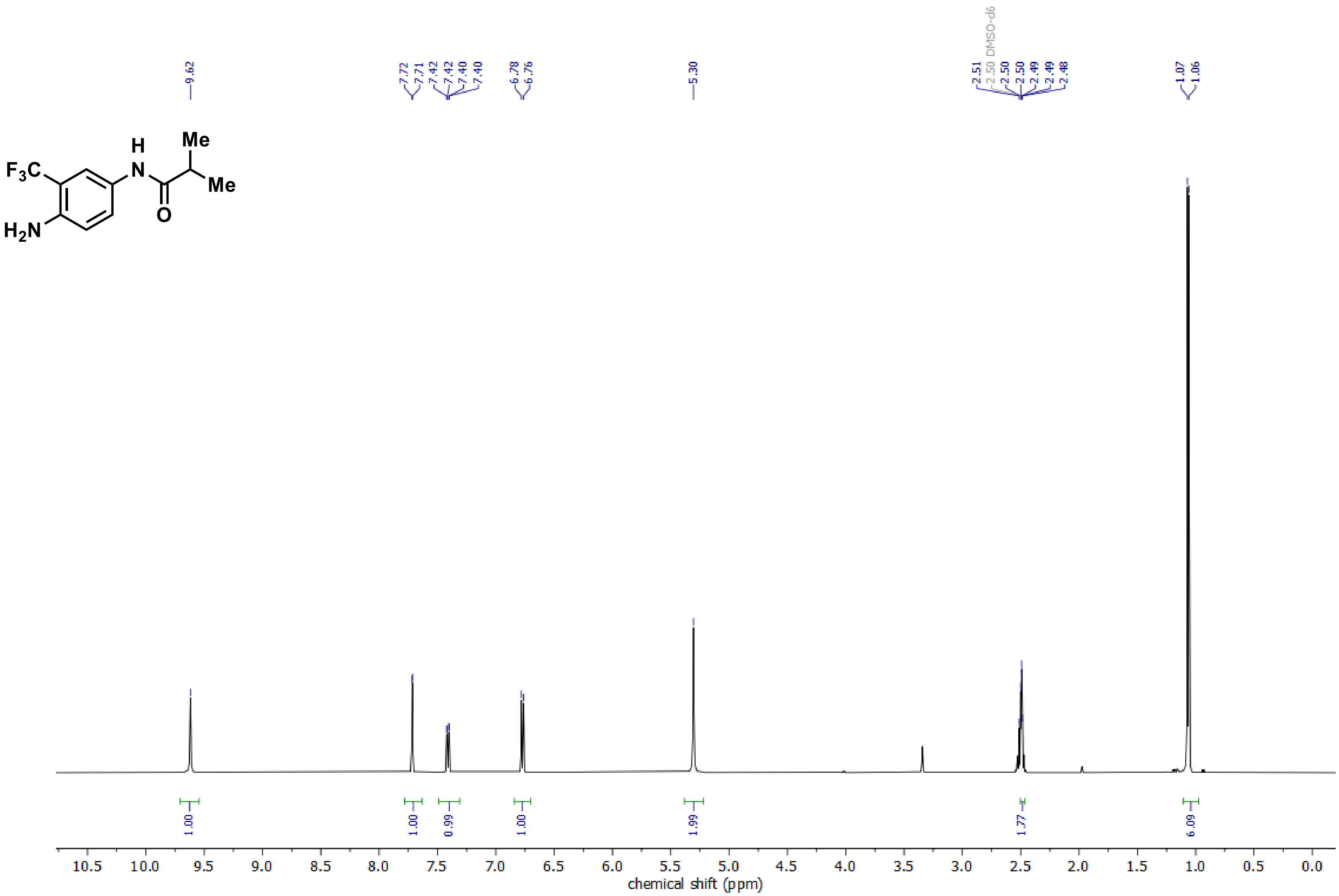

^{19}F NMR spectrum of reduced Flutamide S1

DMSO-d_6, 25°C, 470 MHz

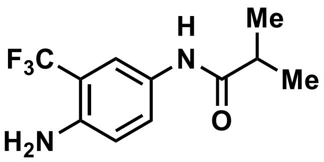

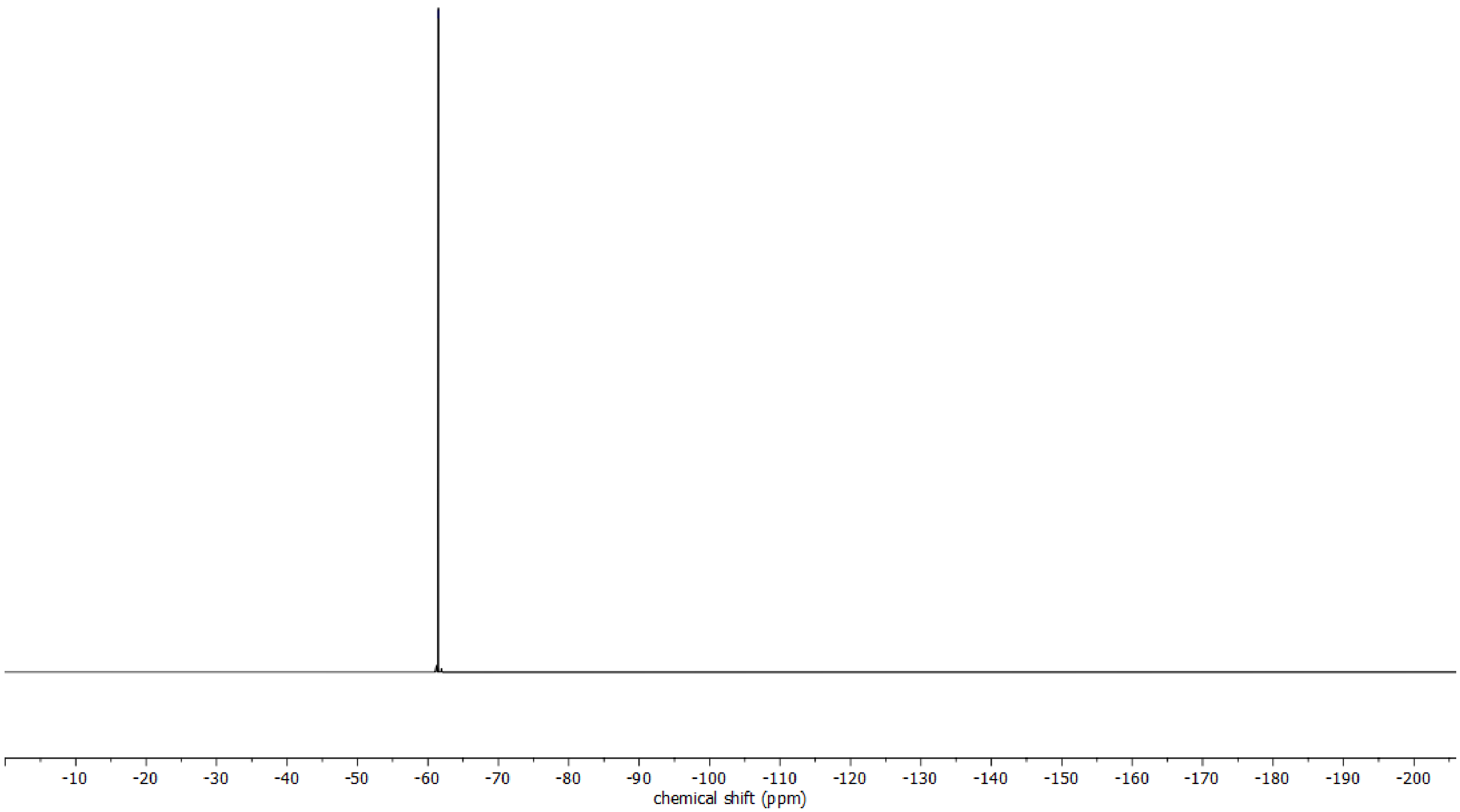

^{13}C NMR spectrum of reduced Flutamide S1

DMSO-d_6, 25°C, 125 MHz

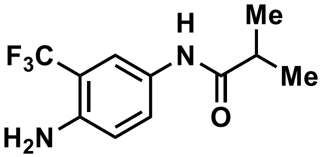

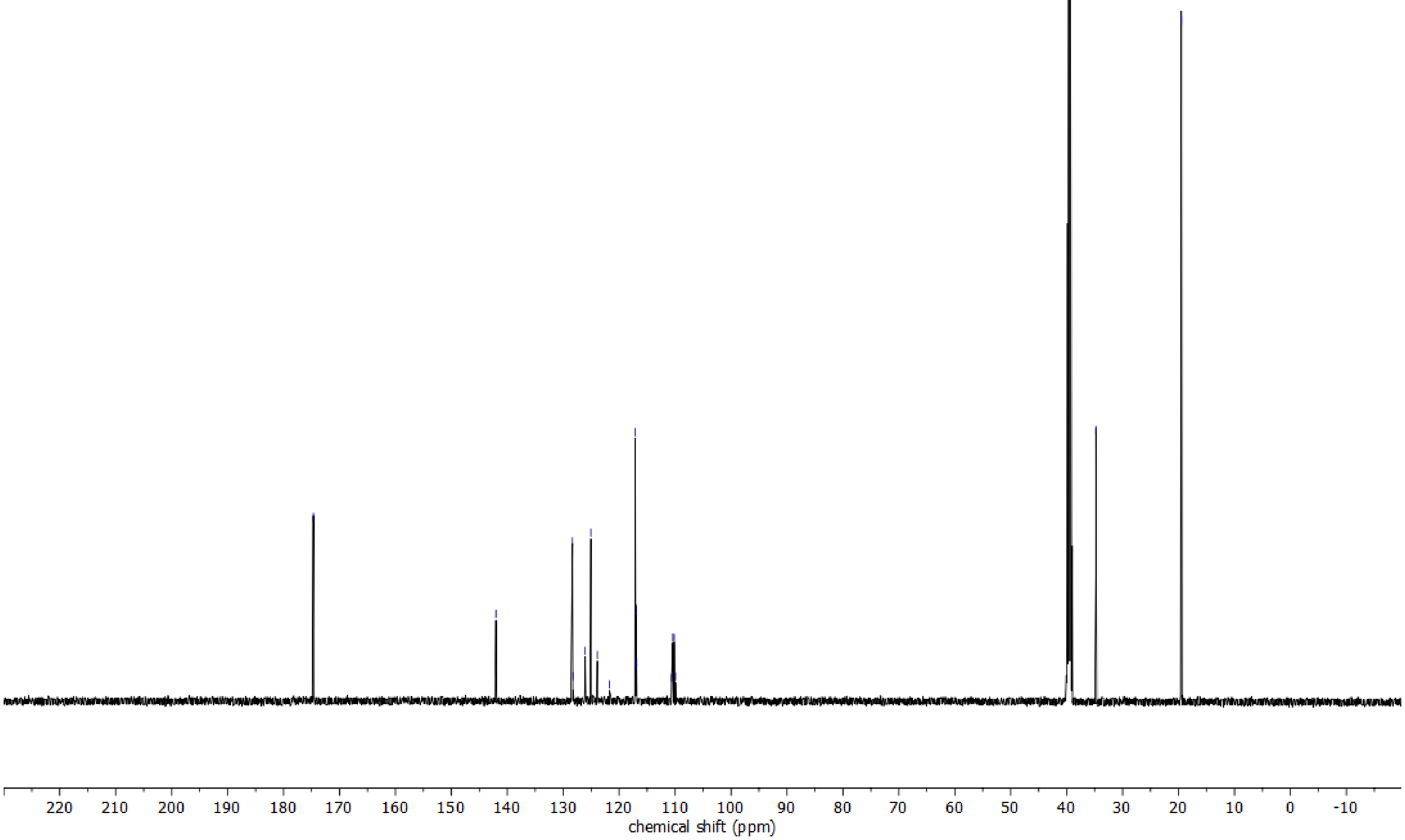

^{1}H NMR of spectrum of [18-crown-6-K]$^{15}NO_3$

CD_3CN, 25°C, 600 MHz

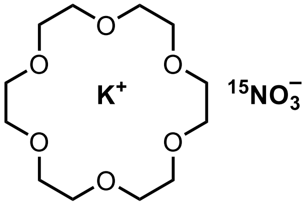

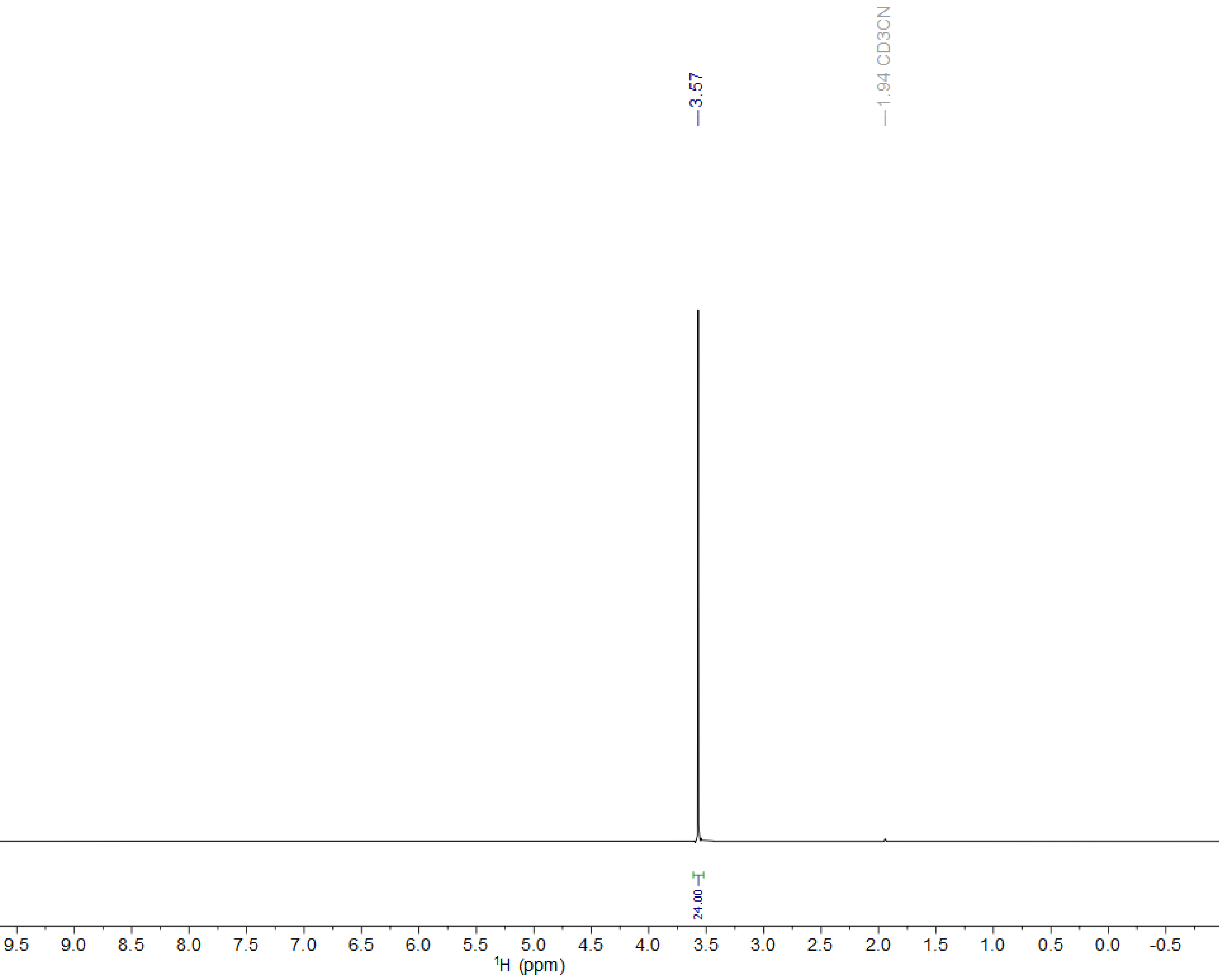

^{13}C NMR of spectrum of [18-crown-6-K]$^{15}NO_3$

CD$_3$CN, 25°C, 151 MHz

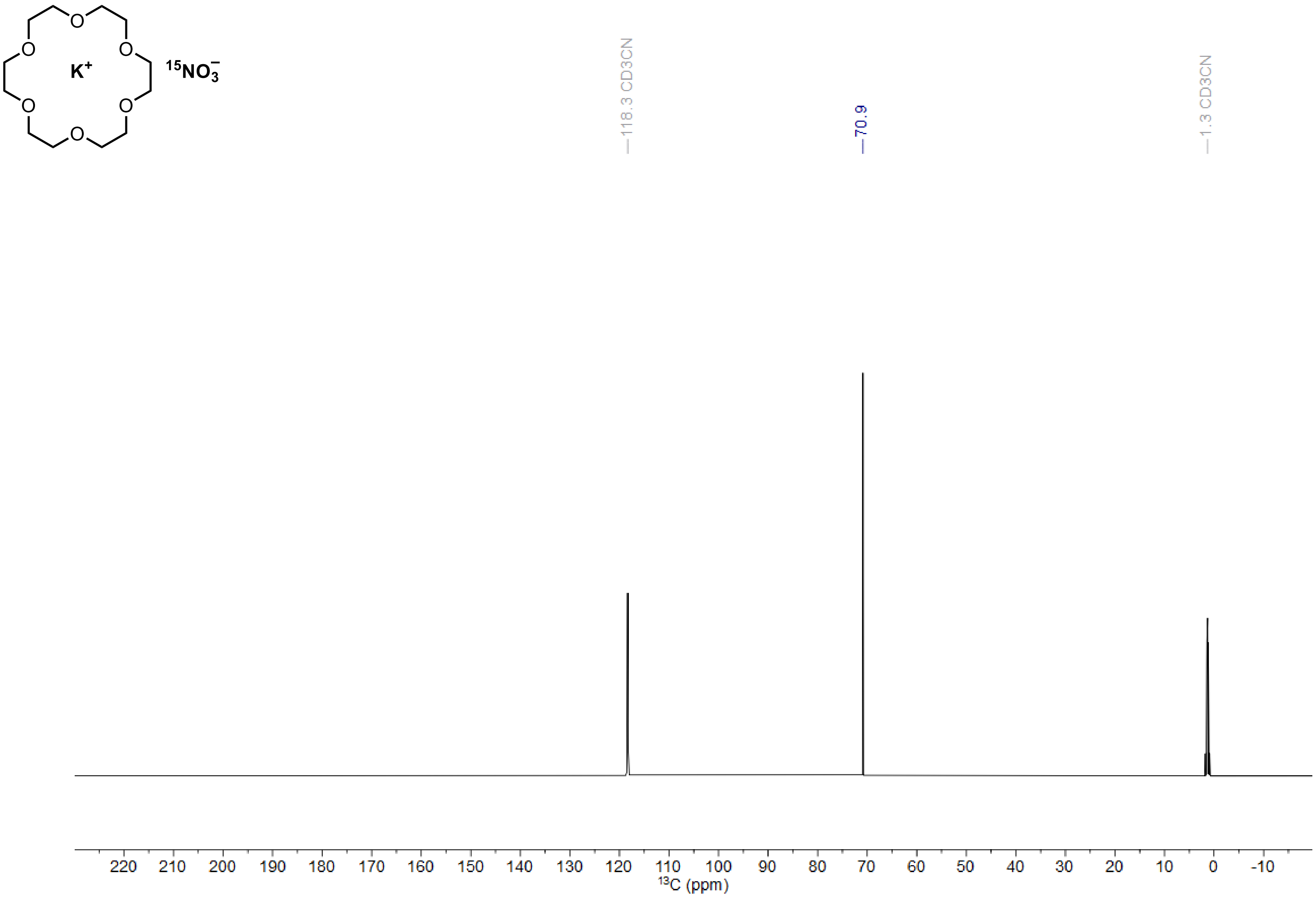

^{15}N NMR of spectrum of [18-crown-6-K]$^{15}NO_3$

CD_3CN, 25°C, 61 MHz

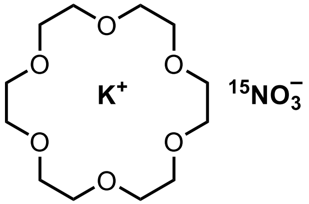

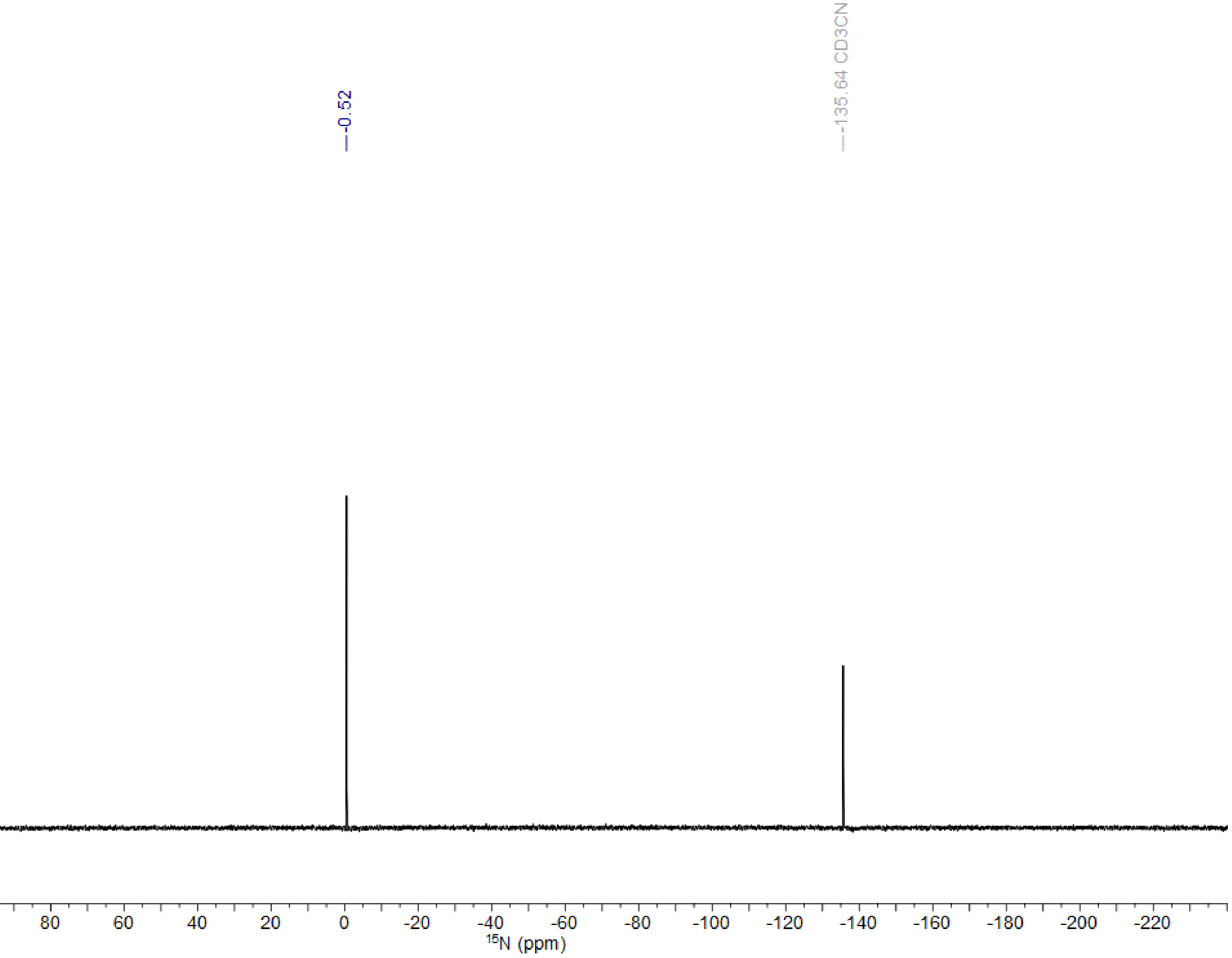

^{39}K NMR of spectrum of [18-crown-6-K]$^{15}NO_3$

CD_3CN, 25°C, 19 MHz

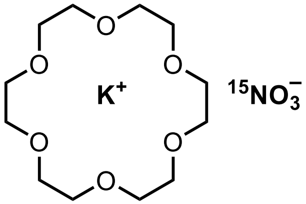

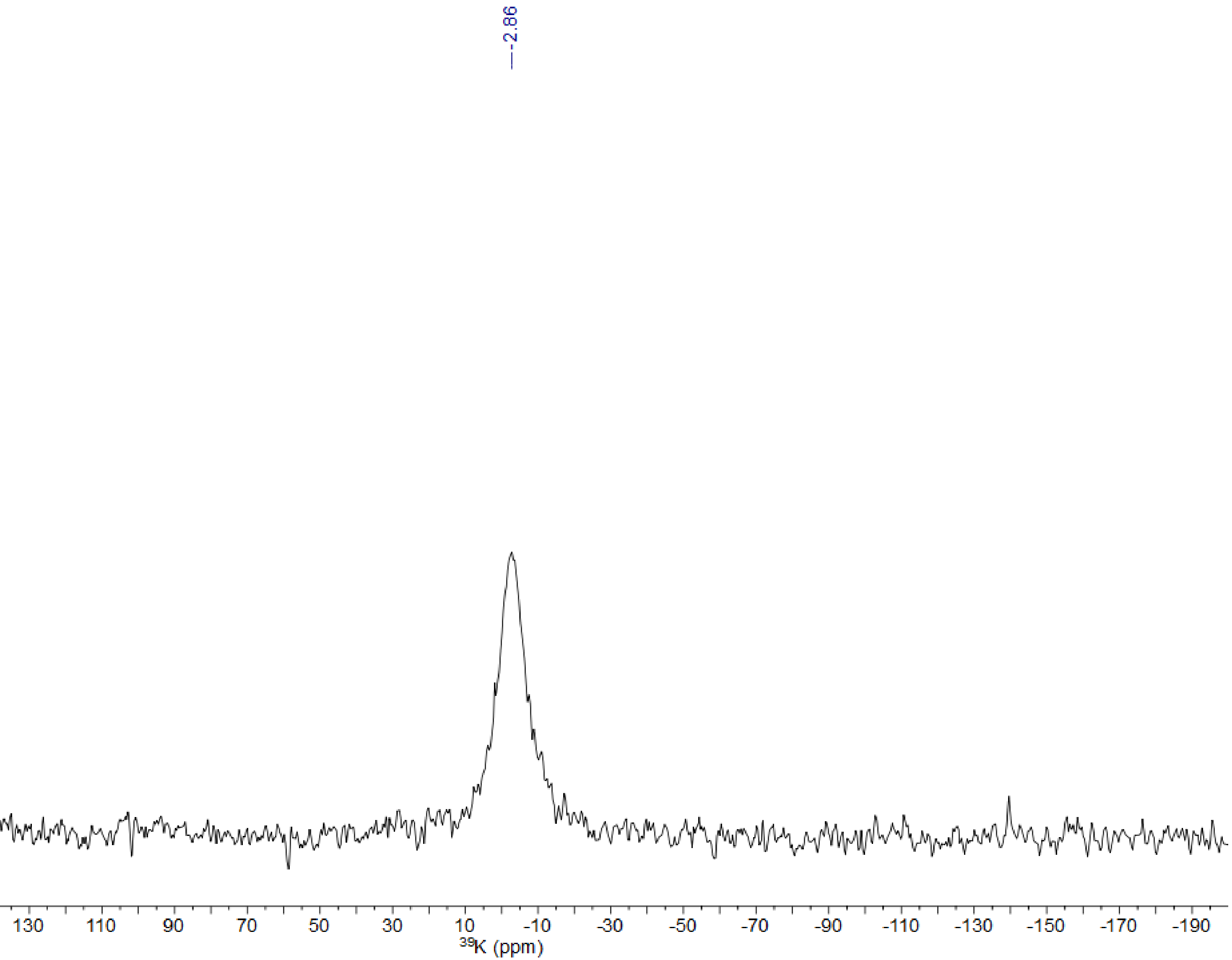

REFERENCES

1. Harris, R. K.; Becker, E. D.; De Menezes, S. M. C.; Granger, P.; Hoffman, R. E.; Zilm, K. W. Further conventions for NMR shielding and chemical shifts (IUPAC Recommendations 2008). *Pure Appl. Chem.* **2008**, *80* (1), 59–84.
2. Fulmer, G. R.; Miller, A. J. M.; Sherden, N. H.; Gottlieb, H. E.; Nudelman, A.; Stoltz, B. M.; Bercaw, J. E.; Goldberg, K. I. NMR Chemical Shifts of Trace Impurities: Common Laboratory Solvents, Organics, and Gases in Deuterated Solvents Relevant to the Organometallic Chemist. *Organometallics* **2010**, *29* (9), 2176–2179.
3. Neese, F., The ORCA program system. *Wiley interdiscip. Rev. Comput. Mol. Sci.* **2012,** *2* (1), 73–78.
4. Neese, F., Software update: the ORCA program system, version 4.0. *Wiley interdiscip. Rev. Comput. Mol. Sci.* **2018,** *8* (1), e1327.
5. Zhao, Y.; Truhlar, D. G., The M06 suite of density functionals for main group thermochemistry, thermochemical kinetics, noncovalent interactions, excited states, and transition elements: two new functionals and systematic testing of four M06-class functionals and 12 other functionals. *Theor. Chem. Acc.* **2008,** *120* (1), 215–241.
6. Weigend, F., Accurate Coulomb-fitting basis sets for H to Rn. *Phys. Chem. Chem. Phys.* **2006,** *8* (9), 1057−1065.
7. Weigend, F.; Ahlrichs, R., Balanced basis sets of split valence, triple zeta valence and quadruple zeta valence quality for H to Rn: design and assessment of accuracy. *Phys. Chem. Chem. Phys.* **2005,** *7* (18), 3297−3305.
8. Marenich, A. V.; Cramer, C. J.; Truhlar, D. G., Universal solvation model based on solute electron density and on a continuum model of the solvent defined by the bulk dielectric constant and atomic surface tensions. *J. Phys. Chem. B* **2009,** *113* (18), 6378−6396.
9. . Raghavachari, K.; Trucks, G. W.; Pople, J. A.; Head-Gordon, M. A Fifth-Order Perturbation Comparison of Electron Correlation Theories. *Chem. Phys. Lett.* 1989, *157*, 479–483.
10. Kendall, R. A; Thom H. Dunning, T. H., Jr.; Harrison, R. J. Electron affinities of the first-row atoms revisited. Systematic basis sets and wave functions, *J. Chem. Phys.* **1992**, *96*, 6796–6806.
11. 11. Woon, D. E.; Dunning, T. H., Jr. Gaussian Basis Sets for Use in Correlated Molecular Calculations. II. The Atoms Aluminum Through Argon. *J. Chem. Phys.* **1993**, *98*, 1358-1371.
12. 12. Altun, A.; Neese, F.; Bistoni, G. Open-Shell Variant of the London Dispersion-Corrected Hartree–Fock Method (HFLD) for the Quantification and Analysis of Noncovalent Interaction Energies. *J. Chem. Theory Comput.* **2022**, *18 (4)*, 2292-2307.
13. *Prudent practices in the laboratory: handling and management of chemical hazards, updated version*. (National Academies Press, Washington, DC, 2011).
14. Mateos, J.; Schulte, T.; Behera, D.; Leutzsch, M.; Altun, A.; Sato, T.; Waldbach, F.; Schnegg, A.; Neese, F.; Ritter, T., Nitrate reduction enables safer aryldiazonium chemistry. *Science* **2024**, 384 (6694), 446-452.

15. Sharma, R.; Sahu, B.; Mali, S. V.; Singh, D.; Kumar, P. B.; Dawange, M.; Mistry, H. Preparation of ROR gamma modulators and therapeutic uses thereof. WO2015145371, 2015.
16. Koo, B. A.; Nam, W. H.; Hong, W. S.; Seo, H. S. Substituted 1H-pyridin-2-one derivatives useful as inhibitors of PDE4, and their preparation, pharmaceutical compositions, and use, particularly for treatment of chronic obstructive pulmonary disease (COPD). WO2004050624, 2004.
17. Morrow BJ, Camerino MA, Walker SR, Stevenson GI, Stupple PA, inventors; CTXT Pty Ltd, assignee. Substituted condensed thiophenes as modulators of sting. WO2019219820, 2019.
18. Bakthavatchalam, R.; Blum, C. A.; Chenard, B. L., Substituted bicyclic quinazolin-4-ylamine derivatives. WO2005023807, 2005.
